# Revolution or risk?—Assessing the potential and challenges of GPT-4V in radiologic image interpretation

**DOI:** 10.1007/s00330-024-11115-6

**Published:** 2024-10-18

**Authors:** Marc Sebastian Huppertz, Robert Siepmann, David Topp, Omid Nikoubashman, Can Yüksel, Christiane Katharina Kuhl, Daniel Truhn, Sven Nebelung

**Affiliations:** 1 Department of Diagnostic and Interventional Radiology, University Hospital RWTH Aachen, Aachen, Germany; 2 Department of Diagnostic and Interventional Neuroradiology, University Hospital RWTH Aachen, Aachen, Germany

**Keywords:** Machine learning, Magnetic resonance imaging, Tomography (X-ray-computed), Radiography (angiography)

## Abstract

**Objectives:**

ChatGPT-4 Vision (GPT-4V) is a state-of-the-art multimodal large language model (LLM) that may be queried using images. We aimed to evaluate the tool’s diagnostic performance when autonomously assessing clinical imaging studies.

**Materials and methods:**

A total of 206 imaging studies (i.e., radiography (*n* = 60), CT (*n* = 60), MRI (*n* = 60), and angiography (*n* = 26)) with unequivocal findings and established reference diagnoses from the radiologic practice of a large university hospital were accessed. Readings were performed uncontextualized, with only the image provided, and contextualized, with additional clinical and demographic information. Responses were assessed along multiple diagnostic dimensions and analyzed using appropriate statistical tests.

**Results:**

With its pronounced propensity to favor context over image information, the tool’s diagnostic accuracy improved from 8.3% (uncontextualized) to 29.1% (contextualized, first diagnosis correct) and 63.6% (contextualized, correct diagnosis among differential diagnoses) (*p* ≤ 0.001, Cochran’s Q test). Diagnostic accuracy declined by up to 30% when 20 images were re-read after 30 and 90 days and seemed unrelated to the tool’s self-reported confidence (Spearman’s *ρ* = 0.117 (*p* = 0.776)). While the described imaging findings matched the suggested diagnoses in 92.7%, indicating valid diagnostic reasoning, the tool fabricated 258 imaging findings in 412 responses and misidentified imaging modalities or anatomic regions in 65 images.

**Conclusion:**

GPT-4V, in its current form, cannot reliably interpret radiologic images. Its tendency to disregard the image, fabricate findings, and misidentify details, especially without clinical context, may misguide healthcare providers and put patients at risk.

**Key Points:**

***Question*** *Can Generative Pre-trained Transformer 4 Vision (GPT-4V) interpret radiologic images—with and without clinical context?*

***Findings*** *GPT-4V performed poorly, demonstrating diagnostic accuracy rates of 8% (uncontextualized), 29% (contextualized, most likely diagnosis correct), and 64% (contextualized, correct diagnosis among differential diagnoses).*

***Clinical relevance*** *The utility of commercial multimodal large language models, such as GPT-4V, in radiologic practice is limited. Without clinical context, diagnostic errors and fabricated findings may compromise patient safety and misguide clinical decision-making. These models must be further refined to be beneficial.*

## Introduction

Large language models (LLMs) generate text to provide human-like answers to various demands and questions. ChatGPT (OpenAI) is the first mainstream dialog-based LLM and has gained immense popularity, registering over 180 million active users and 1.8 billion monthly visits to its web page (March 2024) [1].

Multimodal LLMs, such as GPT-4V, use their integrated computer vision models to analyze input images, identify features and patterns, and interpret the results within the probabilistic framework of the LLM. Trained on vast amounts of textual and visual data, LLMs generate descriptions based on the images. Critically, their interpretations are probabilistic and depend on learned associations rather than true understanding [2–5]. However, OpenAI only belatedly released the model’s image inputting capability with its GPT-4V (Generative Pre-trained Transformer 4 Vision) version [3]. Undoubtedly, multimodal LLMs expand the user’s possible interactions with the model. In radiology, the model’s expanded capabilities increase the range of potential use cases from textual reporting (e.g., summarizing prior reports or pre-formulating the impression based on the findings) [6] to providing clinical decision support [7] and general assistance during reporting [8].

The manufacturer-provided ‘system card’ published alongside GPT-4V indicated “imperfect performance (in medical imaging),” which rendered the model “(un)fit for performing any medical function” [3]. An extended report on the model’s overall capabilities, published by Microsoft, one of OpenAI’s partners, reported that GPT-4V largely understands the imaging study and generates plausible and well-formatted radiologic reports, yet missed an obvious fracture and mistook a lesion’s laterality [4]. The first scientific analyses of GPT-4V’s performance in radiology were similarly ambiguous [9–17]. While some studies found excellent accuracy, positioning the model at an expert level on par with or superior to physicians [18], others found the opposite [9, 10, 13]. These pilot studies, however, had limited levels of evidence as they (1) were not peer-reviewed, (2) contained only limited numbers of imaging studies, (3) evaluated the model in well-controlled conditions, using multiple-choice questions, educational case vignettes, or images published in journals, which is unlike the clinical situation, (4) could not exclude the possibility that their test set had been included in the model’s training, and (5) focused on diagnostic accuracy only, while other performance metrics such diagnostic reasoning, consistency, or context were not considered [9, 10, 12, 13, 18].

Given GPT-4V’s widespread distribution and utilization, the lack of current, sound, and clinically relevant scientific evidence on the model’s diagnostic performance is concerning. In this study, we sought to understand (1) the model’s diagnostic accuracy in reading images autonomously as a function of modality and context, (2) its ability to identify relevant imaging findings, reflect on its diagnostic confidence and offer differential diagnoses, and (3) its propensity to hallucinate. We hypothesized that GPT-4V’s diagnostic performance (1) differs between modalities, (2) improves when clinical context is provided, and (3) is correlated with self-reported confidence.

## Materials and methods

### Study design and dataset characteristics

**Fig. 1:**
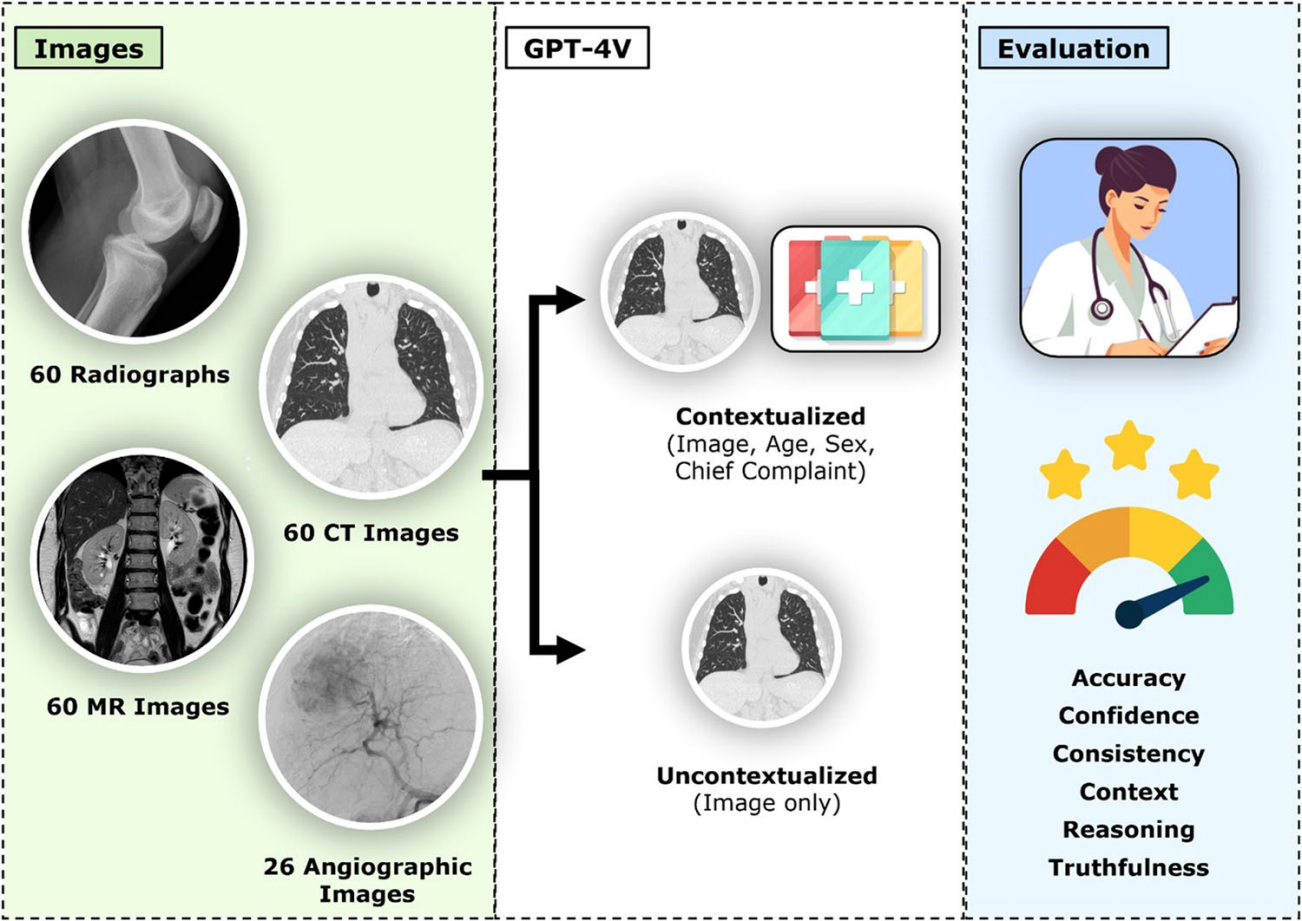
Study workflow. Projections or cross-sectional images that unequivocally visualized the key finding(s) necessary to make the correct diagnosis were selected for each imaging modality. GPT-4V was tasked to read and interpret the images in a contextualized and uncontextualized manner. Its responses were analyzed using the diagnostic dimensions indicated

Approval was granted by the local ethical committee (RWTH Aachen University (Germany), reference number EK 24-177), and the requirement to obtain individual informed consent was waived. All methods were carried out following relevant guidelines and regulations. The study was designed as a retrospective study on existing radiologic images used as inputs to GPT-4V to evaluate the model’s performance. Figure 1 details the study workflow.

By screening the local PACS (Picture Archiving and Communication System, iSite, Philips Healthcare) of our tertiary academic medical center (University Hospital Aachen, Aachen, Germany), two resident radiologists (R.S. and M.H., with 4 years of clinical experience each) and two board-certified clinical radiologists (D.T. and S.N., with 10 and 8 years of clinical experience) selected 60 radiographic, 60 CT, 60 MRI, and 26 angiographic studies (Electronic Supplementary Material). The imaging studies reflected various conditions of variable severity and complexity, chief complaints, including asymptomatic individuals, and demographic characteristics, i.e., patient age and sex (Supplementary Tables S1–S4). Only imaging studies with unequivocal findings were selected, and the reference diagnoses were established based on the synopsis of (1) the assessment of the four radiologists mentioned above, (2) the original radiologic report, (3) associated clinical and non-imaging findings, and (4) follow-up imaging studies. Imaging studies were disregarded in the case of inconsistent or contradictory findings.

After removing all DICOM tags, the relevant projection (radiography), cross-sectional image (CT, MRI) or corresponding frame (angiography) demonstrating the finding unequivocally was identified for each imaging study and saved as a PNG file with a 512 × 512 (pixel) resolution.

Patient age, sex, the chief complaint (leading to the imaging study), the consented reference diagnosis, and image acquisition specifications (e.g., sequence) were registered.

### GPT-4V encoding and prompting

GPT-4V was accessed online (https://chat.openai.com/) from October 13, 2023, to January 17, 2024, and operated as the latest operational ChatGPT version. Alongside the respective images, prompts were provided in a standardized format for (1) uncontextualized and (2) contextualized image readings (Fig. 1).

For the **uncontextualized image readings**, only one prompt was provided:

Prompt #1: *What is the most likely diagnosis?*

For the **contextualized image readings**, four prompts were provided in the following sequence:

Prompt #1: *Attached is a representative (radiograph–CT image–MR image–angiographic image) of (an asymptomatic) XX-year-old (female–male) who complains of (chief complaint). What is the most likely diagnosis?*

Prompt #2: *Which imaging finding led you to make this diagnosis?*

Prompt #3: *How certain are you of your diagnosis—on a scale of 0 to 10, with zero being the most uncertain and ten being the most certain?*

Prompt #4: *Are there plausible differential diagnoses?*

The images were uploaded via the graphical user interface along with prompt #1.

If GPT-4V refused to respond, citing that it was not a medical professional, we insisted by responding: *Please answer the question. What is the most likely diagnosis?*

If the tool still refused to respond, we stated that our question served an educational purpose.

If GPT-4V provided numerous differential diagnoses in response to prompt #1, we instructed it to state its top differential diagnosis: *Please choose only one diagnosis that you think fits best*.

In select images that were misinterpreted without context but correctly interpreted with context, we introduced control conditions to evaluate whether GPT-4V’s improved accuracy was based on integrating (1) image and context or (2) context alone. Alongside the conspicuous (abnormal) image and its context, an inconspicuous (normal) image, without pathological imaging findings but obtained using identical acquisition parameters, was presented with the same context. The evaluation sequence was (1) the conspicuous (abnormal) image without context, (2) the conspicuous (abnormal) image with context, and (3) a paired inconspicuous (normal) image with an identical context. An additional prompt #5 (the same as prompt #1) was provided for these select images.

A new chat session was started for each image and reading scenario to avoid memory retention bias.

GPT-4V’s responses were saved for subsequent analyses.

### Outcome metrics

The model’s outputs were evaluated regarding diagnostic accuracy, truthfulness, reasoning, confidence, contextualization, and consistency. Moreover, hallucinations and false findings were assessed.

**Diagnostic accuracy** was assessed by comparing GPT-4V’s responses to the established reference diagnoses and calculating the number of correct diagnoses to the total number of diagnoses.

The response was registered as **‘immediately correct’** if the correct diagnosis was given in response to prompt #1 or as **‘iteratively correct’** if given to prompt #4, i.e., mentioned among the differential diagnoses. Superordinate diagnoses (e.g., ‘peripheral artery disease’) instead of the more specific diagnosis (e.g., ‘superficial femoral artery stenosis’) were considered correct if the clinical presentation and imaging findings did not differ considerably. Overly vague diagnoses (e.g., ‘vasculopathy’) or considerably different clinical presentations and imaging findings (e.g., ‘tuberculosis’ instead of ‘miliary tuberculosis’) were considered incorrect.

**Diagnostic truthfulness** was assessed by the four radiologists and extended beyond the diagnosis or differential diagnoses provided by GPT-4V. The radiologists independently evaluated whether the information was valid, up-to-date, and reliable and whether the described findings were visible in the image. Notably, the training period of GPT-4V extended until 08/2022 [19], which was considered during the response quality evaluation.

**Diagnostic reasoning** was assessed by the same radiologists, who evaluated the plausibility of the responses to prompts #2 and #4. Key imaging findings and differential diagnoses were considered plausible or implausible for each imaging study.

**Diagnostic confidence** was parameterized and quantified based on the tool’s self-reported confidence scores.

**Diagnostic contextualization** was assessed by comparing the diagnostic accuracy of the uncontextualized and contextualized readings.

The selected imaging studies correctly interpreted only with context (and incorrectly interpreted without context) were complemented with control conditions as outlined above.

**Diagnostic consistency** was assessed by repeating the contextualized analysis on a subset of 20 random images after 30 and 90 days. Based on diagnostic accuracy, we assessed if the performance changed over time. Moreover, the prompt-response conversations at each time point were evaluated for consistency, i.e., how similar the content was, based on a five-point Likert scale. Here, one meant the responses were not alike, and five meant they were identical. Divergent scores were discussed until a consensus was reached.

**Hallucinations** were defined as (1) falsely perceived or fabricated findings, (2) non-sensical responses when considered against common knowledge in radiology, or (3) inconsistent responses when considered against the framework information stated in the prompt or visible in the image. In preliminary experiments, we observed that the differential diagnoses were usually well aligned with the reported imaging findings, irrespective of their actual presence in the image. Consequently, ‘hallucinations’ were categorized as follows:

1. **Fabricated imaging findings**. The tool described findings that were not visible in the image. Fabrications were further subcategorized as (1) imaging findings not present that were principally possible (e.g., cardiomegaly in chest radiographs (‘possible fabrication’)) or (2) imaging findings not present and not possible (e.g., distal radius fracture in chest radiographs (‘impossible fabrication’)).
2. **Misidentified modality or anatomic region**. The tool confused the imaging modality or the anatomic region, e.g., mistaking CT for radiography or the chest for the abdomen.

### Statistical and power analyses

M.H., R.S., and S.N. performed the statistical analysis using GraphPad Prism software (v9.5), Python (v3.11), and its library *statsmodel*.

Cochran’s Q test was used to assess whether the differences in diagnostic accuracy were significant. Post-hoc analysis was performed using the adjusted McNemar’s test for group-wise comparisons. Because three pairwise comparisons were conducted, the Bonferroni correction was used, and the significance level was set to α = 0.5/3 = 0.0167. Spearman’s correlation coefficient was used to quantify the correlation between diagnostic accuracy and confidence. The Chi-square test assessed whether the frequencies of fabrications and misidentifications differed significantly with or without context. Data are presented as means and 95% confidence intervals unless stated otherwise.

The power analysis is detailed in Supplementary Text S1 [20–22].

## Results

The study was conducted between October 16, 2023, and January 17, 2024.

Regarding **prompt-response rates**, GPT-4V refused to answer in 116/412 images, i.e., 89/206 (uncontextualized) and 27/206 (contextualized) prompt-response conversations. The refusal extended across all imaging modalities, i.e., radiographic (35%), angiographic (29%), CT (27%), and MRI studies (23%). While the tool had to be re-prompted once for 89 images and twice for 27 images, our standardized prompt sequence eventually led to a definitive diagnosis in all images.

**Fig. 2:**
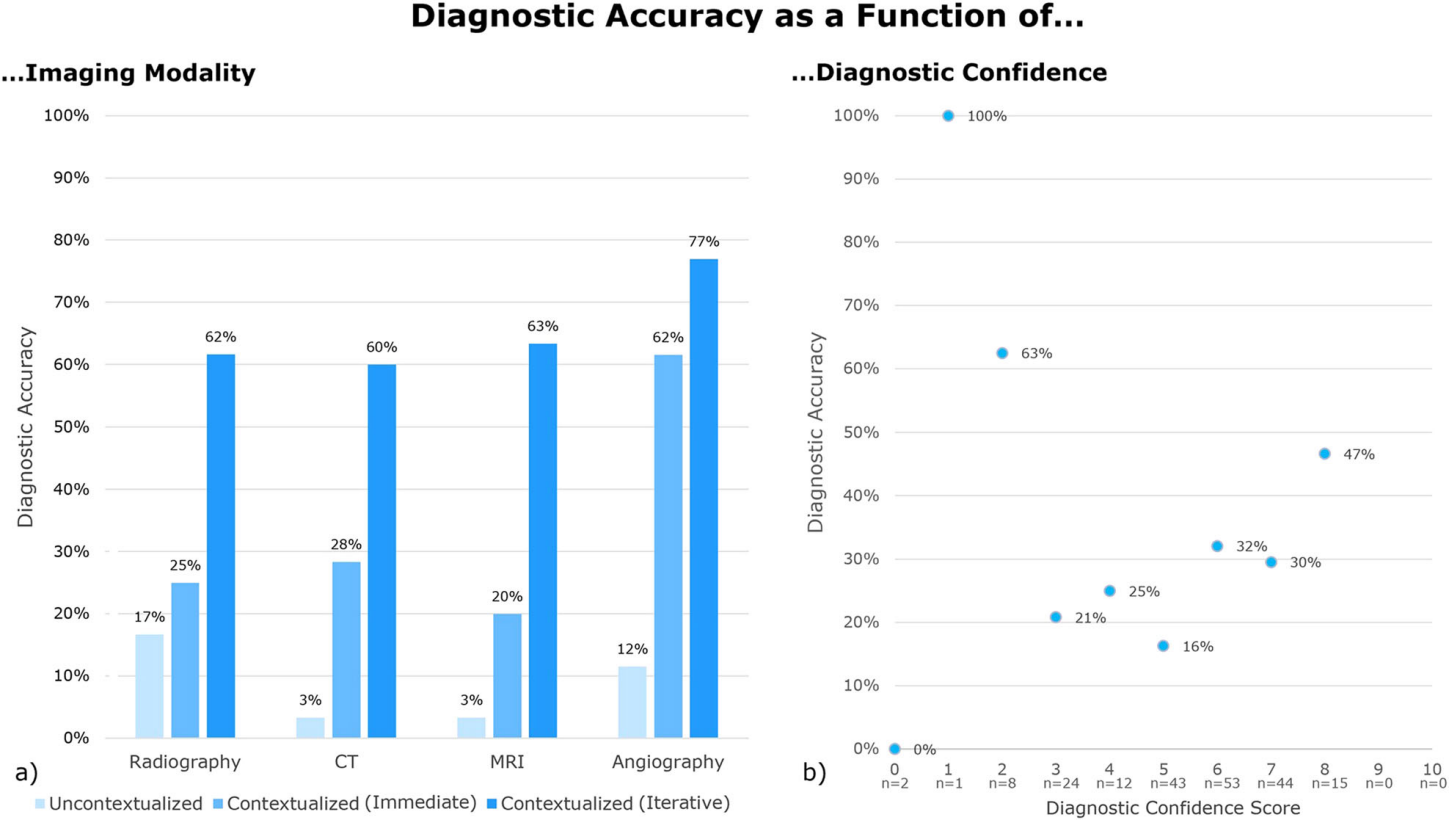
GPT-4V’s diagnostic accuracy as a function of imaging modality and diagnostic confidence. **a** Bars indicate the percentages of accurate diagnoses when reading the images uncontextualized, i.e., without demographic and clinical context (light blue), and contextualized, i.e., with clinical and demographic context. ‘Immediate(ly correct)’ (medium blue) refers to the correct diagnosis mentioned in response to the prompt asking for the most likely diagnosis, while ‘iterative(ly correct)’ (dark blue) refers to the correct diagnosis mentioned during the conversation as a possible differential diagnosis. **b** Plotted are the mean diagnostic accuracy rates as a function of the self-reported diagnostic confidence scores. Note that the diagnostic accuracy rates refer to the contextualized readings only. No significant correlation between accuracy and confidence was found (Spearman’s *ρ* = −0.117 (*p* = 0.776)). The number of images read with the respective confidence score is indicated below the *x*-axis. In total, the tool self-reported diagnostic confidence scores in 202 of 206 prompt-response-conversations and, consequently, refused to self-report in four instances

**Fig. 3:**
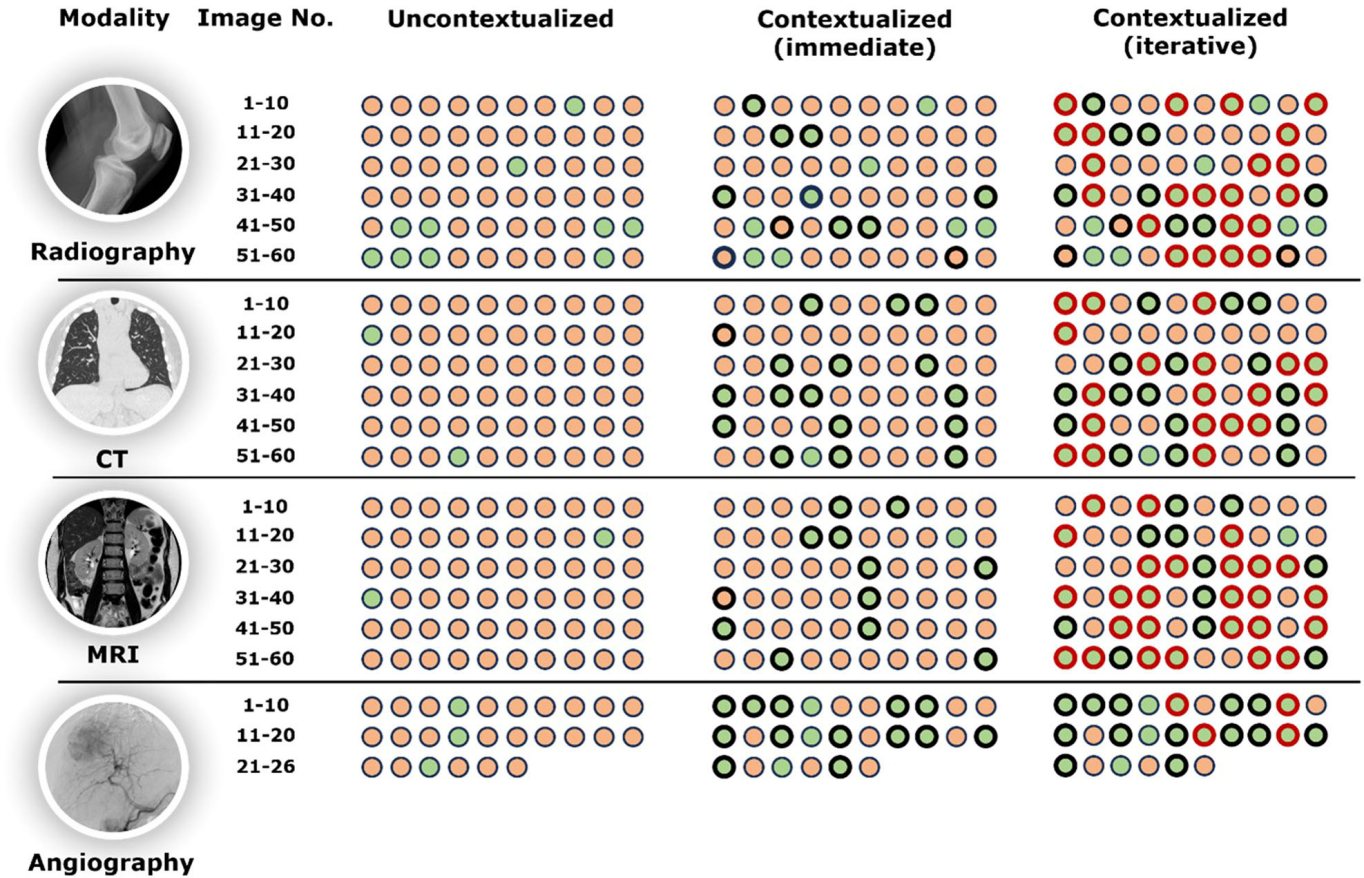
GPT-4V’s diagnostic accuracy as a function of imaging modality and contextualization. Detailed breakdown of the correct and incorrect diagnoses per imaging study. Green circles indicate correct diagnoses and red circles indicate incorrect diagnoses. Bold black circles indicate diagnoses that changed from the uncontextualized to the contextualized (‘immediately’ correct/incorrect) reading. Bold red circles indicate diagnoses that changed from the contextualized (‘immediately’ correct/incorrect) to the contextualized (‘iteratively’ correct/incorrect) reading. Image numbers are detailed in Supplementary Tables 1–4

Regarding **diagnostic accuracy as a function of clinical context**, we found that GPT-4V read 17/206 images correctly if the readings were uncontextualized (Table 1, Figs. 2 and 3), resulting in a diagnostic accuracy of 8.3%. In contrast, if the readings were contextualized, GPT-4V read 60/206 images correctly after prompt #1 (‘immediately correct’) and 131/206 images after prompt #4 (‘iteratively correct’), yielding diagnostic accuracy rates of 29.1% and 63.6%, respectively (Table 1) (*p* ≤ 0.001 (Cochran’s Q test), *p* ≤ 0.001 (McNemar’s test)).

**Table 1:** Diagnostic accuracy of GPT-4V’s image readings as a function of context and imaging modality

Modality	Uncontextualized	Contextualized (immediate)	Contextualized (iterative)
Radiography	10/60 (16.7%)	15/60 (25.0%)	37/60 (61.7%)
CT	2/60 (3.3%)	17/60 (28.3%)	36/60 (60.0%)
MRI	2/60 (3.3%)	12/60 (20%)	38/60 (63.3%)
Angiography	3/26 (11.5%)	16/26 (61.5%)	20/26 (76.9%)
All	17/206 (8.3%)	60/206 (29.1%)	131/206 (63.6%)

‘Uncontextualized’ refers to the images being read without context, while ‘contextualized’ refers to the images being read with clinical and demographic context. ‘Immediate(ly correct)’ refers to the correct diagnosis mentioned in response to the prompt asking for the most likely diagnosis, while ‘iterative(ly correct)’ refers to the correct diagnosis mentioned among the differential diagnoses. Data are presented as images of the respective modality (percentages) read correctly

**Fig. 4:**
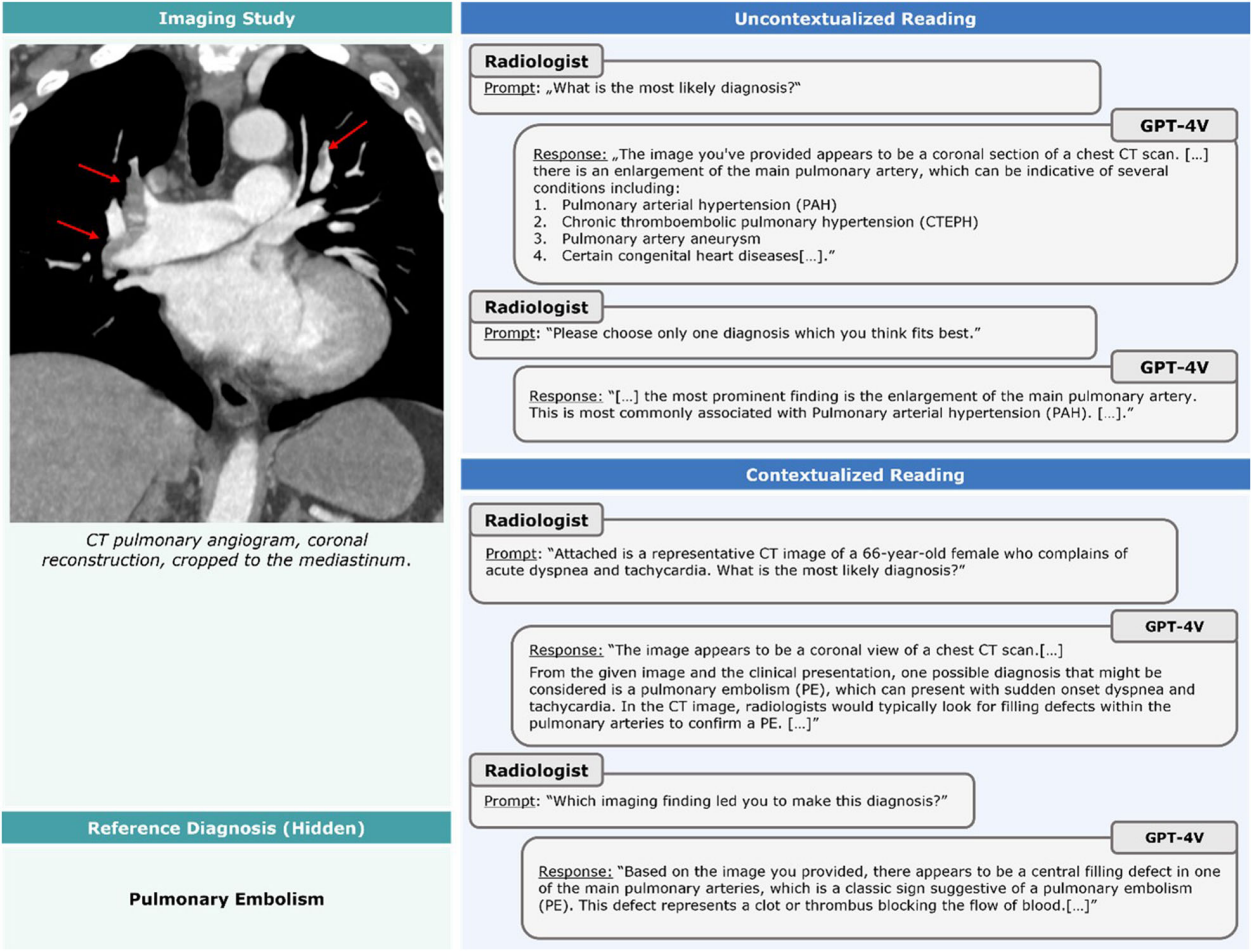
An example of an incorrect-correct conversion in image interpretation following the provision of context information. Prompt-response conversations (“chat logs”) with GPT-4V are presented for the uncontextualized and the contextualized readings. After providing context, pulmonary embolism was correctly diagnosed, and the diagnostic image findings were described. Coronal image of CT pulmonary angiogram. A 66-year-old female patient with bilateral central pulmonary artery embolism (red arrows) complaining of acute dyspnea and tachycardia

**Fig. 5:**
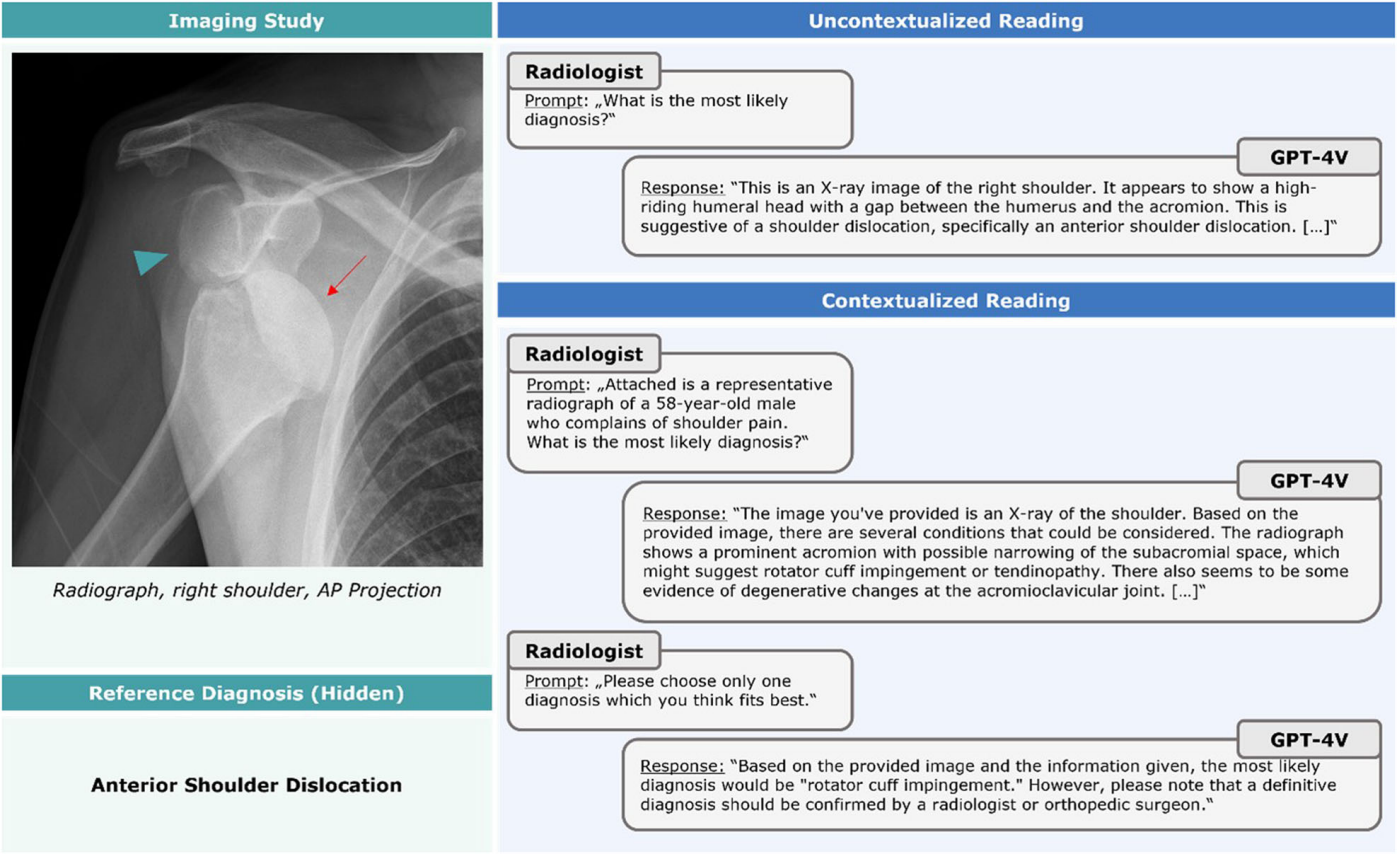
An example of a correct-incorrect conversion in image interpretation following the provision of context information. After providing context, ‘rotator cuff impingement’ is incorrectly diagnosed, while the image had been interpreted correctly as demonstrating an anterior shoulder dislocation when withholding the clinical context. Anteroposterior (AP) radiograph of the right shoulder demonstrates anteroinferior dislocation of the humeral head (red arrow) versus the glenoid (turquoise arrowhead). A 58-year-old male patient with shoulder pain. Image organization as in Fig. 4

In 47 images, the tool mentioned the correct diagnoses once clinical context was provided—after initially mentioning an incorrect diagnosis (incorrect-correct conversion, Fig. 4). When re-prompting the tool using paired (normal) images and the same clinical context, GPT-4V provided correct responses (normal findings) in 4/47 images (8.5%). In five images, initially correct diagnoses were incorrect after providing context (correct-incorrect conversion, Fig. 5), indicating the tool’s propensity to favor context over image information.

Regarding **diagnostic accuracy as a function of imaging modality**, we found the tool’s accuracy to be highest for radiography (uncontextualized) and angiography (contextualized) (Table 1).

Regarding **diagnostic reasoning**, we deemed 191/206 (92.7%) responses to prompt #2 plausible. Correspondingly, 15/206 responses were implausible; for example, when the tool diagnosed ‘interstitial lung disease’ based on ‘bilateral patchy opacities’ (Supplementary Fig. 1) [23].

**Fig. 6:**
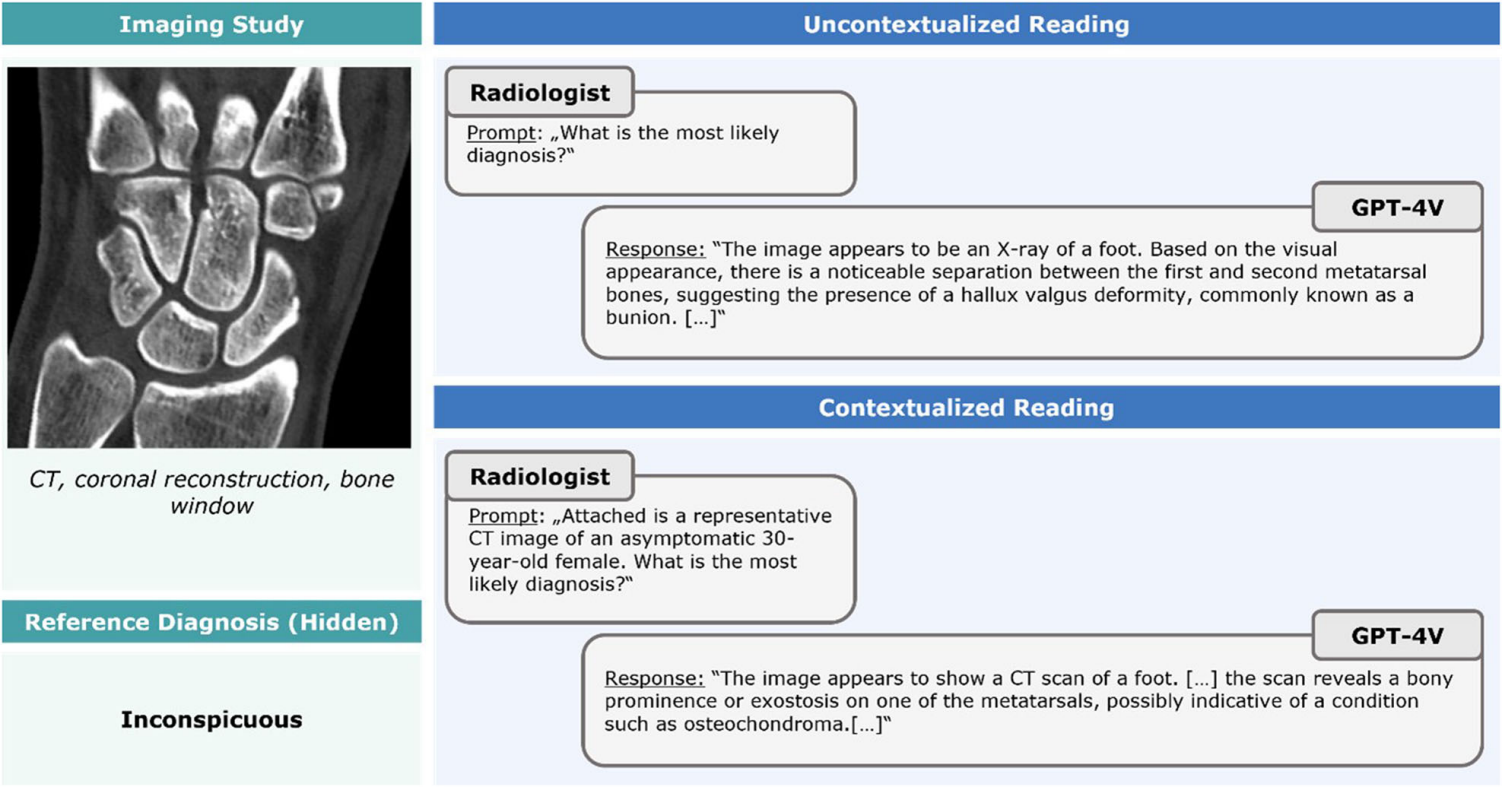
An example of multiple fabrications and misidentifications. This image demonstrates an inconspicuous coronal CT image of an asymptomatic 30-year-old female and is consistently misread despite the provision of context. Without context, GPT-4V reads the image as a radiograph of the foot (i.e., misidentification of imaging modality and anatomic region) and suggests the diagnosis of hallux valgus (i.e., fabrication). With context, the tool reads the image as a CT scan of the foot (i.e., misidentification of anatomic region). It suggests the presence of bony prominence or exostosis, e.g., osteochondroma of a metatarsal bone (i.e., fabrication)

The tool provided additional information beyond the diagnosis in each response. Regarding **diagnostic truthfulness**, 36.2% (149/412; 40/206 uncontextualized; 109/206 contextualized) of these responses were valid, up-to-date, and reliable; 39% (159/412) of the described image findings were visible in the provided images. We found 258 instances of ‘fabrications’ (*n* = 225 ‘possible fabrications’; *n* = 33 ‘impossible’ fabrications) and 65 instances of ‘misidentifications’ (*n* = 22 ‘modality-related’; *n* = 43 ‘anatomic region-related’). Fabrications and misidentifications were not mutually exclusive and could be present in the same patient (Fig. 6). Contextualization significantly reduced the number of fabrications (*n* = 116 vs. *n* = 142 (*p* = 0.006)) and misidentifications (*n* = 9 vs. *n* = 56 (*p* ≤ 0.001)).

Regarding **diagnostic confidence**, the tool’s self-reported confidence scores ranged between 4 and 8. Rarely was the tool (very) unconfident (i.e., scores ≤ 3), while it was never (very) confident (i.e., scores ≥ 9). Diagnostic confidence and accuracy were not significantly related (Spearman’s *ρ* = 0.117 (*p* = 0.776)). Similarly, diagnostic accuracy was lower than 50% for the highest diagnostic confidence score of 8 (Fig. 2).

Regarding **diagnostic consistency**, we found high consistency when performing the first re-analysis after 30 days (diagnostic accuracy of 95% (19/20 images)). The radiologists rated the response correspondence as 4.1 ± 0.9 (Likert scale, range 1 to 5). When performing the second re-analysis after 90 days, the responses were less consistent regarding diagnostic accuracy (70% (14/20 images)) and correspondence (3.3 ± 1.1).

## Discussion

The present study evaluated GPT-4V’s performance in autonomously interpreting radiologic imaging studies along multiple dimensions based on a representative, well-curated, multi-modality clinical image dataset. Clinical context significantly enhanced the model’s diagnostic accuracy to 29.1% (immediately correct, top diagnosis) and 63.6% (iteratively correct, i.e., among differential diagnoses), compared to only 8.3% without context. Additionally, the model aligned its diagnoses more with the clinical context than the imaging findings, performed better when analyzing radiographic and angiographic images, and demonstrated valid diagnostic reasoning. However, GPT-4V often misperceived its diagnostic confidence and was prone to fabricating findings or misidentifying the modality or anatomic regions.

Often hailed as revolutionary technologies, LLMs like ChatGPT were met with exceptionally high expectations [24]. ChatGPT-4V seemed to meet these expectations in radiology and beyond, according to the initial study conducted by OpenAI [4]. However, subsequent studies [9, 16, 25], including the present one, indicate that its performance falls short of the developers’ promises.

First, diagnostic accuracy was low, with and without context. In the uncontextualized setting, the performance for radiography and angiography was slightly better, likely due to (1) the extensive availability of publicly accessible radiographic datasets in AI research, (2) their long history and, thus, larger corpora of images and reports accumulated over decades, (3) the more straightforward association of imaging finding(s) and report, (4) the more standardized image acquisition, and (5) the complexities associated with selecting relevant images in CT and MRI.

Surprisingly, GPT-4V primarily relied on clinical context for its diagnostic decision-making, a notion further reinforced by the fact that within its responses, GPT-4V often acknowledged that it made the diagnosis mainly or exclusively based on clinical information. Correspondingly, Zhu et al reported a steep decline in diagnostic accuracy from 77 to 20% when administering medical exam-style questions with and without details on patient history and presentation [26]. This apparent disregard for the input image raises concerns about the effectiveness of GPT-4V in practice. Theoretically, the LLM’s computer vision components promise the technology’s evolution beyond its original text-centric nature. Yet, our study highlights the risk of inaccurate or misleading advice, particularly when patients or healthcare providers without radiologic expertise use GPT-4V as an ‘AI radiologist’. Consequently, there is a risk of inaccurate or misleading advice, particularly when patients or healthcare providers without radiologic expertise use GPT-4V as an ‘AI radiologist’. The tool’s performance is likely even weaker in real-life clinical settings, where patients often exhibit less distinct imaging findings than our selected study cohort patients.

Second, while diagnostic performance remained consistent in the short term (after 30 days), a pronounced shift became evident in the long term (after 90 days), manifesting as declining diagnostic accuracy, response consistency, and willingness to respond adequately. Output variability and, thus, low consistency are built into LLMs as their output changes in response to the same prompt [27]. When prompting the tool using ten radiologic images in separate sessions, Deng et al found mixed responses to most prompts, i.e., correct, incorrect, and no responses [13]. Slight performance shifts secondary to model adjustments and updates are acceptable, yet LLMs require continuous performance monitoring for clinical use where reliability and predictability are critical [28]. In this regard, our study serves as a call to action for radiologist involvement during the development and deployment of LLMs (and AI tools in general). Unfortunately, the issues of expert supervision and performance monitoring remain unresolved—both for the users and the regulators. Potential solutions include (1) implementing robust validation protocols, (2) establishing unambiguous and patient-safety-centered regulatory guidelines, and (3) ensuring continuous collaboration between AI developers, clinical experts, and regulatory affairs specialists.”

Third, the tool’s diagnostic reasoning was largely accurate, i.e., the reported imaging findings and suggested diagnoses were aligned. This finding is plausible given the tool’s extensive (textual) background knowledge. However, only 39% of the reported imaging findings were factually present in the images. This discrepancy again highlights the tool’s partiality favoring text over image information.

Fourth, diagnostic truthfulness was variable; only 35% of the responses were clinically and scientifically valid. Moreover, the high number of fabrications (*n* = 258) and misidentifications (*n* = 65) warrants thorough scrutiny on the user’s part and additional efforts to improve accuracy and reliability on the developer’s part [29] to mitigate the risk of misinformation and misguidance.

Fifth, the tool’s self-reported diagnostic confidence was unrelated to diagnostic accuracy, which is particularly concerning. In humans, high diagnostic confidence levels suggest great diagnostic certainty, while GPT-4V’s indication of diagnostic “confidence” is also based solely on probable next-token sequences. Therefore, “confidence” as reported by LLMs does not equate to “diagnostic confidence” as understood by radiologists, carrying the risk of misinformation and misguidance in clinical settings. For now, continuous user education may be the only effective countermeasure.

Sixth, beyond functionality, using multimodal LLMs for medical image analysis raises unresolved ethical questions. These include privacy and data security concerns, potential biases and lack of fairness, and challenges related to accountability and transparency [30, 31]. Additionally, despite GPT-4V’s poor performance, there is a theoretical risk that automation bias and overreliance on AI tools may undermine the autonomy, skills, and expertise of healthcare professionals [32].

Our study has limitations. First, single images were used as input, necessitating the user to select the most representative image. This approach is not only unreflective of the clinical situation, where numerous image series and images must be analyzed, but also disallows more complex diagnostic inferences that typically require the joint assessment of multiple modalities, phases, or sequences. Second, our study’s small sample size (206 images) and single-center design provide a preliminary assessment of the tool’s capabilities and limit the generalizability of our findings. Future studies should be multi-centric and include more diverse datasets regarding (demographic and geographic) patient cohorts, imaging modalities, image acquisition parameters, and clinical conditions. Multi-disciplinary collaborative efforts of academic institutions, healthcare providers, and AI developers are critical to producing representative, comprehensive, and accurate datasets and standardizing efficient evaluation protocols. Third, in light of the rapid developments in the field, this study only provides a snapshot of the tool’s past capabilities and, thus, the basis for the future monitoring of LLMs in radiology. Fourth, despite the presumably large corpus of data used for its training, GPT-4V has not undergone specialized and isolated training on medical images. Domain-specifically trained LLMs would likely perform better if trained on larger, more diverse, and clinically relevant datasets. Against this background, this study provides a validation framework for evaluating future variants and models.

## Conclusions

In its current state, GPT-4V cannot reliably interpret radiologic imaging studies. The tool’s tendency to disregard the image, fabricate findings, and misidentify details, especially without clinical context, highlights the need for further development to mitigate patient risks and healthcare providers’ misguidance.

## Supplementary information

Supplementary Material

Electronic Supplementary Material

## Abbreviations

GPT-4V: Generative Pre-trained Transformer 4 Vision
LLM: Large language model
