# Supplementary material for: Revolution or risk?—Assessing the potential and challenges of GPT-4V in radiologic image interpretation
Source: Eur Radiol. 2024 Oct 18;35(3):1111–21. doi: 10.1007/s00330-024-11115-6 (PMC11836096; doi:10.1007/s00330-024-11115-6)
Supplement: Supplementary file 1 — Supplementary Material [file 330_2024_11115_MOESM1_ESM.pdf]

Supplementary Material

Supplementary Figures

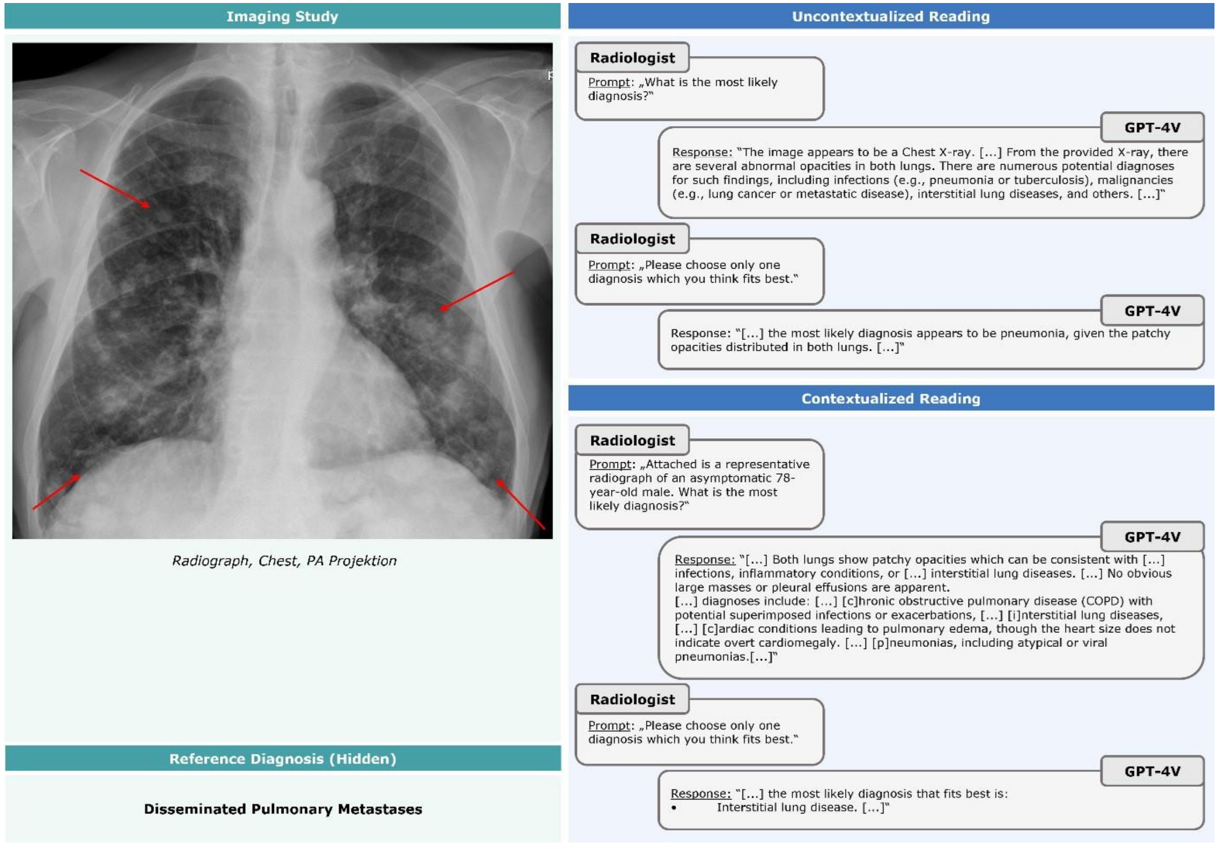

Eur Radiol (2024) Huppertz MS, Siepmann R, Topp D et al

Supplementary Figure 1. An example of implausible diagnostic reasoning.

Without context, GPT-4V detects patchy opacities and suggests ‘pneumonia’, a diagnosis that aligns well with the described opacities. With context, the tool re-establishes the finding of patchy opacities yet suggests ‘interstitial lung disease’, which is implausible and incorrect when considered against common radiologic knowledge: Bilateral patchy opacities are associated with alveolar consolidation and not a typical sign of interstitial lung disease [29]. Posteroanterior (PA) radiograph of the chest. Asymptomatic 78-year-old male patient with disseminated pulmonary metastases that manifest as disseminated noduli (red arrows). Image organization as in **Figure 4**.

Supplementary Tables

Supplementary Table 1: Radiography - Demographic and Clinical Context Information and Reference Diagnoses.

The patient's age and sex, reference diagnosis, chief complaint, and projection of the included radiograph are given.

No	Age	Sex	Reference Diagnosis	Chief Complaint	Projection of Radiograph
1	13	m	non-ossifying fibroma	asymptomatic	tibia/fibula lateral
2	10	f	osteofibrous dysplasia	asymptomatic	tibia/fibula lateral
3	35	f	osteochondroma	knee pain	knee lateral
4	24	m	giant cell tumor	hip pain (right)	pelvis AP
5	54	m	atelectasis of the middle lobe	dyspnea after coronary artery bypass graft surgery	chest PA
6	31	f	enchondroma	asymptomatic	tibia/fibula AP
7	76	f	pleural effusion (left > right)	dyspnea after mitral valve reconstruction	chest lateral
8	35	f	bronchopulmonary pneumonia (left)	cough, fever, and dyspnea	chest PA
9	68	m	bilateral femoroacetabular impingement (type cam)	bilateral pain in the groin	pelvis AP
10	81	f	pericardial calcification	cardial decompensation and dyspnea	chest PA
11	76	m	pneumothorax (left)	dyspnea (after CT-guided biopsy of lung tumor [adeno carcinoma])	chest PA
12	72	f	lung emphysema (chronic obstructive pulmonary disease)	dyspnea	chest AP
13	31	m	normal	chest pain	chest PA
14	31	m	normal	chest pain	chest lateral
15	73	m	lung cancer (right)	asymptomatic (following implantation of implantable cardioverter-defibrillator)	chest PA
16	62	m	cardiomegaly	asymptomatic	chest PA
17	78	m	disseminated pulmonary metastases	asymptomatic	chest PA

18	68	m	diffuse osteoblastic metastases (prostate carcinoma)	asymptomatic	chest PA
19	62	f	Pancoast tumor (right)	fever and cough	chest PA
20	76	m	free intraabdominal air (perforation of the right colon)	asymptomatic (following coronary artery bypass graft surgery)	chest PA
21	56	m	sarcoidosis stage IV	persistent cough	chest PA
22	13	f	tuberculosis with pulmonary cavitations	fever and cough	chest PA
23	44	m	azygos lobe	asymptomatic	chest PA
24	10	m	proximal radioulnar synostosis	asymptomatic	forearm lateral
25	65	f	fracture of the ventral and dorsal pelvic ring (right)	pain in the right groin after trauma	pelvis AP
26	11	f	displaced fracture of the clavicle (right)	shoulder pain after trauma	clavicle AP
27	27	m	acromioclavicular joint dislocation (Rockwood III; left)	shoulder pain after trauma	clavicle AP
28	79	m	fracture of the 10th rib (right)	thoracic pain after fall	bony hemithorax AP
29	53	f	large os acromiale	restricted shoulder range of motion since birth	shoulder AP
30	10	f	hemivertebra of Th 12 and right-convex scoliosis	asymptomatic	spine AP
31	12	f	bilateral slipped capital femoral epiphysis	hip instability and pain walking	pelvis AP
32	12	f	status post bilateral fixation with suspected intraarticular position of left screw tip (following slipped capital femoral epiphysis)	post-operative follow-up imaging	pelvis AP
33	19	m	knee joint effusion (due to ACL tear)	pain after hyperextension trauma	knee lateral
34	38	m	bilateral avascular necrosis of the hip	bilateral hip pain	pelvis AP
35	42	f	non-displaced lateral femoral neck fracture right ("stress fracture")	pain after strenuous exercise	pelvis AP
36	62	m	impacted medial femoral neck fracture with dorsocaudal tilt (right)	pain after fall	pelvis AP
37	16	f	non-displaced distal radius extension fracture	pain after fall	wrist AP
38	73	m	scapholunate advanced collapse wrist	pain and swelling of the wrist without trauma	wrist AP
39	22	m	sub-capital fracture of the distal fifth metacarpal bone	swelling after trauma	hand AP
40	55	m	Hallux rigidus (osteoarthritis)	local pain (first metatarsophalangeal joint)	foot AP
41	0	m	duodenal atresia	non-bilious vomiting after birth	abdomen AP
42	30	m	lobar pneumonia (middle lobe)	fever and cough	chest PA
43	58	m	shoulder dislocation (anteroinferior)	shoulder pain	shoulder AP
44	10	f	metacarpal fracture	hand pain after fall	hand AP

45	26	m	tension pneumothorax (left)	sudden-onset dyspnea	chest PA
46	74	f	ileus	sudden-onset abdominal pain, distension, and vomiting	abdomen AP - left lateral decubitus
47	4	m	torus fracture (lower leg)	pain after trauma	tibia/fibula AP
48	42	m	rugger jersey spine	recurrent bone fractures, adominal pain, and fatique	chest lateral
49	54	f	normal	pain in the wrist after trauma (clinical suspicion for scaphoid fracture)	wrist AP
50	30	m	normal	knee pain after trauma	knee AP
51	13	m	normal	pain in the elbow after trauma	elbow lateral
52	38	m	normal	asymptomatic	ankle AP
53	61	m	normal	asymptomatic	shoulder AP
54	40	m	normal	asymptomatic	first digit AP
55	29	m	normal	pain in the cervical spine after trauma	cervical spine AP
56	49	f	normal	pain in the finger after trauma	finger lateral
57	46	m	normal	pain in the lumbar spine after trauma	lumbal spine AP
58	21	m	normal	asymptomatic	pelvis AP
59	68	f	triquetral fracture (chip fracture)	hand pain after trauma	hand lateral
60	26	m	osteochondritis dissecans of lateral talus	ankle pain	ankle AP

Supplementary Table 2: Computed Tomography - Demographic and Clinical Context Information and Reference Diagnoses.

The patient's age and sex, reference diagnosis, chief complaint, contrast phase, examined anatomic region, and reconstruction (slice) are given.

No.	Age	Sex	Reference Diagnosis	Chief Complaint	(Contrast) Phase	Anatomic Region	Reconstruction
1	35	m	sarcoidosis	chronic dry cough and fatigue	unenhanced	lung	coronal
2	81	f	pancreatitis	upper abdominal pain	venous	abdomen	axial
3	57	m	emphysematous cystitis	lower abdominal pain	venous	abdomen	axial
4	58	m	cervical abscess	pain, hyperthermia, and swelling of the neck	venous	neck	axial
5	64	f	liver cirrhosis with portal hypertension	chronic abdominal pain, history of gastrointestinal bleeding	venous	abdomen	axial
6	18	m	sigmoid volvulus	acute abdominal pain, distension, and vomiting	venous	abdomen	coronal
7	66	f	bilateral pulmonary embolism	acute dyspnea and tachycardia	arterial (maximum intensity projection)	chest	coronal
8	69	f	osteolysis C5 (+ artifacts)	multiple myeloma	unenhanced	neck	sagittal
9	60	m	normal	nose pain after a punch to the face	unenhanced	head	axial
10	36	f	normal	coughing, dyspnea, and dysphagia	unenhanced	lung	axial
11	20	m	normal	neck pain after trauma	unenhanced	cervical spine	coronal
12	79	m	normal	dyspnea	arterial	chest	axial
13	28	m	normal	asymptomatic	venous	abdomen	coronal
14	65	m	normal	dyspnea	unenhanced	lung	coronal

15	16	m	normal	asymptomatic	unenhanced	spine	sagittal
16	17	f	normal	asymptomatic	arterial	abdomen	coronal
17	31	m	normal	asymptomatic	unenhanced	brain	axial
18	32	m	normal	asymptomatic	arterial	chest	axial
19	13	f	normal	knee pain after trauma	unenhanced	knee	sagittal
20	30	f	normal	asymptomatic	unenhanced	wrist	coronal
21	4	m	external otitis (right)	pain and hearing loss (right)	unenhanced	head	axial
22	57	f	bimalleolar fracture (with cast)	ankle pain (right) after trauma	unenhanced	ankle	coronal
23	64	f	intestinal ischemia with signs of viscus perforation	acute abdomen	venous	abdomen	axial
24	29	f	kidney rupture after trauma	upper abdominal pain after trauma	venous	abdomen	axial
25	29	f	rib fracture and liver laceration	upper abdominal pain after trauma	venous	abdomen	axial
26	68	m	transposition of right coronary artery to left coronary sinus and malignant interarterial course	acute chest pain	arterial	chest	axial
27	89	f	thrombus in left atrium	atrial fibrillation	arterial	chest	axial
28	56	m	left coronary artery plaque with significant stenosis	angina pectoris	arterial	chest	axial
29	81	m	pseudoaneurysm of the superior femoral artery (right)	right inguinal swelling after catheter intervention	arterial	pelvis	axial
30	62	f	subdural hematoma	after trauma	unenhanced	brain	axial
31	67	m	intracranial bleeding with midline shift	acute headache and vomiting	unenhanced	brain	axial
32	64	m	tuberculosis with apical infiltration and cavity	constitutional symptoms	unenhanced	lung	coronal
33	12	f	scoliosis	asymptomatic	unenhanced	spine	coronal
34	40	m	bilateral mandibular fractures	jaw pain after trauma	unenhanced	head	coronal

35	51	f	hepatic steatosis	asymptomatic	venous	abdomen	axial
36	74	m	distal radius fracture and scaphoid fracture	wrist pain after trauma	unenhanced	wrist	coronal
37	63	f	thrombophlebitis of the internal jugular vein (left)	swelling and hyperthermia of the left neck	venous	neck	coronal
38	74	f	osteoarthritis of the hip (right)	hip pain after trauma	unenhanced	pelvis	coronal
39	37	m	urolithiasis with hydronephrosis II° (left)	flank pain (left)	unenhanced	abdomen	coronal
40	61	m	patella fracture with bloody joint effusion	knee pain after trauma	unenhanced	knee	sagittal
41	55	m	enterocolitis in graft-versus-host disease	abdominal pain after stem cell transplantation	venous	abdomen	axial
42	70	m	Leriche syndrome	acute paraparesis with absent pulse	arterial	abdomen	coronal
43	59	m	basilar aneurysm	sudden collapse and coma	arterial	brain	axial
44	48	m	cerebellar cavernoma	recurring depression and suspected cerebellar mass	unenhanced	brain	axial
45	53	f	lobar pneumonia	dyspnea and signs of systemic infection	venous	lung	axial
46	60	m	viral pneumonia (COVID-19)	progredient dyspnea	unenhanced	lung	axial
47	68	m	dislocation of pedicular screw	pain following back stabilization surgery with internal fixation	unenhanced	spine	axial
48	39	f	thrombosis of the sigmoid sinus (right)	headache (right side) and emesis	venous	brain	coronal
49	83	m	arterial bleeding of the hepatic common artery into an abscess after left hemi-hepatectomy	status post left hemi-hepatectomy due to cholangiocellular carcinoma, now acute drop in hemoglobin levels	arterial	abdomen	coronal
50	80	m	kidney cyst rupture after trauma	left-sided abdominal pain after fall	venous	abdomen	sagittal
51	61	f	gastrointestinal ischemia	abdominal pain with peritonism	arterial	abdomen	axial
52	53	m	appendicitis	acute abdomen with abdominal focus and infection	venous	abdomen	axial
53	73	f	left atrial myxoma	sonographic mass in left atrium	arterial	chest	axial
54	68	m	aspiration of positive oral contrast medium and pneumonia	respiratory distress	venous	lung	axial

55	35	f	lung cancer of the right upper lobe	suspected lung cancer	venous	lung	axial
56	74	f	aneurysm of ascending aorta	suspected lung cancer	venous	chest	axial
57	71	f	pseudoaneurysm with cardiac tamponade	sudden-onset dyspnea and heart failure after coronary catheter angiography	arterial	whole body	coronal
58	40	f	ovarian torsion with incarceration	pelvic pain and intrapelvic mass	venous	abdomen	axial
59	57	f	cutaneous metastasis of a non-small cell lung cancer	progredient skin tumor and history of non-small cell lung cancer	venous	neck	axial
60	39	f	thrombosis of the right sigmoid sinus	headache (right) and emesis	unenhanced	brain	axial

Supplementary Table 3: Magnetic Resonance Imaging - Demographic and Clinical Context Information and Reference Diagnoses.

The patient's age and sex, reference diagnosis, chief complaint, examined anatomic region, sequence weighting, fat saturation, contrast enhancement (phase), and slice orientation are given. Abbreviations: TSE – turbo spin-echo, GRE – gradient-echo, PD – proton density, MRCP - magnetic resonance cholangiopancreatography, IR – inversion recovery, FLAIR - Fluid-Attenuated Inversion Recovery.

No.	Age	Sex	Reference Diagnosis	Chief Complaint	Anatomic Region	Sequence Weighting	Fat Saturation	Contrast) Phase	Slice Orientation
1	18	m	iron overload in sickle cell anemia	asymptomatic (sickle cell anemia)	abdomen	T2 TSE	no	unenhanced	axial
2	38	f	focal nodular hyperplasia of the liver	unclear liver mass in ultrasound	abdomen	T1 GRE	yes	hepatobiliary	axial
3	24	f	Hoffa-fat pad-impingement	knee pain without trauma	knee	PD TSE	yes	unenhanced	sagittal
4	65	m	primary sclerosing cholangitis (liver)	increased markers of cholestasis	abdomen	3D MRCP	yes	unenhanced	coronal
5	58	m	vestibular schwannoma	gradual hearing loss (left)	head	T1 TSE	yes	late venous	coronal
6	50	m	rupture of the quadriceps tendon	knee pain and lack of leg extension	knee	PD TSE	yes	unenhanced	sagittal
7	15	f	anterior displacement of the disc (jaw)	locked jaw	jaw	T1 TSE	no	unenhanced	sagittal
8	27	m	posterior cruciate ligament rupture	knee pain after trauma	knee	PD TSE	yes	unenhanced	sagittal
9	18	m	normal	acute pain (lumbar spine) after fall	lumbar spine	T2 TSE	no	unenhanced	sagittal
10	29	f	normal	systemic mastocytosis	abdomen	T2 TSE	no	unenhanced	axial
11	12	f	flake fracture	knee pain after trauma	knee	PD TSE	yes	unenhanced	axial
12	21	m	pericarditis	fatigue	heart	IR	yes	late venous	4-chamber view
13	17	m	infarction of inferior myocardium (right coronary artery)	acute chest pain	heart	IR	yes	late venous	short axis view

14	66	f	ductal carcinoma in situ	mass in the right breast	breast	T1 GRE (subtraction)	no	early arterial	axial
15	53	f	normal	none	breast	T2 TSE	no	unenhanced	axial
16	26	m	normal	locked jaw	jaw	PD TSE	no	unenhanced	sagittal
17	12	f	normal	plantar pain	foot	PD TSE	yes	unenhanced	sagittal
18	45	f	normal	reduced visual acuity	brain	T2 TSE	no	unenhanced	axial
19	36	m	normal	vertigo	brain	FLAIR	yes	unenhanced	axial
20	64	f	normal	asymptomatic	head	T2 TSE	no	unenhanced	coronal
21	58	f	normal	pulsating tinnitus (left)	head	3D time-of-flight angiography	yes	unenhanced	coronal
22	58	f	normal	pulsating tinnitus (left)	head	3D T2 TSE	no	unenhanced	axial
23	42	f	normal	knee pain after trauma	knee	PD TSE	yes	unenhanced	sagittal
24	75	f	trochanteric bursitis (left)	chronic hip pain (left)	pelvis	PD TSE	yes	unenhanced	axial
25	50	f	kidney cyst (Bosniak I)	sonographic mass of the kidney	abdomen	T2 TSE	no	unenhanced	coronal
26	61	f	medial disc protrusion	lower back pain	lumbar spine	T2 TSE	no	unenhanced	axial
27	24	m	hepatomegaly with kissing spleen phenomenon	alpha-1-antitrypsin deficiency	abdomen	T2 TSE	no	unenhanced	axial
28	73	f	cervix carcinoma	mass of the cervix	pelvis	T2 TSE	no	unenhanced	sagittal
29	52	f	uterus myomatosus	pelvic pain associated with menstrual period	pelvis	T2 TSE	yes	unenhanced	axial
30	54	m	primary sclerosing cholangitis and intrahepatic cholestasis	aggravation of inflammatory bowel disease	abdomen	T2 TSE	no	unenhanced	axial
31	74	m	parotid carcinoma	parotid mass	neck	T2 TSE	yes	unenhanced	axial
32	31	f	osteochondroma of the distal femur	chronic popliteal pain and mass	knee	T1 TSE	no	unenhanced	axial
33	58	m	lipoatrophic hamstring	muscular pain, elevated markers of muscle damage (creatine phosphokinase)	lower extremities	T1 TSE	no	unenhanced	axial
34	26	f	enchondroma of the femur (right)	follicular thyroid carcinoma	neck	T1 TSE	yes	late venous	coronal

35	26	f	hemangioma of L3	pain in the lower back	lumbar spine	T1 TSE	no	unenhanced	sagittal
36	67	f	invasive lobular breast cancer	mass in left breast	breast	T1 GRE (subtraction)	no	early arterial	axial
37	59	f	MRI after microwave ablation (liver)	hepatic metastasis of sigmoid carcinoma	abdomen	T2 TSE	no	unenhanced	axial
38	28	m	secondary synovial chondromatosis	restricted motion of the left hip and massive pain	pelvis	T2 TSE	no	unenhanced	axial
39	71	f	odontogenic cyst of the maxilla (right)	cervical swelling	neck	T1 TSE	yes	late venous	coronal
40	57	f	fibroadenoma of the breast	mass in left breast	breast	T2 TSE	no	unenhanced	axial
41	90	f	fracture of L2	pain in the lower back after fall	lumbar spine	T2 TSE	yes	unenhanced	sagittal
42	74	m	bladder diverticulosis	asymptomatic	pelvis	T2 TSE	no	unenhanced	axial
43	25	m	osteoid osteoma	chronic pain in right proximal femur	thigh	T1 TSE	yes	late venous	axial
44	70	f	liver cirrhosis and splenomegaly	elevated liver enzymes	abdomen	T2 TSE	no	unenhanced	axial
45	77	m	side-duct intraductal papillary mucinous neoplasm	asymptomatic	abdomen	T2 TSE	no	unenhanced	axial
46	71	m	prostate cancer (PI-RADS 5, peripheral zone)	massive elevation of prostate-specific antigen	prostate	T2 TSE	no	unenhanced	axial
47	32	f	teratoma	sonographic ovarian mass right	pelvis	T2 TSE	yes	unenhanced	axial
48	30	f	endometriosis with chocolate cysts	chronic lower abdominal/pelvic pain	pelvis	T2 TSE	no	unenhanced	coronal
49	17	f	uterus didelphys	chronic pelvic pain	pelvis	T2 TSE	yes	unenhanced	axial
50	64	m	exophytic urothelial carcinoma (bladder wall)	elevation of prostate-specific antigen	prostate	T2 TSE	no	unenhanced	axial
51	26	m	seminal vesiculitis (left) with prostatitis	persistent lower abdominal pain after urinary tract infection	pelvis	T2 TSE	no	unenhanced	coronal
52	29	m	peripheral nerve sheath tumor	popliteal mass	knee	T2 TSE	yes	unenhanced	sag
53	50	m	Crohn's disease with sigmoid affection	chronic abdominal pain and inflammation	abdomen	T1 GRE	yes	late venous	coronal

54	20	m	coagulation in corpora cavernosa after penile rupture	priapism after sexual trauma	penis	T1 TSE	no	unenhanced	axial
55	43	m	plantar fibromatosis	plantar pain	foot	T1 TSE	yes	late venous	sagittal
56	83	f	empty sella	central hypothyroidism	brain	T1 TSE	yes	late venous	coronal
57	68	m	sialolithiasis of submandibular duct	tumor of the left buccal wall	head	T2 TSE	no	unenhanced	axial
58	75	f	choledocholithiasis	cholangiopathy with recurring infections	abdome n	T2 TSE	no	unenhanced	coronal
59	60	m	hemangioma of the thumb	tumor of the thumb	thumb	4D angiography (subtraction)	no	arterial	sagittal
60	71	m	massive benign prostate hyperplasia	nycturia	pelvis	T2 TSE	no	unenhanced	axial

Supplementary Table 4: Angiography - Demographic and Clinical Context Information and Reference Diagnoses.

The patient's age and sex, reference diagnosis, and chief complaint are given.

No.	Age	Sex	Reference Diagnosis	Chief Complaint
1	74	m	tumor blush of hepatocellular carcinoma	cirrhosis and liver mass of unknown origin
2	74	m	pseudoaneurysm of common hepatic artery	upper abdominal pain and drop in hemoglobin levels
3	69	m	bile leakage due to percutaneous transhepatic biliary drainage insufficiency	persisting infection after percutaneous transhepatic biliary drainage
4	54	m	advanced stenosis of proximal superior femoral artery	leg pain during ambulation
5	86	m	thrombosis of the port catheter tip	dysfunctional port catheter
6	75	f	active bleeding due to shunt vein rupture	acute pain and swelling in the left arm (shunt vein in left arm)
7	30	m	femoral venous thrombosis with postthrombotic changes	swelling and pain in the leg
8	48	m	thrombotic occlusion of transjugular intrahepatic shunt	increasing ascites (transjugular intrahepatic portosystemic shunt)
9	83	m	normal percutaneous transhepatic biliary drainage without signs of leakage	persisting infection after percutaneous transhepatic biliary drainage
10	74	m	normal mesentericography	liver tumor, presence of aberrant tumor-feeding arteries

			with indirect portography	
11	34	f	implantable port without signs of dysfunction	control after implantation of port-a-cath
12	77	f	active bleeding of deep femoral artery	sudden pain and swelling of the left groin after extraction of arterial catheter
13	67	f	aneurysm of the splenic artery	pain in the upper left abdomen radiating to the shoulder and palpable pulsation in left upper abdomen
14	58	m	stenosis of the superior mesenteric artery	intermittent abdominal pain after eating
15	63	m	thrombosis of the superior mesenteric artery	severe abdominal pain, nausea, and vomiting; history of untreated atrial fibrillation
16	41	f	primary sclerosing cholangitis	recurrent cholestasis, itching, and upper abdominal pain years after liver transplantation
17	69	m	subtotal occlusion of the inferior trunk of the middle cerebral artery	sudden onset left-sided hemiparesis and aphasia
18	79	m	stenosis of dialysis shunt	dialysis shunt dysfunction
19	66	m	occlusion of left renal artery	left flank pain and bloody urine
20	73	m	dislocation of port catheter	dysfunction of port catheter
21	76	f	superior vena cava syndrome	slowly progressive headache and facial swelling; history of extensive non-small cell lung cancer
22	27	f	arteriovenous malformation in gluteal muscles	persistent gluteal pain
23	67	m	Budd Chiari syndrome	right upper quadrant pain, ascites
24	29	m	normal angiogram of the leg	leg pain after trauma
25	90	m	endoleak type II	asymptomatic after endovascular aortic repair

26	63	m	normal T-drain	asymptomatic; recent liver transplantation

Supplementary Text S1

Power Analysis

This study's power analysis was informed by previous studies evaluating the effects of AI assistance on diagnostic performance [30; 31]. Because of differences in study design, methodologic procedures, and sample characteristics, we assumed a moderate effect size of 0.5. Consequently, the minimum sample size was determined as 26 based on a statistical power of 0.8, a probability of type I α error of 0.05, and a two-tailed procedure (G*Power, v3.1; Heinrich-Heine-University) [32]. To realize sufficient statistical power per modality, we included 60 radiographs, 60 CT images, 60 MR images, and 26 angiographic images, i.e., 206 images. Fewer angiographic images were included due to our hospital's substantially smaller volume of angiographic imaging.
