# Supplementary figures and images for: Revolution or risk?—Assessing the potential and challenges of GPT-4V in radiologic image interpretation (part 2 of 2)
Source: Eur Radiol. 2024 Oct 18;35(3):1111–21. doi: 10.1007/s00330-024-11115-6 (PMC11836096; doi:10.1007/s00330-024-11115-6)

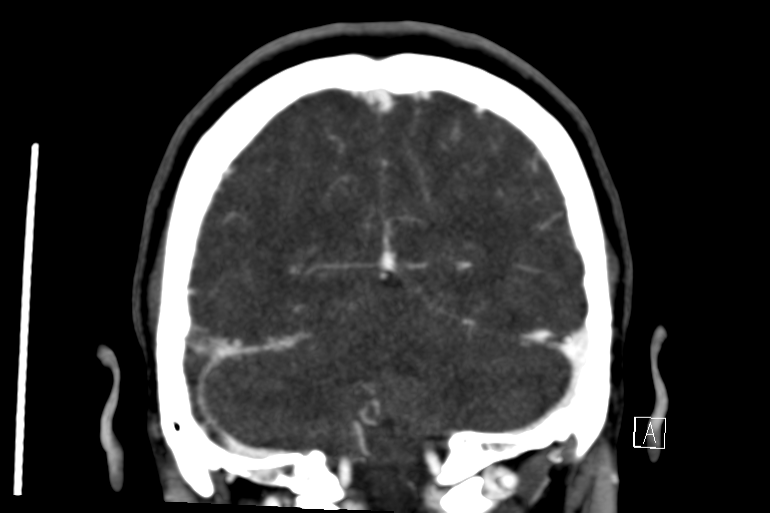

Supplement: Supplementary file 2 — Electronic Supplementary Material [file 330_2024_11115_MOESM2_ESM.zip › Digital Supplementary Material/Computed Tomography/48Computed Tomography.PNG]

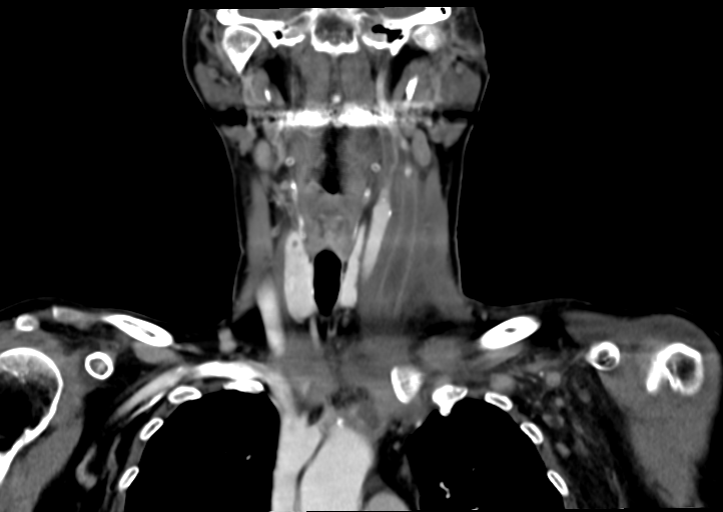

Supplement: Supplementary file 2 — Electronic Supplementary Material [file 330_2024_11115_MOESM2_ESM.zip › Digital Supplementary Material/Computed Tomography/37Computed Tomography.PNG]

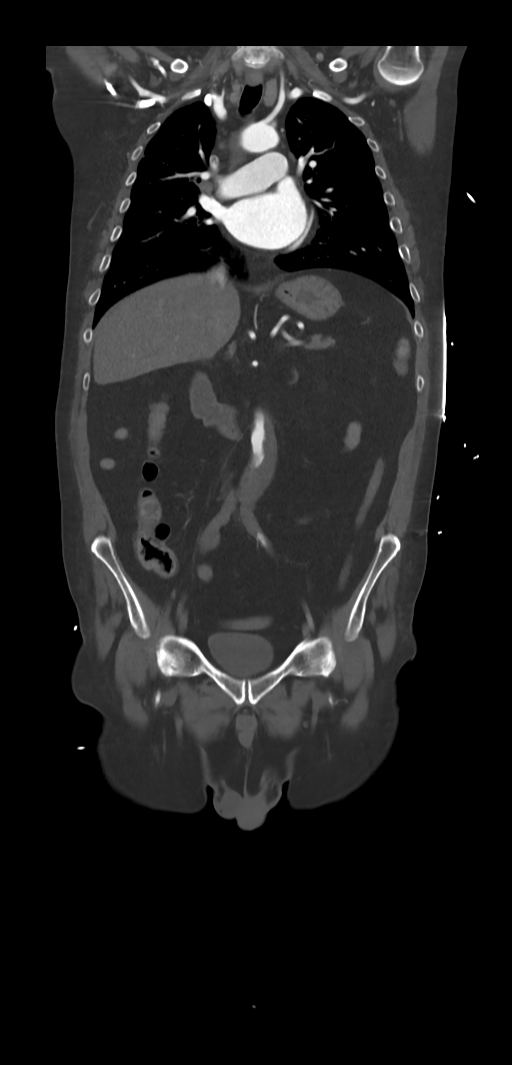

Supplement: Supplementary file 2 — Electronic Supplementary Material [file 330_2024_11115_MOESM2_ESM.zip › Digital Supplementary Material/Computed Tomography/42Computed Tomography.PNG]

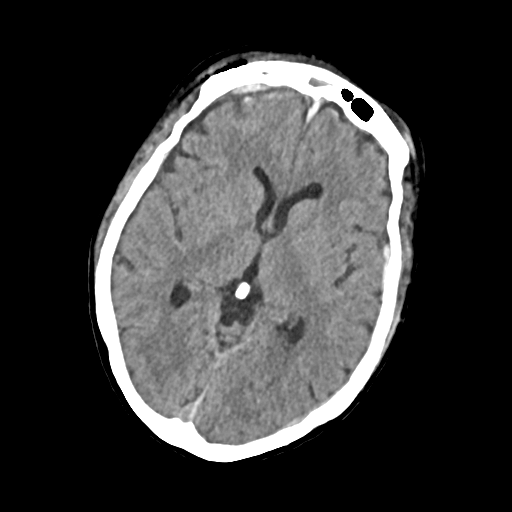

Supplement: Supplementary file 2 — Electronic Supplementary Material [file 330_2024_11115_MOESM2_ESM.zip › Digital Supplementary Material/Computed Tomography/30Computed Tomography.PNG]

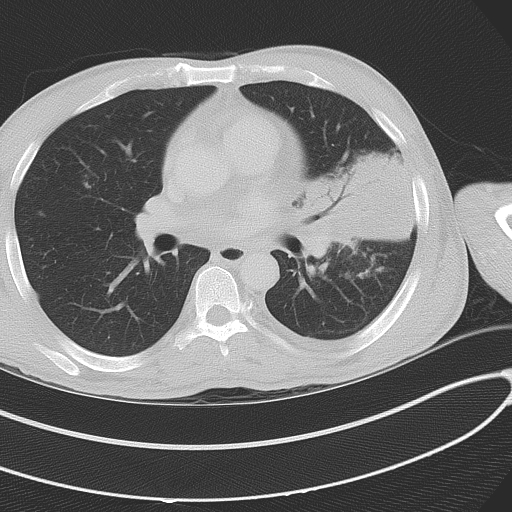

Supplement: Supplementary file 2 — Electronic Supplementary Material [file 330_2024_11115_MOESM2_ESM.zip › Digital Supplementary Material/Computed Tomography/45Computed Tomography.PNG]

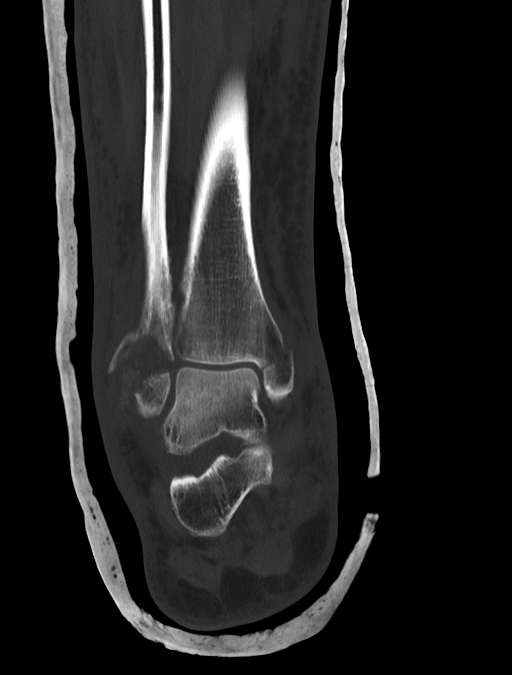

Supplement: Supplementary file 2 — Electronic Supplementary Material [file 330_2024_11115_MOESM2_ESM.zip › Digital Supplementary Material/Computed Tomography/22Computed Tomography.PNG]

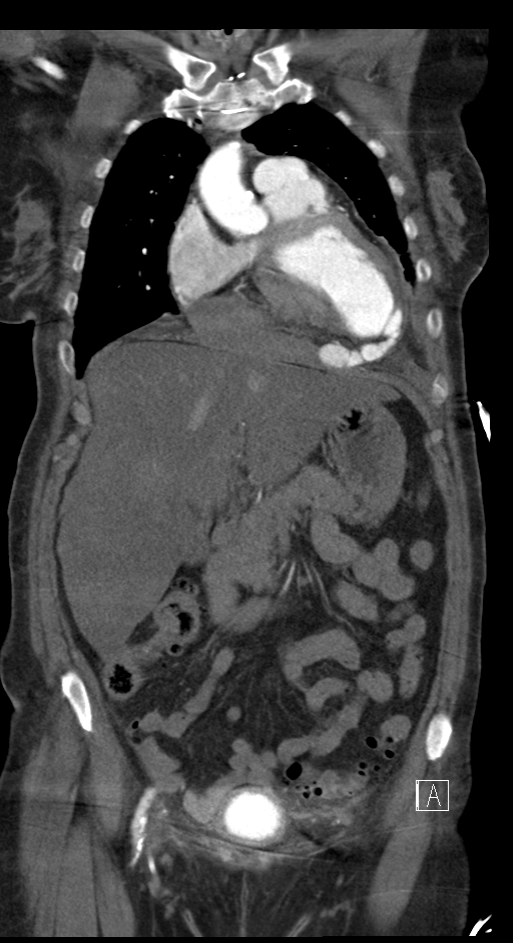

Supplement: Supplementary file 2 — Electronic Supplementary Material [file 330_2024_11115_MOESM2_ESM.zip › Digital Supplementary Material/Computed Tomography/57Computed Tomography.PNG]

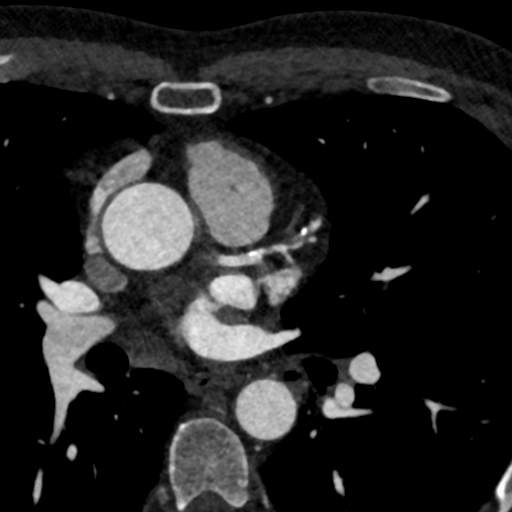

Supplement: Supplementary file 2 — Electronic Supplementary Material [file 330_2024_11115_MOESM2_ESM.zip › Digital Supplementary Material/Computed Tomography/28Computed Tomography.PNG]

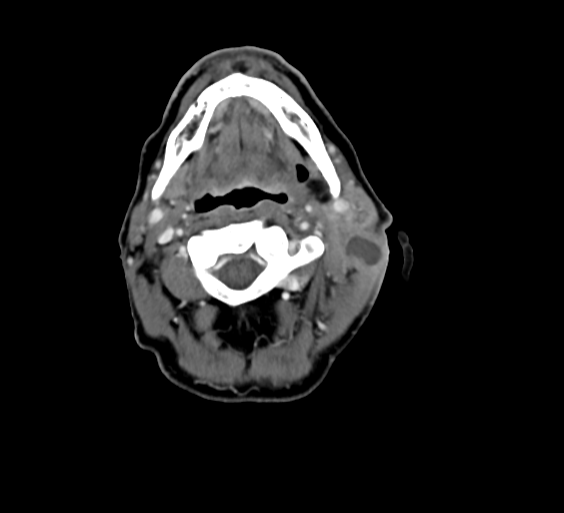

Supplement: Supplementary file 2 — Electronic Supplementary Material [file 330_2024_11115_MOESM2_ESM.zip › Digital Supplementary Material/Computed Tomography/4Computed Tomography.PNG]

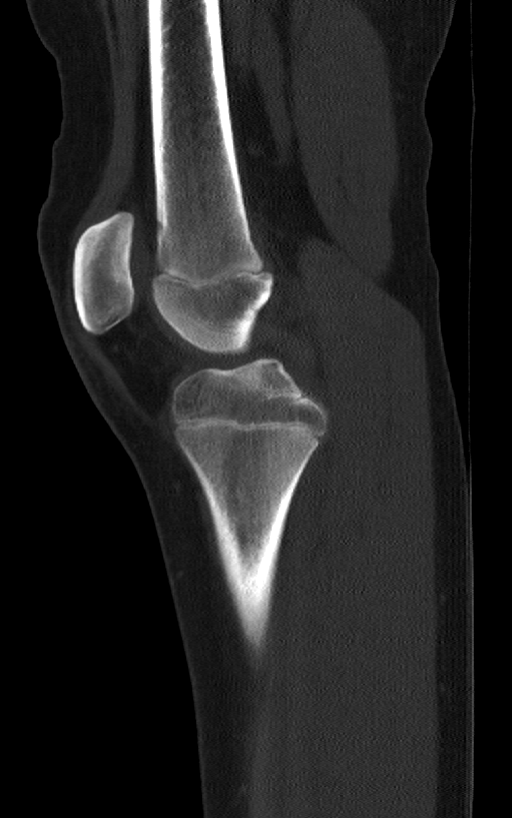

Supplement: Supplementary file 2 — Electronic Supplementary Material [file 330_2024_11115_MOESM2_ESM.zip › Digital Supplementary Material/Computed Tomography/19Computed Tomography.PNG]

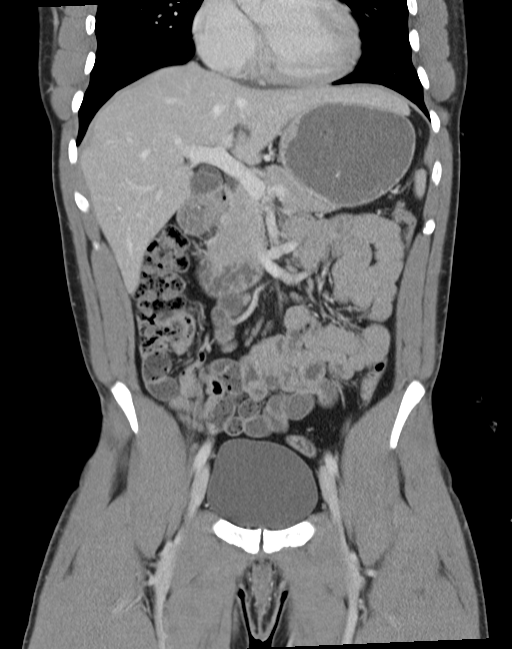

Supplement: Supplementary file 2 — Electronic Supplementary Material [file 330_2024_11115_MOESM2_ESM.zip › Digital Supplementary Material/Computed Tomography/13Computed Tomography.PNG]

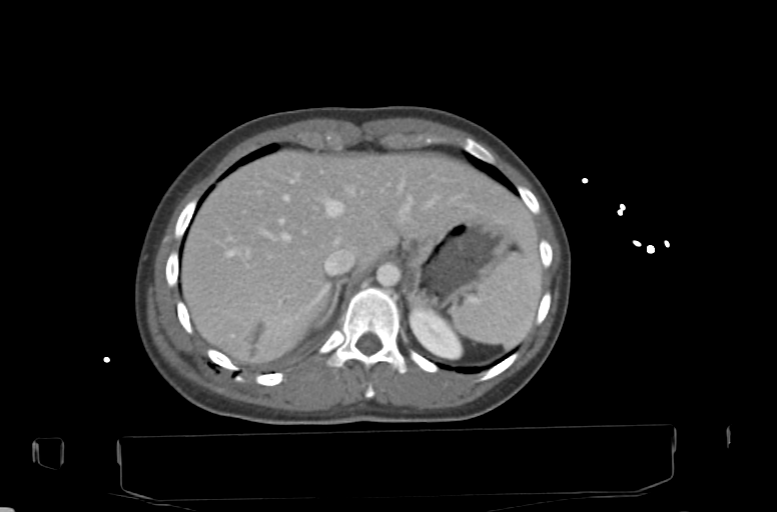

Supplement: Supplementary file 2 — Electronic Supplementary Material [file 330_2024_11115_MOESM2_ESM.zip › Digital Supplementary Material/Computed Tomography/25Computed Tomography.PNG]

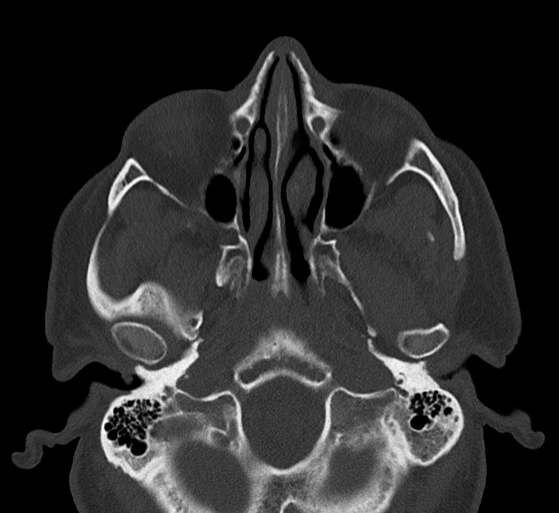

Supplement: Supplementary file 2 — Electronic Supplementary Material [file 330_2024_11115_MOESM2_ESM.zip › Digital Supplementary Material/Computed Tomography/9Computed Tomography.PNG]

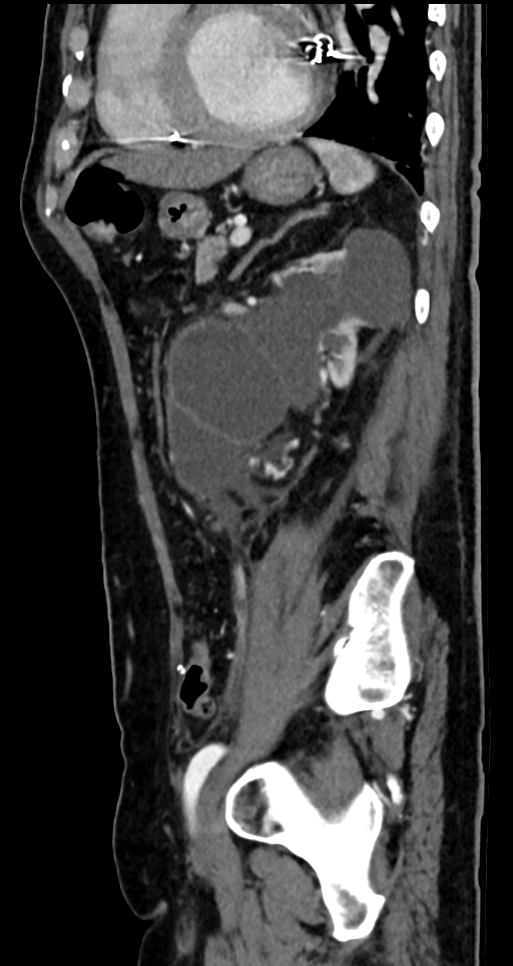

Supplement: Supplementary file 2 — Electronic Supplementary Material [file 330_2024_11115_MOESM2_ESM.zip › Digital Supplementary Material/Computed Tomography/50Computed Tomography.PNG]

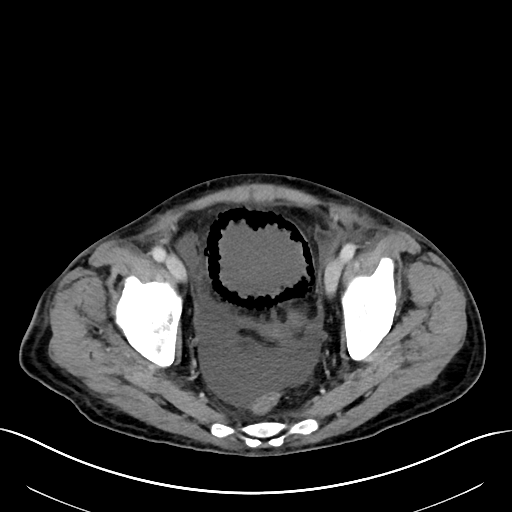

Supplement: Supplementary file 2 — Electronic Supplementary Material [file 330_2024_11115_MOESM2_ESM.zip › Digital Supplementary Material/Computed Tomography/3Computed Tomography.PNG]

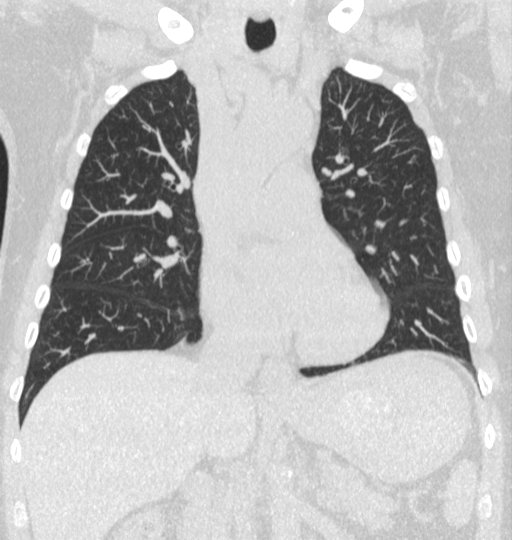

Supplement: Supplementary file 2 — Electronic Supplementary Material [file 330_2024_11115_MOESM2_ESM.zip › Digital Supplementary Material/Computed Tomography/14Computed Tomography.PNG]

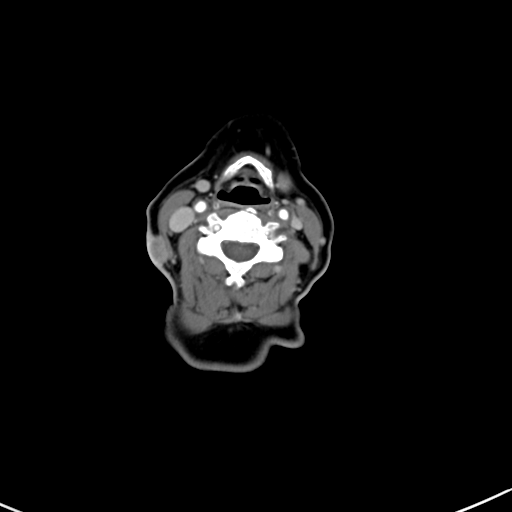

Supplement: Supplementary file 2 — Electronic Supplementary Material [file 330_2024_11115_MOESM2_ESM.zip › Digital Supplementary Material/Computed Tomography/59Computed Tomography.PNG]

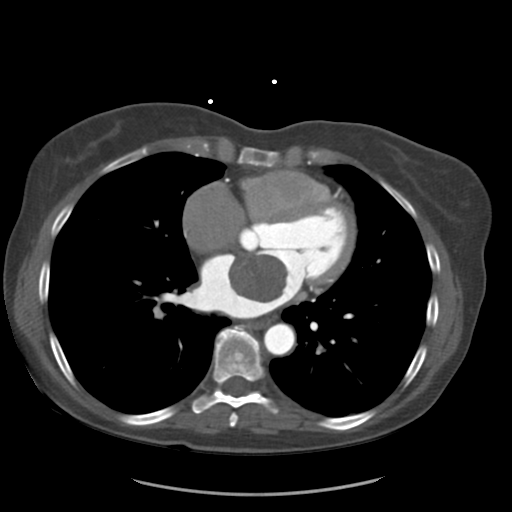

Supplement: Supplementary file 2 — Electronic Supplementary Material [file 330_2024_11115_MOESM2_ESM.zip › Digital Supplementary Material/Computed Tomography/53Computed Tomography.PNG]

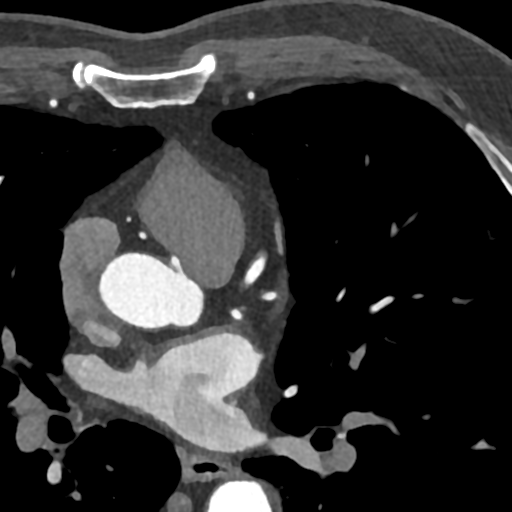

Supplement: Supplementary file 2 — Electronic Supplementary Material [file 330_2024_11115_MOESM2_ESM.zip › Digital Supplementary Material/Computed Tomography/26Computed Tomography.PNG]

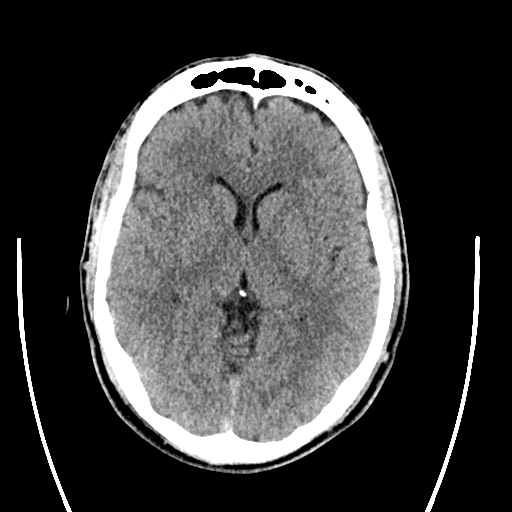

Supplement: Supplementary file 2 — Electronic Supplementary Material [file 330_2024_11115_MOESM2_ESM.zip › Digital Supplementary Material/Computed Tomography/17Computed Tomography.PNG]

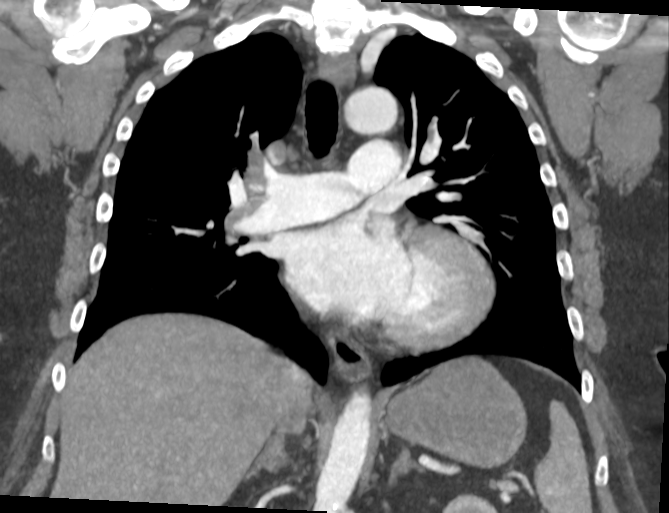

Supplement: Supplementary file 2 — Electronic Supplementary Material [file 330_2024_11115_MOESM2_ESM.zip › Digital Supplementary Material/Computed Tomography/7Computed Tomography.PNG]

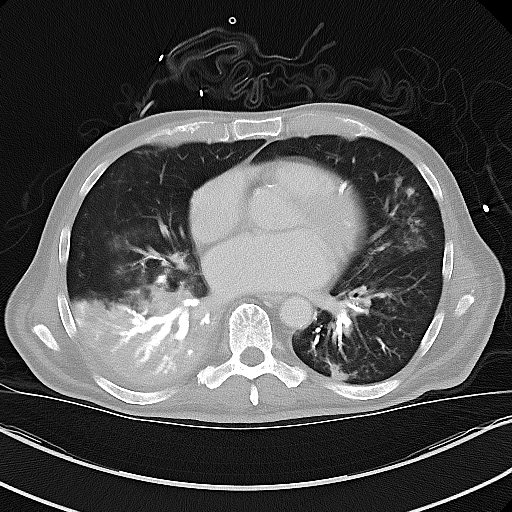

Supplement: Supplementary file 2 — Electronic Supplementary Material [file 330_2024_11115_MOESM2_ESM.zip › Digital Supplementary Material/Computed Tomography/54Computed Tomography.PNG]

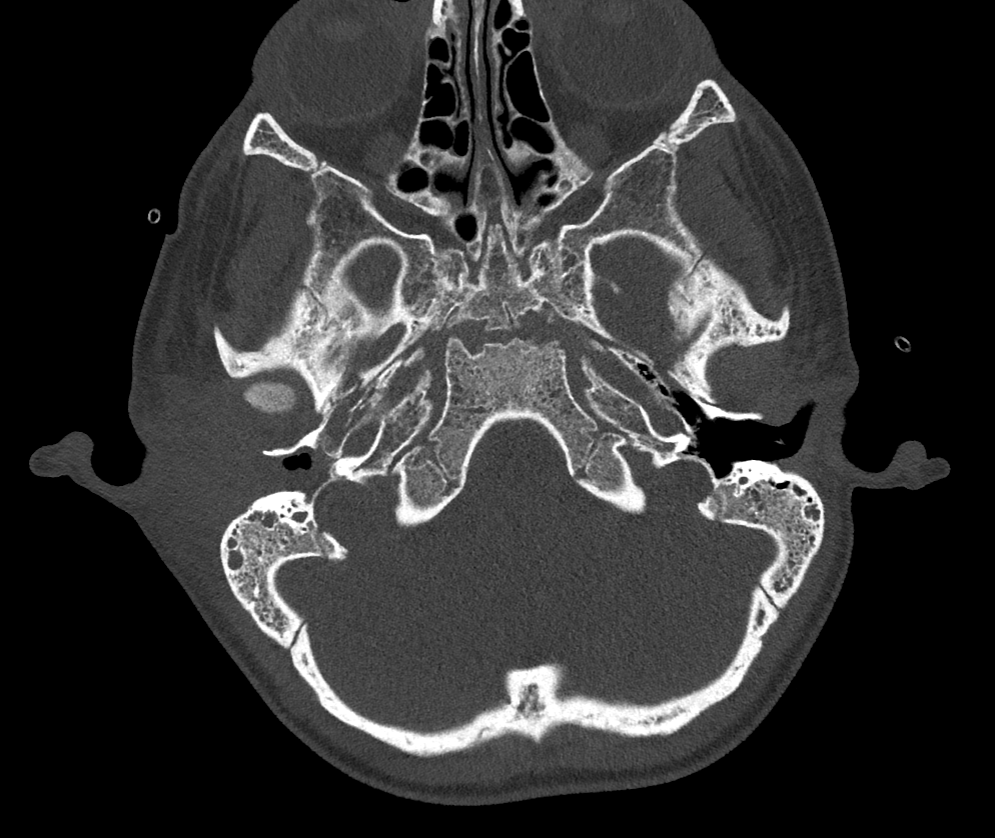

Supplement: Supplementary file 2 — Electronic Supplementary Material [file 330_2024_11115_MOESM2_ESM.zip › Digital Supplementary Material/Computed Tomography/21Computed Tomography.PNG]

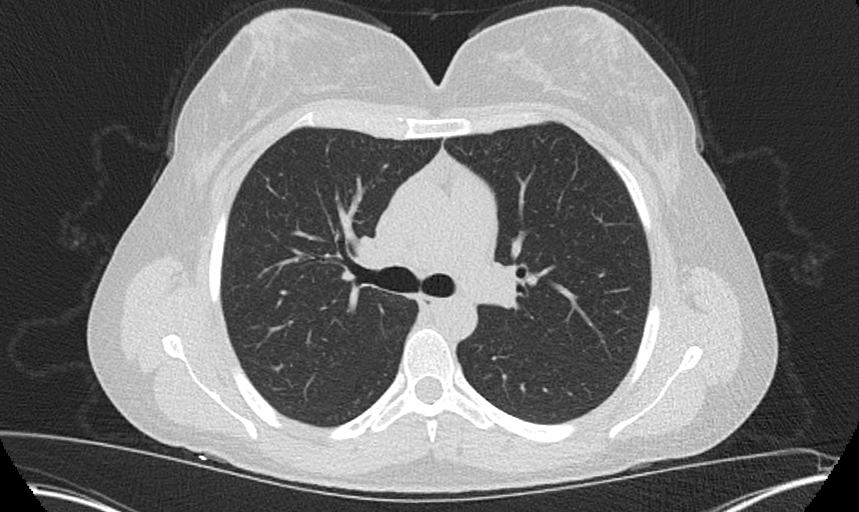

Supplement: Supplementary file 2 — Electronic Supplementary Material [file 330_2024_11115_MOESM2_ESM.zip › Digital Supplementary Material/Computed Tomography/10Computed Tomography.PNG]

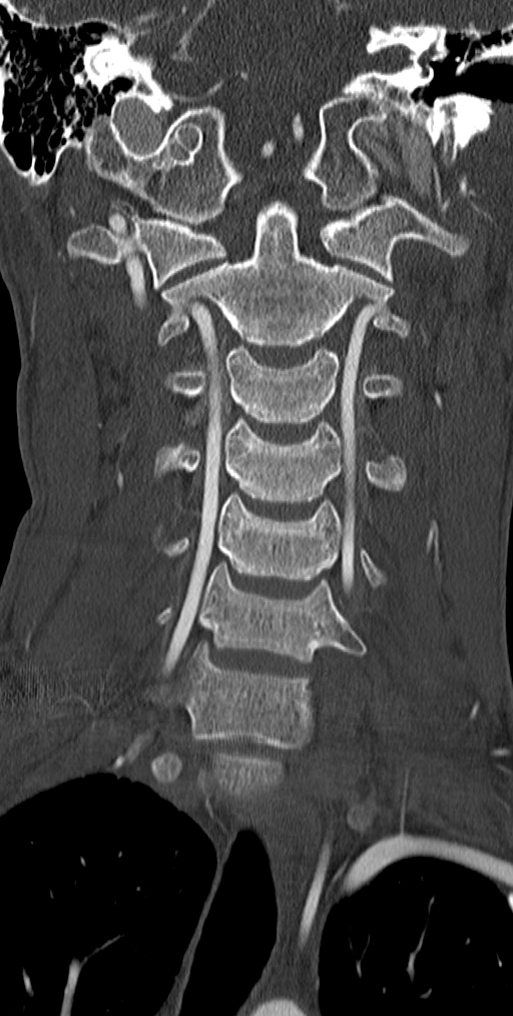

Supplement: Supplementary file 2 — Electronic Supplementary Material [file 330_2024_11115_MOESM2_ESM.zip › Digital Supplementary Material/Computed Tomography/11Computed Tomography.PNG]

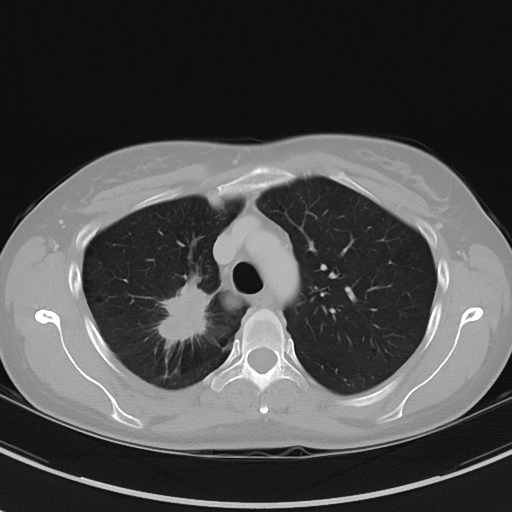

Supplement: Supplementary file 2 — Electronic Supplementary Material [file 330_2024_11115_MOESM2_ESM.zip › Digital Supplementary Material/Computed Tomography/55Computed Tomography.PNG]

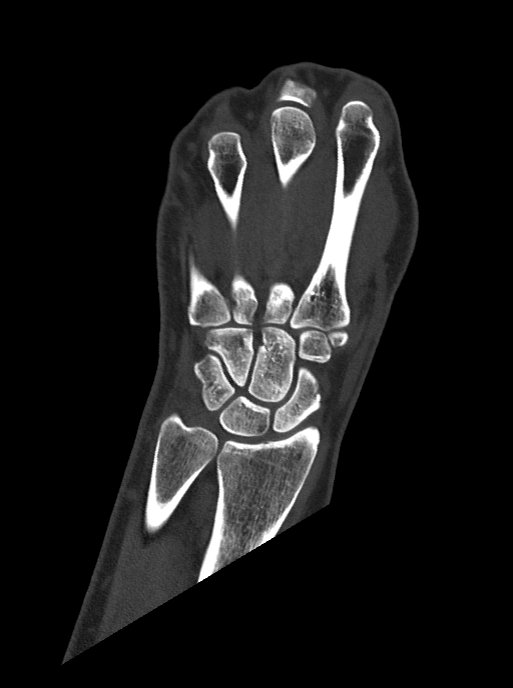

Supplement: Supplementary file 2 — Electronic Supplementary Material [file 330_2024_11115_MOESM2_ESM.zip › Digital Supplementary Material/Computed Tomography/20Computed Tomography.PNG]

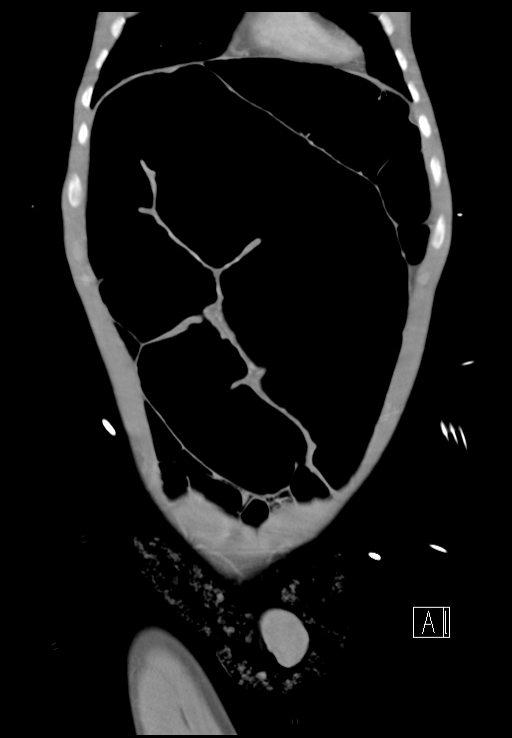

Supplement: Supplementary file 2 — Electronic Supplementary Material [file 330_2024_11115_MOESM2_ESM.zip › Digital Supplementary Material/Computed Tomography/6Computed Tomography.PNG]

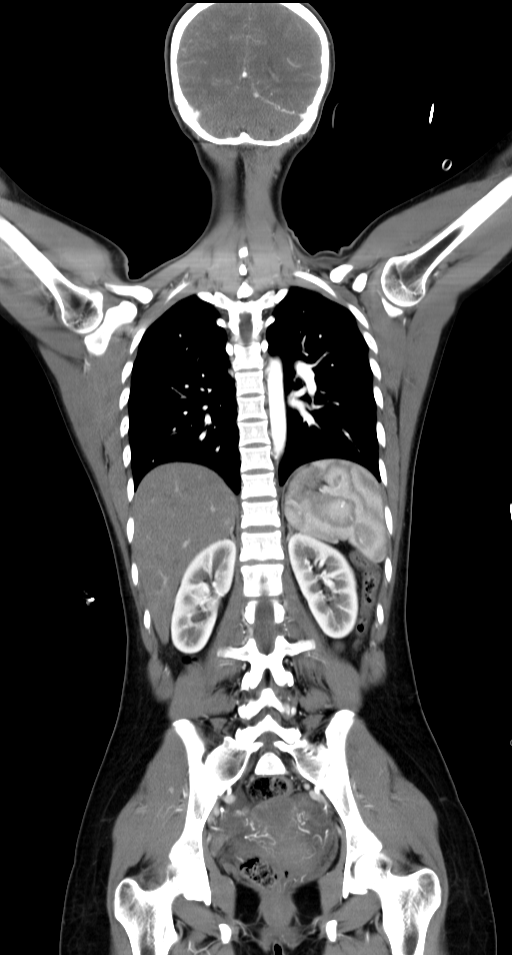

Supplement: Supplementary file 2 — Electronic Supplementary Material [file 330_2024_11115_MOESM2_ESM.zip › Digital Supplementary Material/Computed Tomography/16Computed Tomography.PNG]

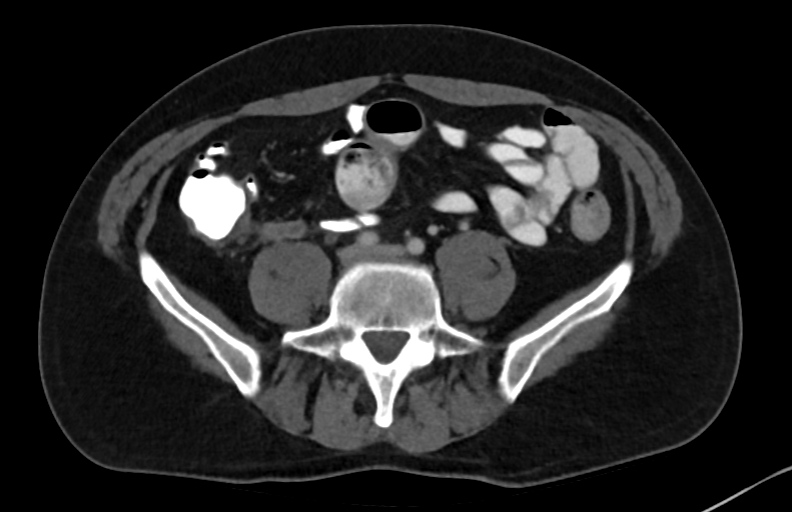

Supplement: Supplementary file 2 — Electronic Supplementary Material [file 330_2024_11115_MOESM2_ESM.zip › Digital Supplementary Material/Computed Tomography/52Computed Tomography.PNG]

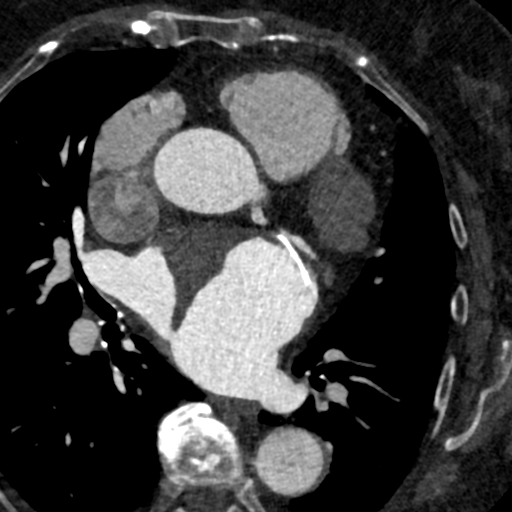

Supplement: Supplementary file 2 — Electronic Supplementary Material [file 330_2024_11115_MOESM2_ESM.zip › Digital Supplementary Material/Computed Tomography/27Computed Tomography.PNG]

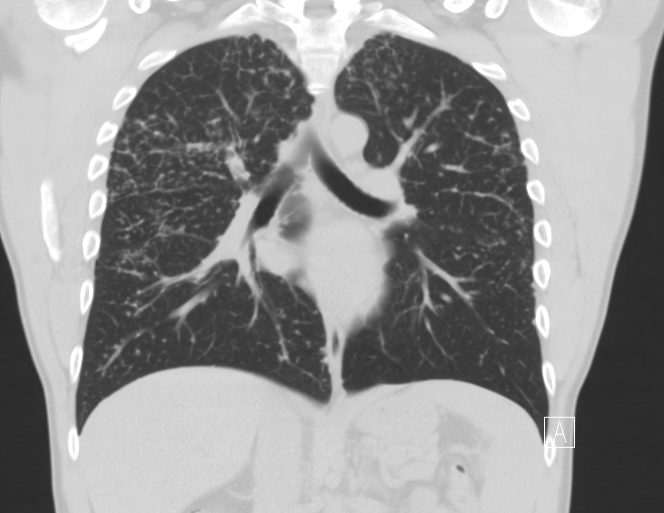

Supplement: Supplementary file 2 — Electronic Supplementary Material [file 330_2024_11115_MOESM2_ESM.zip › Digital Supplementary Material/Computed Tomography/1Computed Tomography.PNG]

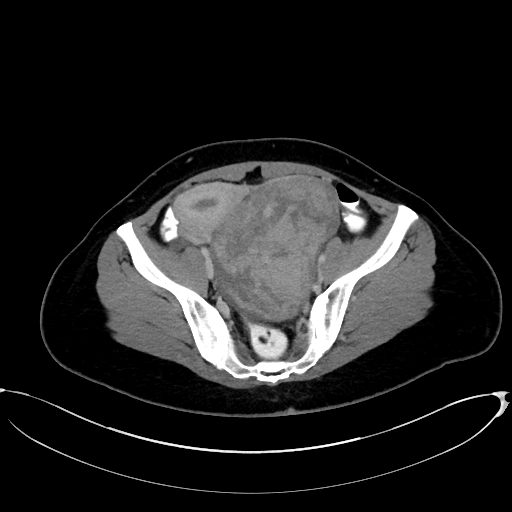

Supplement: Supplementary file 2 — Electronic Supplementary Material [file 330_2024_11115_MOESM2_ESM.zip › Digital Supplementary Material/Computed Tomography/58Computed Tomography.PNG]

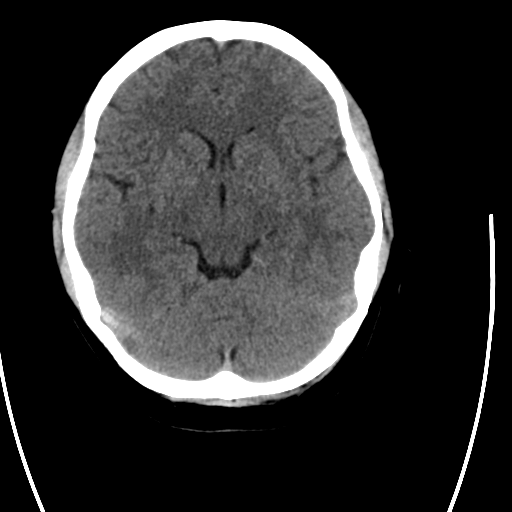

Supplement: Supplementary file 2 — Electronic Supplementary Material [file 330_2024_11115_MOESM2_ESM.zip › Digital Supplementary Material/Computed Tomography/60Computed Tomography.PNG]

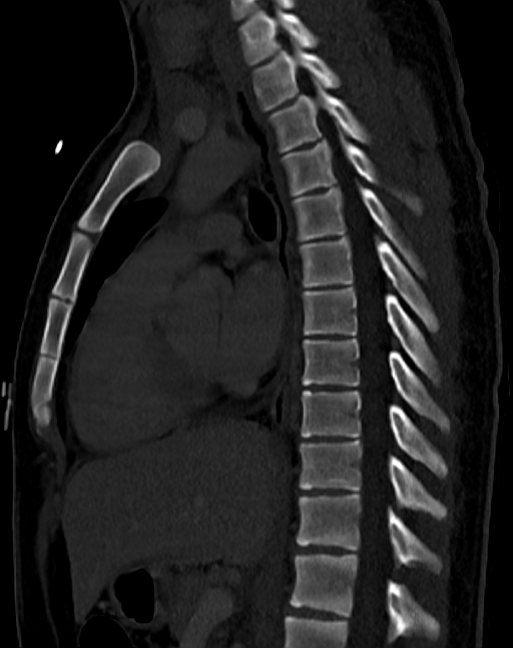

Supplement: Supplementary file 2 — Electronic Supplementary Material [file 330_2024_11115_MOESM2_ESM.zip › Digital Supplementary Material/Computed Tomography/15Computed Tomography.PNG]

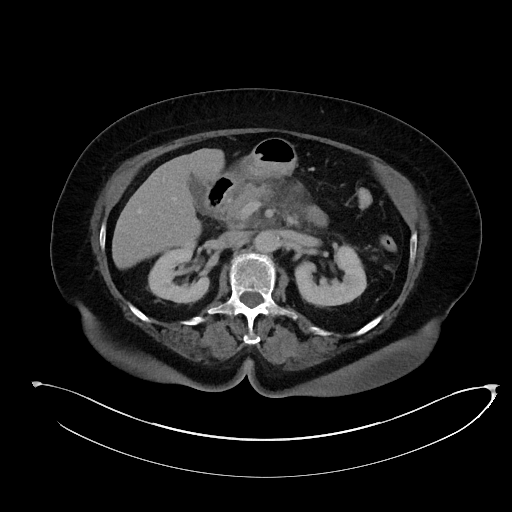

Supplement: Supplementary file 2 — Electronic Supplementary Material [file 330_2024_11115_MOESM2_ESM.zip › Digital Supplementary Material/Computed Tomography/2Computed Tomography.PNG]

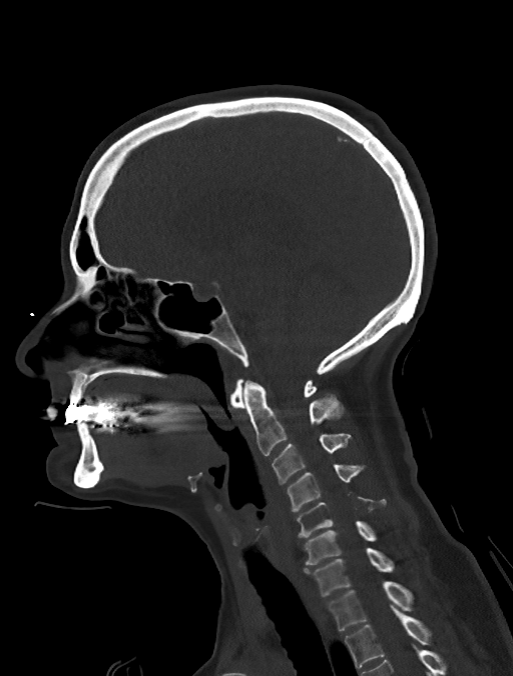

Supplement: Supplementary file 2 — Electronic Supplementary Material [file 330_2024_11115_MOESM2_ESM.zip › Digital Supplementary Material/Computed Tomography/8Computed Tomography.PNG]

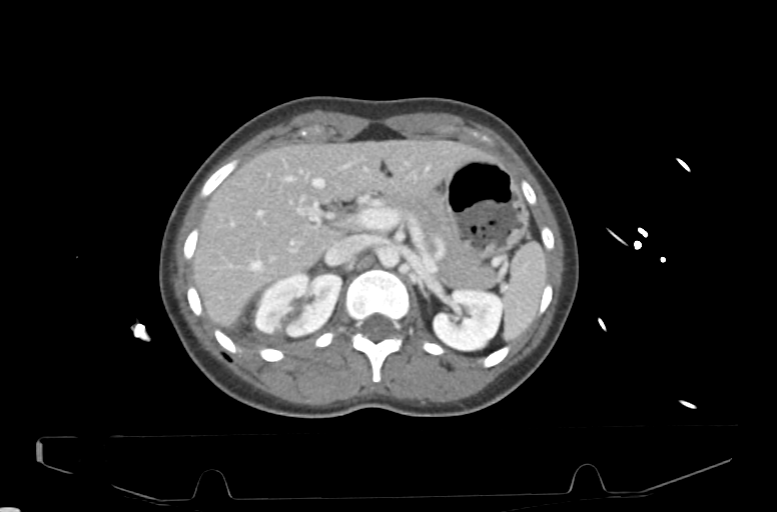

Supplement: Supplementary file 2 — Electronic Supplementary Material [file 330_2024_11115_MOESM2_ESM.zip › Digital Supplementary Material/Computed Tomography/24Computed Tomography.PNG]

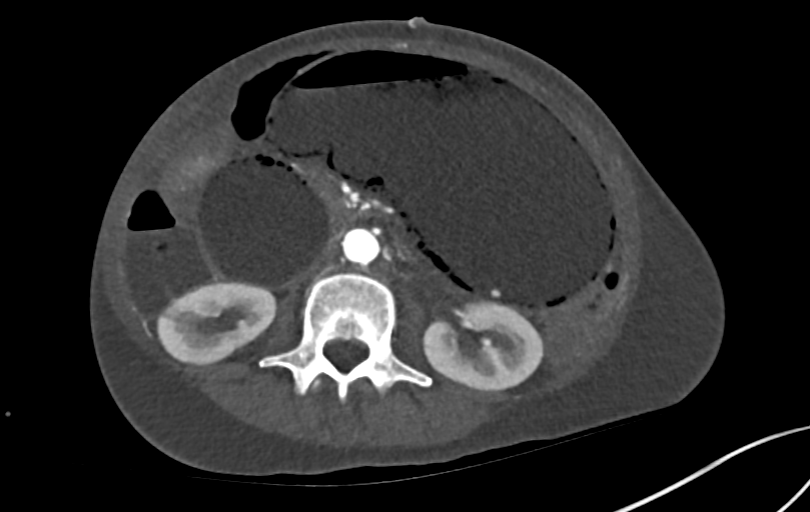

Supplement: Supplementary file 2 — Electronic Supplementary Material [file 330_2024_11115_MOESM2_ESM.zip › Digital Supplementary Material/Computed Tomography/51Computed Tomography.PNG]

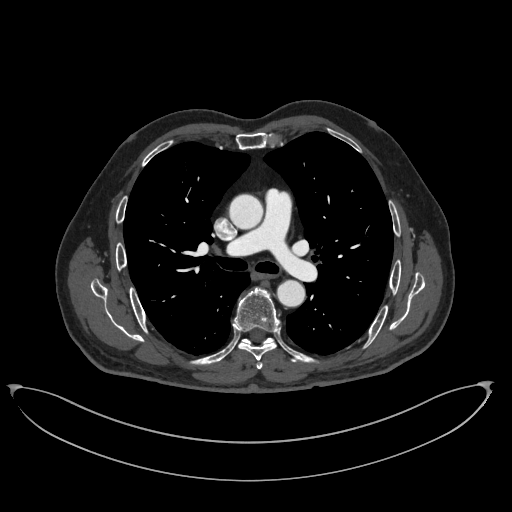

Supplement: Supplementary file 2 — Electronic Supplementary Material [file 330_2024_11115_MOESM2_ESM.zip › Digital Supplementary Material/Computed Tomography/12Computed Tomography.PNG]

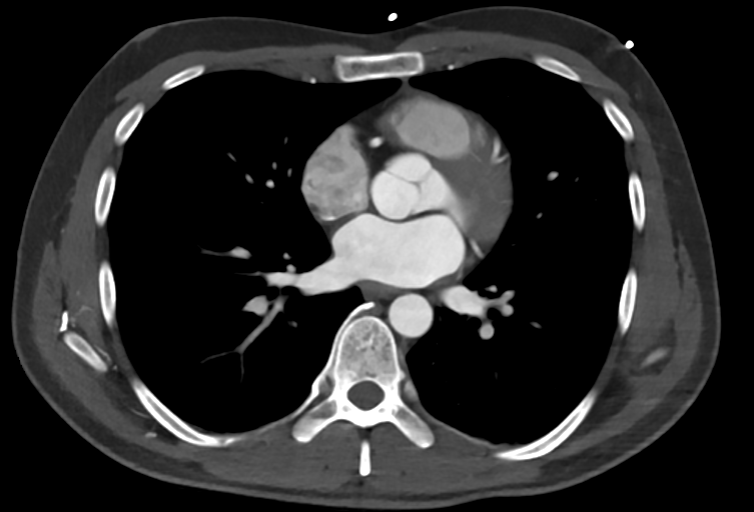

Supplement: Supplementary file 2 — Electronic Supplementary Material [file 330_2024_11115_MOESM2_ESM.zip › Digital Supplementary Material/Computed Tomography/18Computed Tomography.PNG]

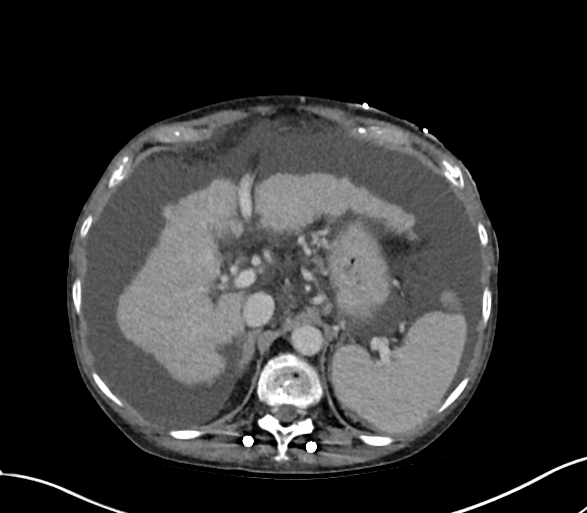

Supplement: Supplementary file 2 — Electronic Supplementary Material [file 330_2024_11115_MOESM2_ESM.zip › Digital Supplementary Material/Computed Tomography/5Computed Tomography.PNG]

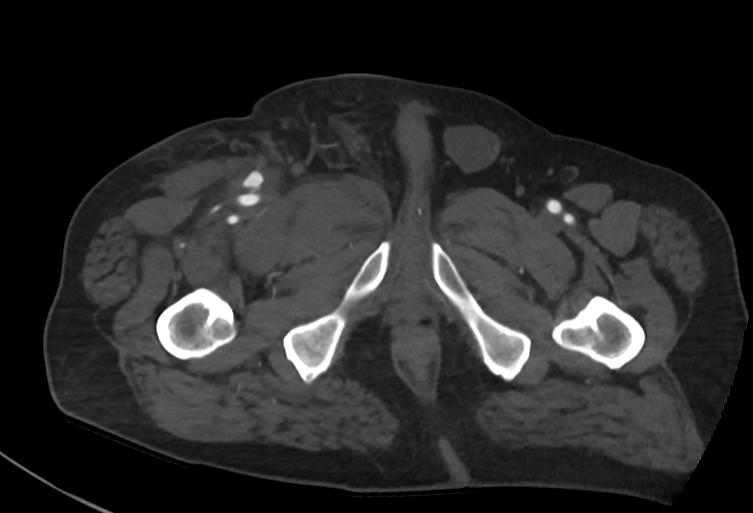

Supplement: Supplementary file 2 — Electronic Supplementary Material [file 330_2024_11115_MOESM2_ESM.zip › Digital Supplementary Material/Computed Tomography/29Computed Tomography.PNG]

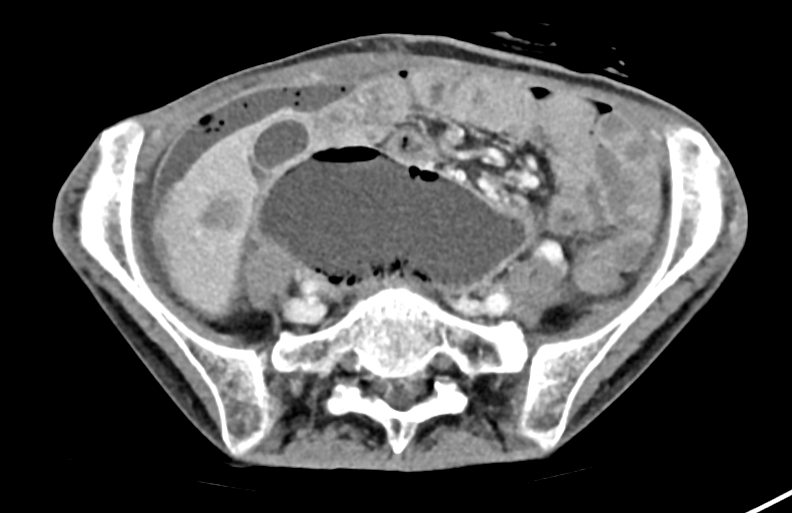

Supplement: Supplementary file 2 — Electronic Supplementary Material [file 330_2024_11115_MOESM2_ESM.zip › Digital Supplementary Material/Computed Tomography/23Computed Tomography.PNG]

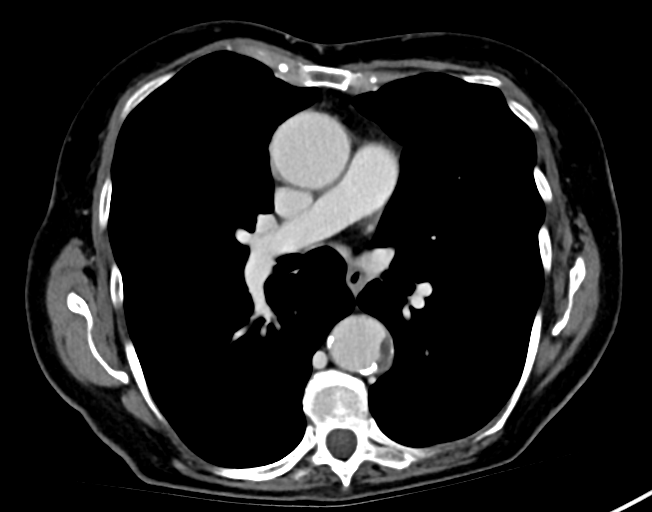

Supplement: Supplementary file 2 — Electronic Supplementary Material [file 330_2024_11115_MOESM2_ESM.zip › Digital Supplementary Material/Computed Tomography/56Computed Tomography.PNG]

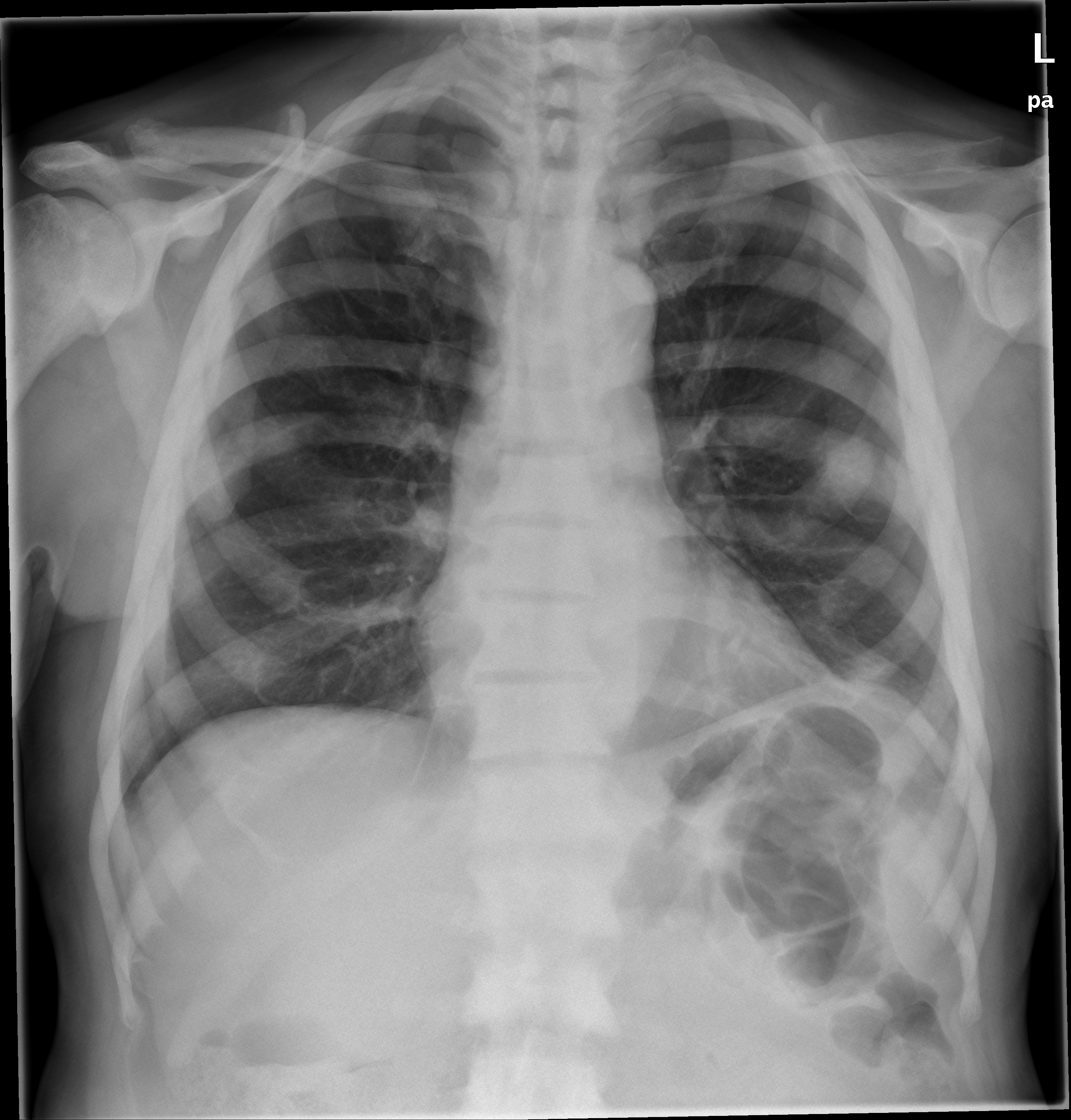

Supplement: Supplementary file 2 — Electronic Supplementary Material [file 330_2024_11115_MOESM2_ESM.zip › Digital Supplementary Material/Radiography/18Radiography.PNG]

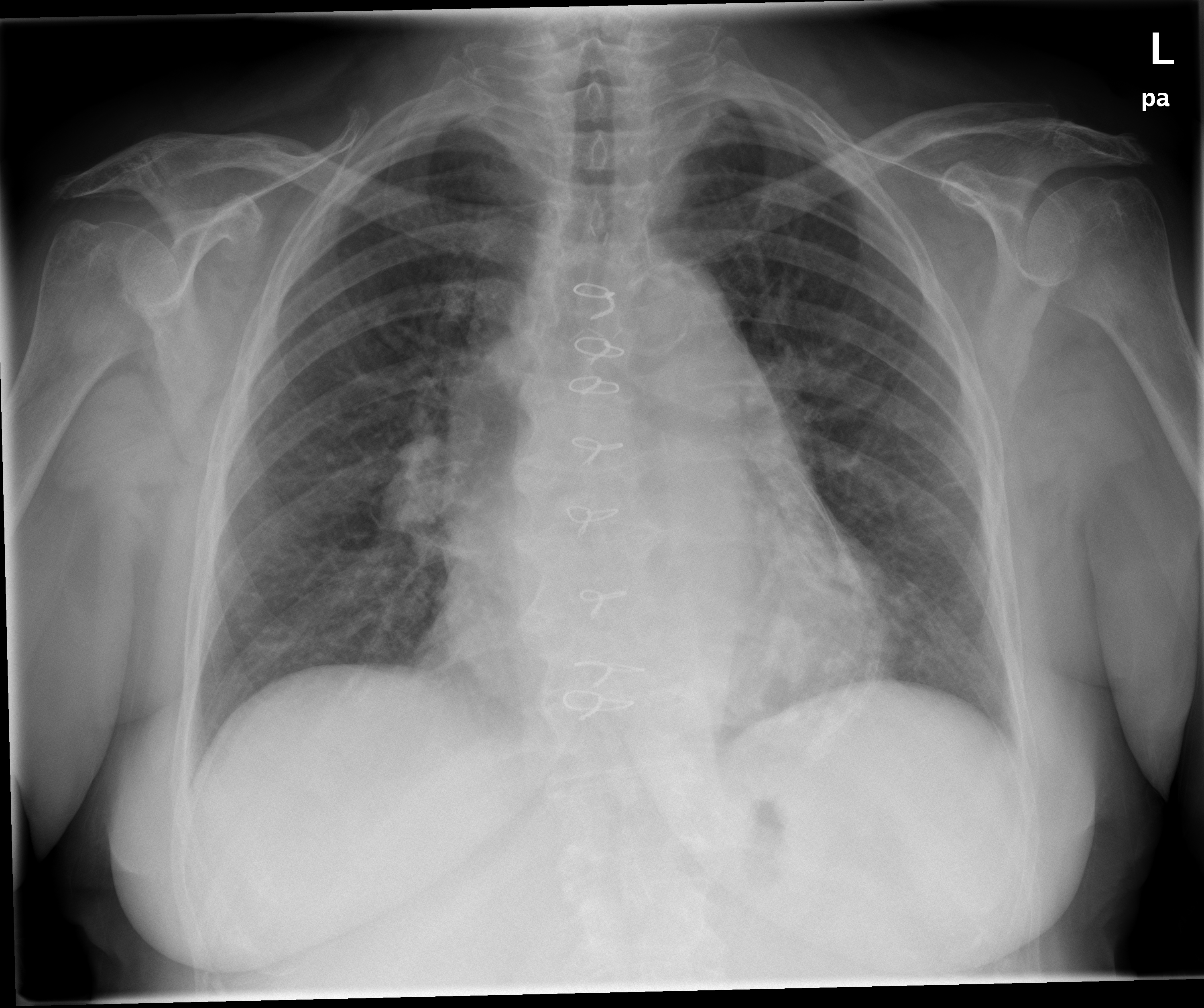

Supplement: Supplementary file 2 — Electronic Supplementary Material [file 330_2024_11115_MOESM2_ESM.zip › Digital Supplementary Material/Radiography/10Radiography.PNG]

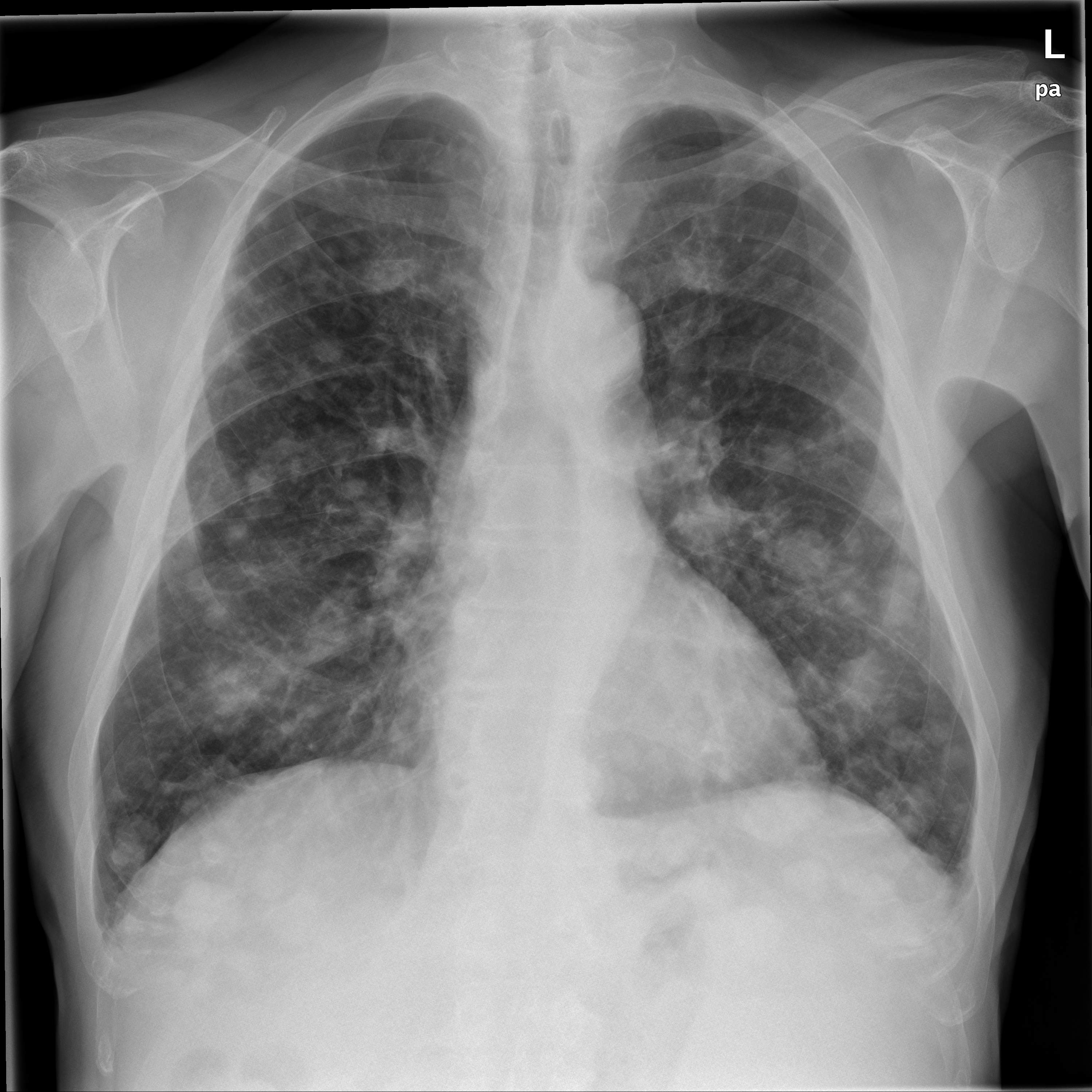

Supplement: Supplementary file 2 — Electronic Supplementary Material [file 330_2024_11115_MOESM2_ESM.zip › Digital Supplementary Material/Radiography/17Radiography.PNG]

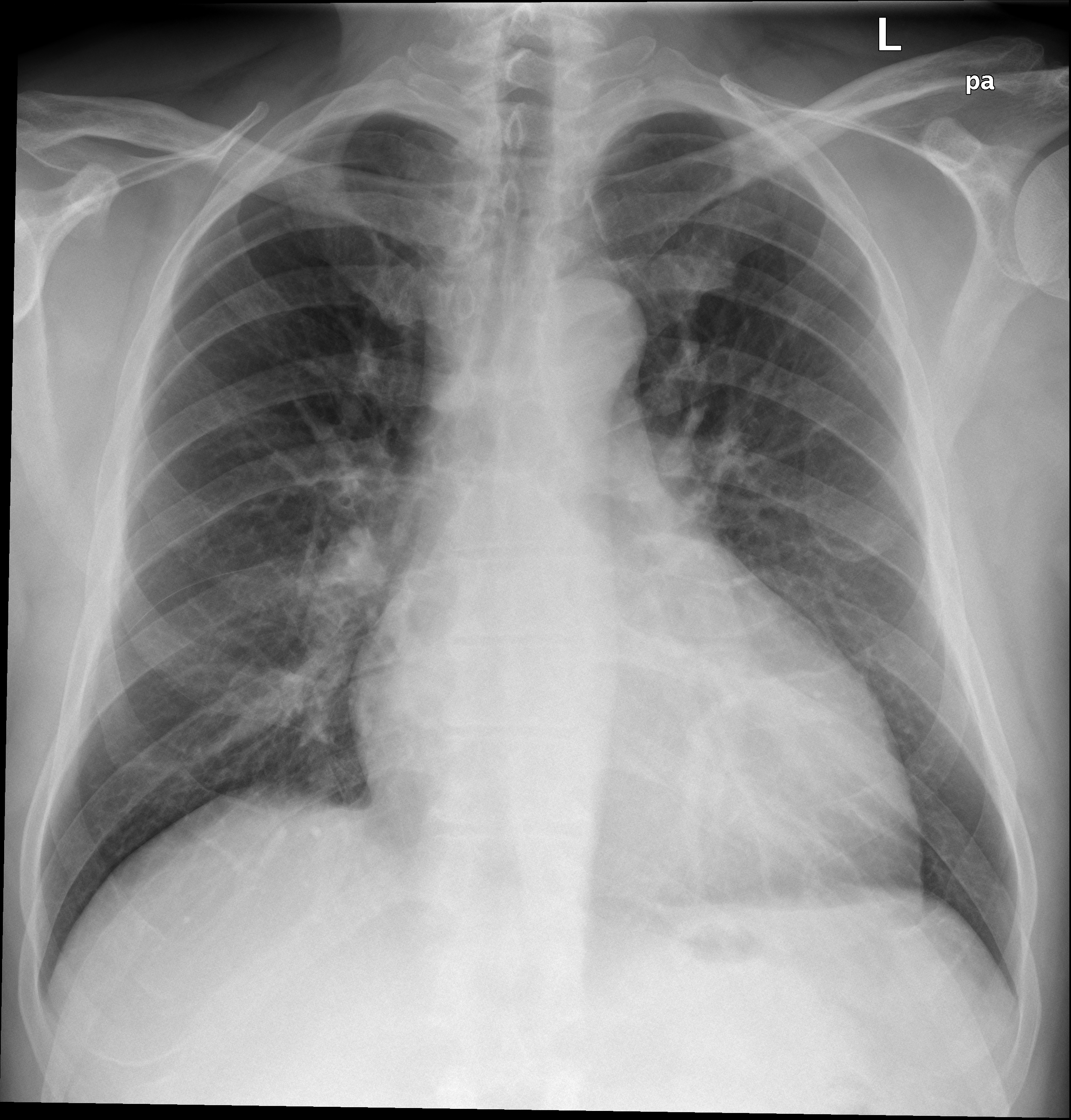

Supplement: Supplementary file 2 — Electronic Supplementary Material [file 330_2024_11115_MOESM2_ESM.zip › Digital Supplementary Material/Radiography/16Radiography.PNG]

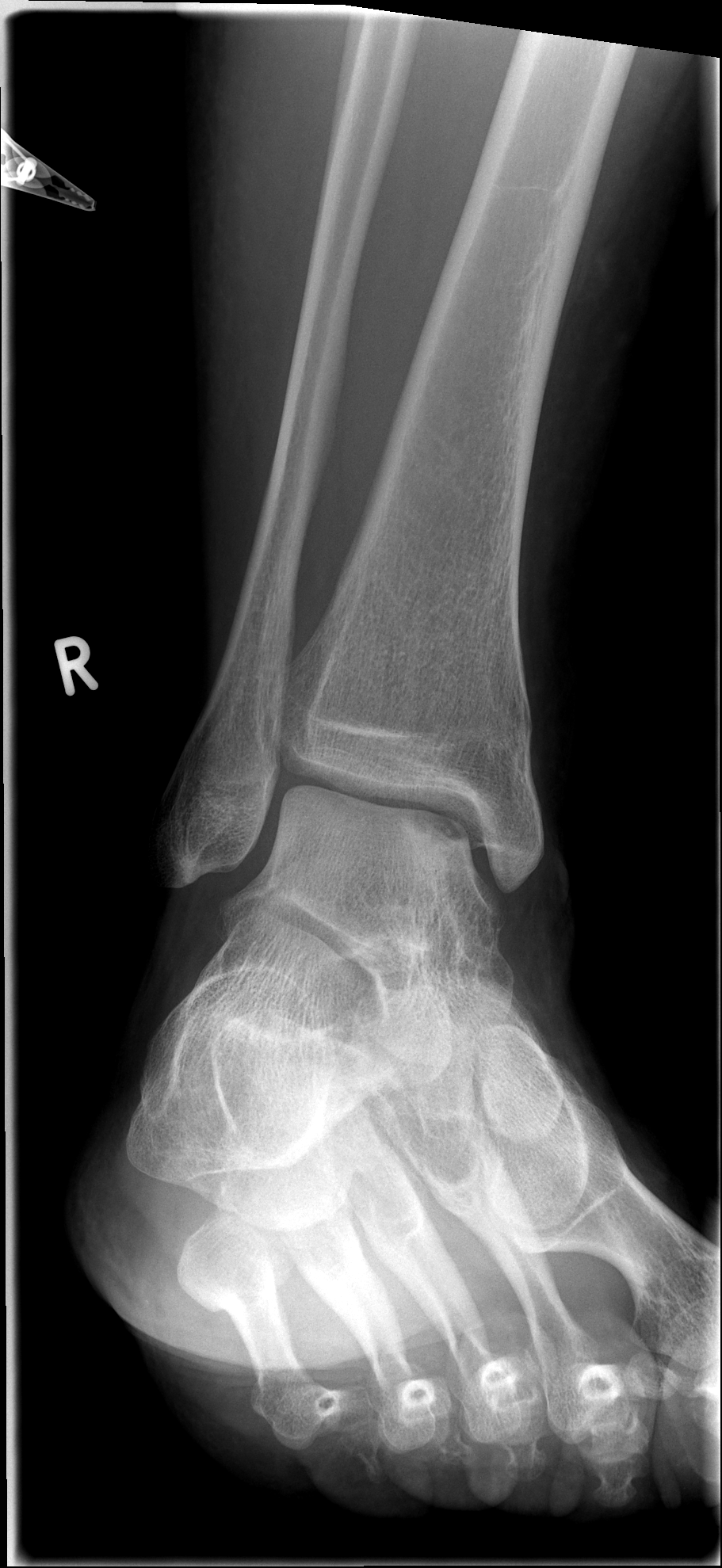

Supplement: Supplementary file 2 — Electronic Supplementary Material [file 330_2024_11115_MOESM2_ESM.zip › Digital Supplementary Material/Radiography/60Radiography.PNG]

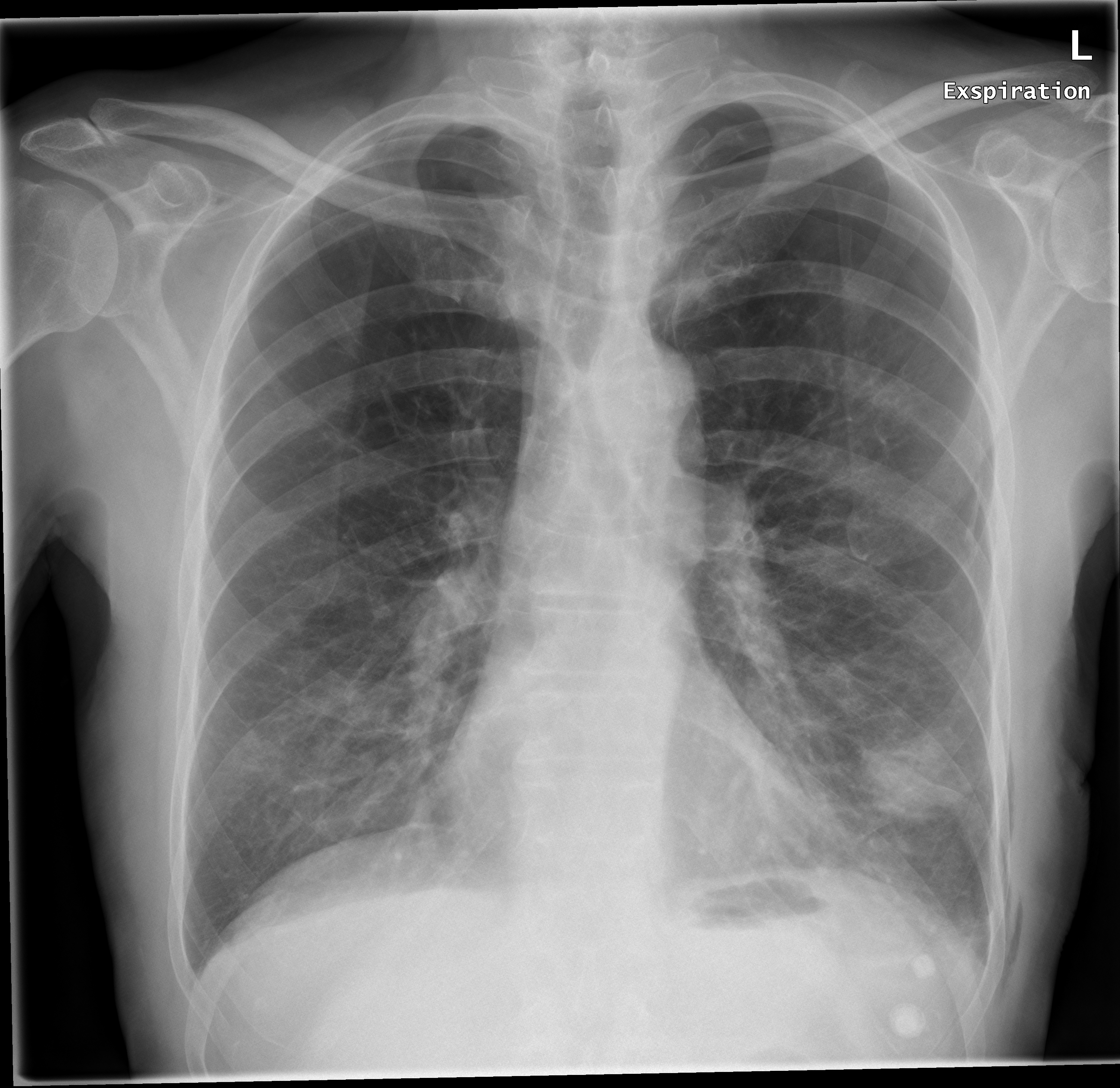

Supplement: Supplementary file 2 — Electronic Supplementary Material [file 330_2024_11115_MOESM2_ESM.zip › Digital Supplementary Material/Radiography/11Radiography.PNG]

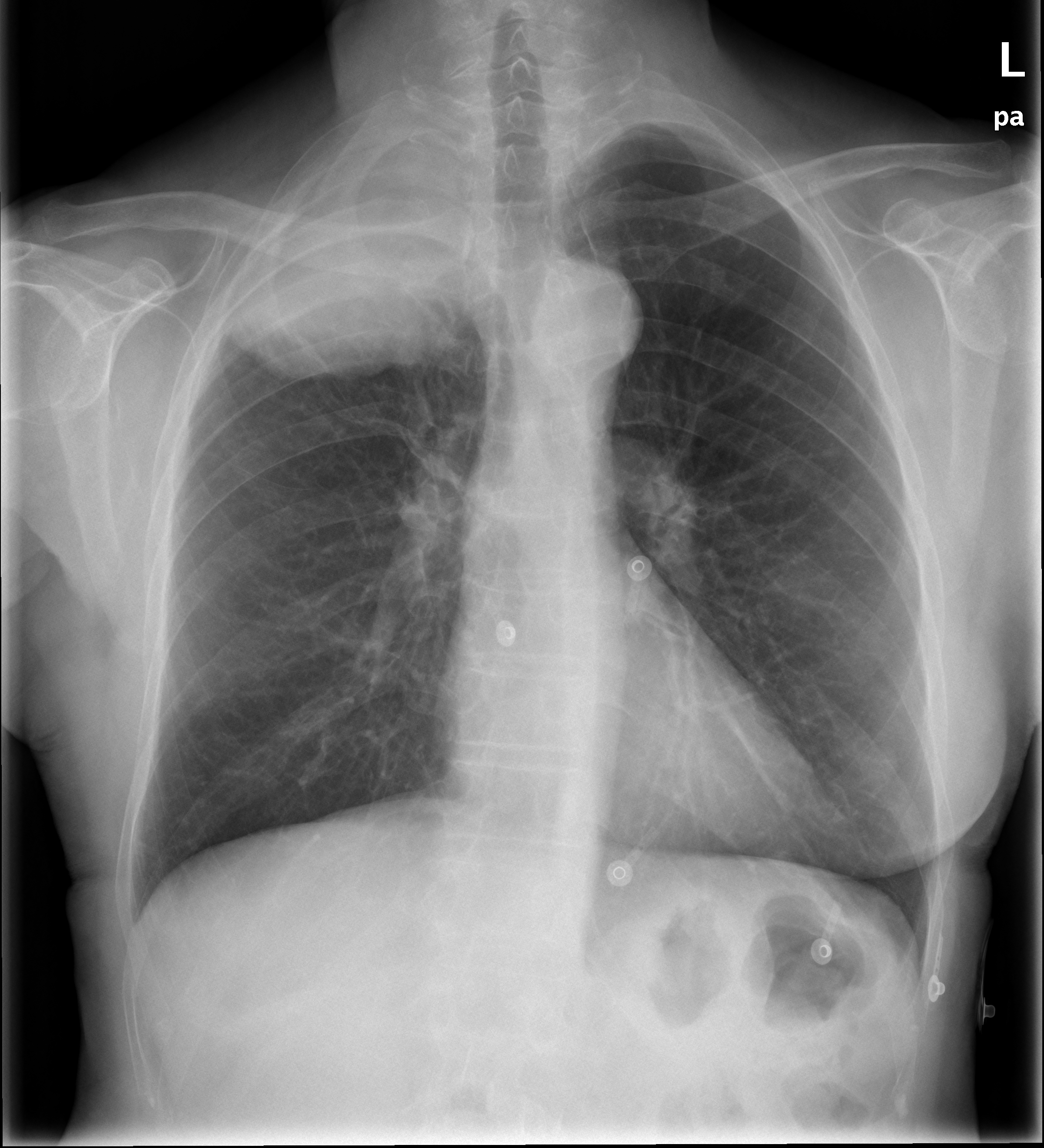

Supplement: Supplementary file 2 — Electronic Supplementary Material [file 330_2024_11115_MOESM2_ESM.zip › Digital Supplementary Material/Radiography/19Radiography.PNG]

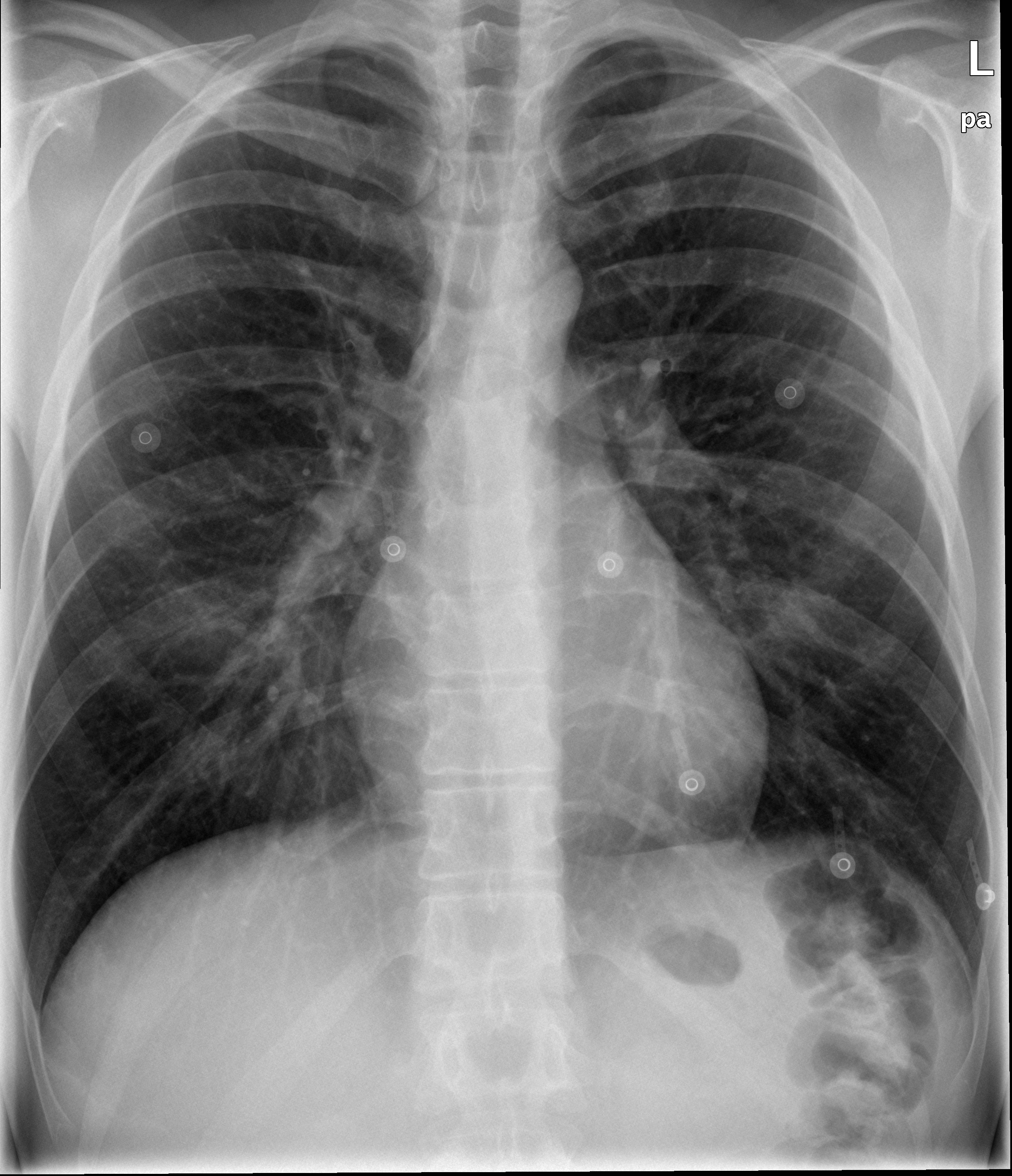

Supplement: Supplementary file 2 — Electronic Supplementary Material [file 330_2024_11115_MOESM2_ESM.zip › Digital Supplementary Material/Radiography/13Radiography.PNG]

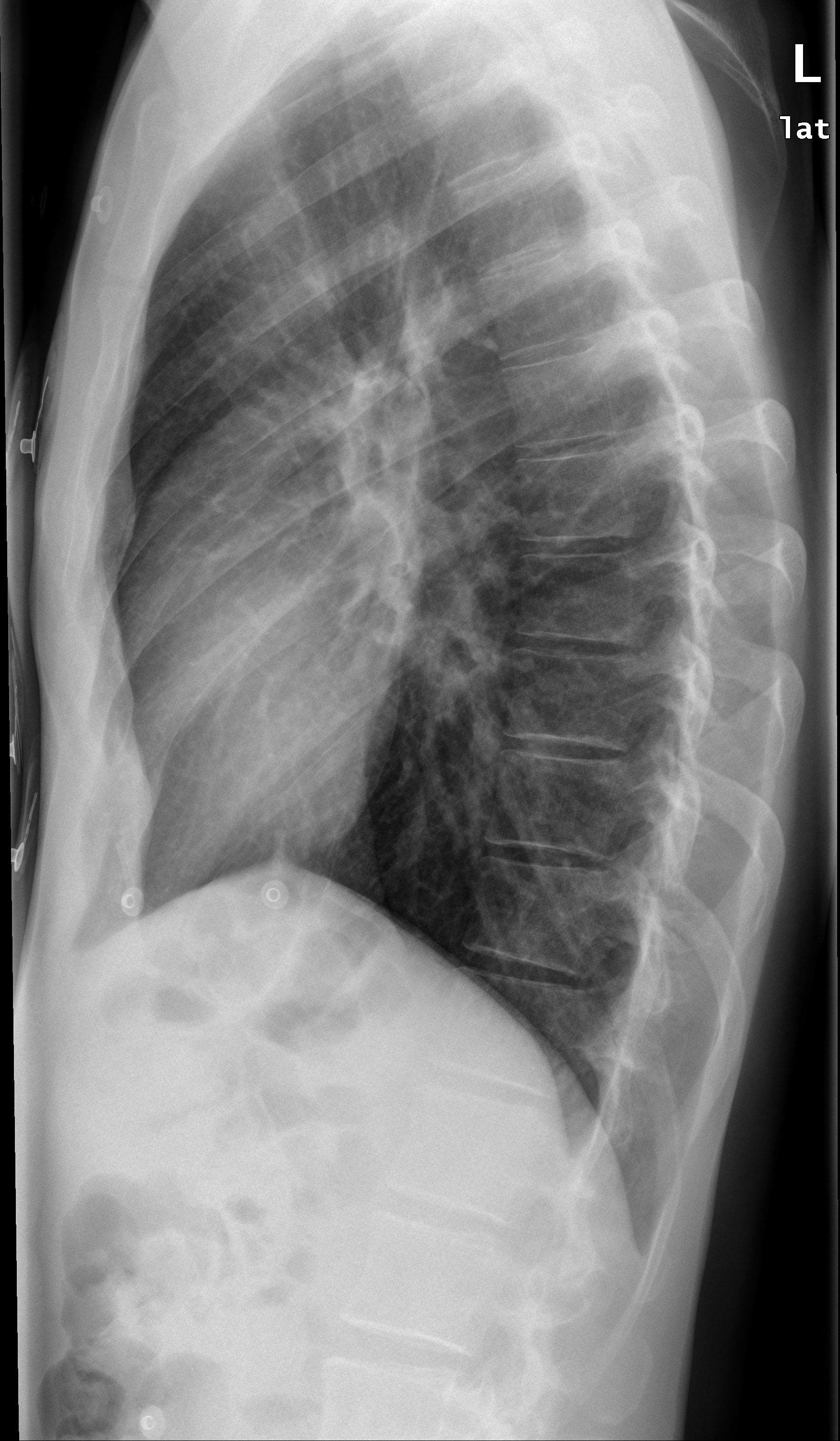

Supplement: Supplementary file 2 — Electronic Supplementary Material [file 330_2024_11115_MOESM2_ESM.zip › Digital Supplementary Material/Radiography/14Radiography.PNG]

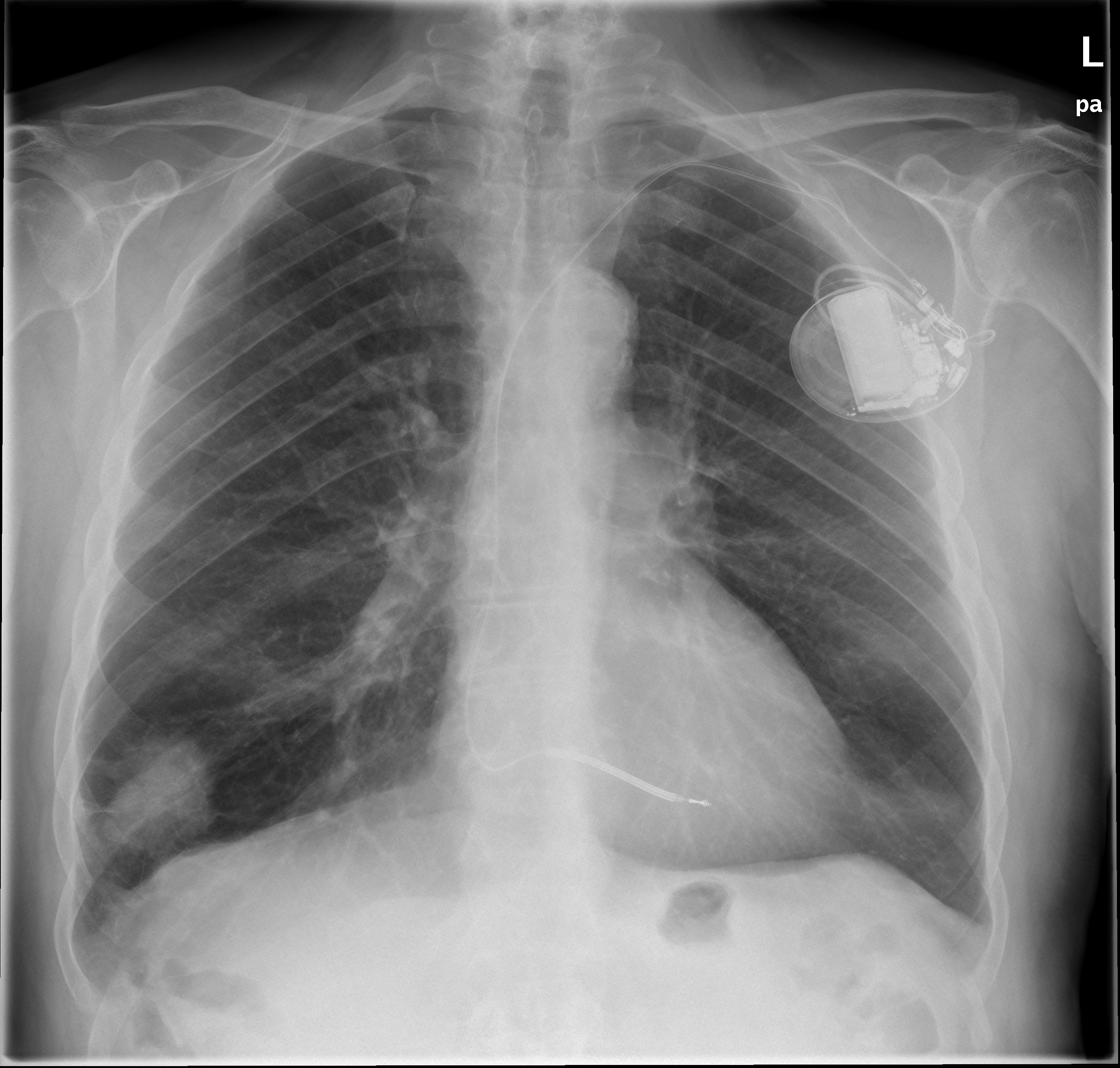

Supplement: Supplementary file 2 — Electronic Supplementary Material [file 330_2024_11115_MOESM2_ESM.zip › Digital Supplementary Material/Radiography/15Radiography.PNG]

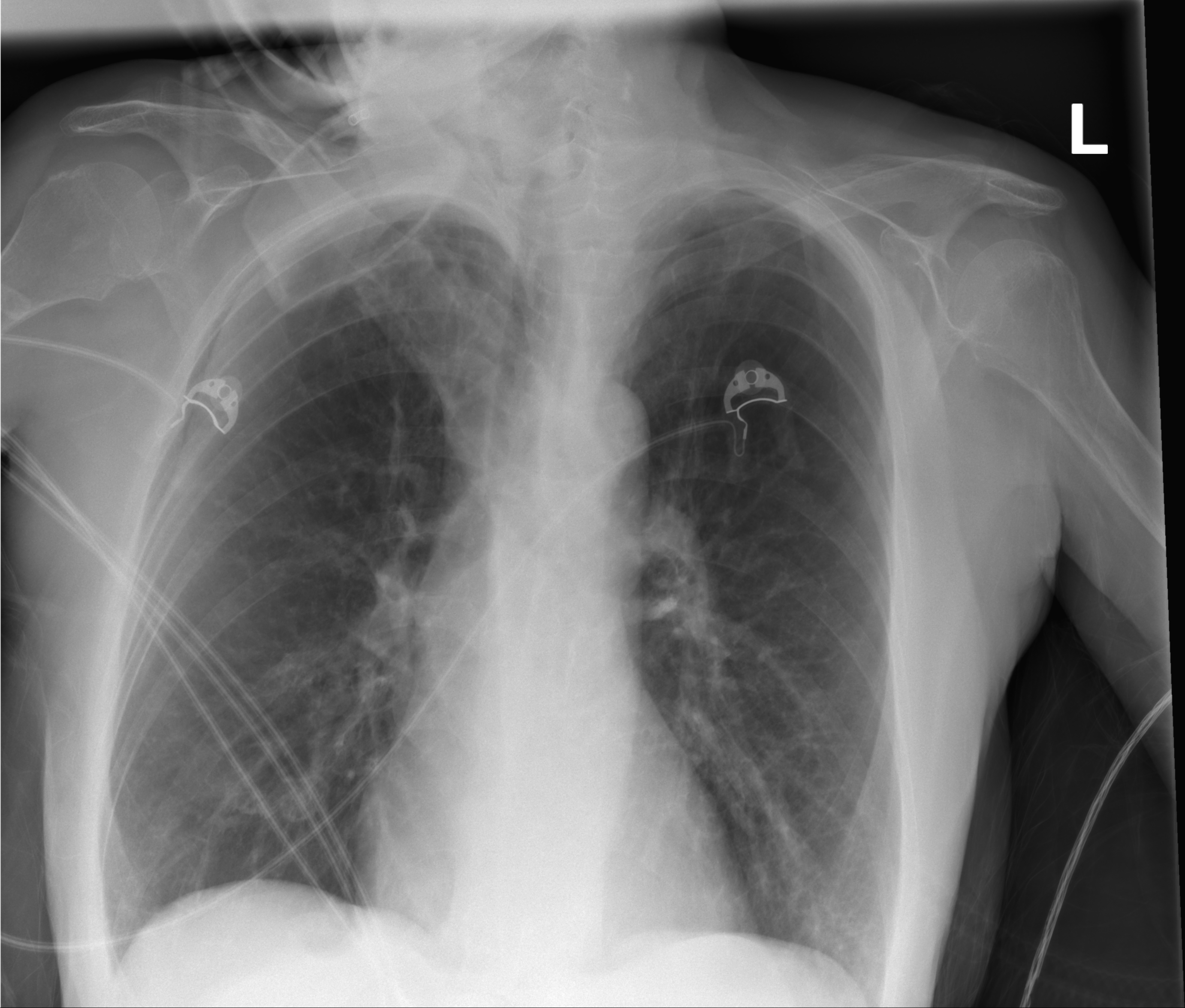

Supplement: Supplementary file 2 — Electronic Supplementary Material [file 330_2024_11115_MOESM2_ESM.zip › Digital Supplementary Material/Radiography/12Radiography.PNG]

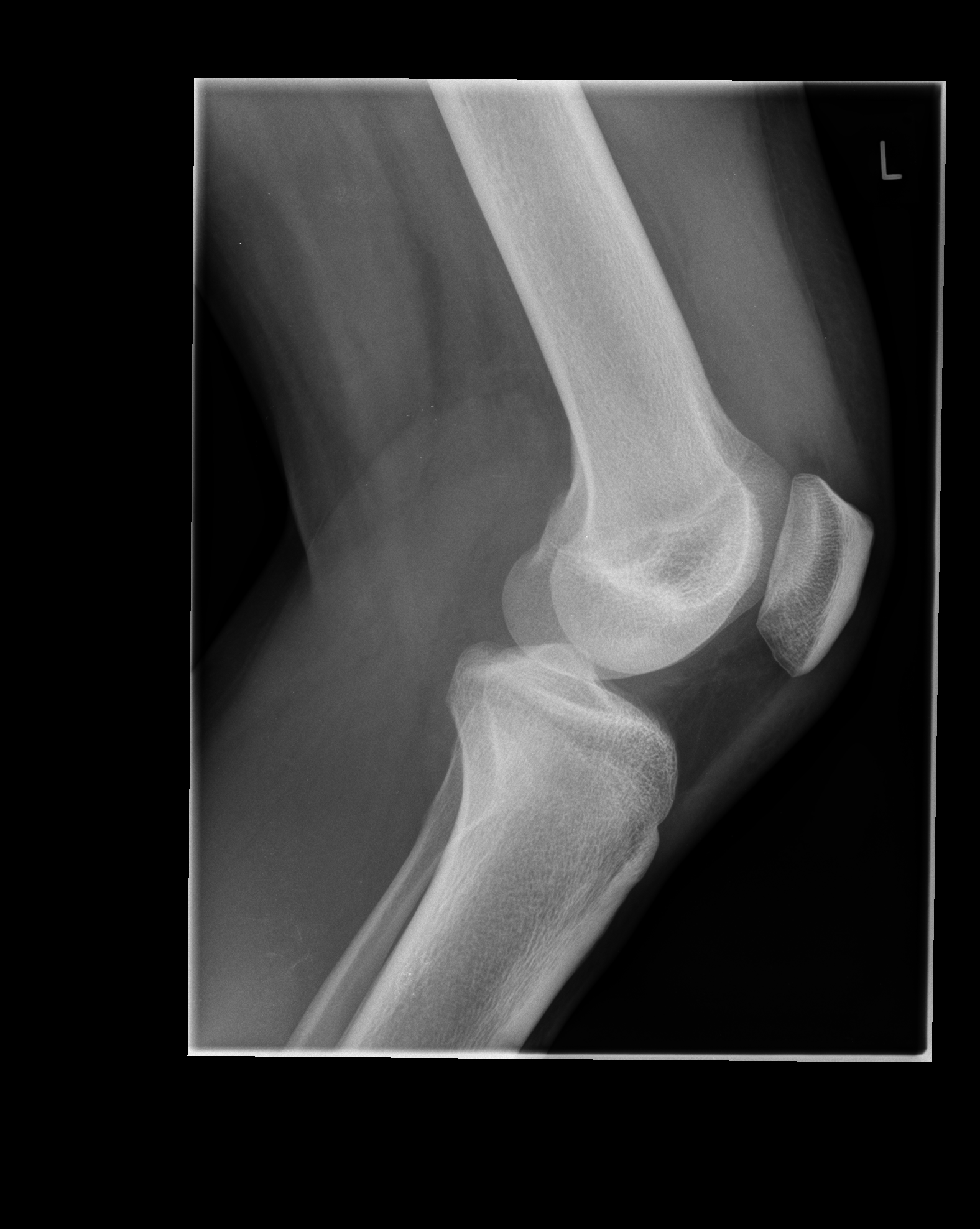

Supplement: Supplementary file 2 — Electronic Supplementary Material [file 330_2024_11115_MOESM2_ESM.zip › Digital Supplementary Material/Radiography/33Radiography.PNG]

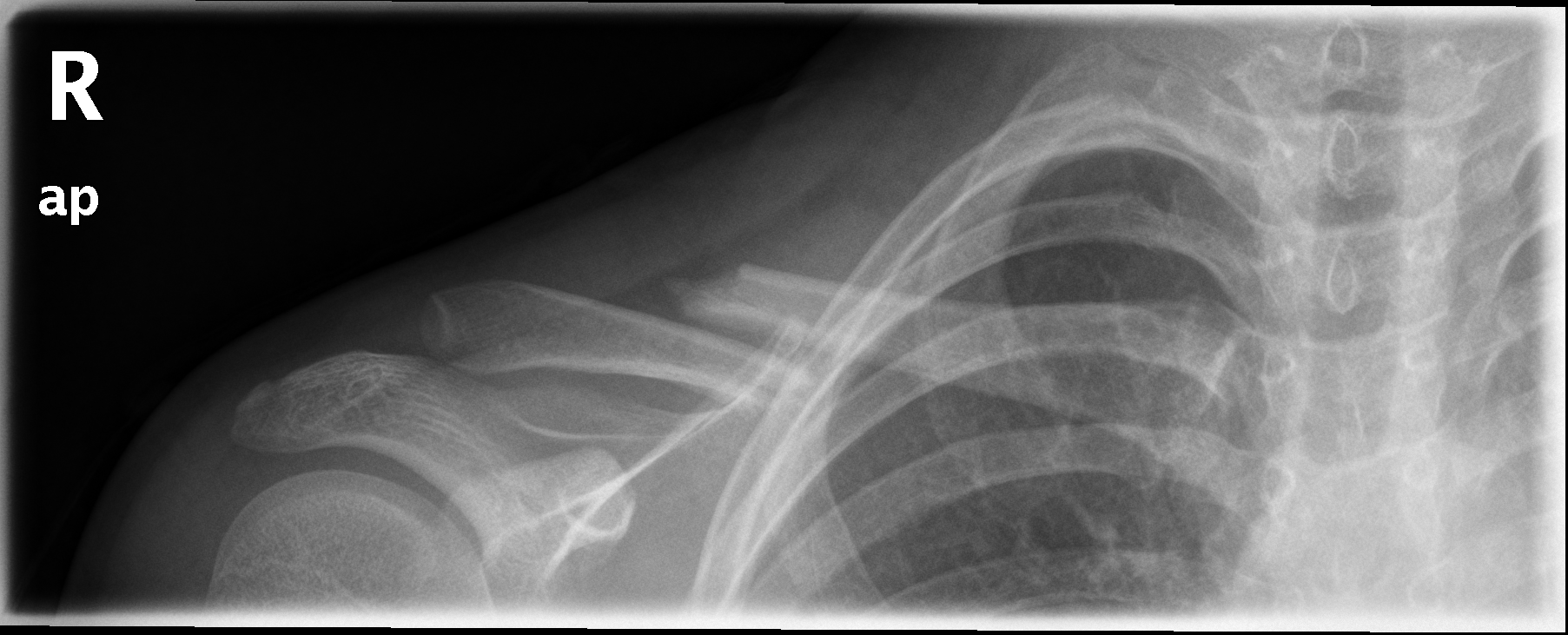

Supplement: Supplementary file 2 — Electronic Supplementary Material [file 330_2024_11115_MOESM2_ESM.zip › Digital Supplementary Material/Radiography/26Radiography.PNG]

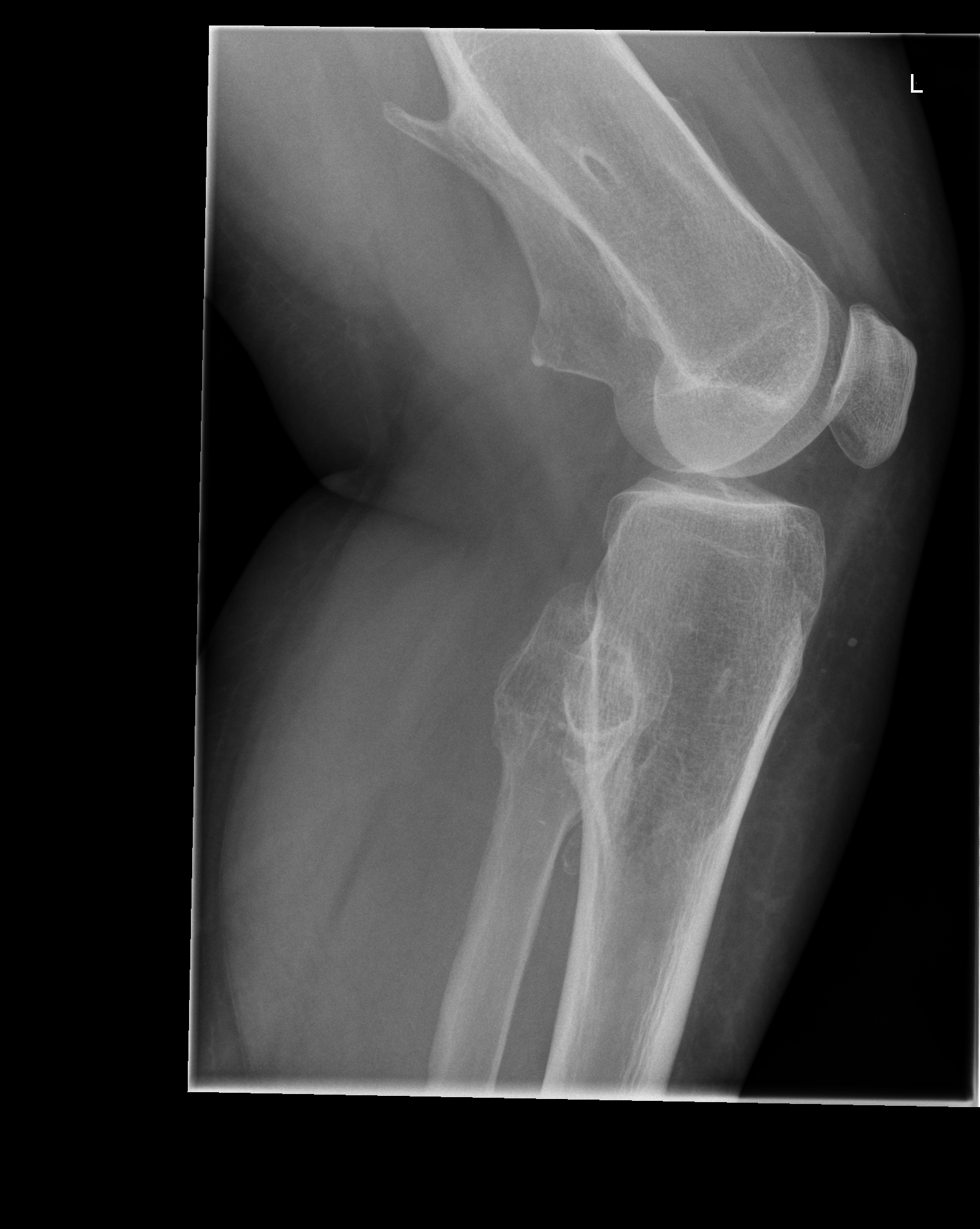

Supplement: Supplementary file 2 — Electronic Supplementary Material [file 330_2024_11115_MOESM2_ESM.zip › Digital Supplementary Material/Radiography/3Radiography.PNG]

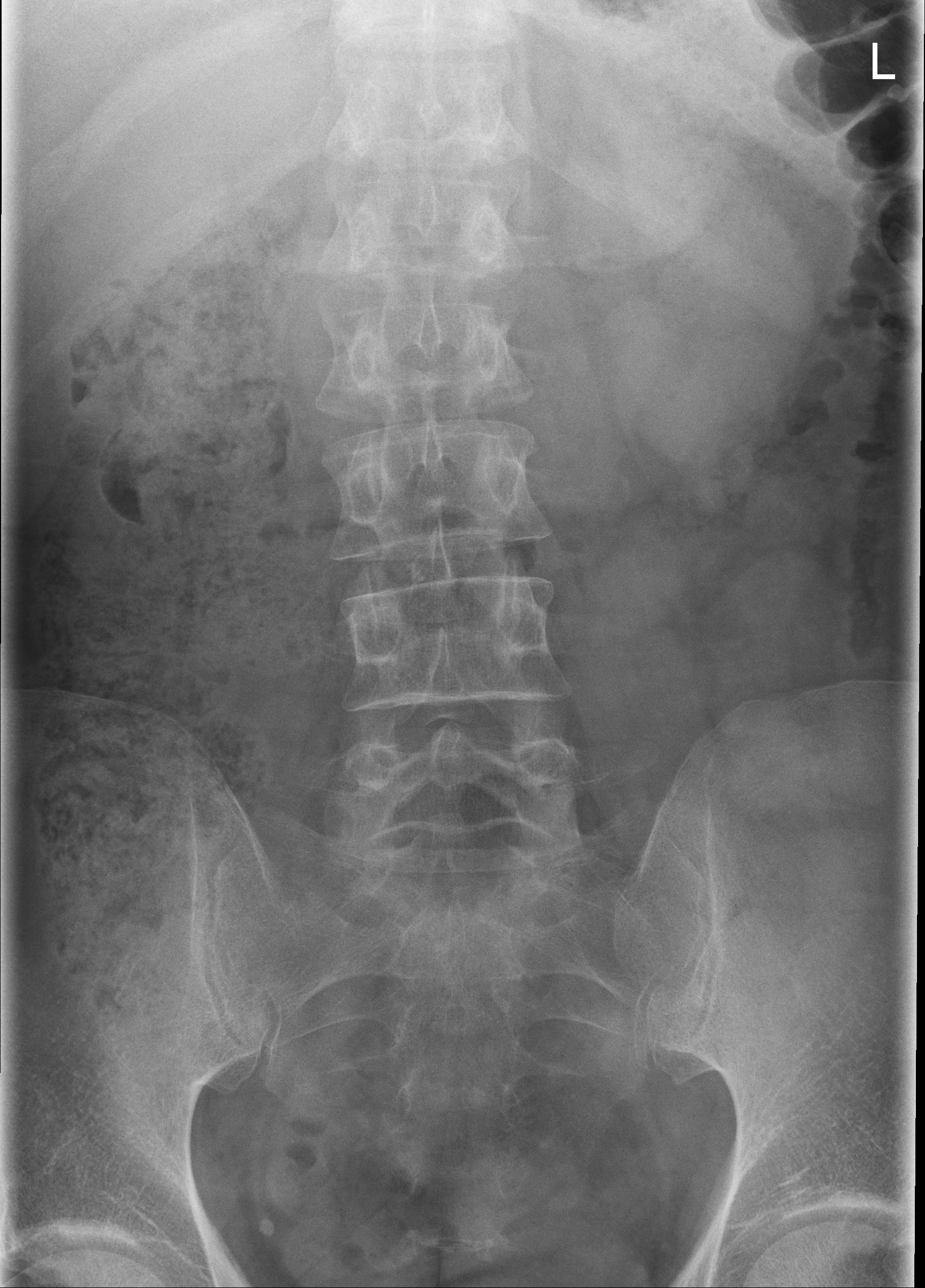

Supplement: Supplementary file 2 — Electronic Supplementary Material [file 330_2024_11115_MOESM2_ESM.zip › Digital Supplementary Material/Radiography/57Radiography.PNG]

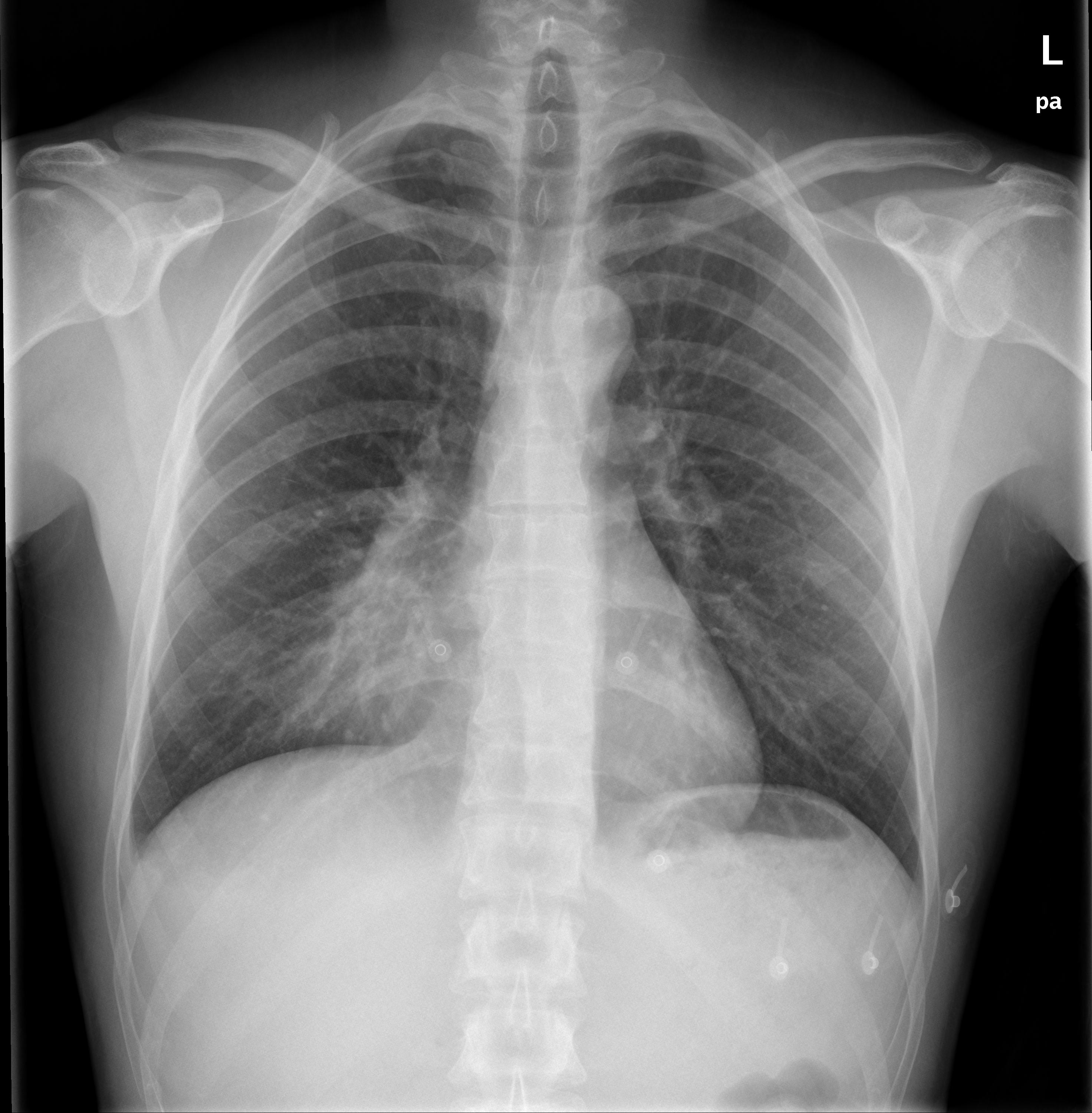

Supplement: Supplementary file 2 — Electronic Supplementary Material [file 330_2024_11115_MOESM2_ESM.zip › Digital Supplementary Material/Radiography/42Radiography.PNG]

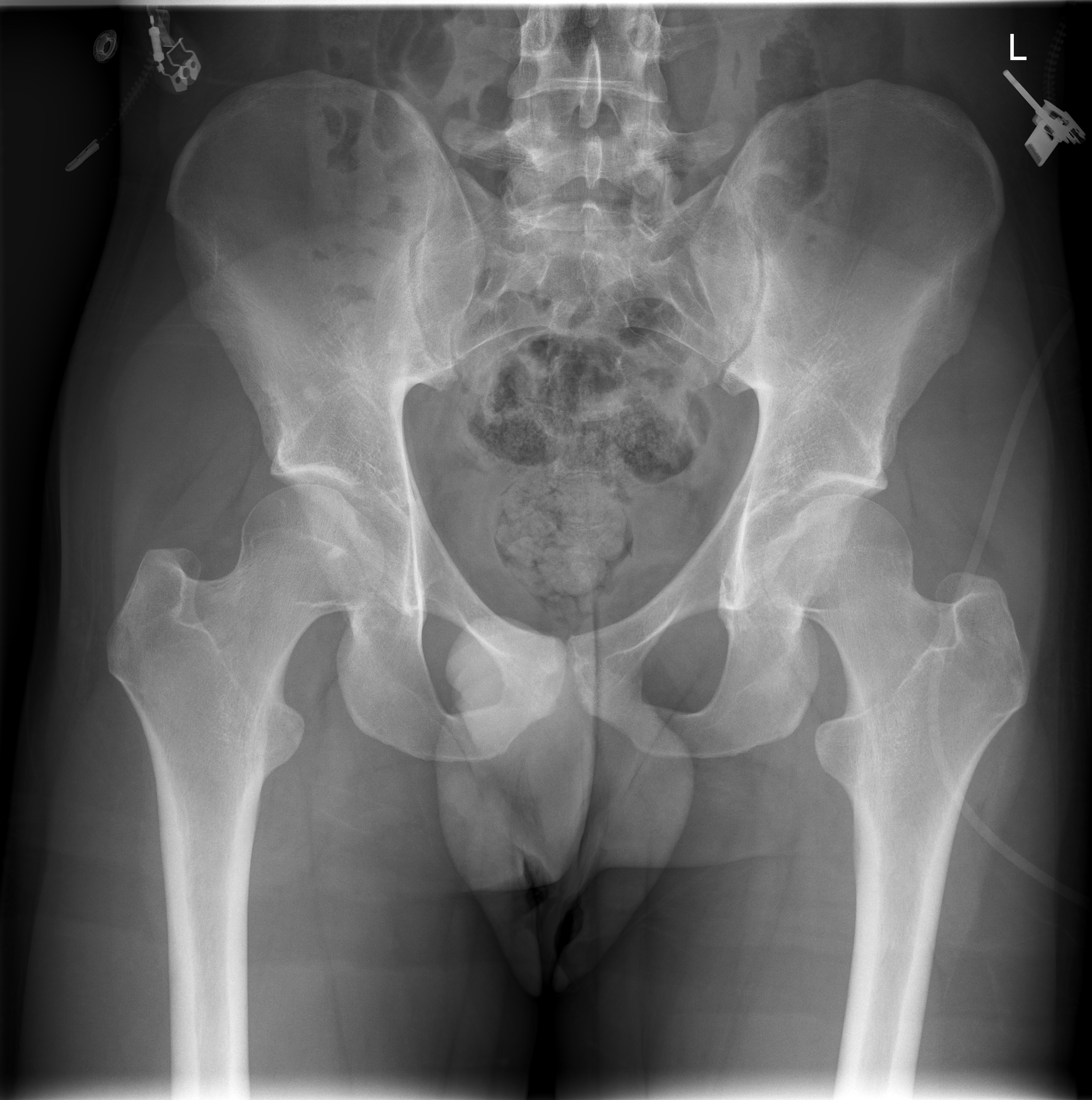

Supplement: Supplementary file 2 — Electronic Supplementary Material [file 330_2024_11115_MOESM2_ESM.zip › Digital Supplementary Material/Radiography/58Radiography.PNG]

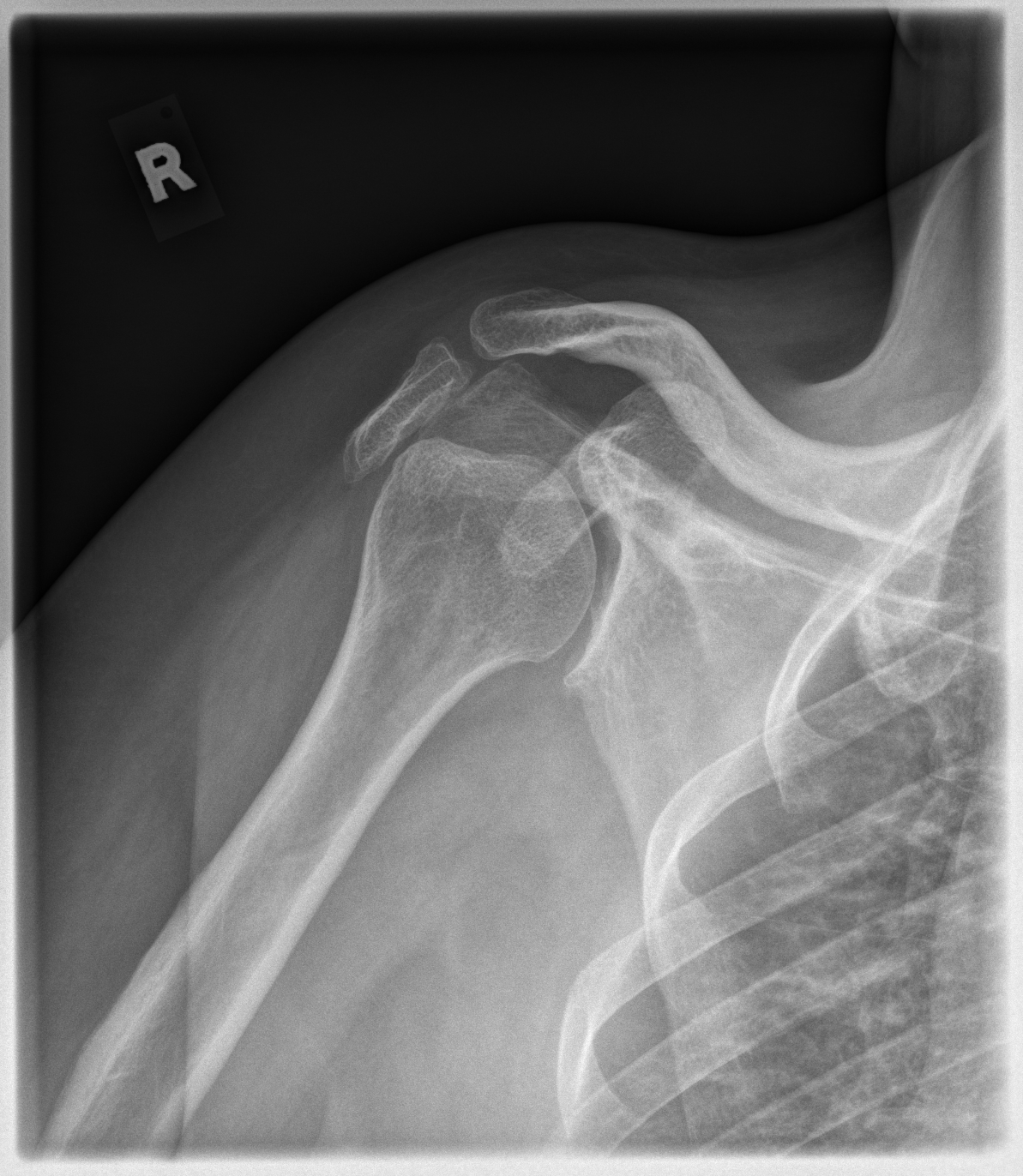

Supplement: Supplementary file 2 — Electronic Supplementary Material [file 330_2024_11115_MOESM2_ESM.zip › Digital Supplementary Material/Radiography/29Radiography.PNG]

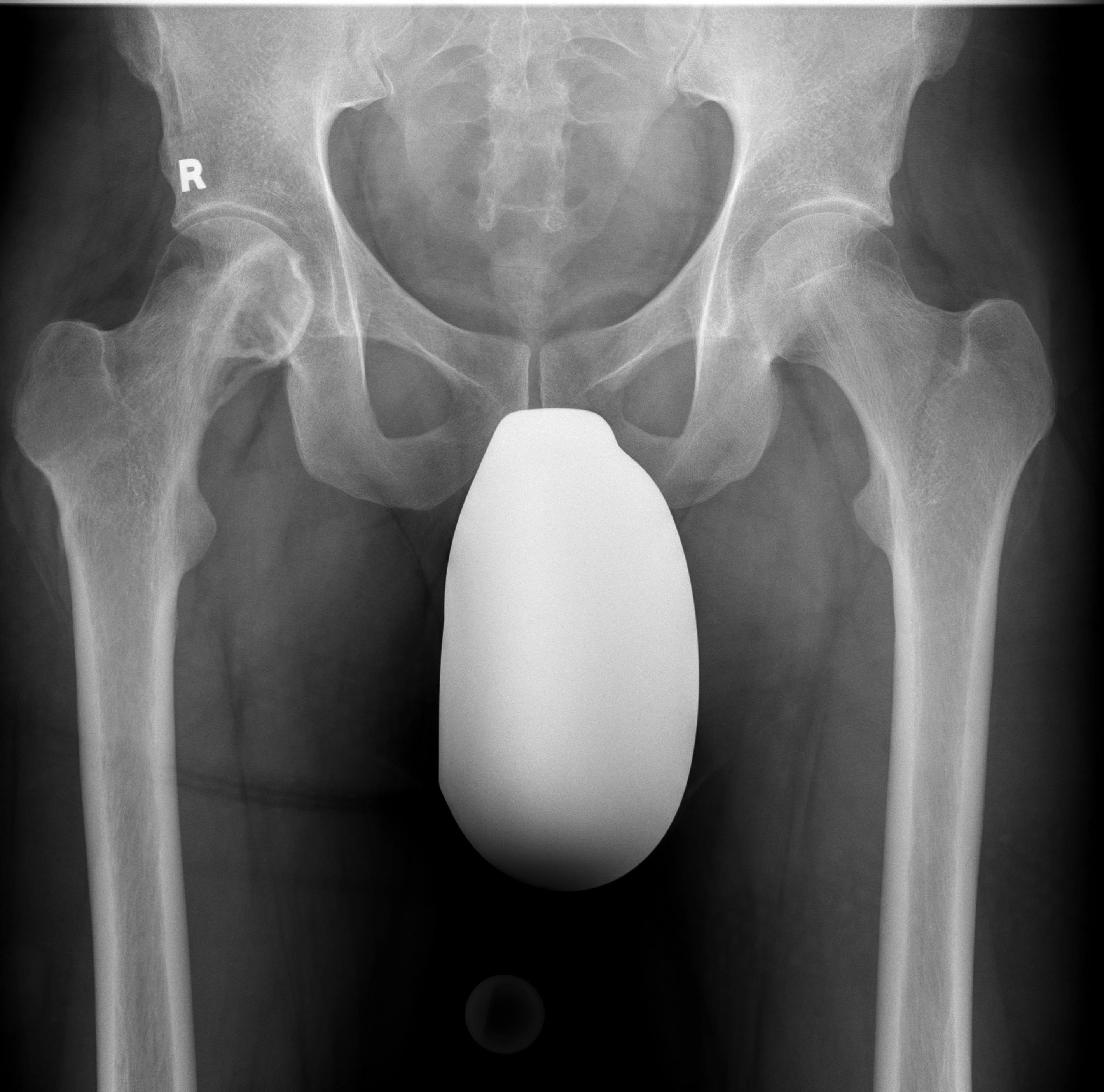

Supplement: Supplementary file 2 — Electronic Supplementary Material [file 330_2024_11115_MOESM2_ESM.zip › Digital Supplementary Material/Radiography/4Radiography.PNG]

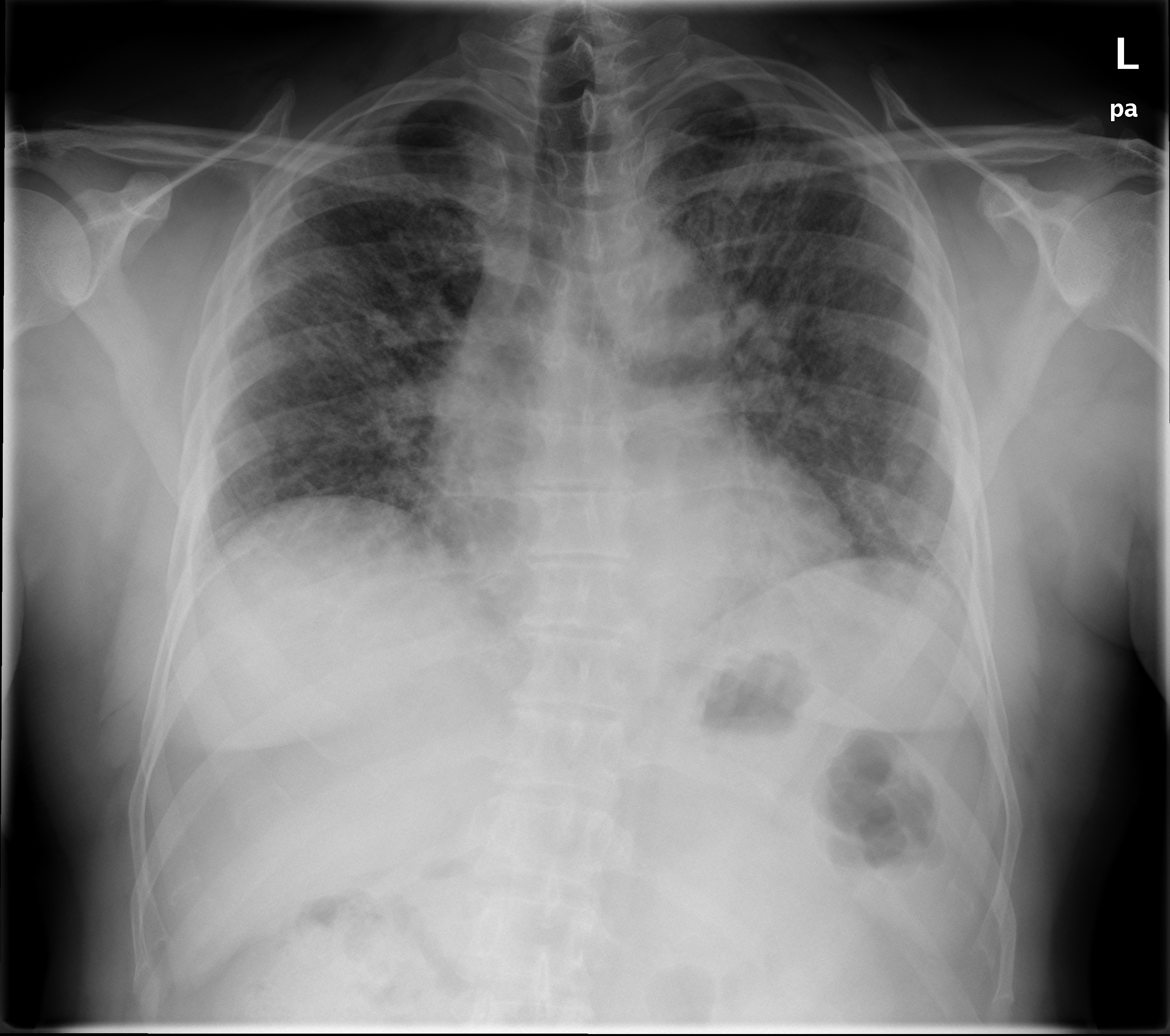

Supplement: Supplementary file 2 — Electronic Supplementary Material [file 330_2024_11115_MOESM2_ESM.zip › Digital Supplementary Material/Radiography/21Radiography.PNG]

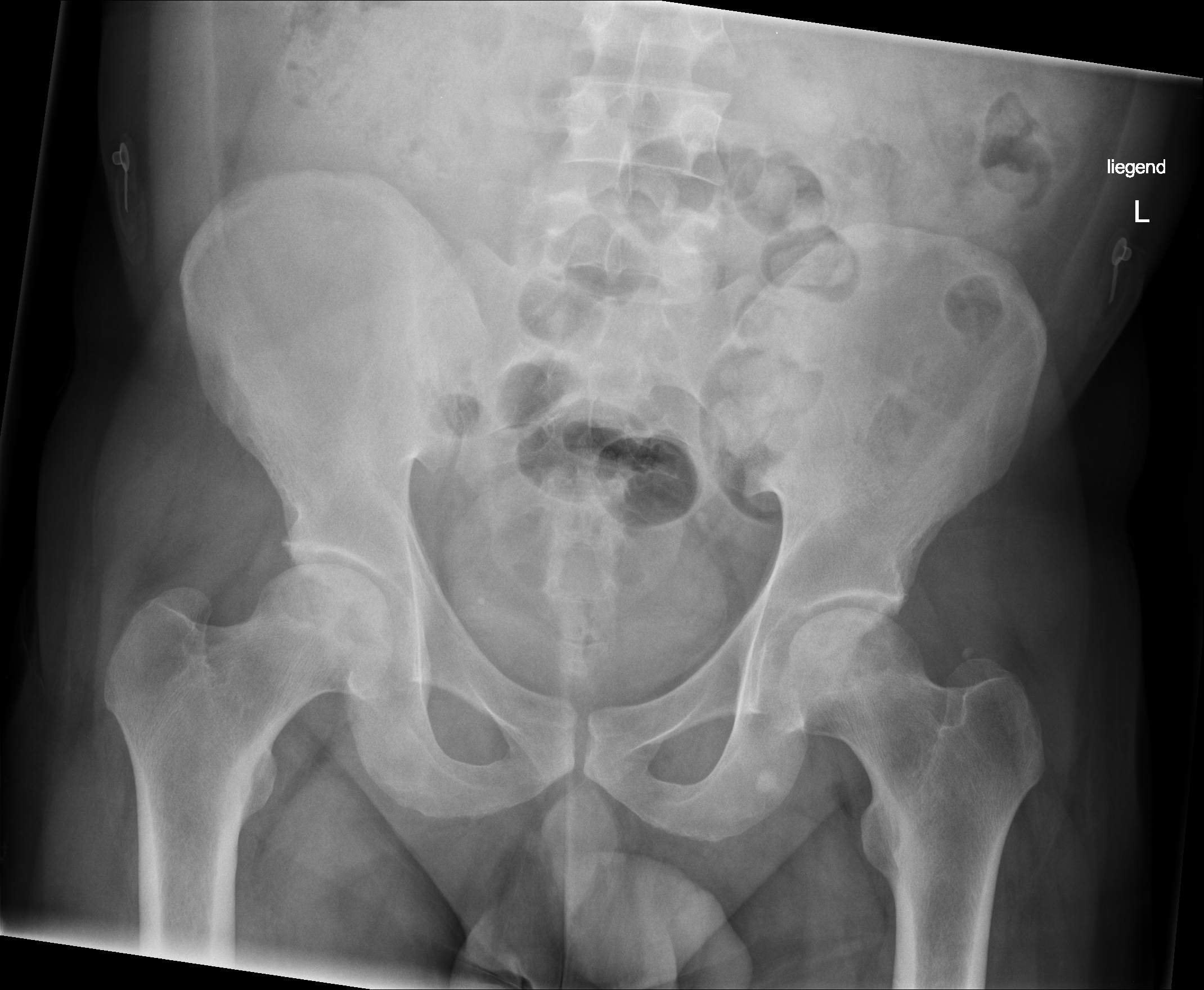

Supplement: Supplementary file 2 — Electronic Supplementary Material [file 330_2024_11115_MOESM2_ESM.zip › Digital Supplementary Material/Radiography/34Radiography.PNG]

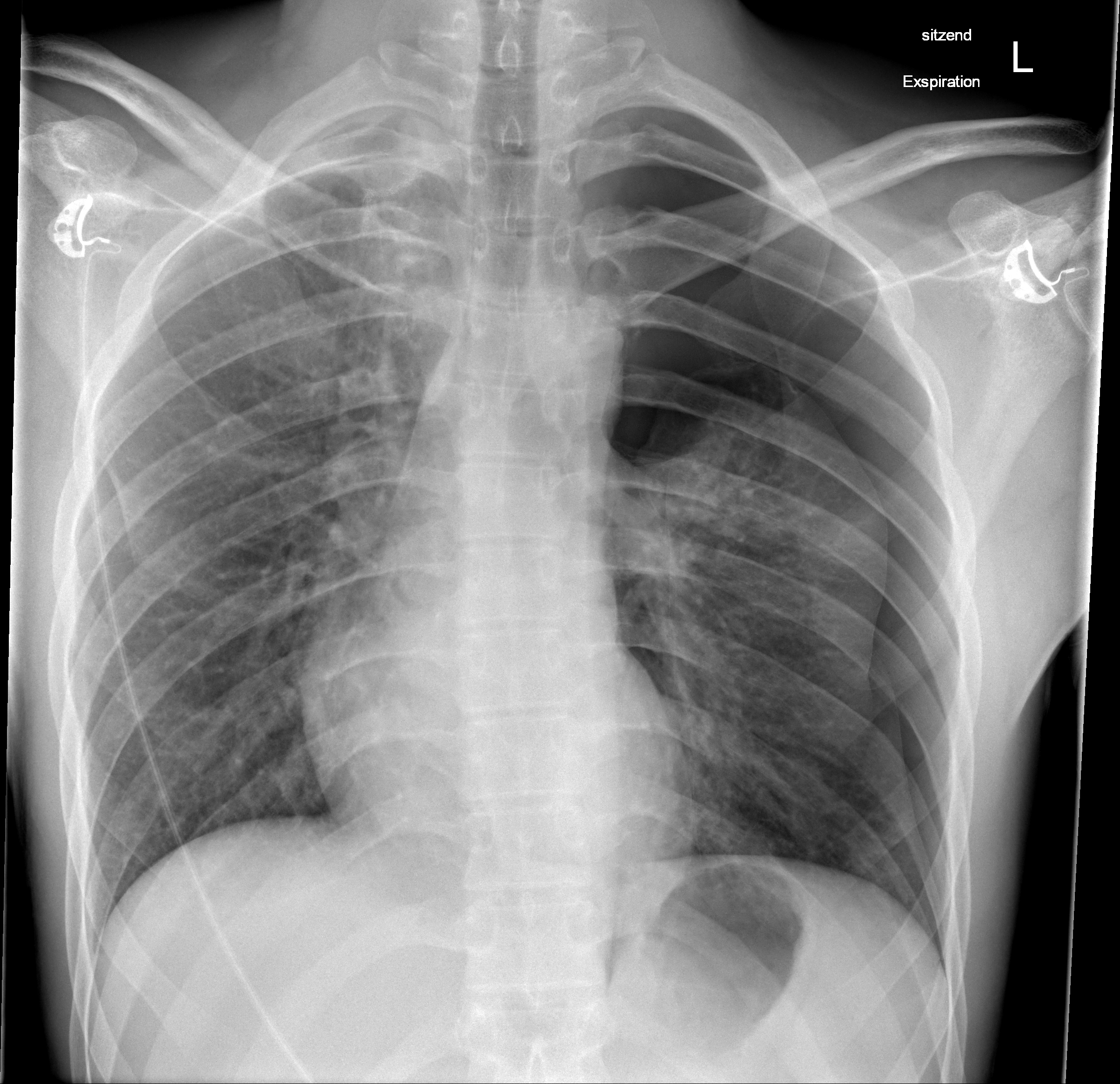

Supplement: Supplementary file 2 — Electronic Supplementary Material [file 330_2024_11115_MOESM2_ESM.zip › Digital Supplementary Material/Radiography/45Radiography.PNG]

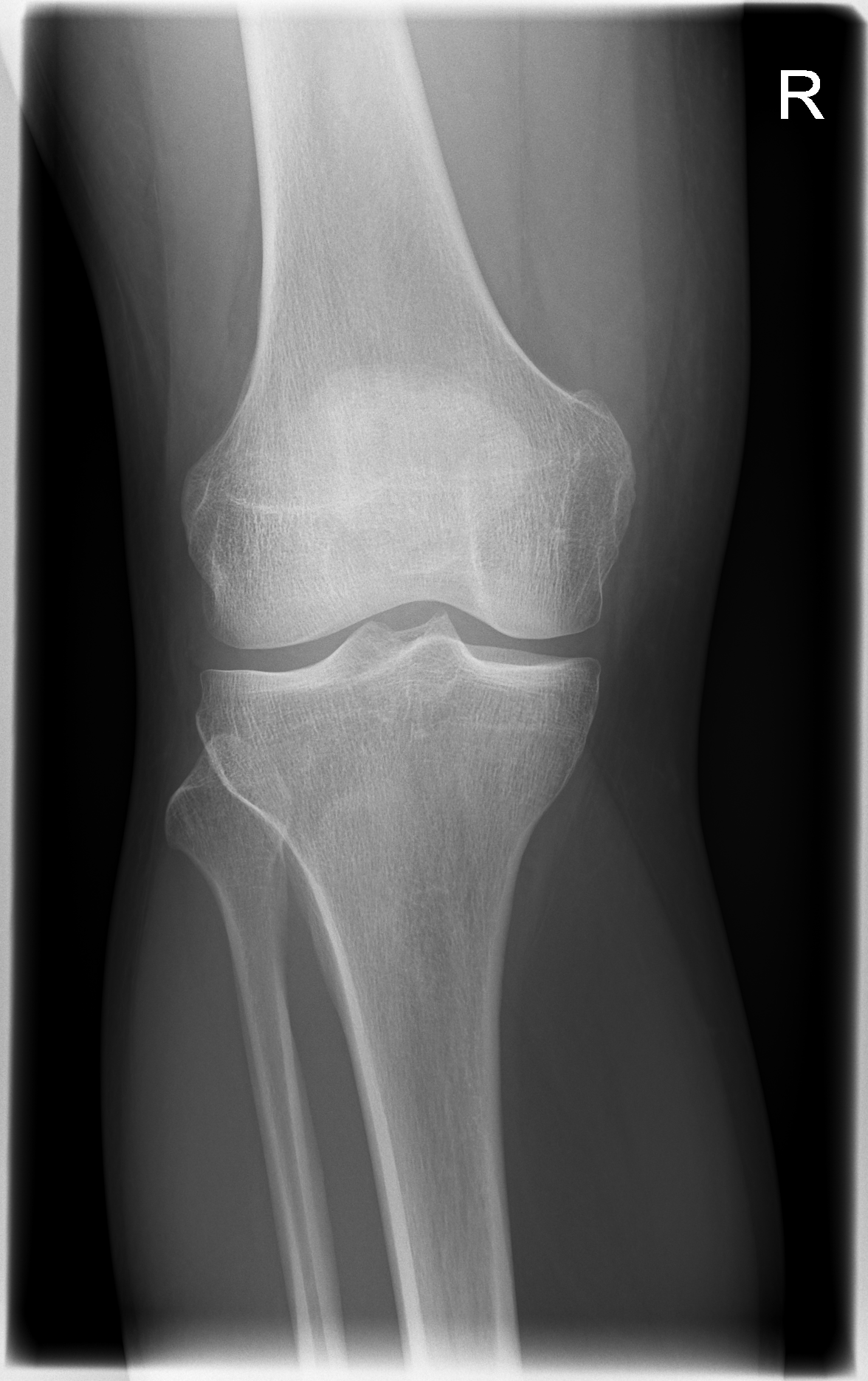

Supplement: Supplementary file 2 — Electronic Supplementary Material [file 330_2024_11115_MOESM2_ESM.zip › Digital Supplementary Material/Radiography/50Radiography.PNG]

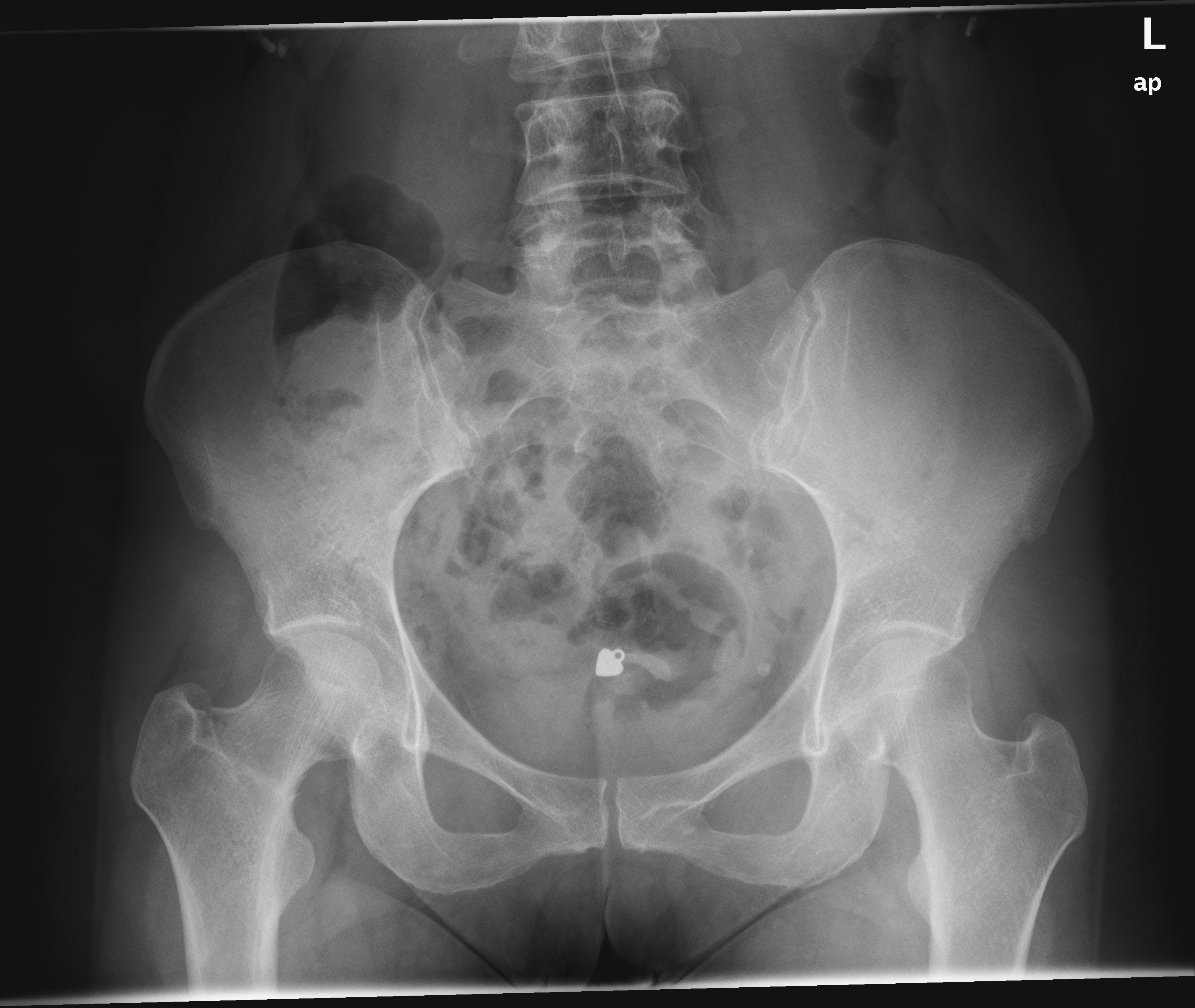

Supplement: Supplementary file 2 — Electronic Supplementary Material [file 330_2024_11115_MOESM2_ESM.zip › Digital Supplementary Material/Radiography/35Radiography.PNG]

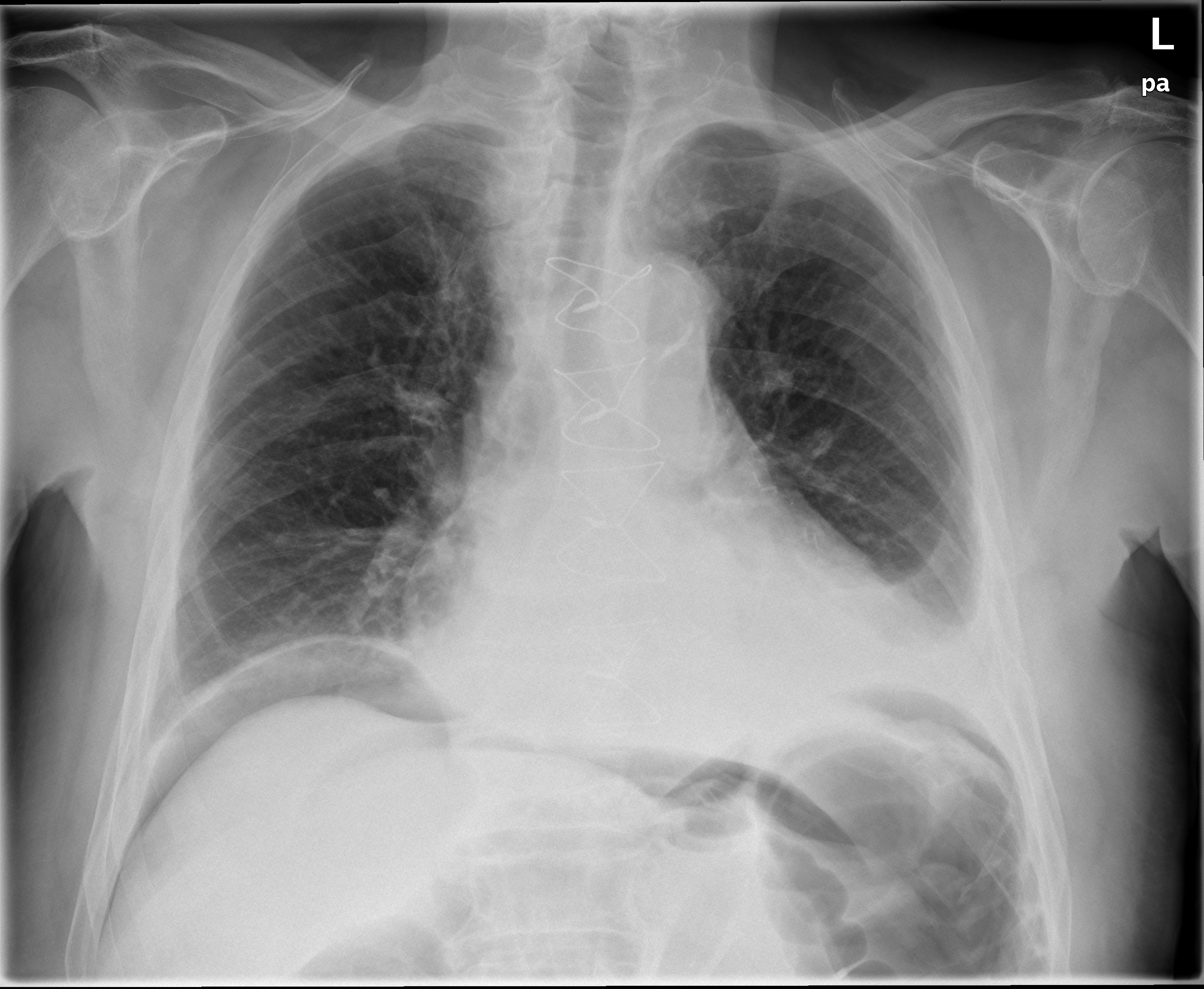

Supplement: Supplementary file 2 — Electronic Supplementary Material [file 330_2024_11115_MOESM2_ESM.zip › Digital Supplementary Material/Radiography/20Radiography.PNG]

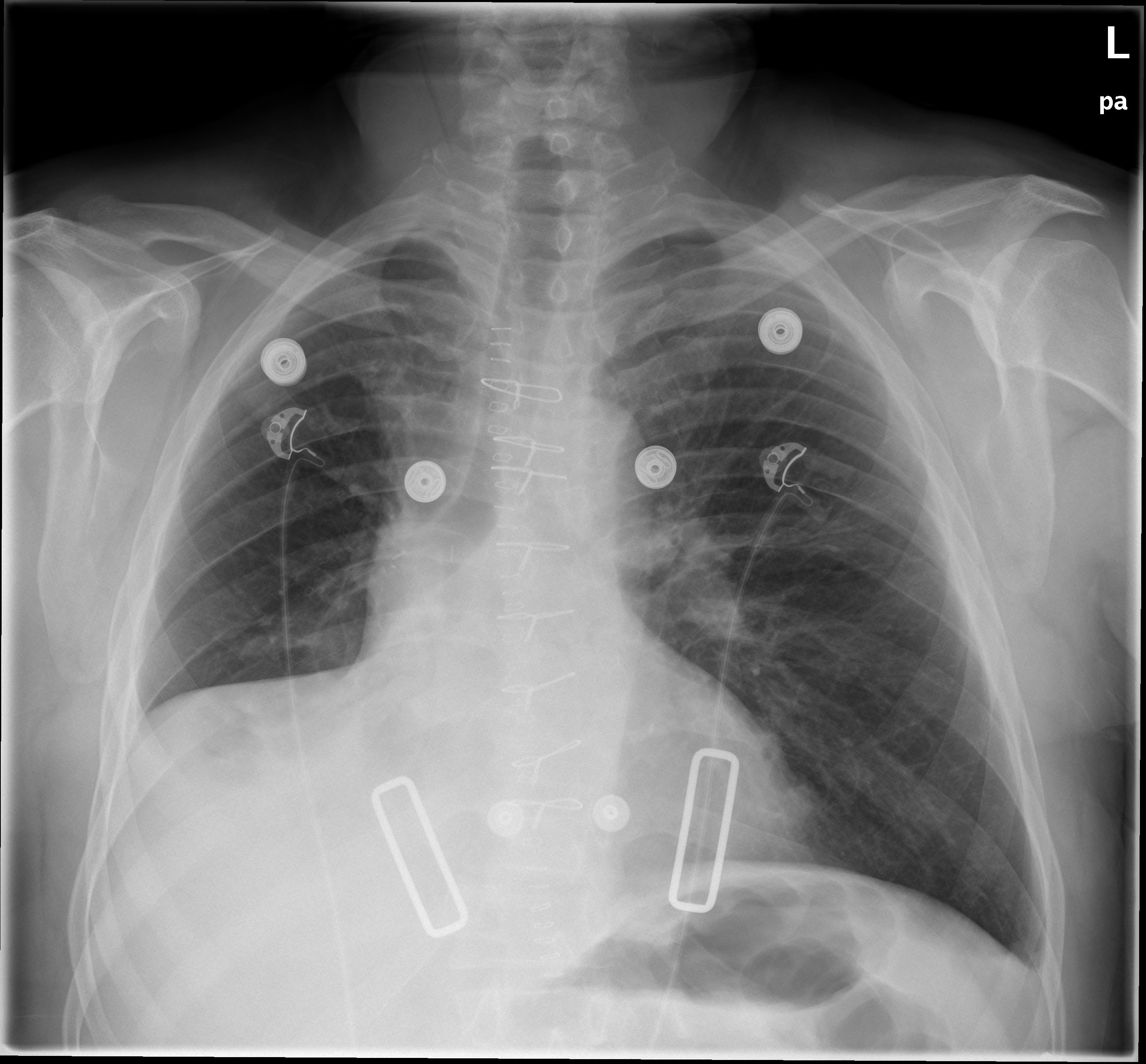

Supplement: Supplementary file 2 — Electronic Supplementary Material [file 330_2024_11115_MOESM2_ESM.zip › Digital Supplementary Material/Radiography/5Radiography.PNG]

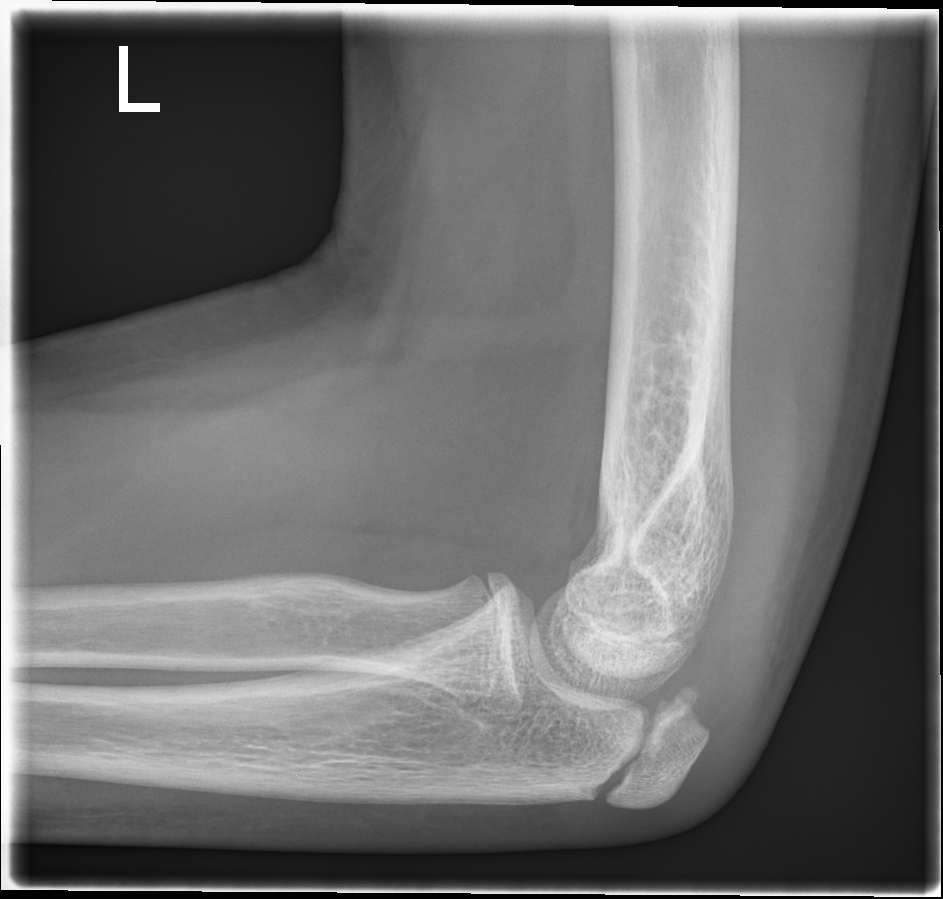

Supplement: Supplementary file 2 — Electronic Supplementary Material [file 330_2024_11115_MOESM2_ESM.zip › Digital Supplementary Material/Radiography/51Radiography.PNG]

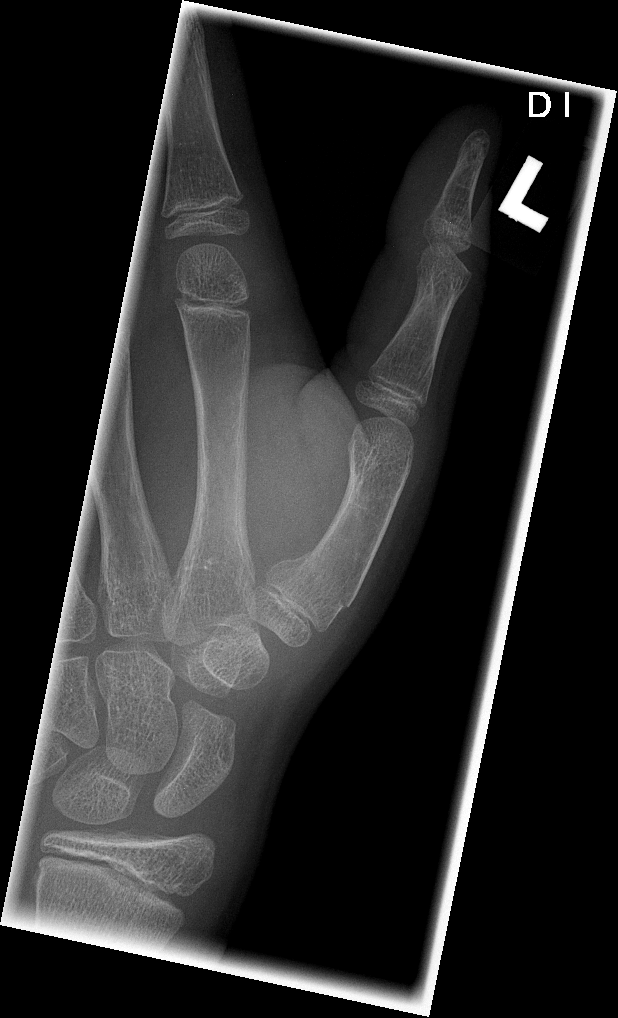

Supplement: Supplementary file 2 — Electronic Supplementary Material [file 330_2024_11115_MOESM2_ESM.zip › Digital Supplementary Material/Radiography/44Radiography.PNG]

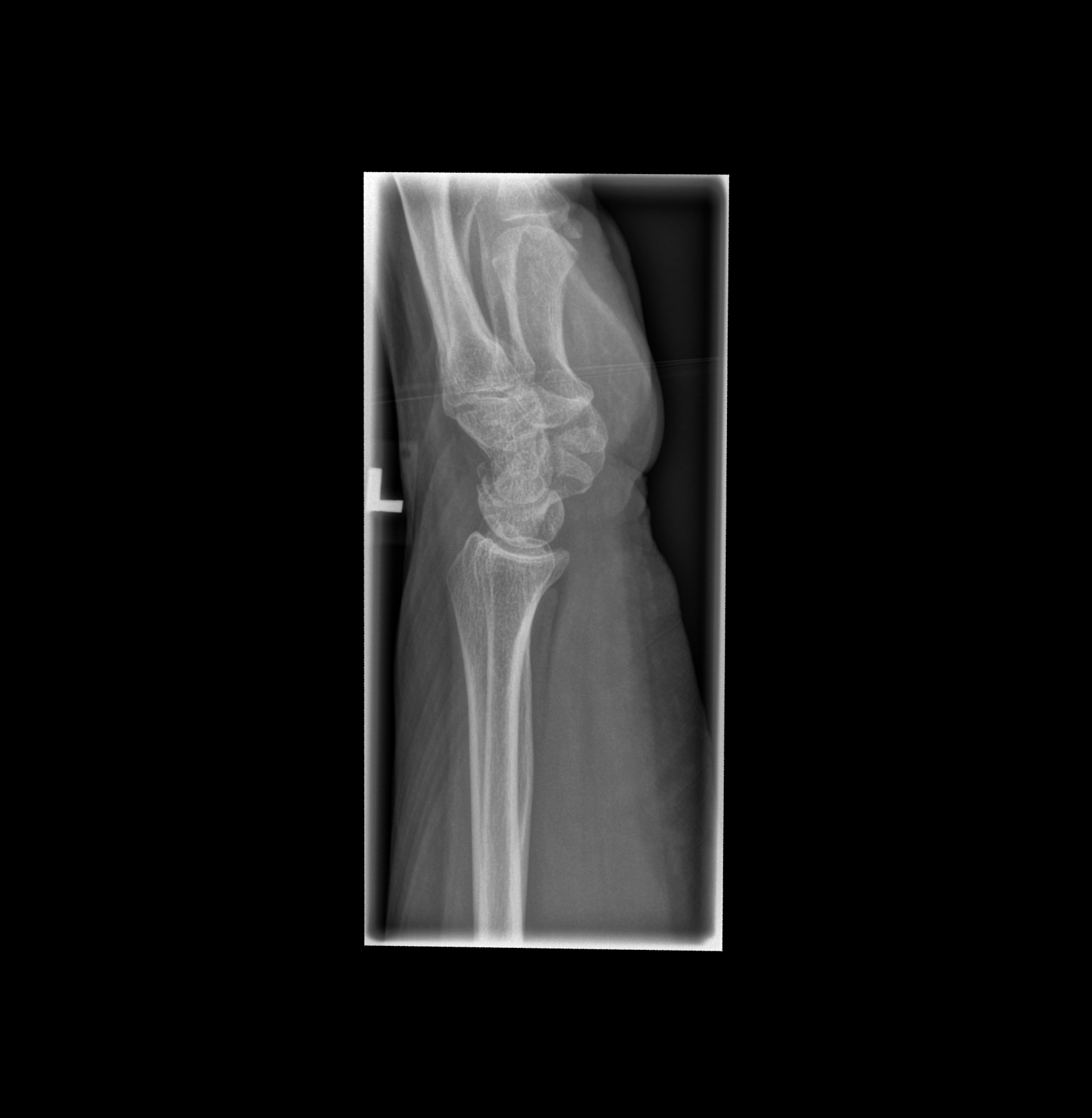

Supplement: Supplementary file 2 — Electronic Supplementary Material [file 330_2024_11115_MOESM2_ESM.zip › Digital Supplementary Material/Radiography/59Radiography.PNG]

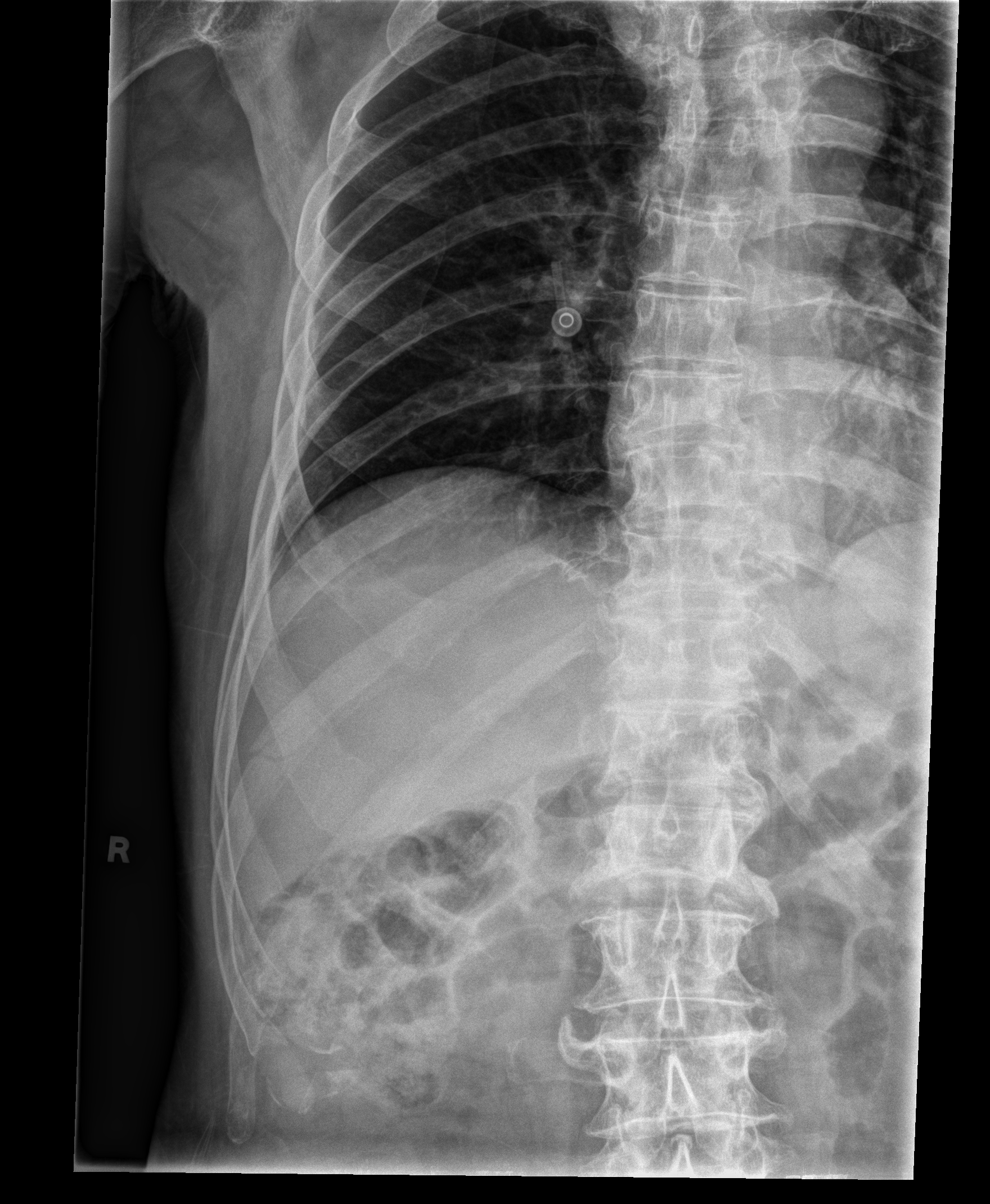

Supplement: Supplementary file 2 — Electronic Supplementary Material [file 330_2024_11115_MOESM2_ESM.zip › Digital Supplementary Material/Radiography/28Radiography.PNG]

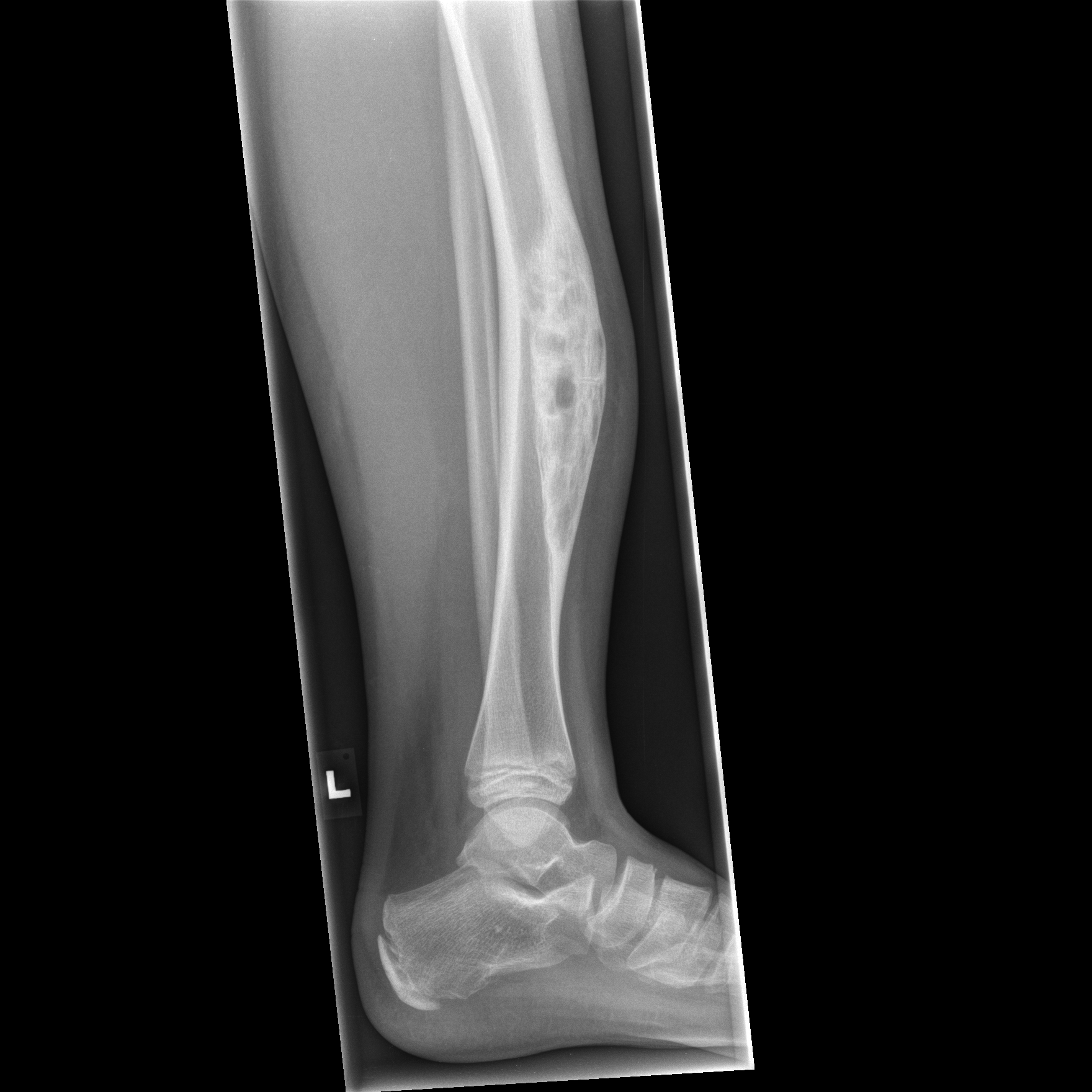

Supplement: Supplementary file 2 — Electronic Supplementary Material [file 330_2024_11115_MOESM2_ESM.zip › Digital Supplementary Material/Radiography/2Radiography.PNG]

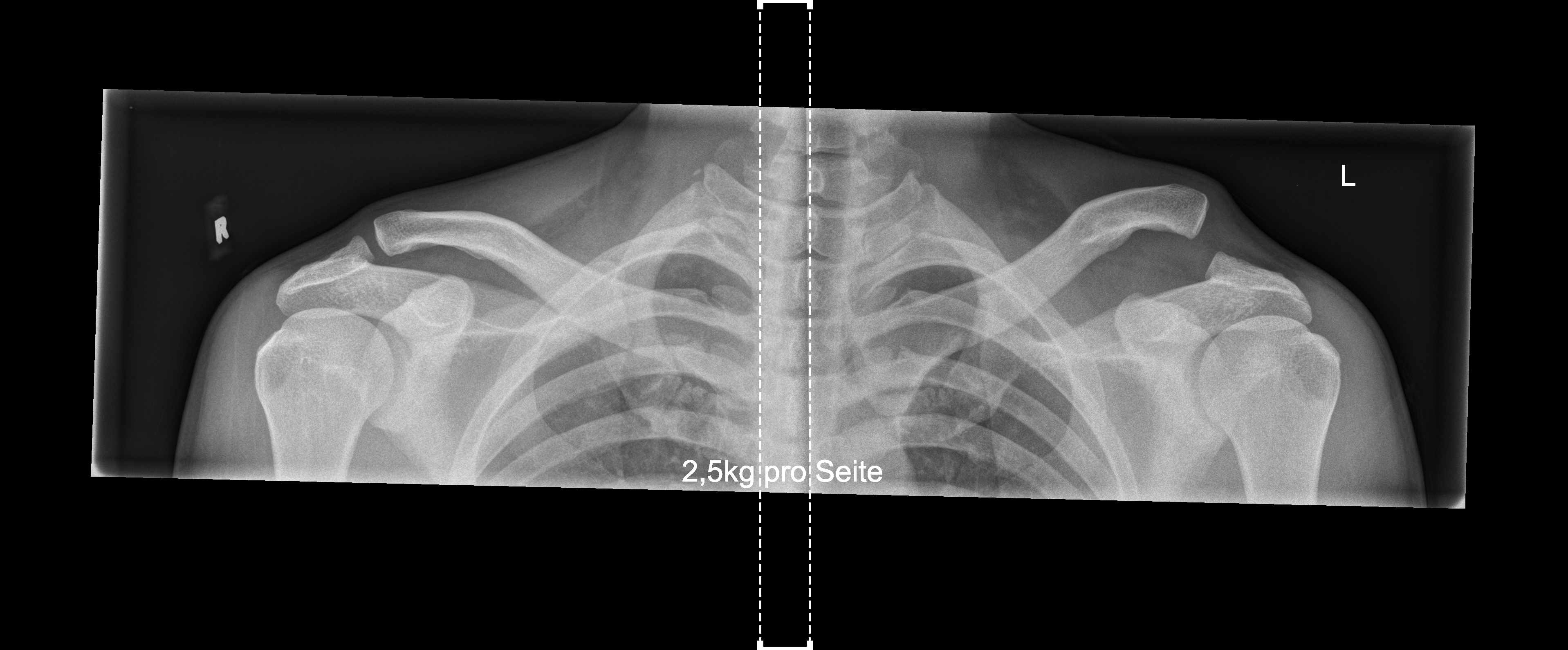

Supplement: Supplementary file 2 — Electronic Supplementary Material [file 330_2024_11115_MOESM2_ESM.zip › Digital Supplementary Material/Radiography/27Radiography.PNG]

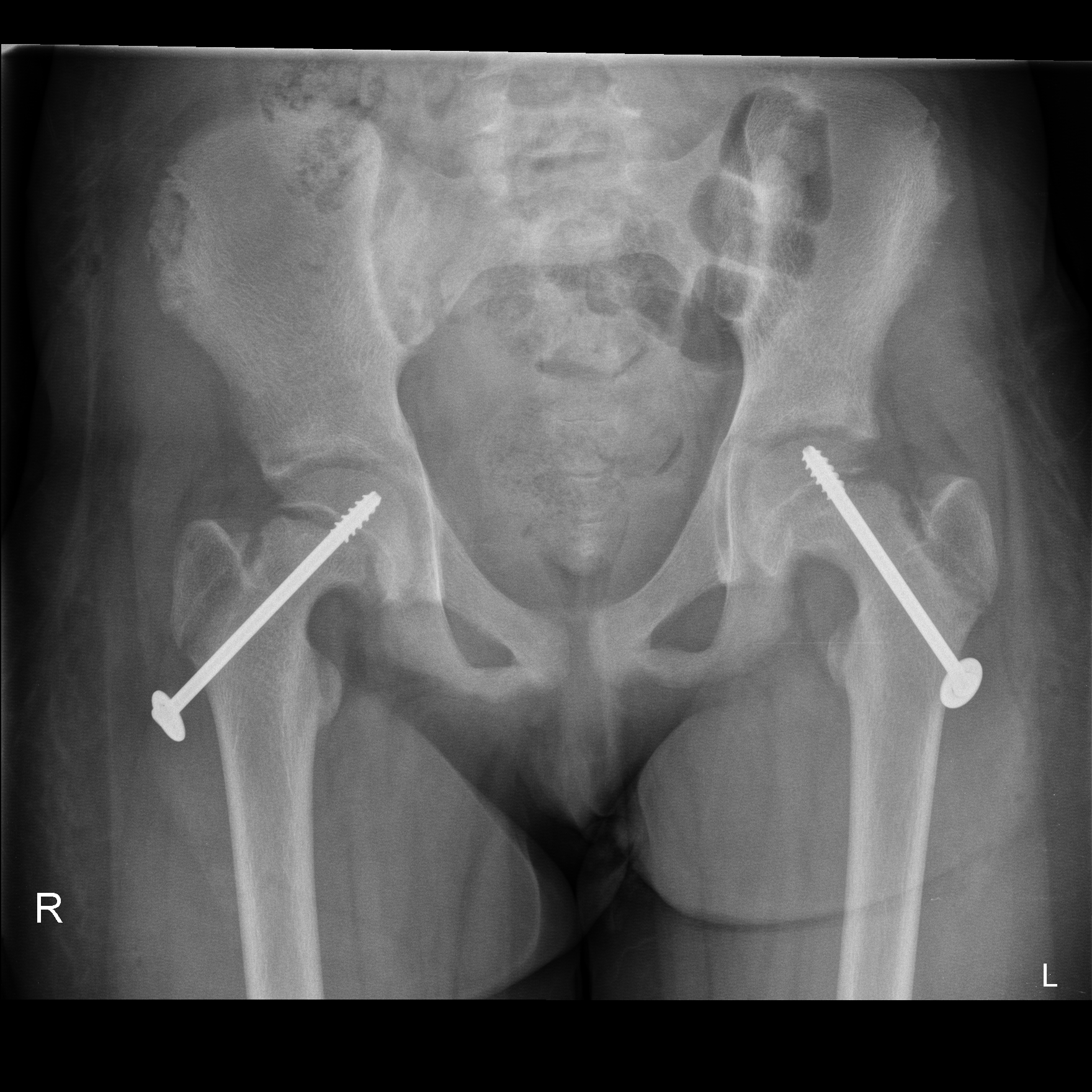

Supplement: Supplementary file 2 — Electronic Supplementary Material [file 330_2024_11115_MOESM2_ESM.zip › Digital Supplementary Material/Radiography/32Radiography.PNG]

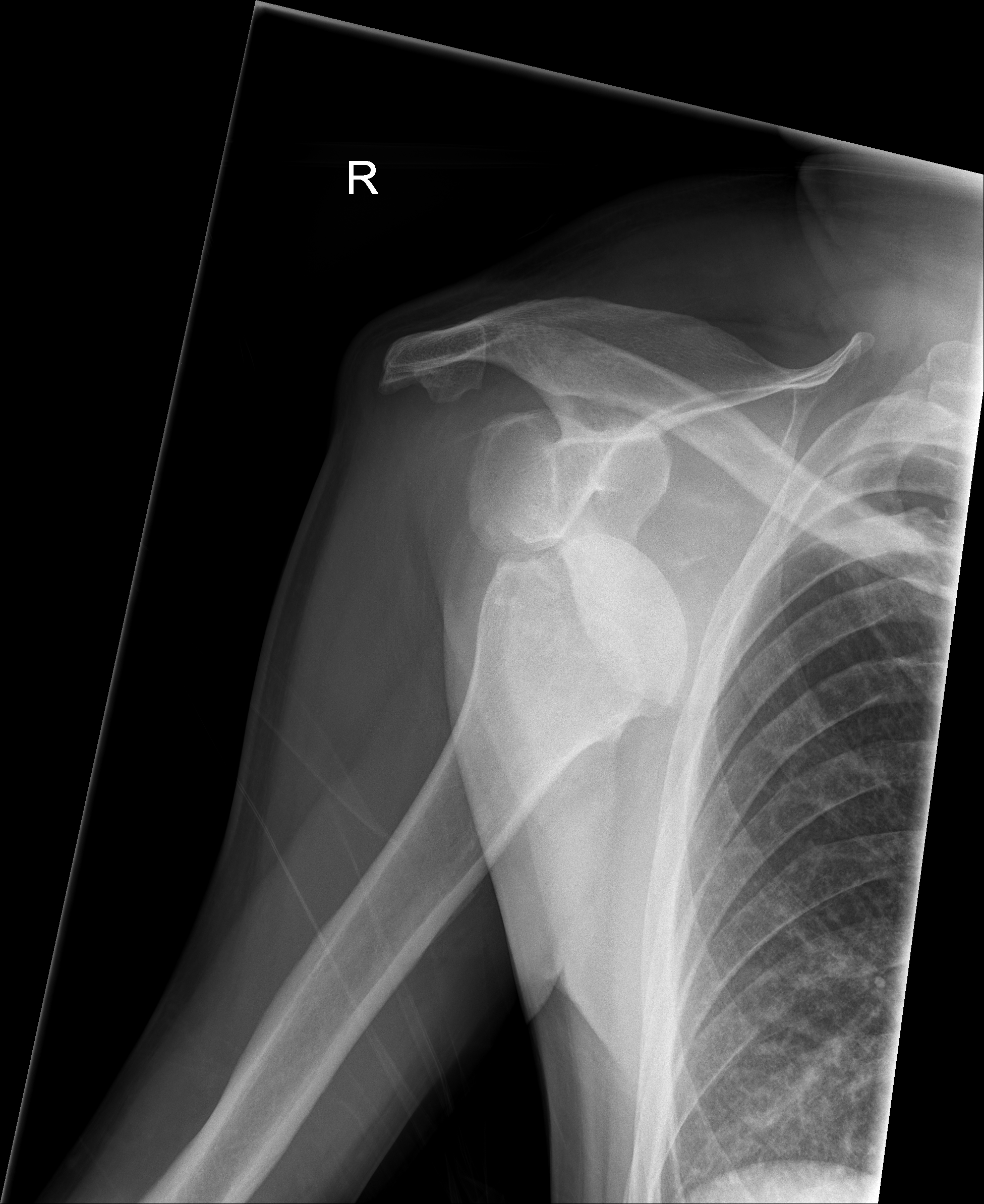

Supplement: Supplementary file 2 — Electronic Supplementary Material [file 330_2024_11115_MOESM2_ESM.zip › Digital Supplementary Material/Radiography/43Radiography.PNG]

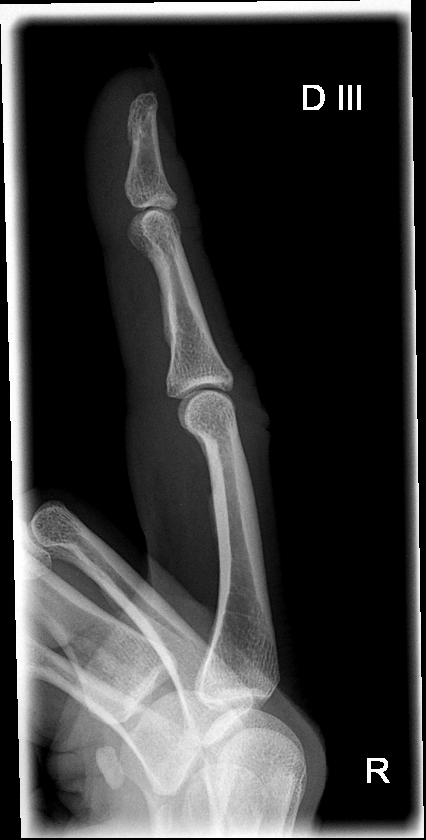

Supplement: Supplementary file 2 — Electronic Supplementary Material [file 330_2024_11115_MOESM2_ESM.zip › Digital Supplementary Material/Radiography/56Radiography.PNG]

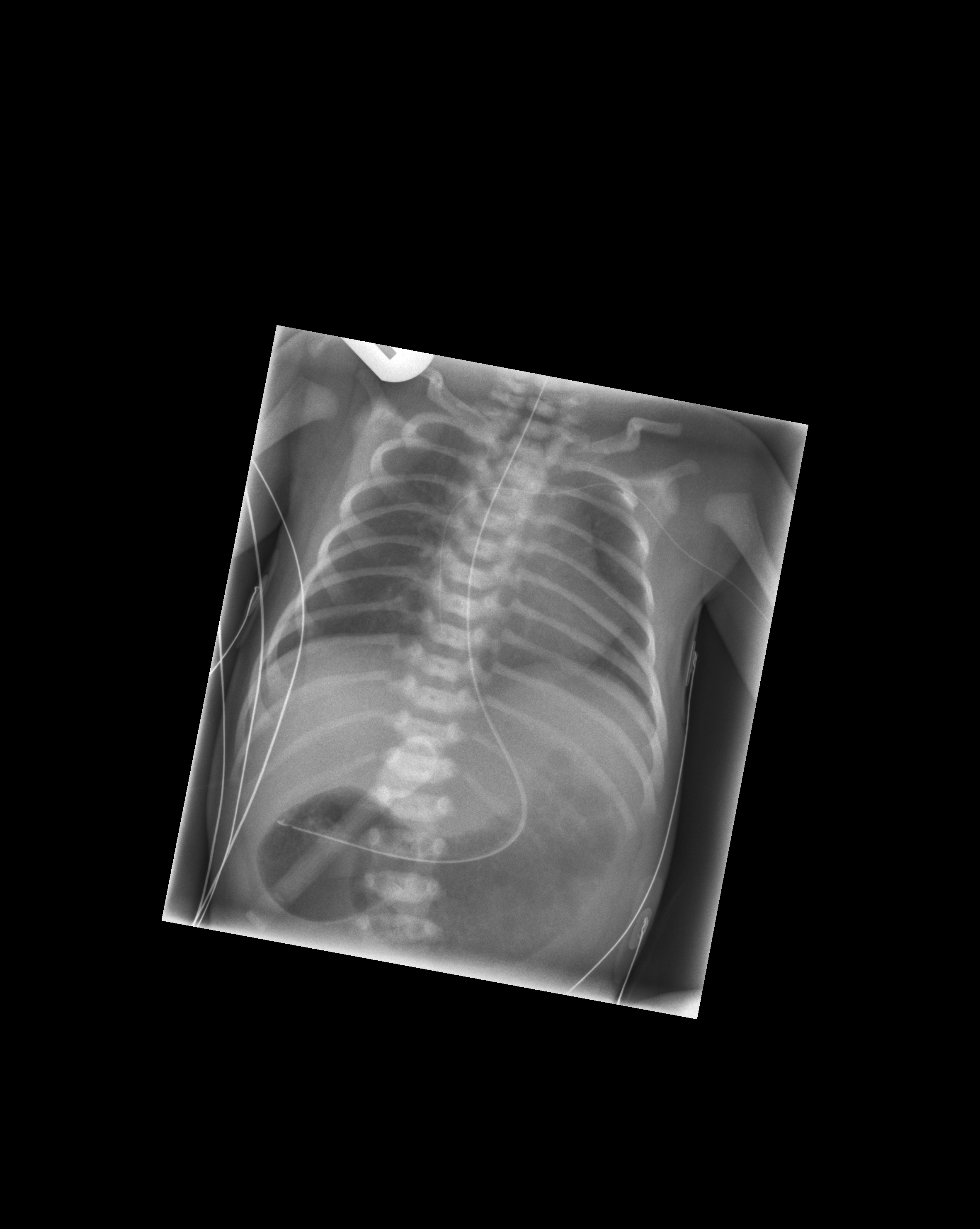

Supplement: Supplementary file 2 — Electronic Supplementary Material [file 330_2024_11115_MOESM2_ESM.zip › Digital Supplementary Material/Radiography/41Radiography.PNG]

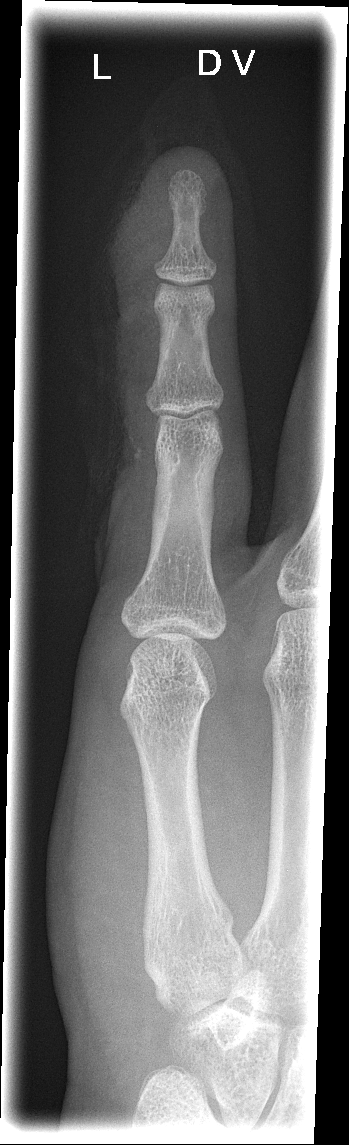

Supplement: Supplementary file 2 — Electronic Supplementary Material [file 330_2024_11115_MOESM2_ESM.zip › Digital Supplementary Material/Radiography/54Radiography.PNG]

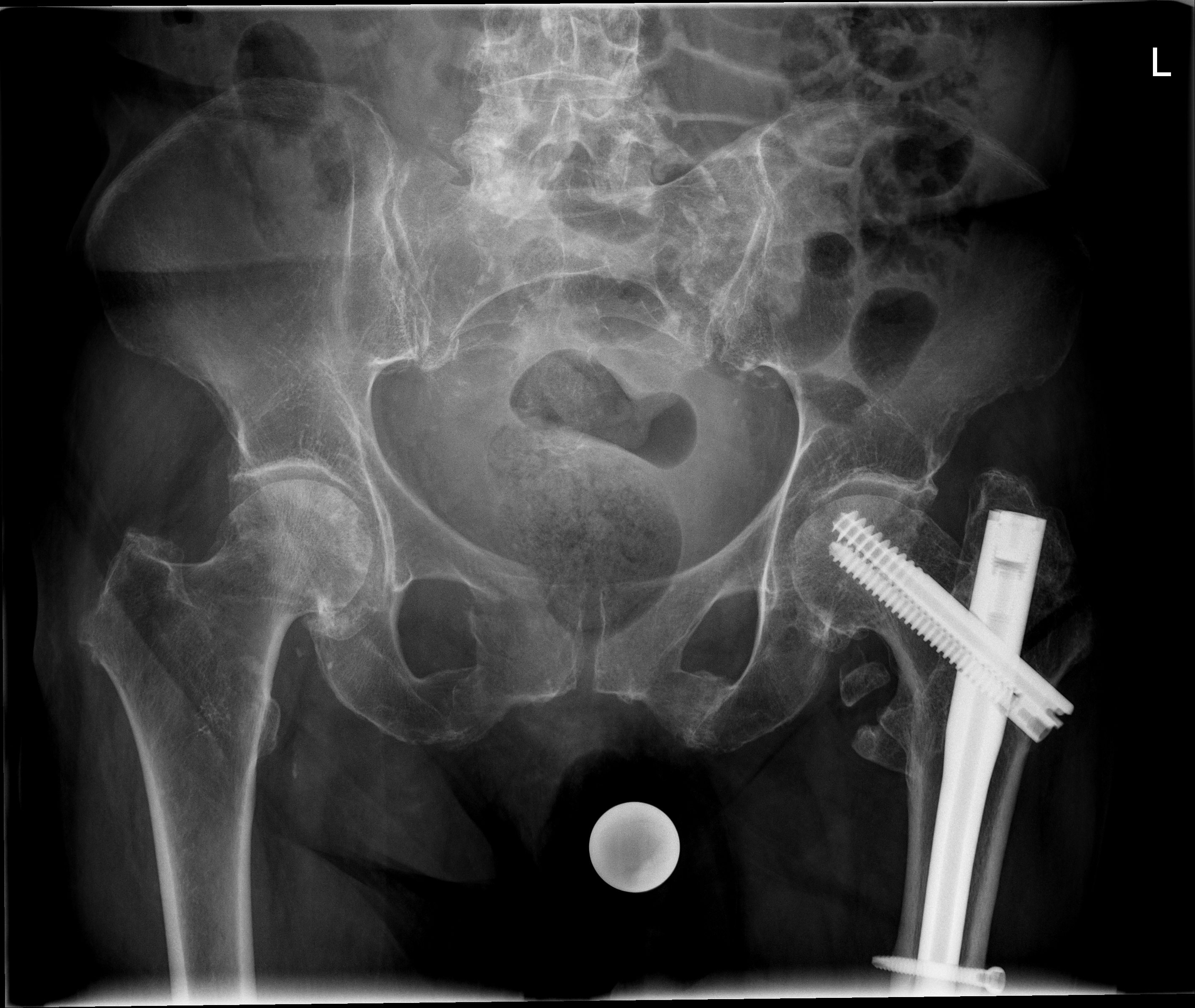

Supplement: Supplementary file 2 — Electronic Supplementary Material [file 330_2024_11115_MOESM2_ESM.zip › Digital Supplementary Material/Radiography/25Radiography.PNG]

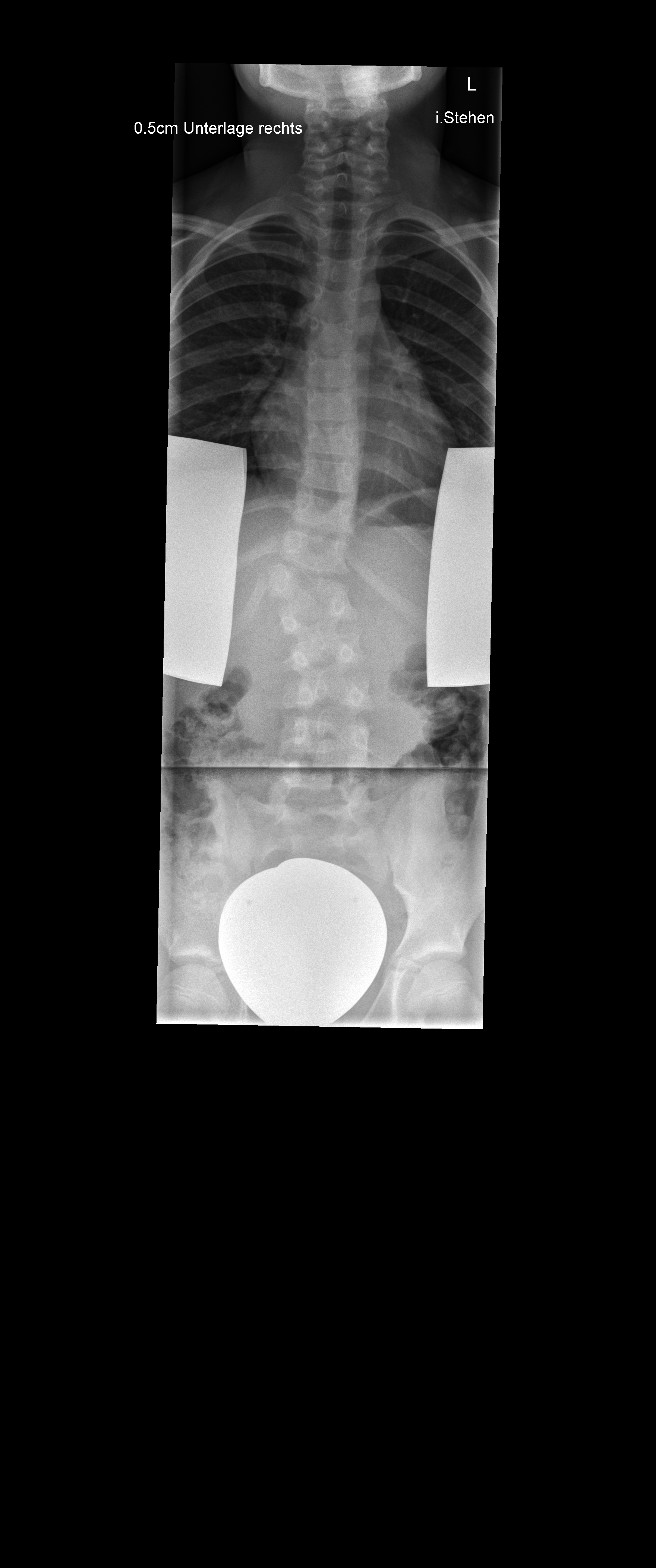

Supplement: Supplementary file 2 — Electronic Supplementary Material [file 330_2024_11115_MOESM2_ESM.zip › Digital Supplementary Material/Radiography/30Radiography.PNG]

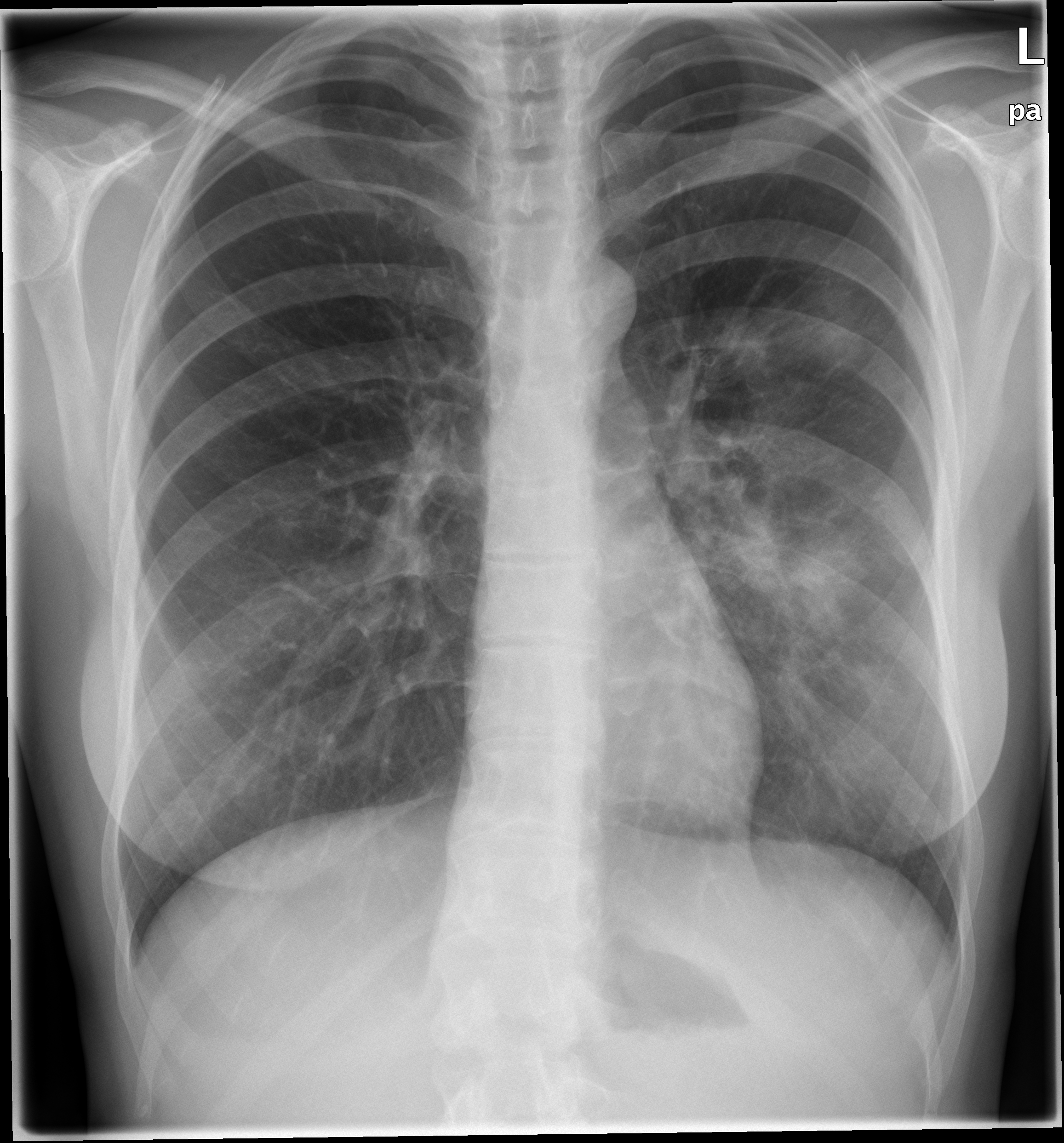

Supplement: Supplementary file 2 — Electronic Supplementary Material [file 330_2024_11115_MOESM2_ESM.zip › Digital Supplementary Material/Radiography/8Radiography.PNG]

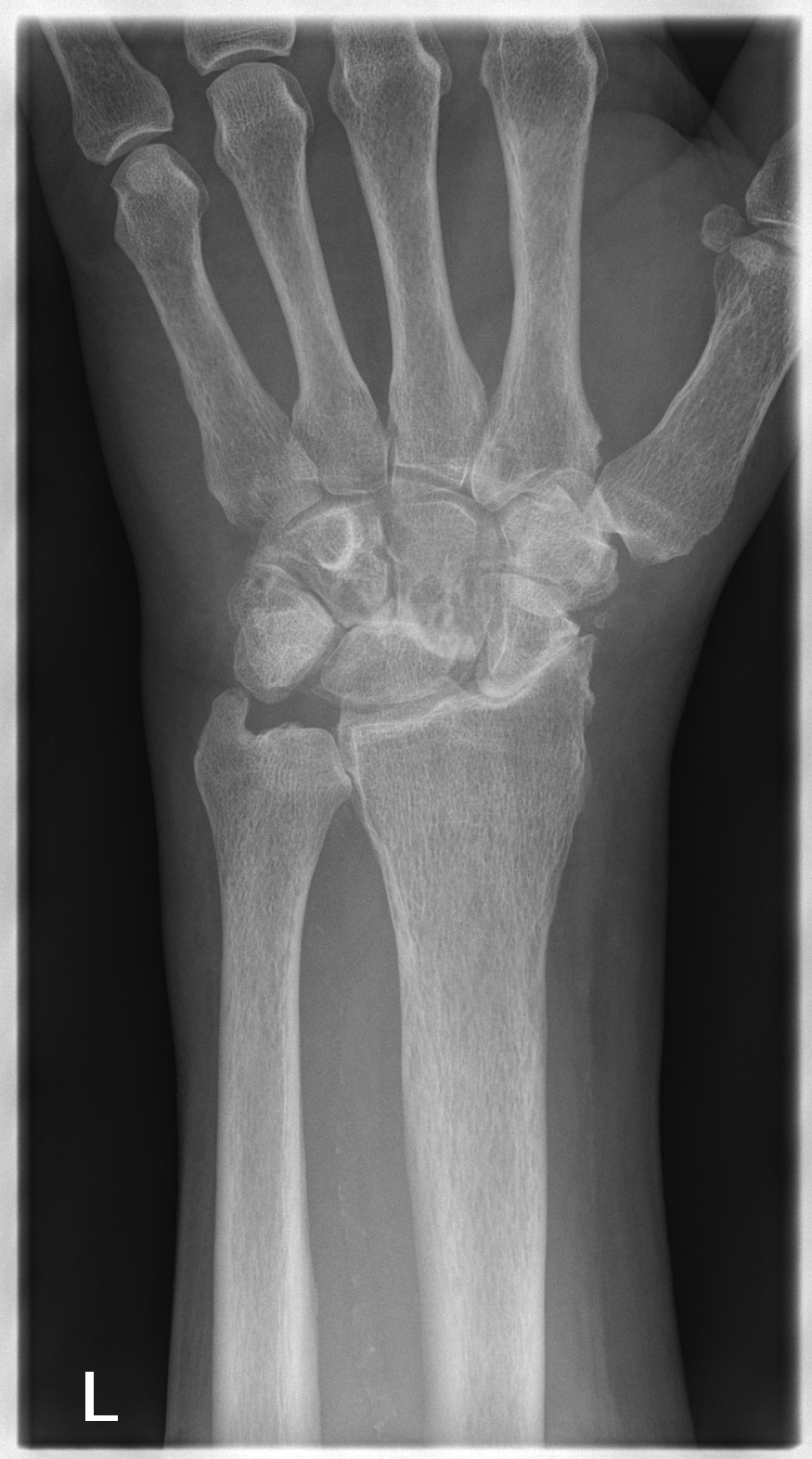

Supplement: Supplementary file 2 — Electronic Supplementary Material [file 330_2024_11115_MOESM2_ESM.zip › Digital Supplementary Material/Radiography/38Radiography.PNG]

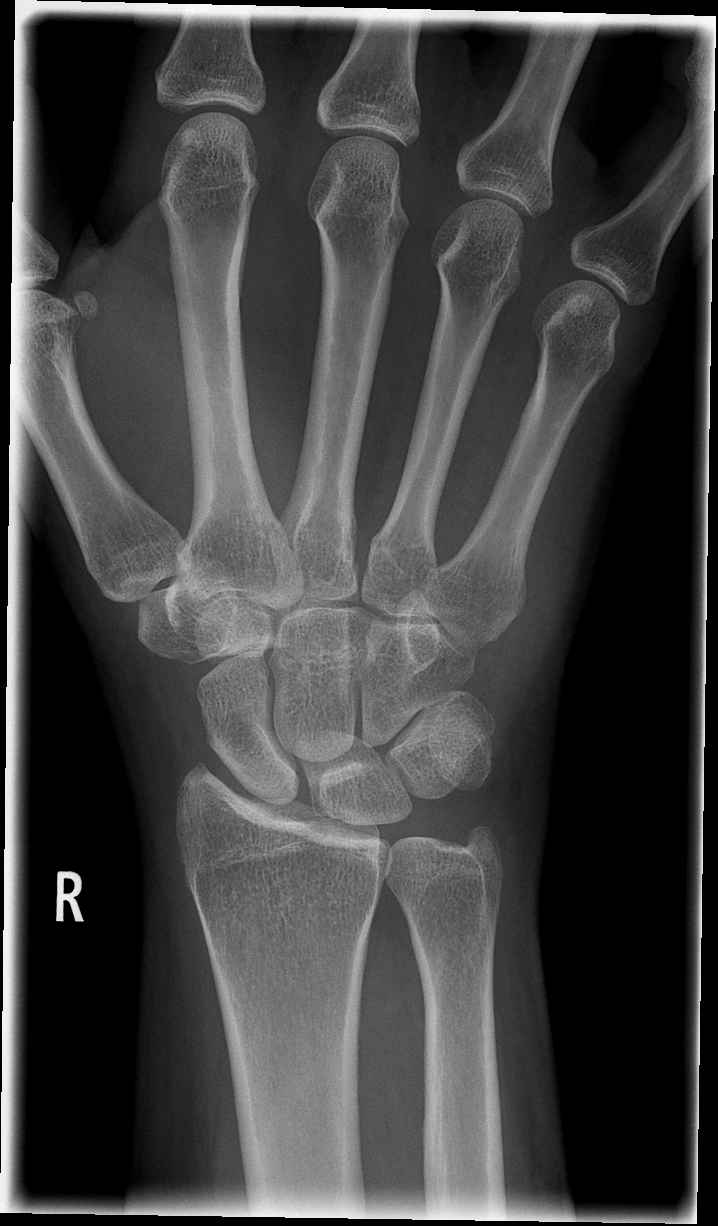

Supplement: Supplementary file 2 — Electronic Supplementary Material [file 330_2024_11115_MOESM2_ESM.zip › Digital Supplementary Material/Radiography/49Radiography.PNG]

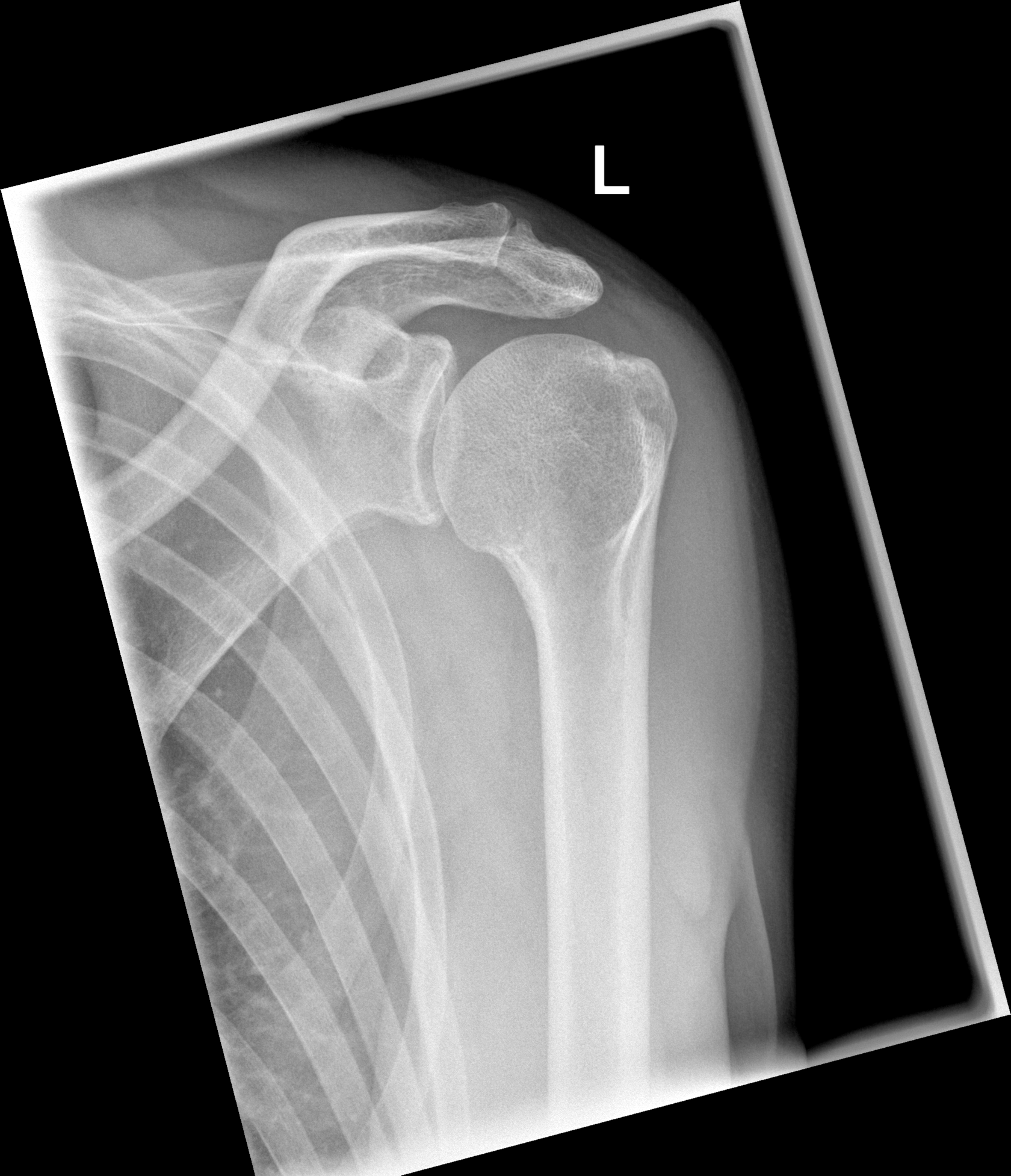

Supplement: Supplementary file 2 — Electronic Supplementary Material [file 330_2024_11115_MOESM2_ESM.zip › Digital Supplementary Material/Radiography/53Radiography.PNG]

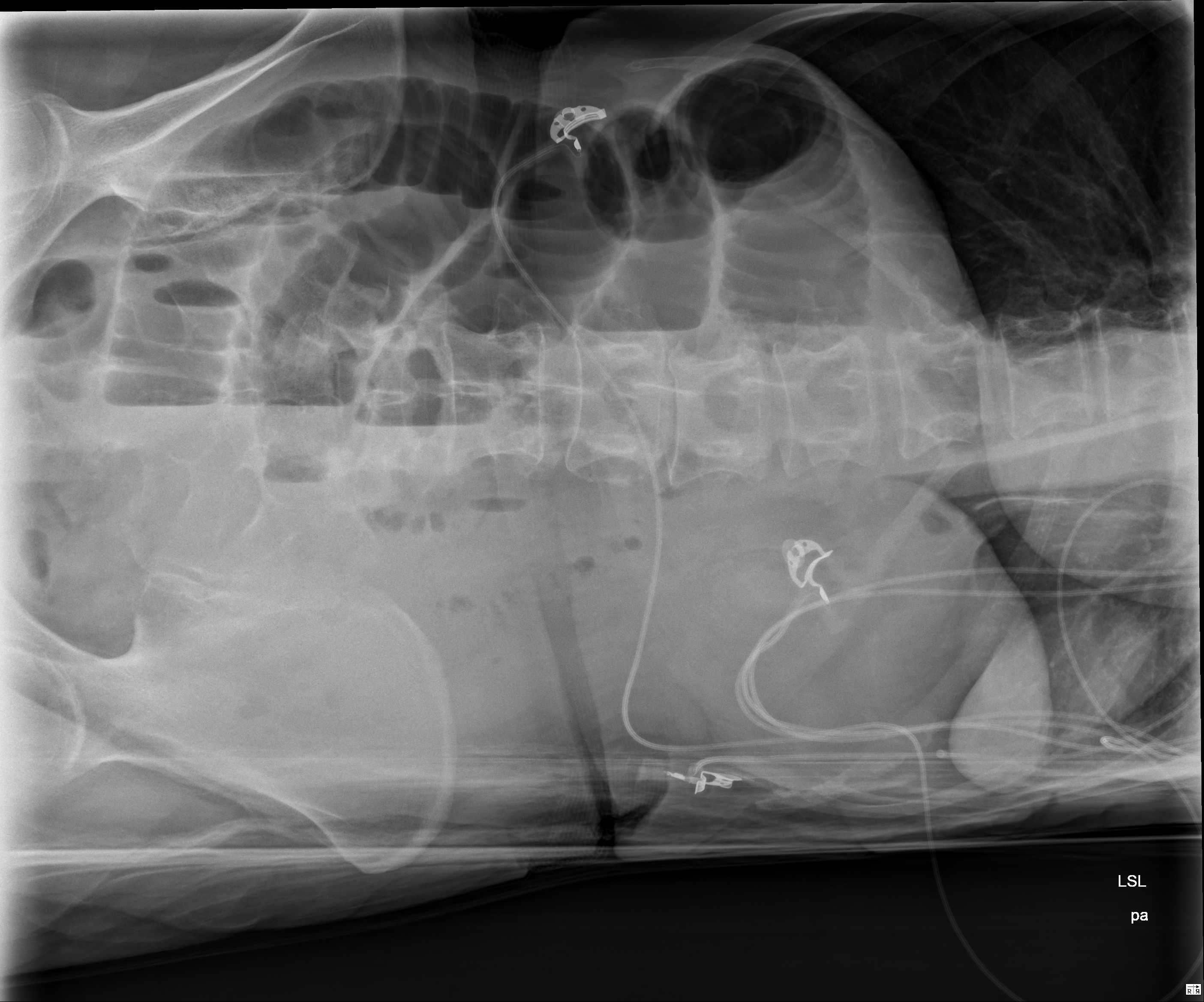

Supplement: Supplementary file 2 — Electronic Supplementary Material [file 330_2024_11115_MOESM2_ESM.zip › Digital Supplementary Material/Radiography/46Radiography.PNG]

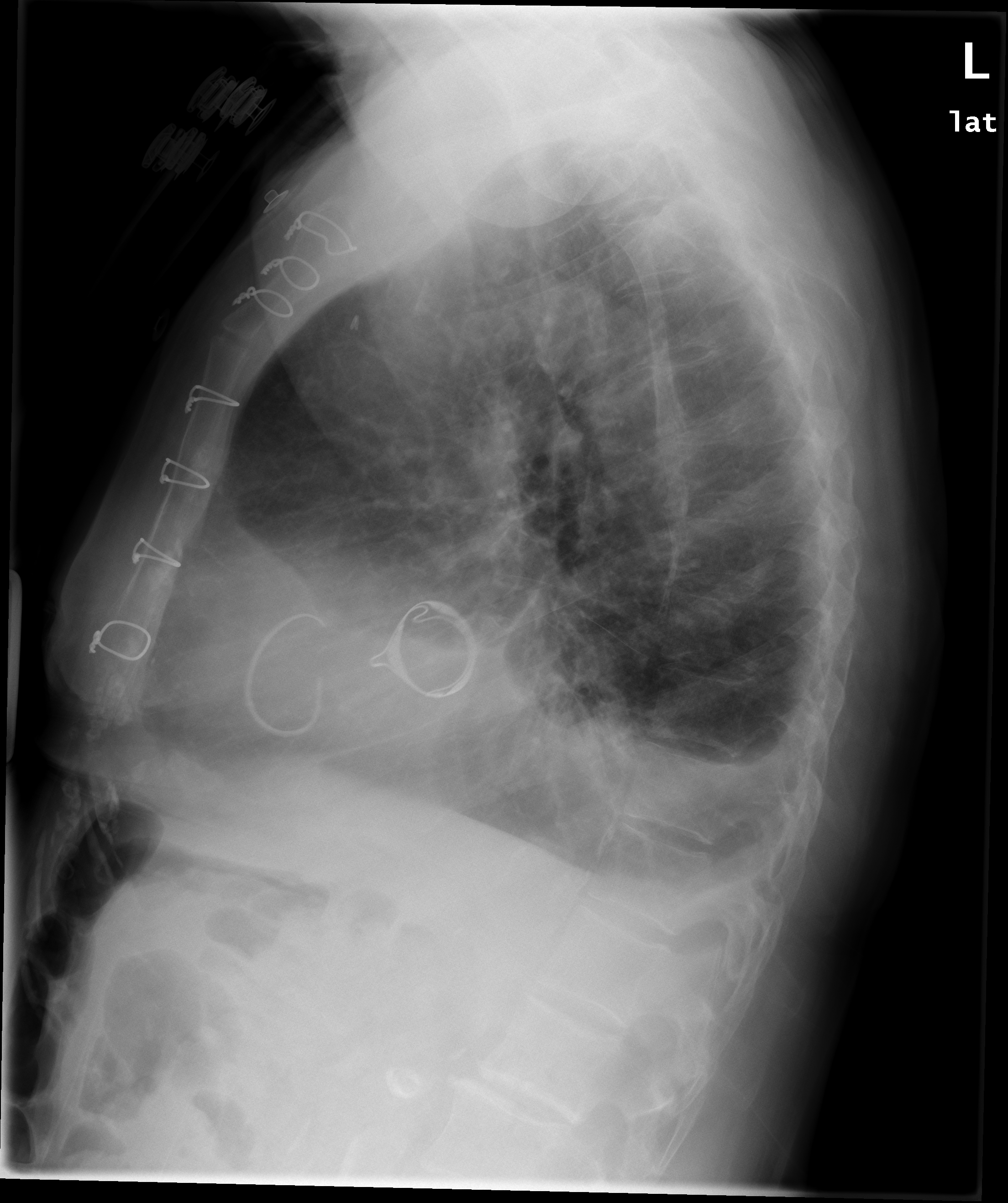

Supplement: Supplementary file 2 — Electronic Supplementary Material [file 330_2024_11115_MOESM2_ESM.zip › Digital Supplementary Material/Radiography/7Radiography.PNG]

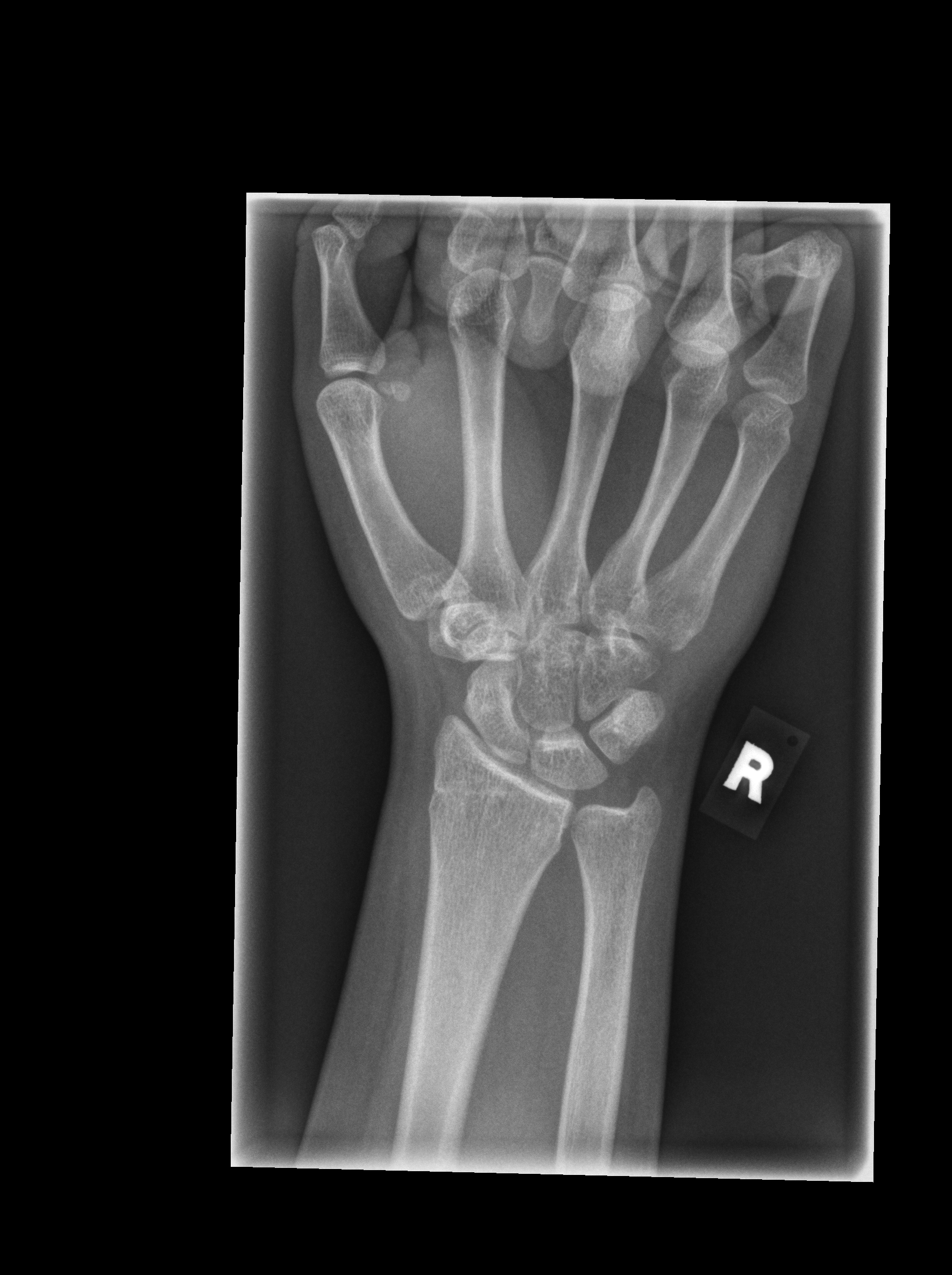

Supplement: Supplementary file 2 — Electronic Supplementary Material [file 330_2024_11115_MOESM2_ESM.zip › Digital Supplementary Material/Radiography/37Radiography.PNG]

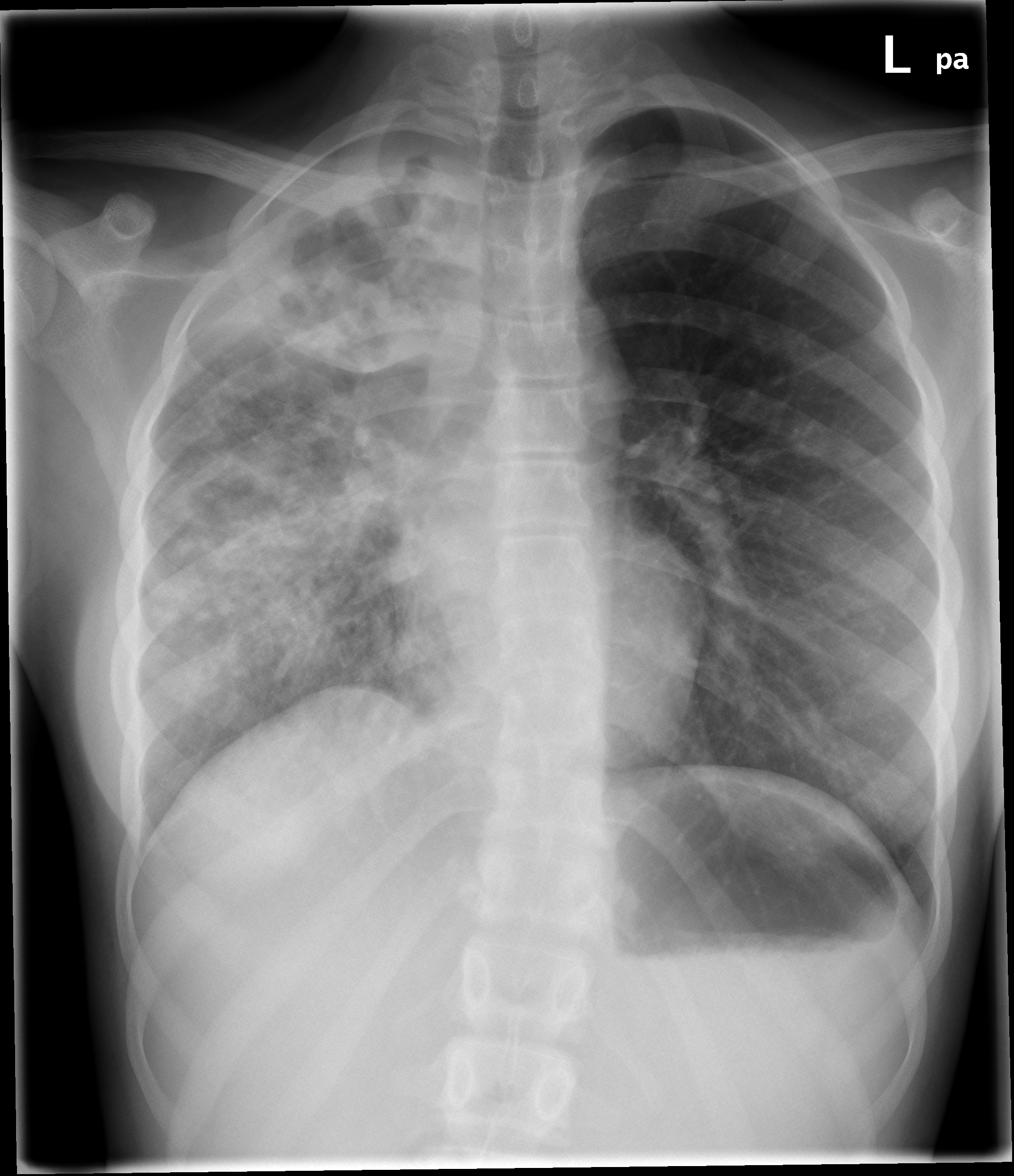

Supplement: Supplementary file 2 — Electronic Supplementary Material [file 330_2024_11115_MOESM2_ESM.zip › Digital Supplementary Material/Radiography/22Radiography.PNG]

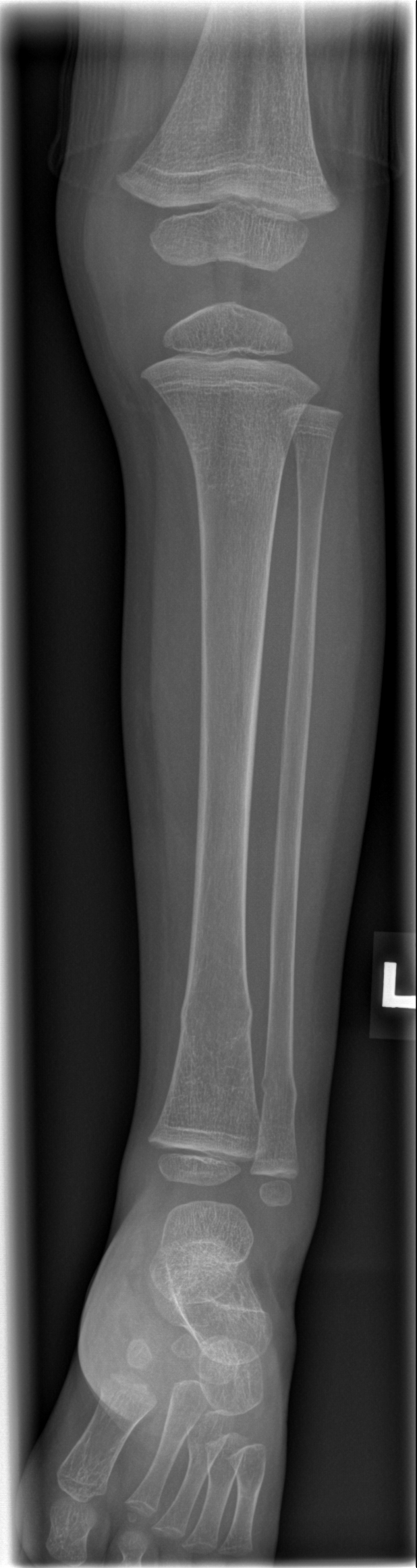

Supplement: Supplementary file 2 — Electronic Supplementary Material [file 330_2024_11115_MOESM2_ESM.zip › Digital Supplementary Material/Radiography/47Radiography.PNG]

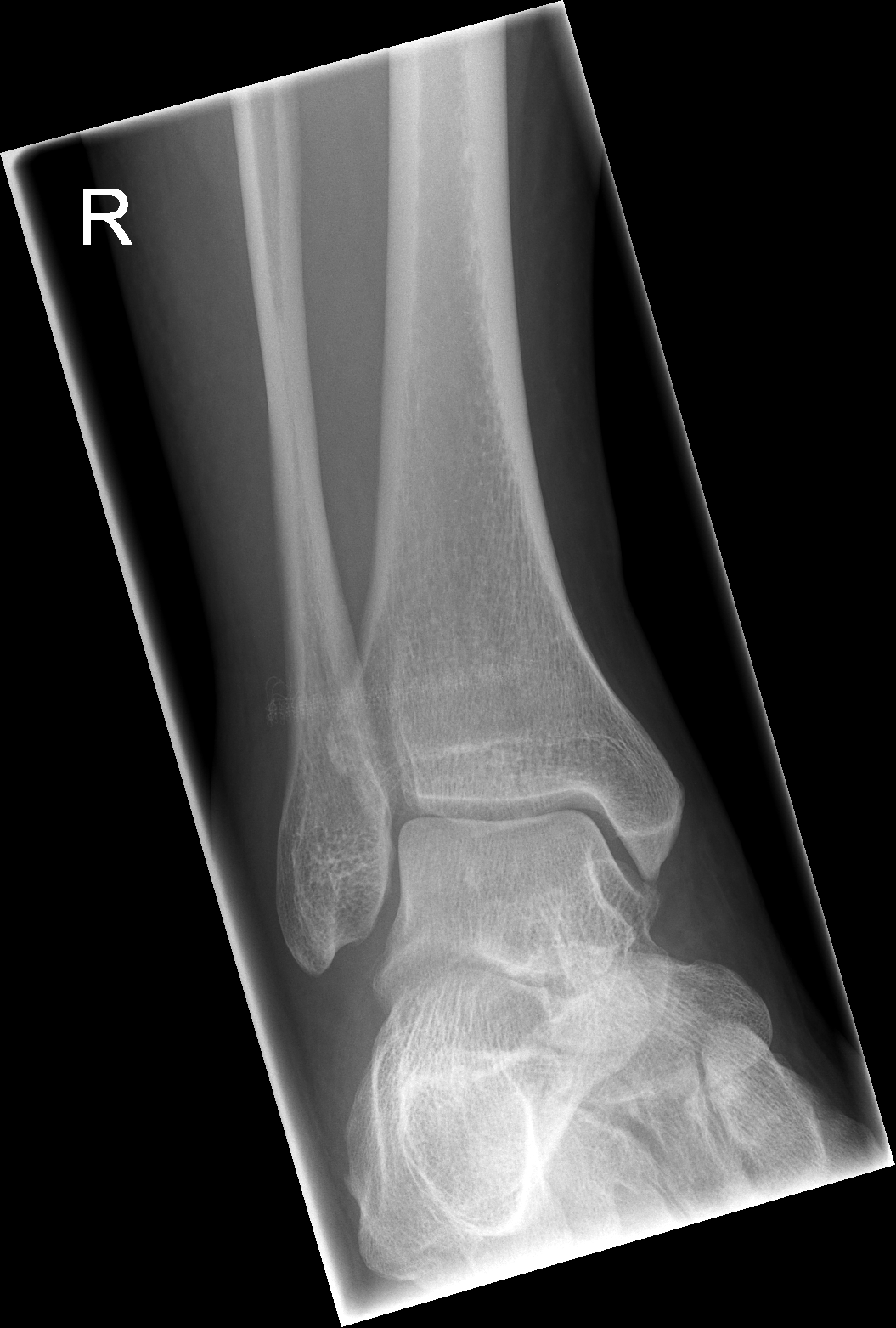

Supplement: Supplementary file 2 — Electronic Supplementary Material [file 330_2024_11115_MOESM2_ESM.zip › Digital Supplementary Material/Radiography/52Radiography.PNG]

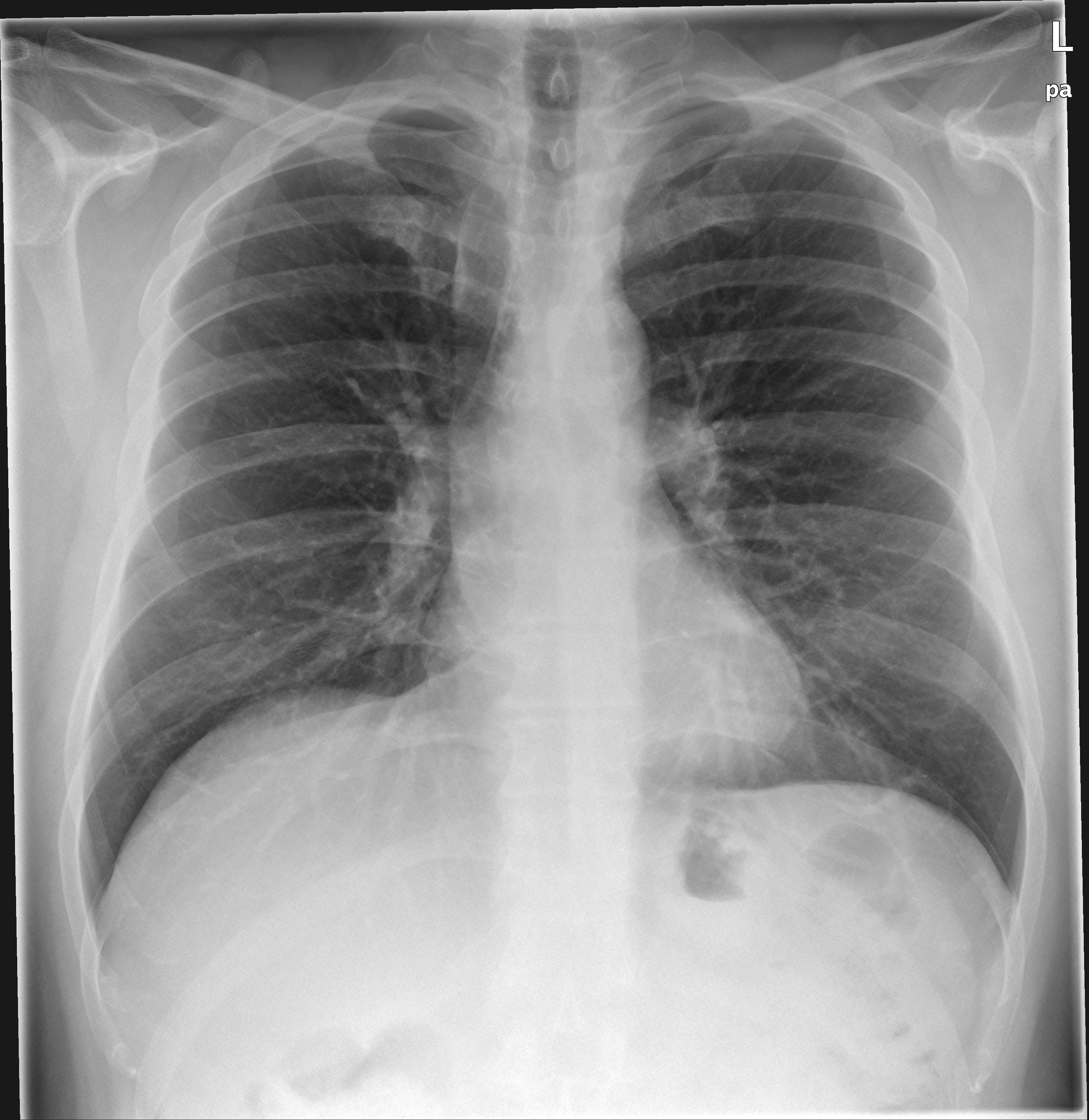

Supplement: Supplementary file 2 — Electronic Supplementary Material [file 330_2024_11115_MOESM2_ESM.zip › Digital Supplementary Material/Radiography/23Radiography.PNG]

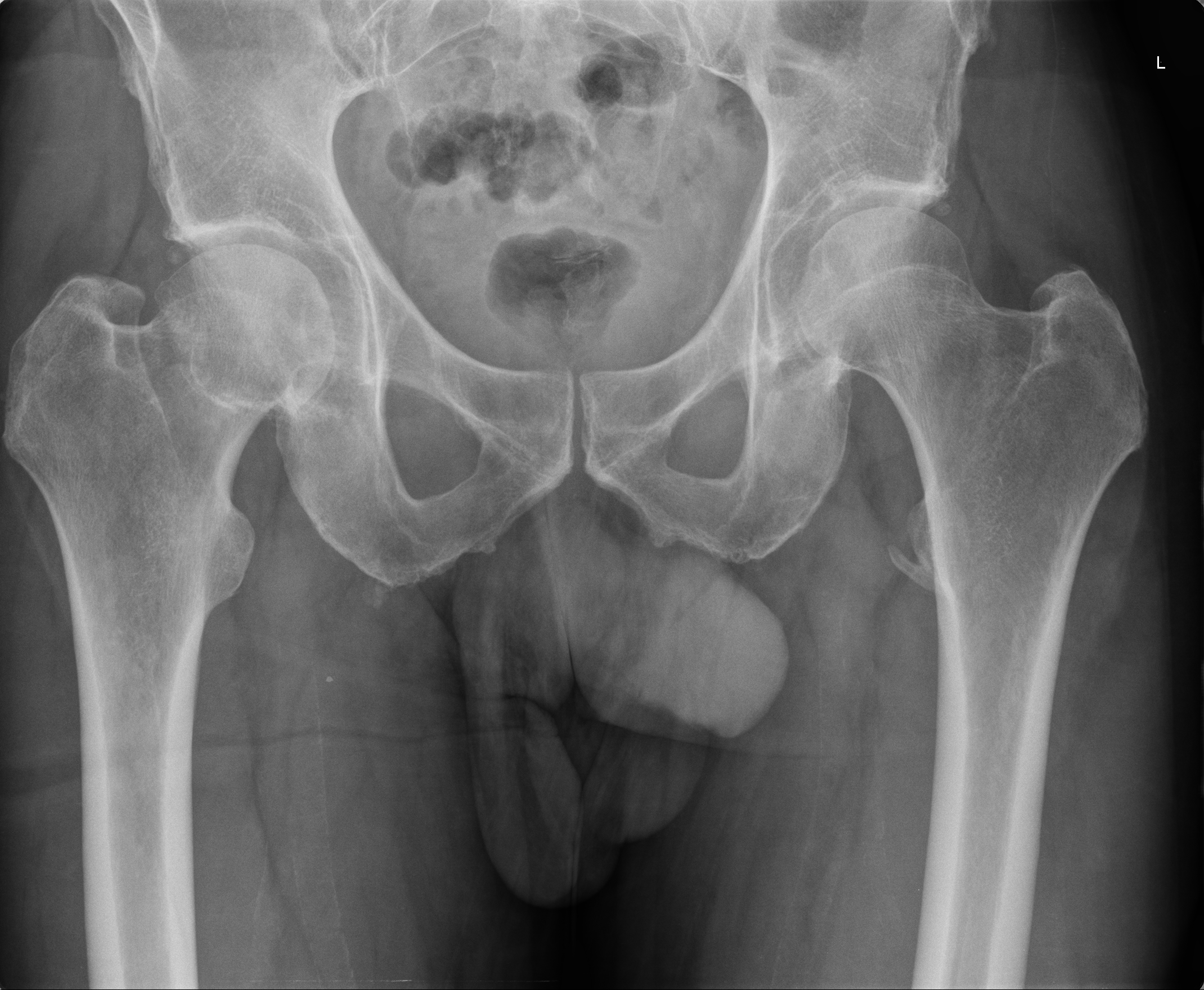

Supplement: Supplementary file 2 — Electronic Supplementary Material [file 330_2024_11115_MOESM2_ESM.zip › Digital Supplementary Material/Radiography/36Radiography.PNG]

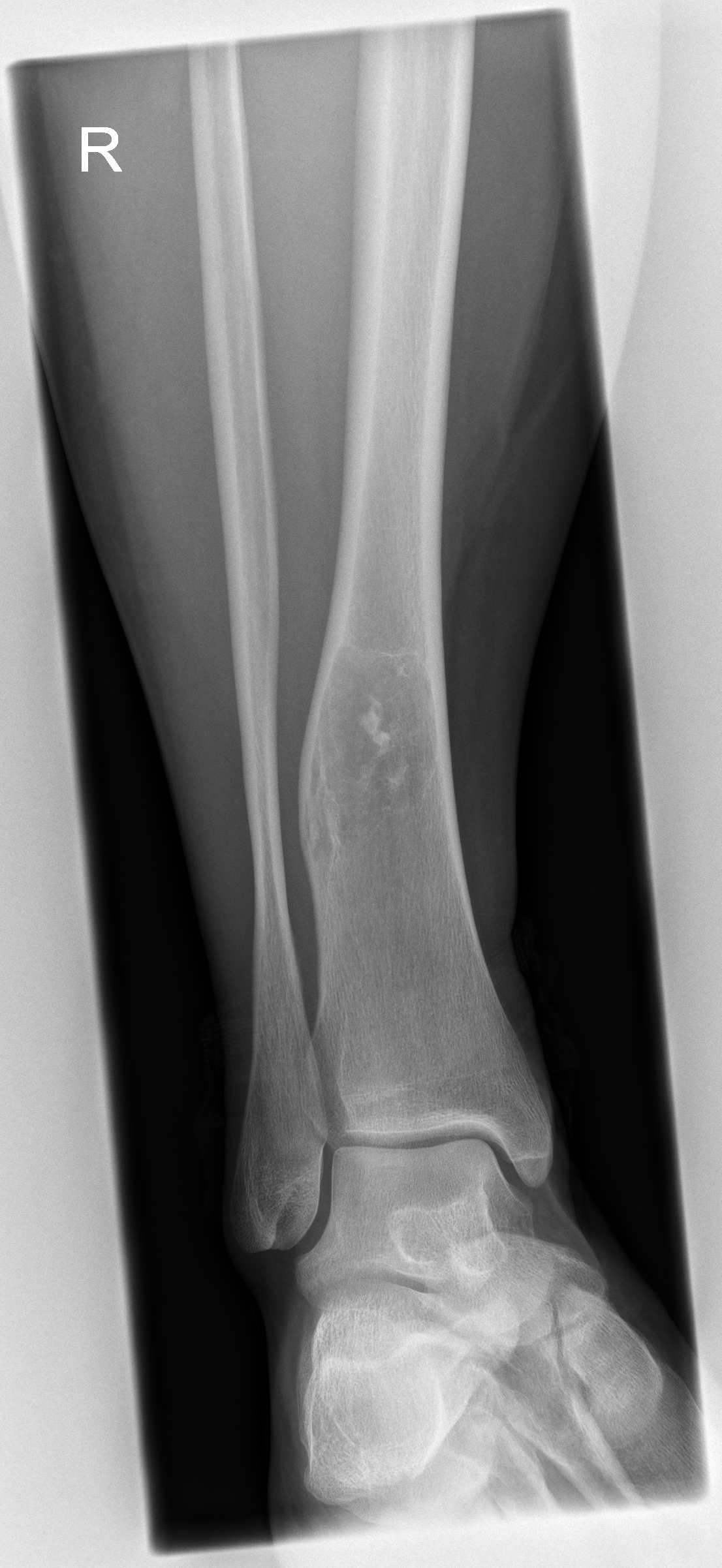

Supplement: Supplementary file 2 — Electronic Supplementary Material [file 330_2024_11115_MOESM2_ESM.zip › Digital Supplementary Material/Radiography/6Radiography.PNG]

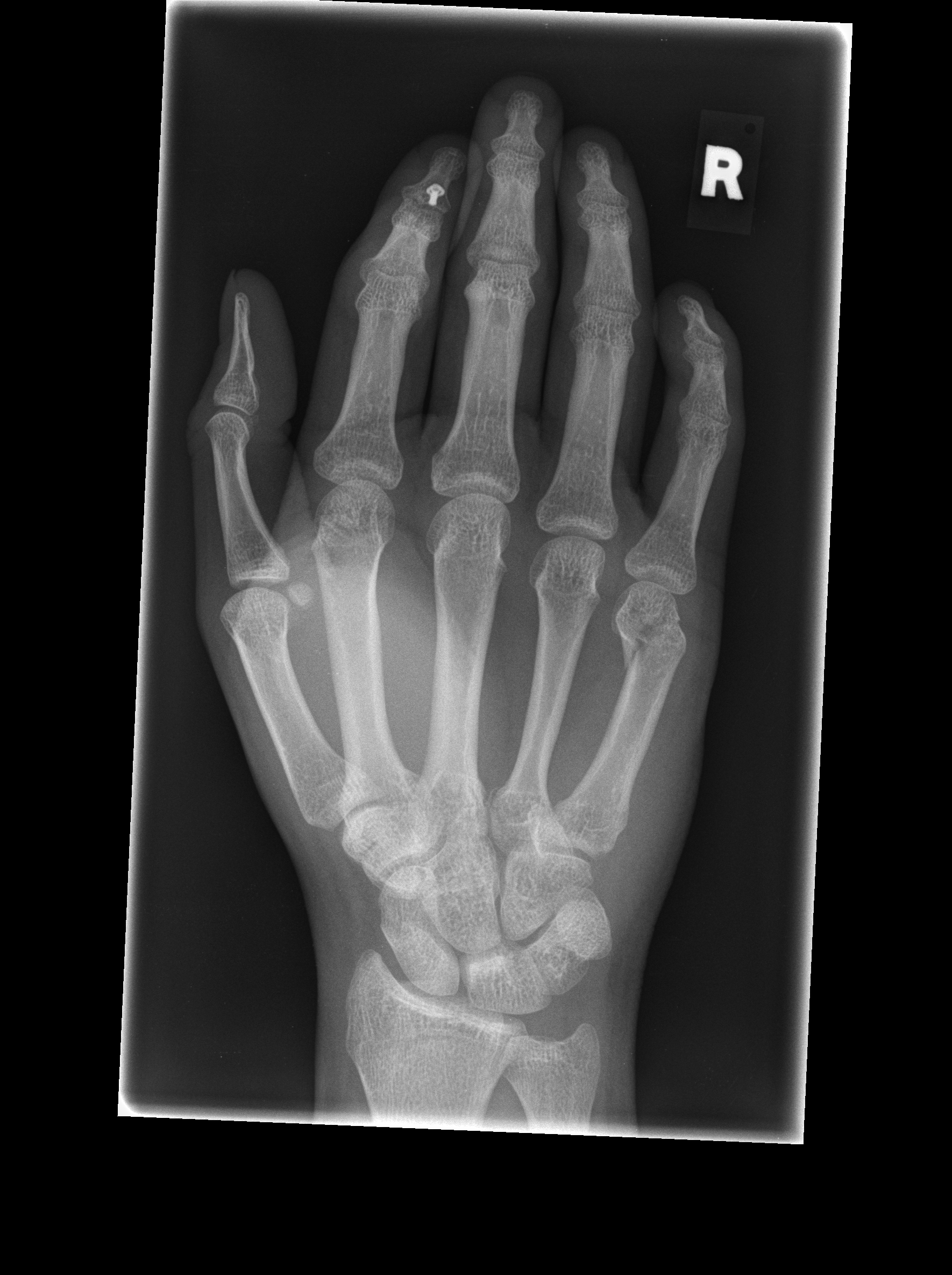

Supplement: Supplementary file 2 — Electronic Supplementary Material [file 330_2024_11115_MOESM2_ESM.zip › Digital Supplementary Material/Radiography/39Radiography.PNG]

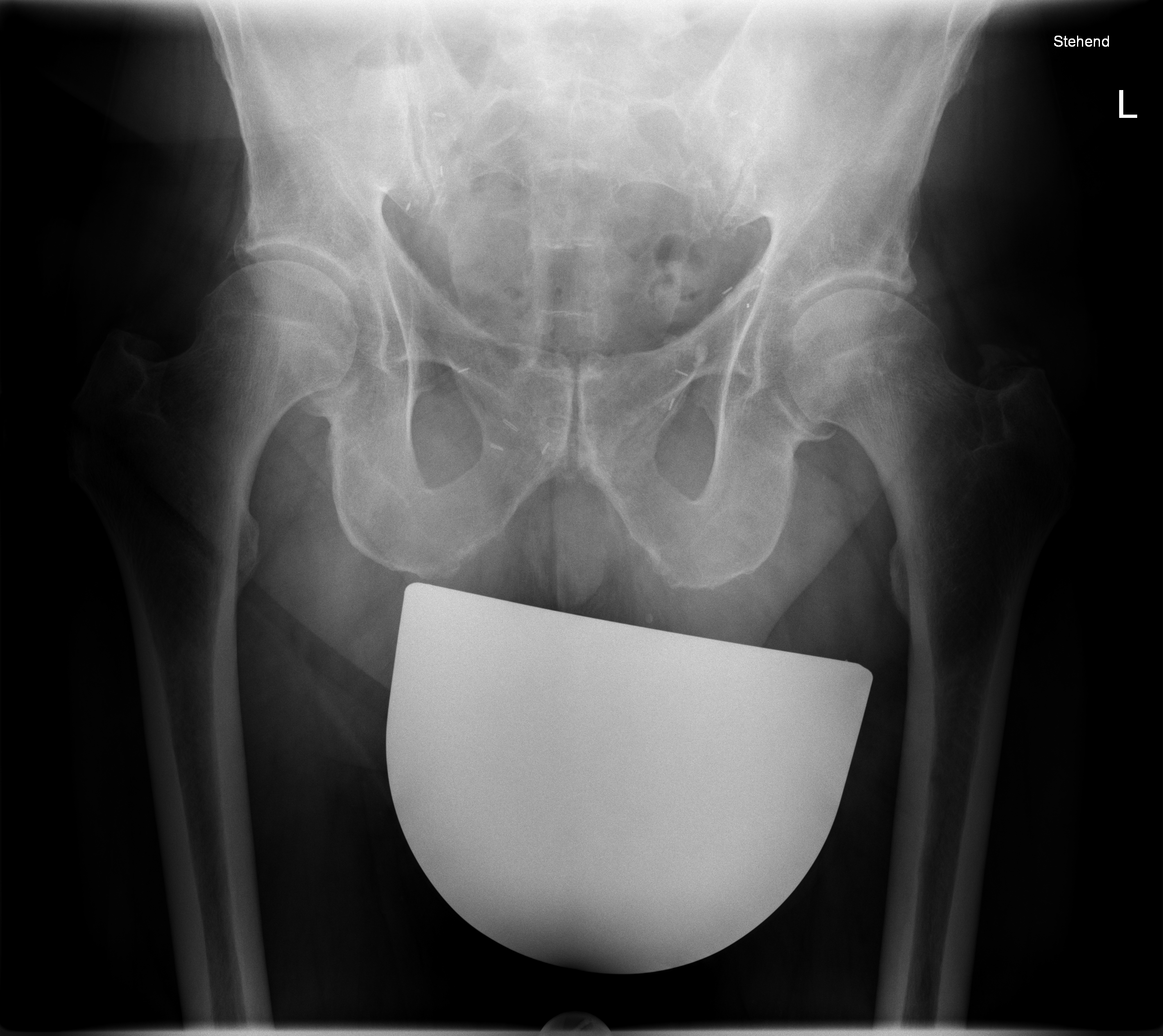

Supplement: Supplementary file 2 — Electronic Supplementary Material [file 330_2024_11115_MOESM2_ESM.zip › Digital Supplementary Material/Radiography/9Radiography.PNG]

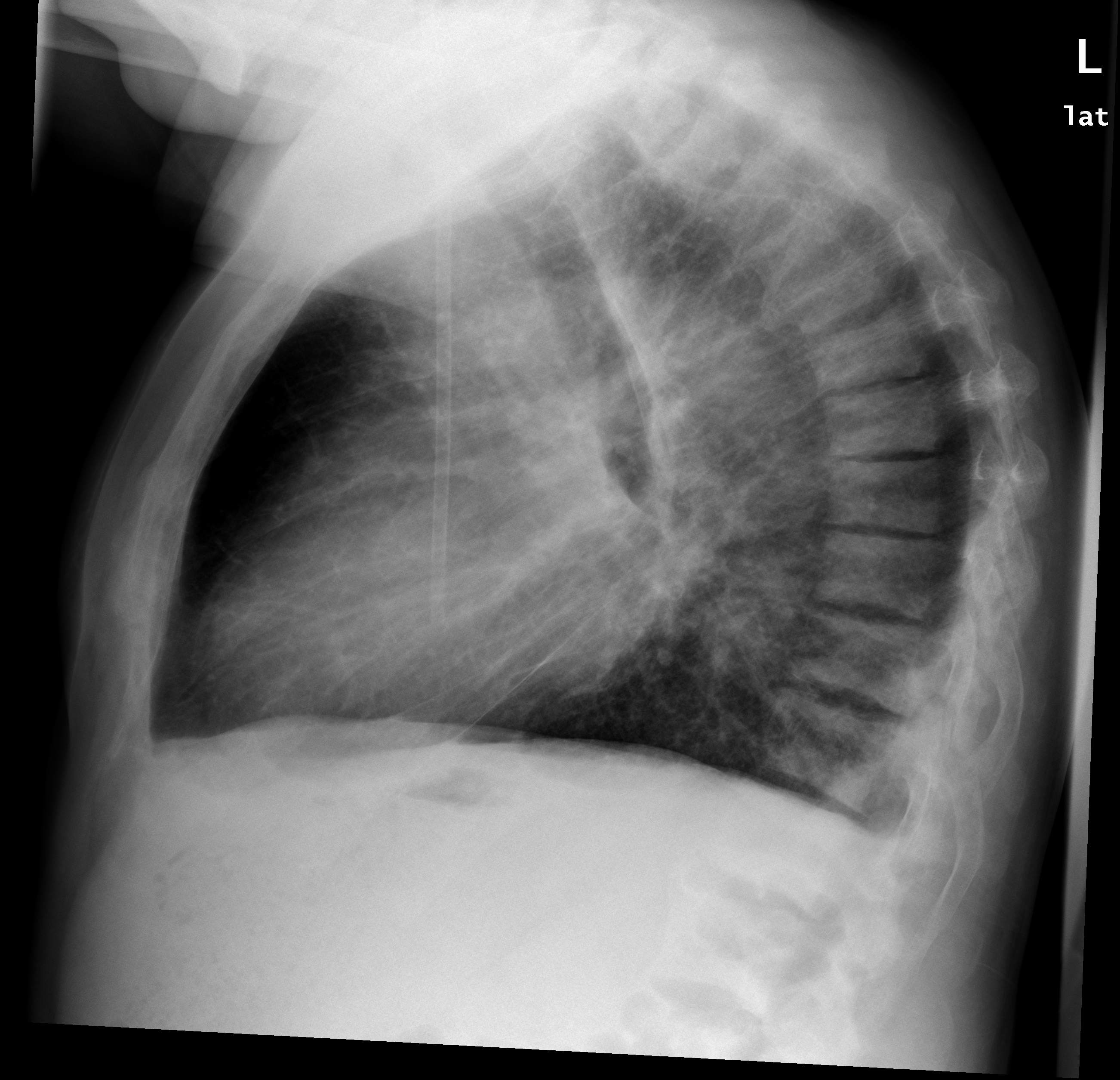

Supplement: Supplementary file 2 — Electronic Supplementary Material [file 330_2024_11115_MOESM2_ESM.zip › Digital Supplementary Material/Radiography/48Radiography.PNG]
