# Supplementary figures and images for: Revolution or risk?—Assessing the potential and challenges of GPT-4V in radiologic image interpretation (part 1 of 2)
Source: Eur Radiol. 2024 Oct 18;35(3):1111–21. doi: 10.1007/s00330-024-11115-6 (PMC11836096; doi:10.1007/s00330-024-11115-6)

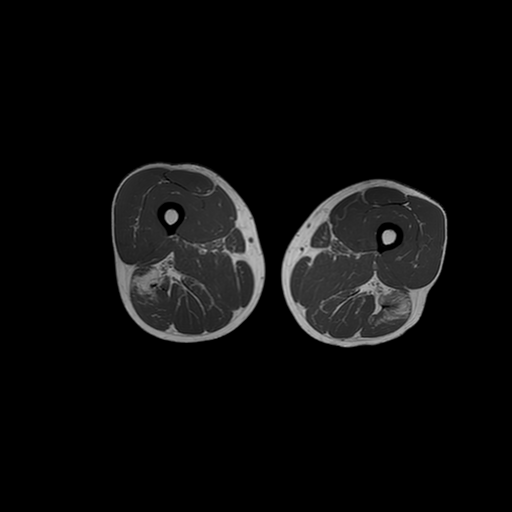

Supplement: Supplementary file 2 — Electronic Supplementary Material [file 330_2024_11115_MOESM2_ESM.zip › Digital Supplementary Material/Magnetic Resonance Imaging/33Magnetic Resonance Imaging.PNG]

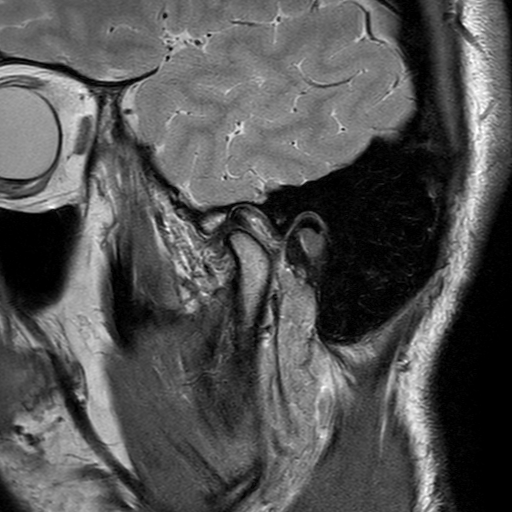

Supplement: Supplementary file 2 — Electronic Supplementary Material [file 330_2024_11115_MOESM2_ESM.zip › Digital Supplementary Material/Magnetic Resonance Imaging/16Magnetic Resonance Imaging.PNG]

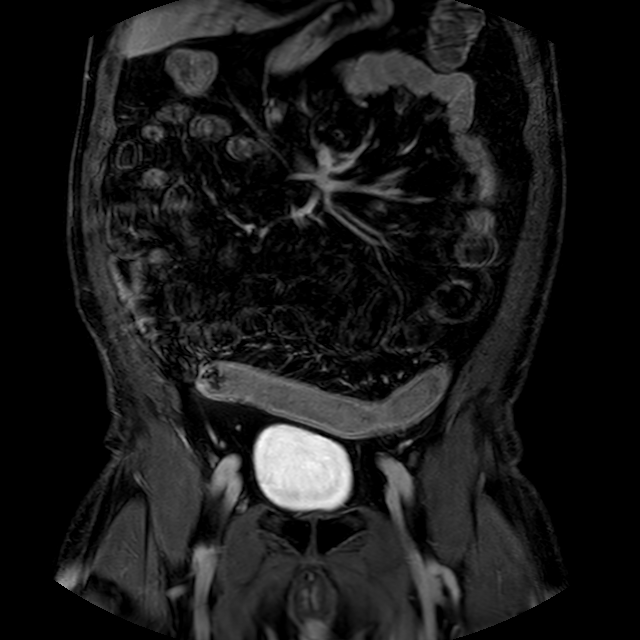

Supplement: Supplementary file 2 — Electronic Supplementary Material [file 330_2024_11115_MOESM2_ESM.zip › Digital Supplementary Material/Magnetic Resonance Imaging/53Magnetic Resonance Imaging.PNG]

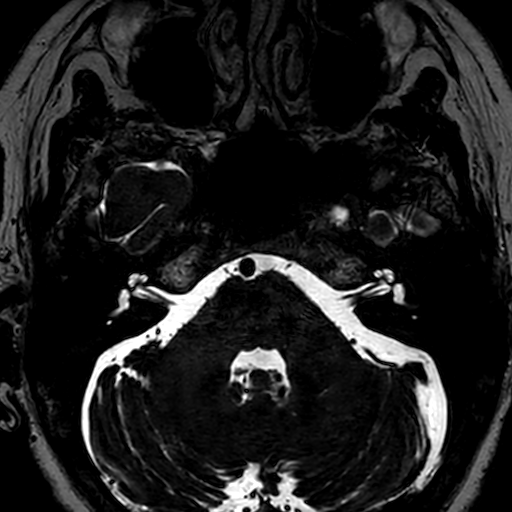

Supplement: Supplementary file 2 — Electronic Supplementary Material [file 330_2024_11115_MOESM2_ESM.zip › Digital Supplementary Material/Magnetic Resonance Imaging/22Magnetic Resonance Imaging.PNG]

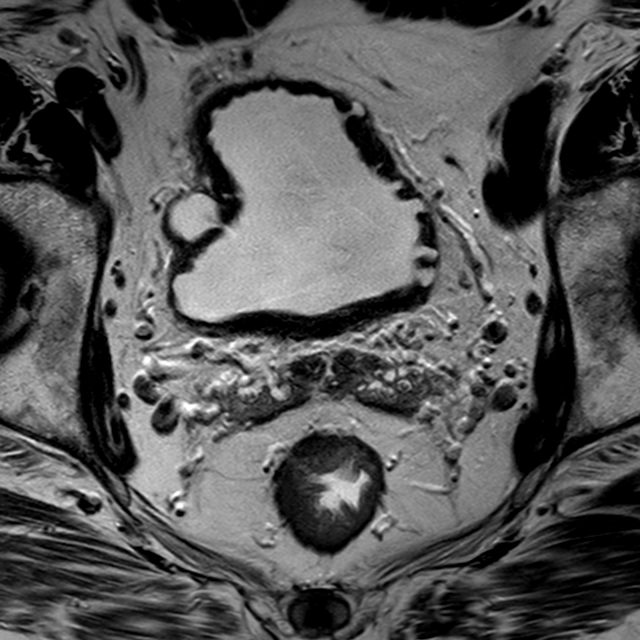

Supplement: Supplementary file 2 — Electronic Supplementary Material [file 330_2024_11115_MOESM2_ESM.zip › Digital Supplementary Material/Magnetic Resonance Imaging/42Magnetic Resonance Imaging.PNG]

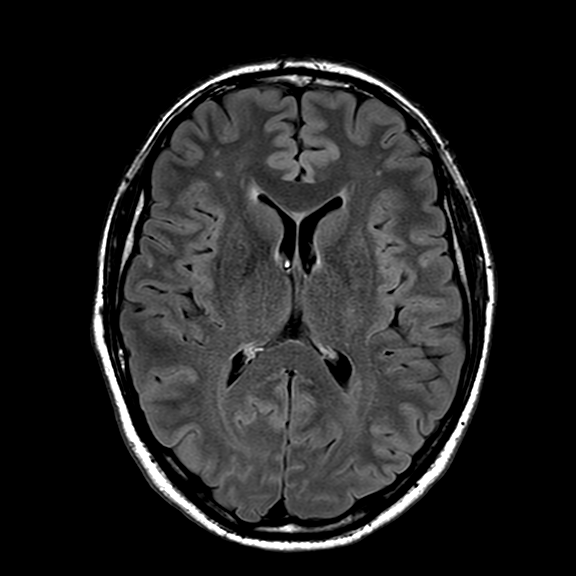

Supplement: Supplementary file 2 — Electronic Supplementary Material [file 330_2024_11115_MOESM2_ESM.zip › Digital Supplementary Material/Magnetic Resonance Imaging/19Magnetic Resonance Imaging.PNG]

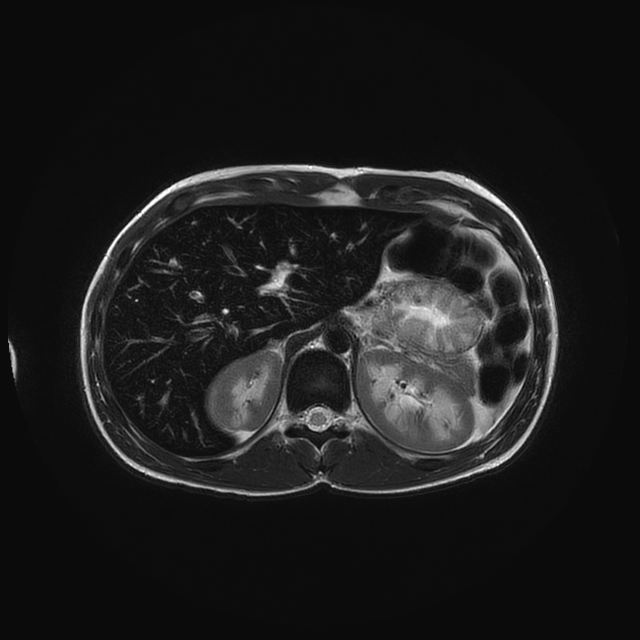

Supplement: Supplementary file 2 — Electronic Supplementary Material [file 330_2024_11115_MOESM2_ESM.zip › Digital Supplementary Material/Magnetic Resonance Imaging/1Magnetic Resonance Imaging.PNG]

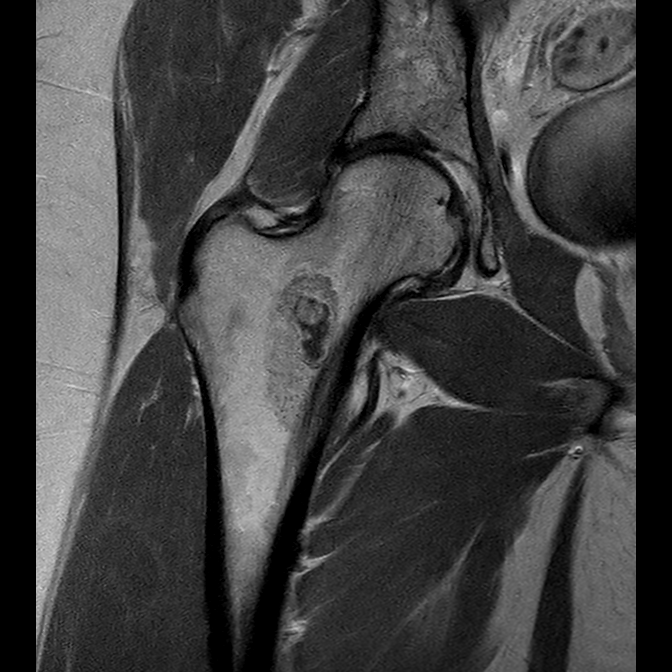

Supplement: Supplementary file 2 — Electronic Supplementary Material [file 330_2024_11115_MOESM2_ESM.zip › Digital Supplementary Material/Magnetic Resonance Imaging/34Magnetic Resonance Imaging.PNG]

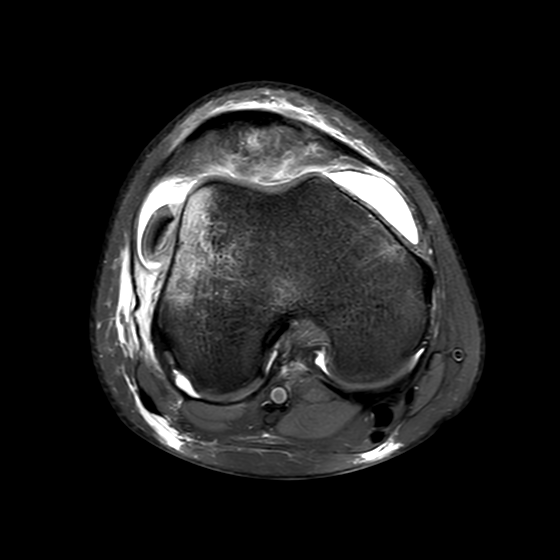

Supplement: Supplementary file 2 — Electronic Supplementary Material [file 330_2024_11115_MOESM2_ESM.zip › Digital Supplementary Material/Magnetic Resonance Imaging/11Magnetic Resonance Imaging.PNG]

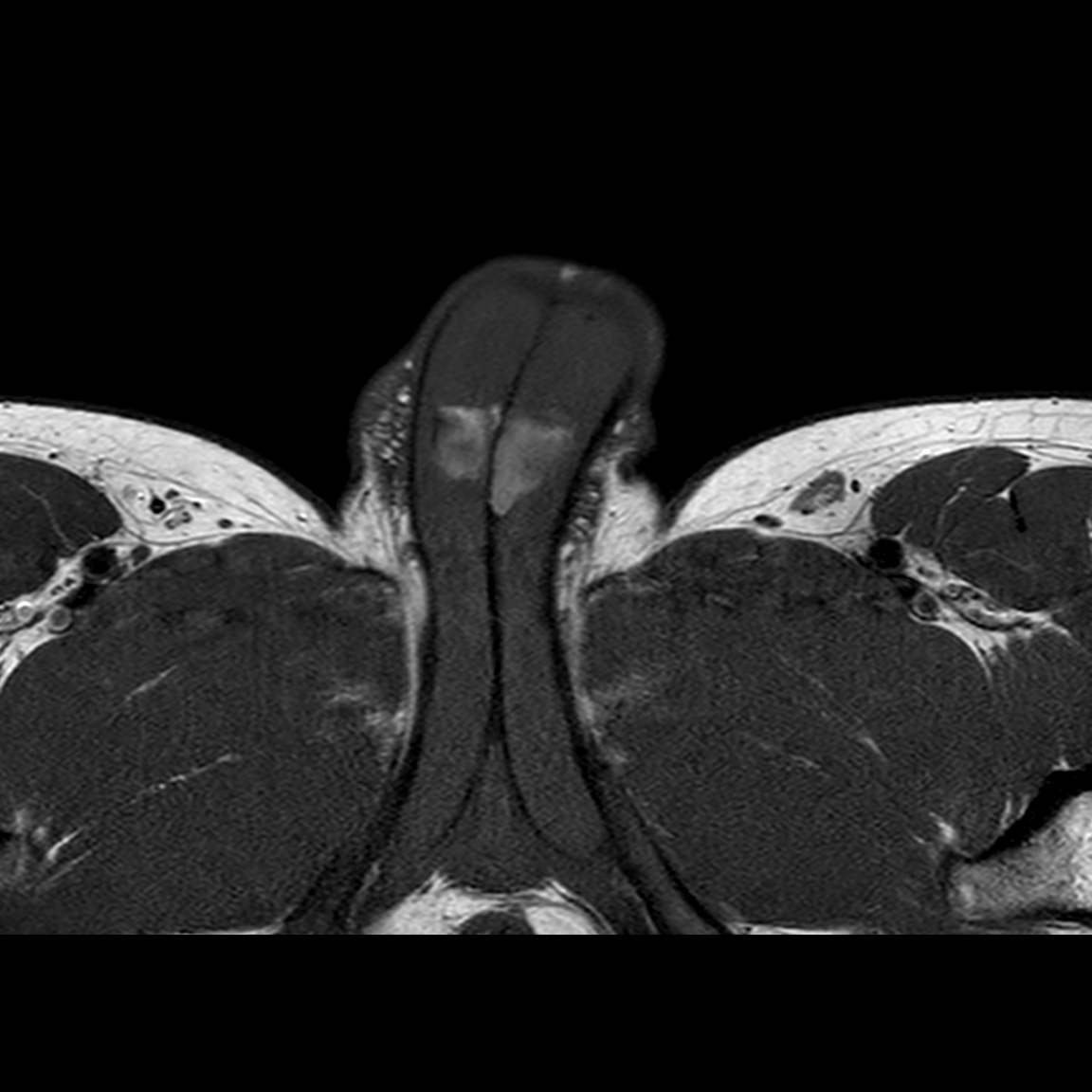

Supplement: Supplementary file 2 — Electronic Supplementary Material [file 330_2024_11115_MOESM2_ESM.zip › Digital Supplementary Material/Magnetic Resonance Imaging/54Magnetic Resonance Imaging.PNG]

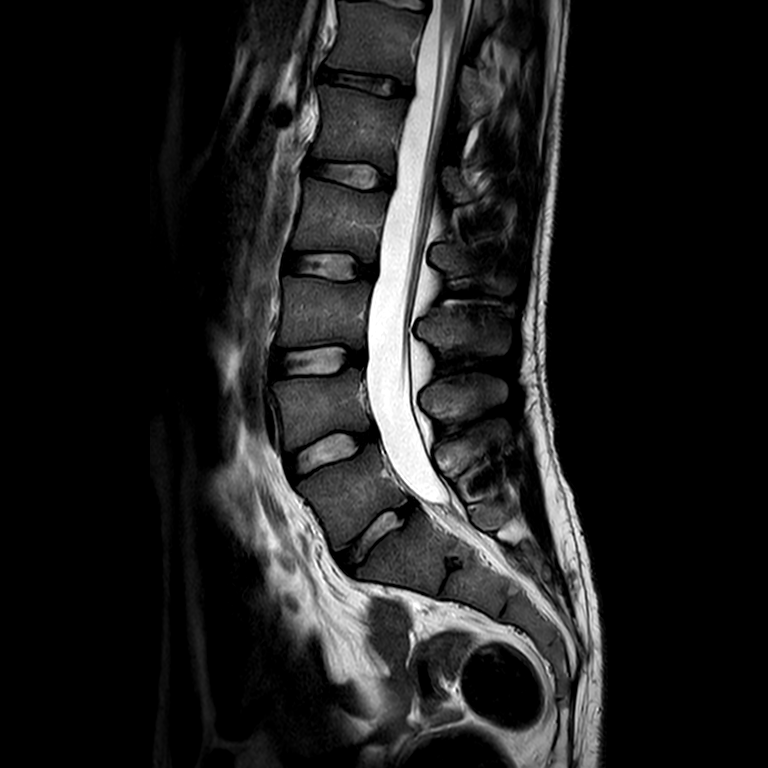

Supplement: Supplementary file 2 — Electronic Supplementary Material [file 330_2024_11115_MOESM2_ESM.zip › Digital Supplementary Material/Magnetic Resonance Imaging/9Magnetic Resonance Imaging.PNG]

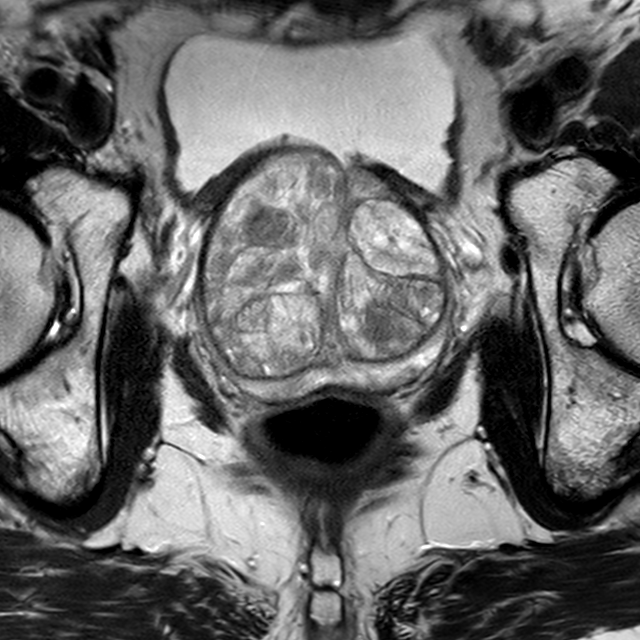

Supplement: Supplementary file 2 — Electronic Supplementary Material [file 330_2024_11115_MOESM2_ESM.zip › Digital Supplementary Material/Magnetic Resonance Imaging/60Magnetic Resonance Imaging.PNG]

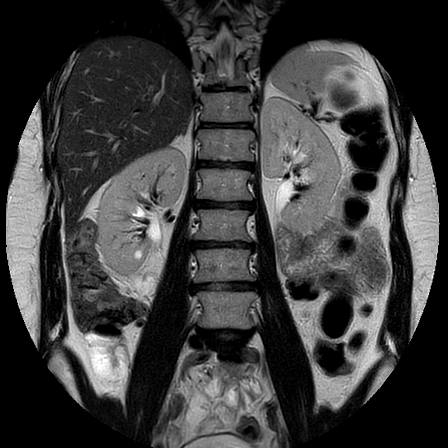

Supplement: Supplementary file 2 — Electronic Supplementary Material [file 330_2024_11115_MOESM2_ESM.zip › Digital Supplementary Material/Magnetic Resonance Imaging/25Magnetic Resonance Imaging.PNG]

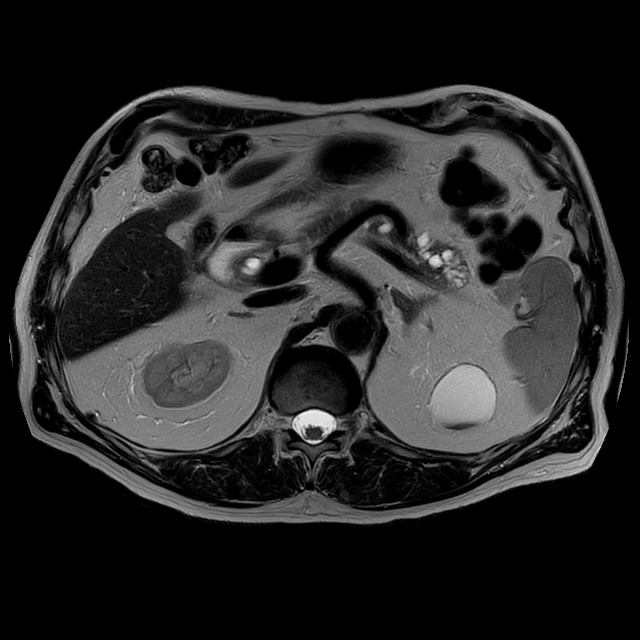

Supplement: Supplementary file 2 — Electronic Supplementary Material [file 330_2024_11115_MOESM2_ESM.zip › Digital Supplementary Material/Magnetic Resonance Imaging/45Magnetic Resonance Imaging.PNG]

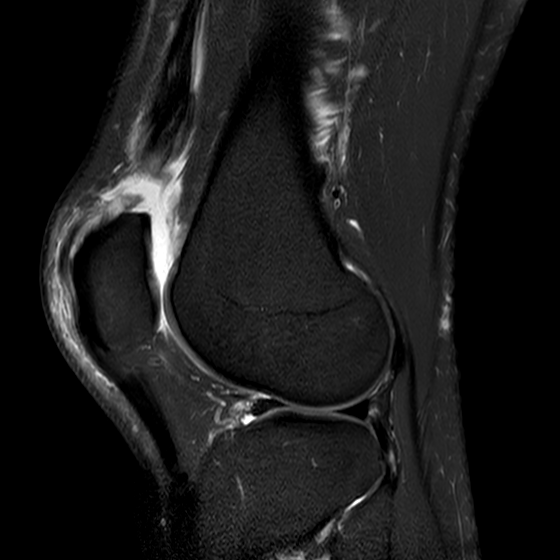

Supplement: Supplementary file 2 — Electronic Supplementary Material [file 330_2024_11115_MOESM2_ESM.zip › Digital Supplementary Material/Magnetic Resonance Imaging/6Magnetic Resonance Imaging.PNG]

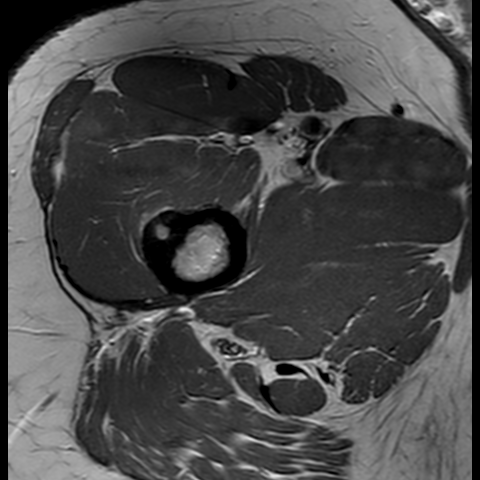

Supplement: Supplementary file 2 — Electronic Supplementary Material [file 330_2024_11115_MOESM2_ESM.zip › Digital Supplementary Material/Magnetic Resonance Imaging/43Magnetic Resonance Imaging.PNG]

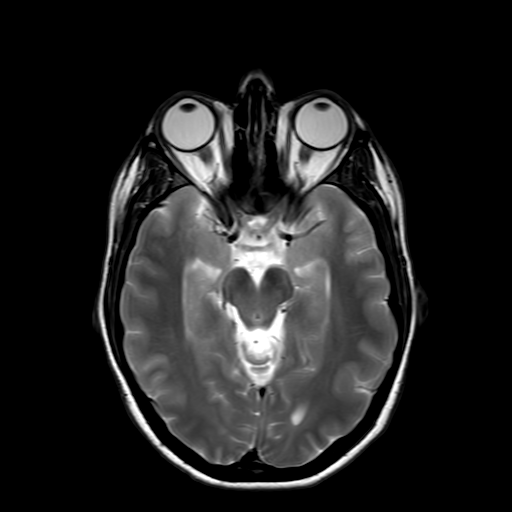

Supplement: Supplementary file 2 — Electronic Supplementary Material [file 330_2024_11115_MOESM2_ESM.zip › Digital Supplementary Material/Magnetic Resonance Imaging/18Magnetic Resonance Imaging.PNG]

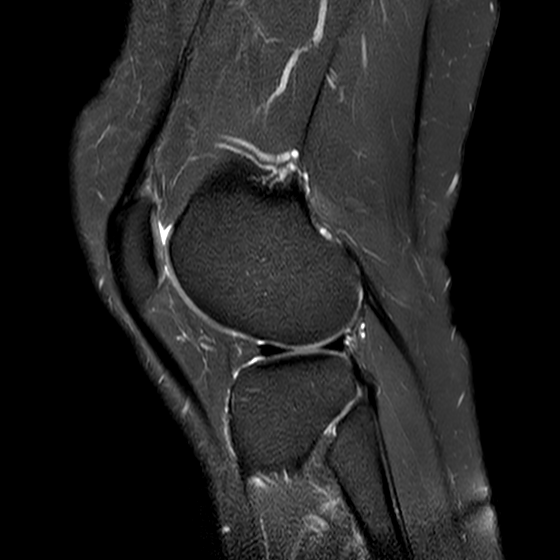

Supplement: Supplementary file 2 — Electronic Supplementary Material [file 330_2024_11115_MOESM2_ESM.zip › Digital Supplementary Material/Magnetic Resonance Imaging/23Magnetic Resonance Imaging.PNG]

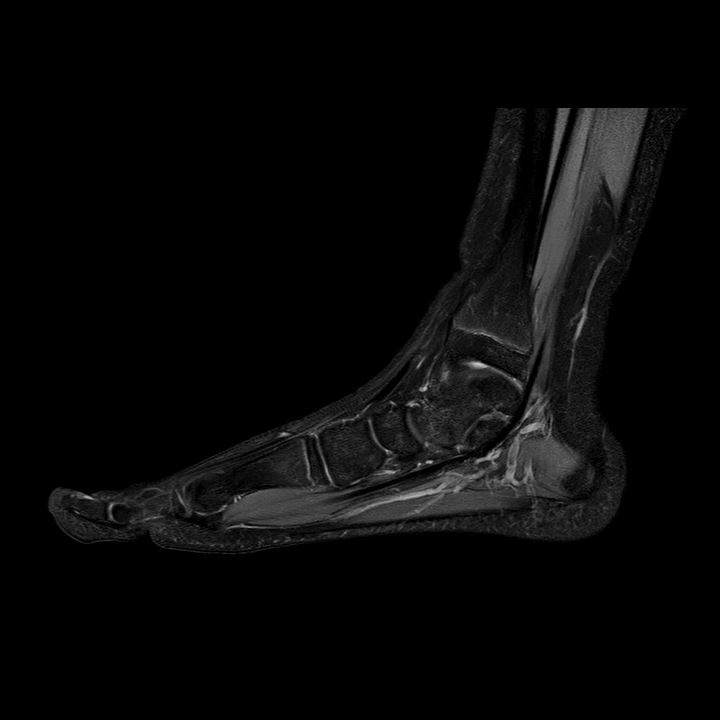

Supplement: Supplementary file 2 — Electronic Supplementary Material [file 330_2024_11115_MOESM2_ESM.zip › Digital Supplementary Material/Magnetic Resonance Imaging/17Magnetic Resonance Imaging.PNG]

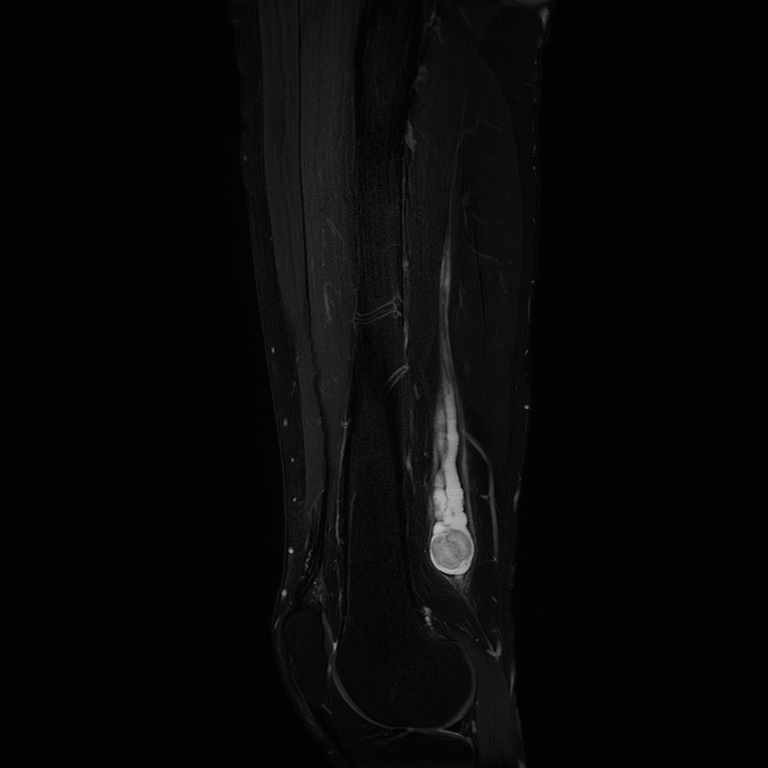

Supplement: Supplementary file 2 — Electronic Supplementary Material [file 330_2024_11115_MOESM2_ESM.zip › Digital Supplementary Material/Magnetic Resonance Imaging/52Magnetic Resonance Imaging.PNG]

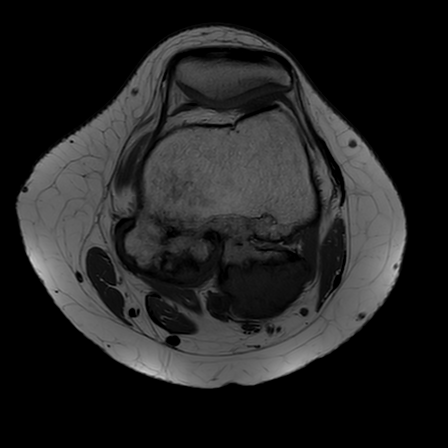

Supplement: Supplementary file 2 — Electronic Supplementary Material [file 330_2024_11115_MOESM2_ESM.zip › Digital Supplementary Material/Magnetic Resonance Imaging/32Magnetic Resonance Imaging.PNG]

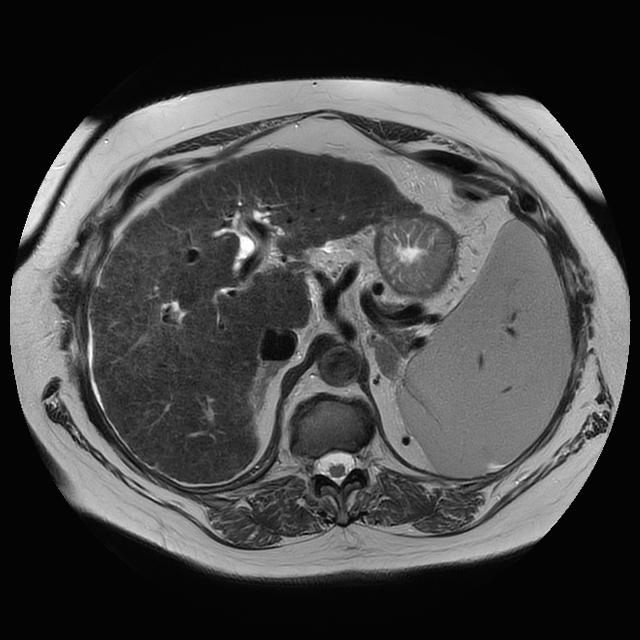

Supplement: Supplementary file 2 — Electronic Supplementary Material [file 330_2024_11115_MOESM2_ESM.zip › Digital Supplementary Material/Magnetic Resonance Imaging/44Magnetic Resonance Imaging.PNG]

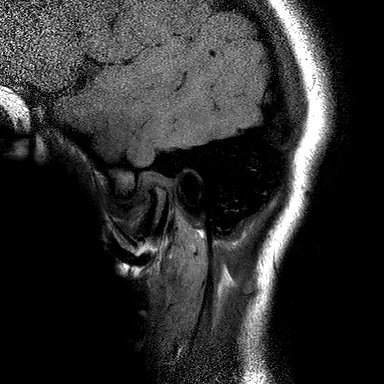

Supplement: Supplementary file 2 — Electronic Supplementary Material [file 330_2024_11115_MOESM2_ESM.zip › Digital Supplementary Material/Magnetic Resonance Imaging/7Magnetic Resonance Imaging.PNG]

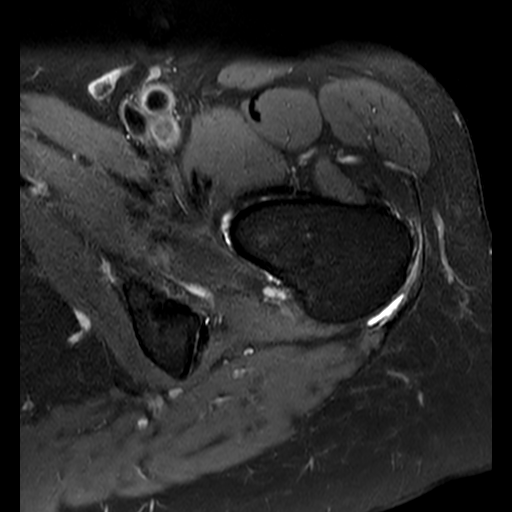

Supplement: Supplementary file 2 — Electronic Supplementary Material [file 330_2024_11115_MOESM2_ESM.zip › Digital Supplementary Material/Magnetic Resonance Imaging/24Magnetic Resonance Imaging.PNG]

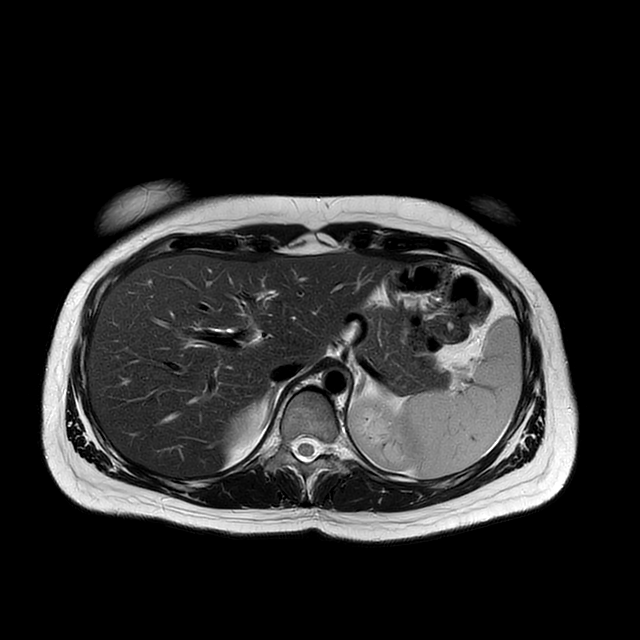

Supplement: Supplementary file 2 — Electronic Supplementary Material [file 330_2024_11115_MOESM2_ESM.zip › Digital Supplementary Material/Magnetic Resonance Imaging/10Magnetic Resonance Imaging.PNG]

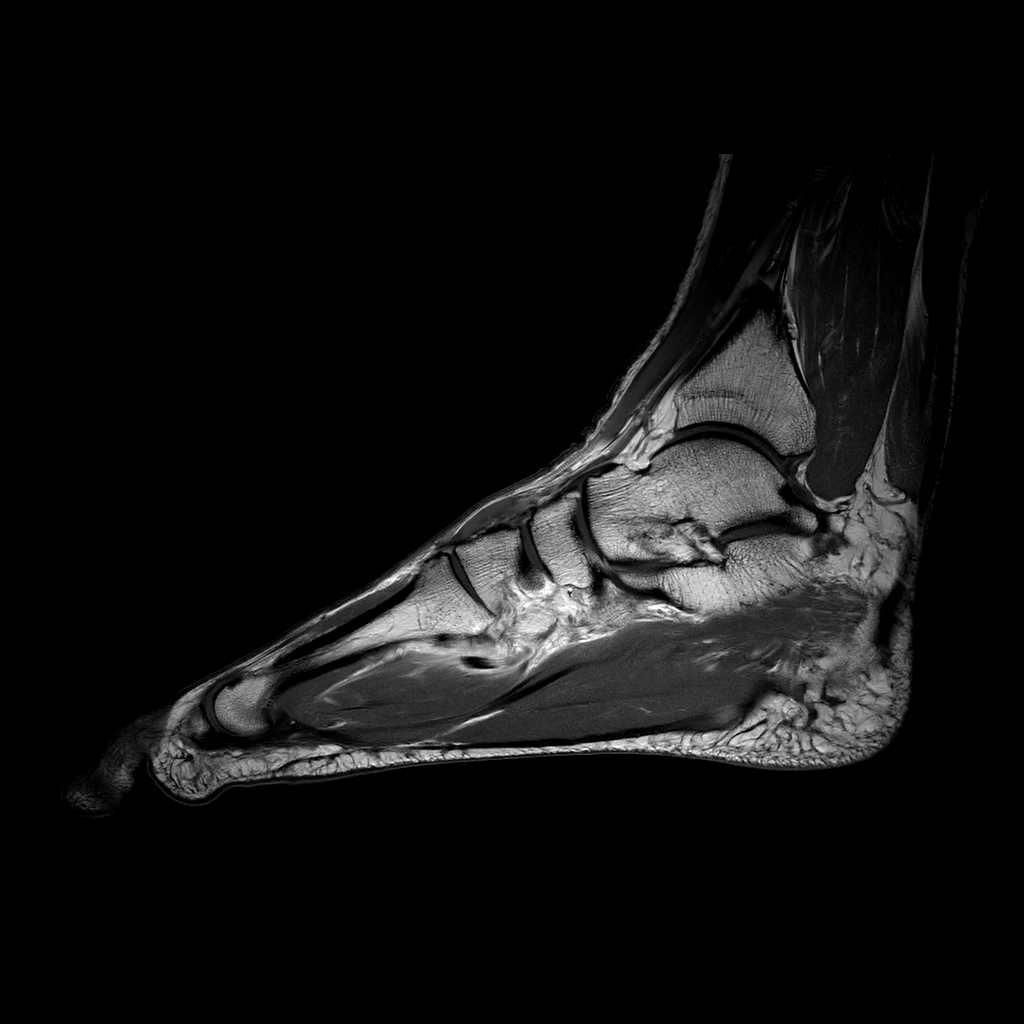

Supplement: Supplementary file 2 — Electronic Supplementary Material [file 330_2024_11115_MOESM2_ESM.zip › Digital Supplementary Material/Magnetic Resonance Imaging/55Magnetic Resonance Imaging.PNG]

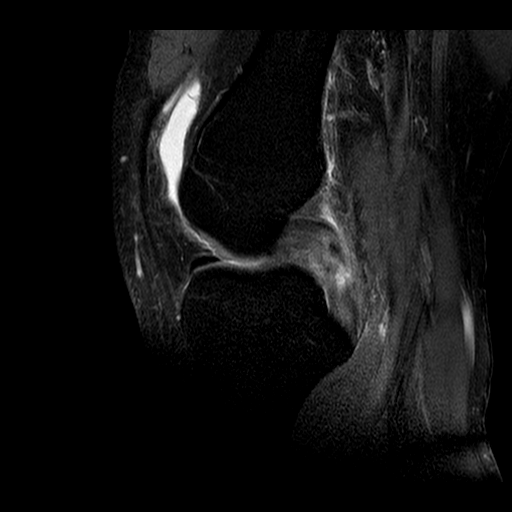

Supplement: Supplementary file 2 — Electronic Supplementary Material [file 330_2024_11115_MOESM2_ESM.zip › Digital Supplementary Material/Magnetic Resonance Imaging/8Magnetic Resonance Imaging.PNG]

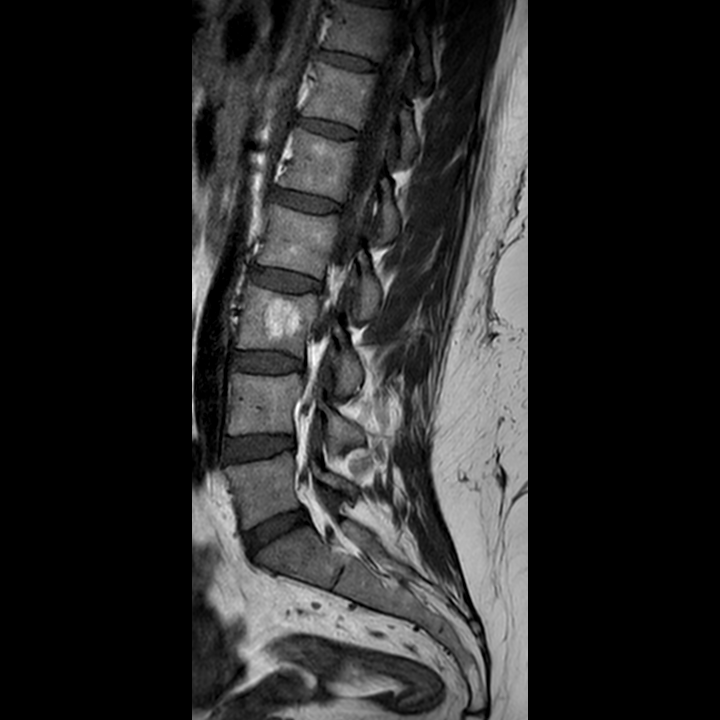

Supplement: Supplementary file 2 — Electronic Supplementary Material [file 330_2024_11115_MOESM2_ESM.zip › Digital Supplementary Material/Magnetic Resonance Imaging/35Magnetic Resonance Imaging.PNG]

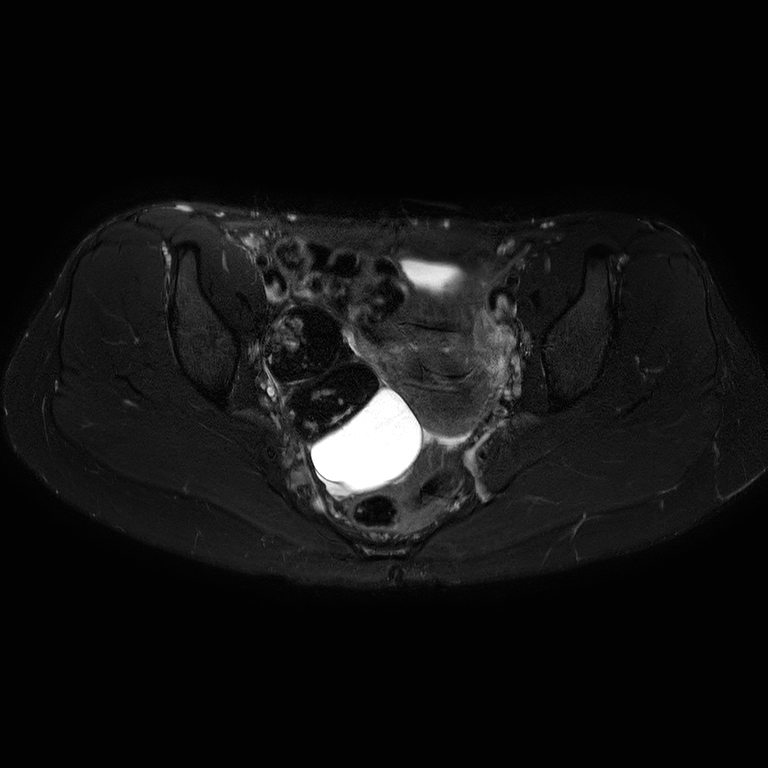

Supplement: Supplementary file 2 — Electronic Supplementary Material [file 330_2024_11115_MOESM2_ESM.zip › Digital Supplementary Material/Magnetic Resonance Imaging/47Magnetic Resonance Imaging.PNG]

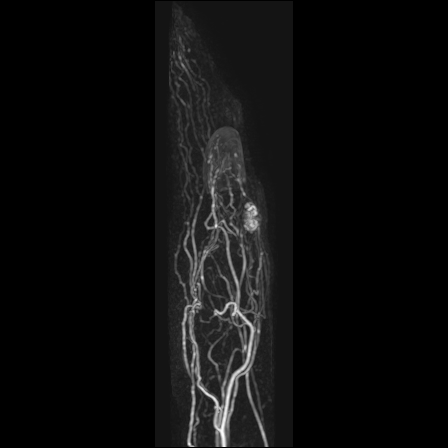

Supplement: Supplementary file 2 — Electronic Supplementary Material [file 330_2024_11115_MOESM2_ESM.zip › Digital Supplementary Material/Magnetic Resonance Imaging/59Magnetic Resonance Imaging.PNG]

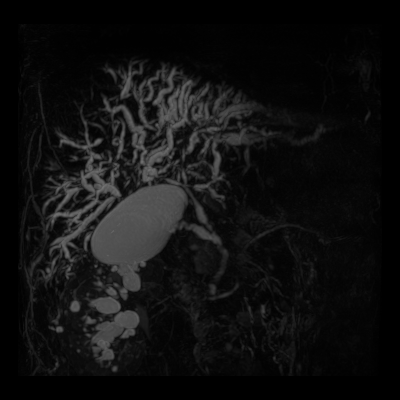

Supplement: Supplementary file 2 — Electronic Supplementary Material [file 330_2024_11115_MOESM2_ESM.zip › Digital Supplementary Material/Magnetic Resonance Imaging/4Magnetic Resonance Imaging.PNG]

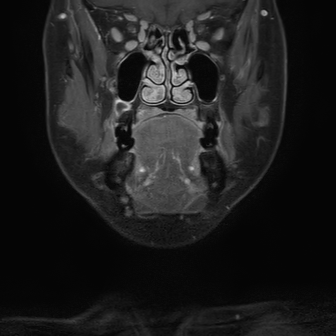

Supplement: Supplementary file 2 — Electronic Supplementary Material [file 330_2024_11115_MOESM2_ESM.zip › Digital Supplementary Material/Magnetic Resonance Imaging/39Magnetic Resonance Imaging.PNG]

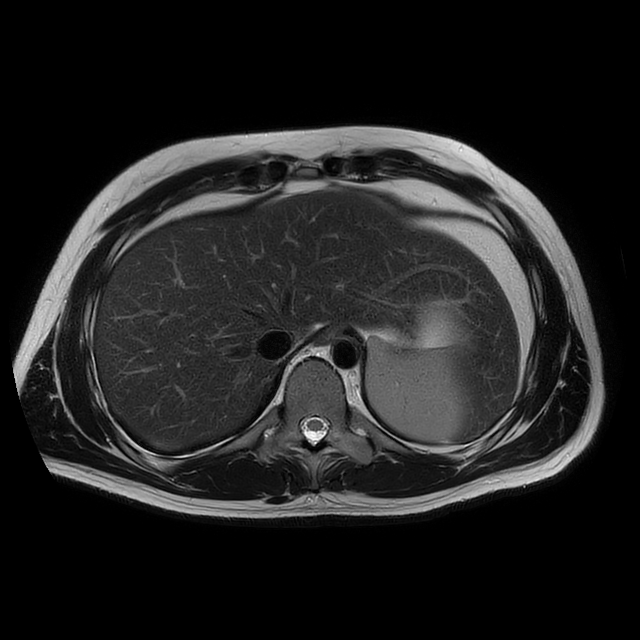

Supplement: Supplementary file 2 — Electronic Supplementary Material [file 330_2024_11115_MOESM2_ESM.zip › Digital Supplementary Material/Magnetic Resonance Imaging/27Magnetic Resonance Imaging.PNG]

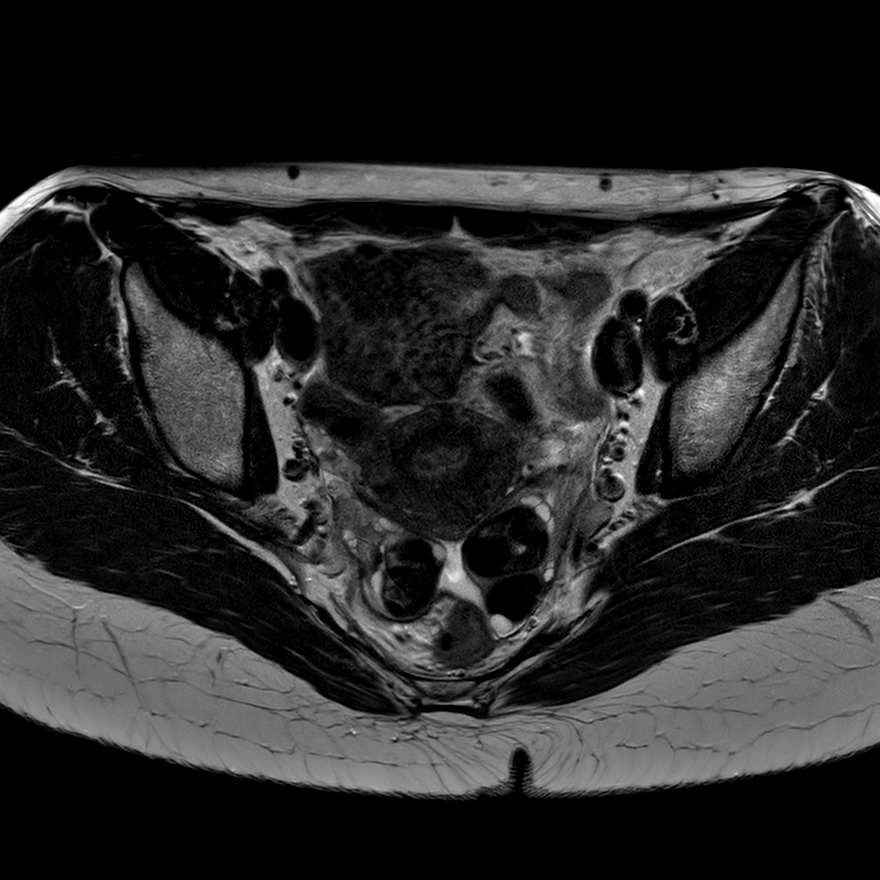

Supplement: Supplementary file 2 — Electronic Supplementary Material [file 330_2024_11115_MOESM2_ESM.zip › Digital Supplementary Material/Magnetic Resonance Imaging/48Magnetic Resonance Imaging.PNG]

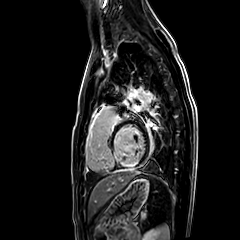

Supplement: Supplementary file 2 — Electronic Supplementary Material [file 330_2024_11115_MOESM2_ESM.zip › Digital Supplementary Material/Magnetic Resonance Imaging/13Magnetic Resonance Imaging.PNG]

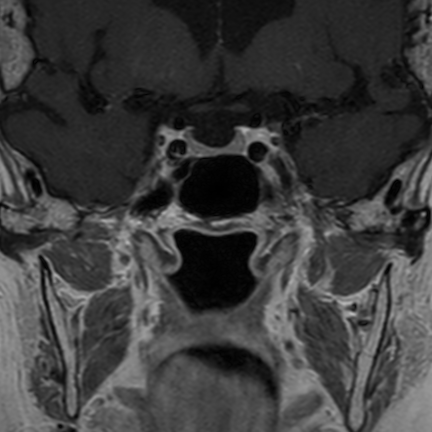

Supplement: Supplementary file 2 — Electronic Supplementary Material [file 330_2024_11115_MOESM2_ESM.zip › Digital Supplementary Material/Magnetic Resonance Imaging/56Magnetic Resonance Imaging.PNG]

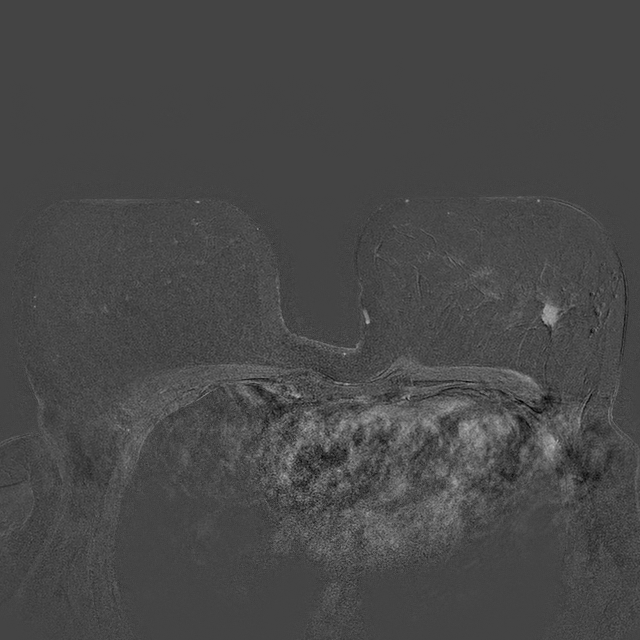

Supplement: Supplementary file 2 — Electronic Supplementary Material [file 330_2024_11115_MOESM2_ESM.zip › Digital Supplementary Material/Magnetic Resonance Imaging/36Magnetic Resonance Imaging.PNG]

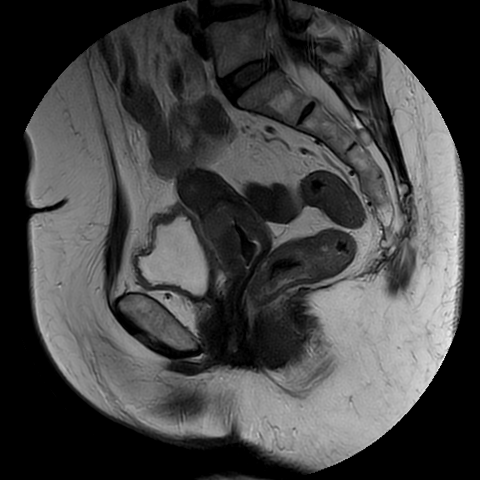

Supplement: Supplementary file 2 — Electronic Supplementary Material [file 330_2024_11115_MOESM2_ESM.zip › Digital Supplementary Material/Magnetic Resonance Imaging/28Magnetic Resonance Imaging.PNG]

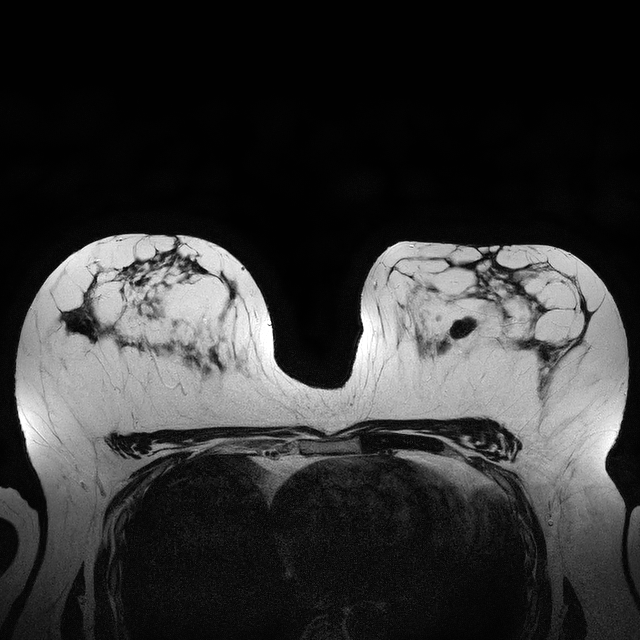

Supplement: Supplementary file 2 — Electronic Supplementary Material [file 330_2024_11115_MOESM2_ESM.zip › Digital Supplementary Material/Magnetic Resonance Imaging/40Magnetic Resonance Imaging.PNG]

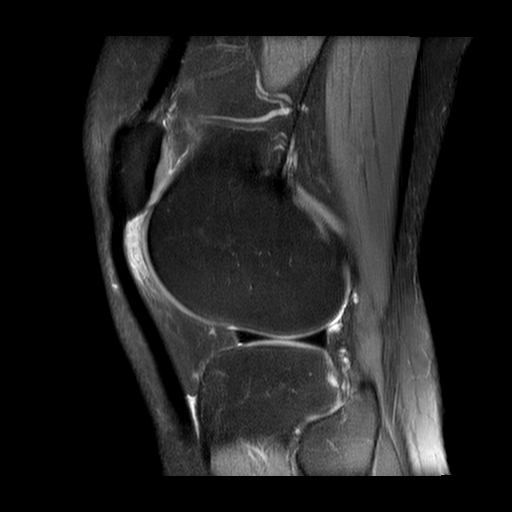

Supplement: Supplementary file 2 — Electronic Supplementary Material [file 330_2024_11115_MOESM2_ESM.zip › Digital Supplementary Material/Magnetic Resonance Imaging/3Magnetic Resonance Imaging.PNG]

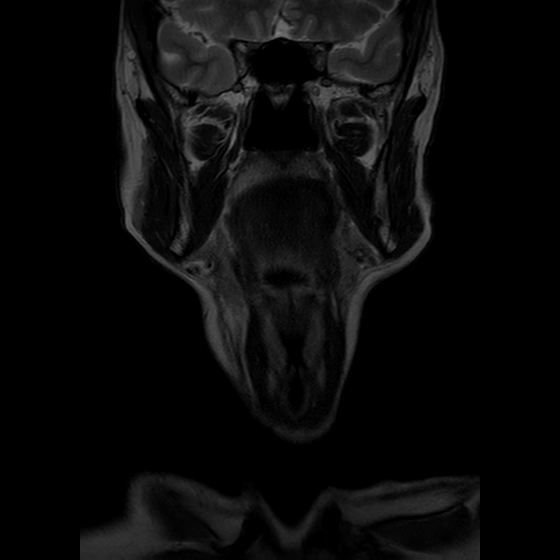

Supplement: Supplementary file 2 — Electronic Supplementary Material [file 330_2024_11115_MOESM2_ESM.zip › Digital Supplementary Material/Magnetic Resonance Imaging/20Magnetic Resonance Imaging.PNG]

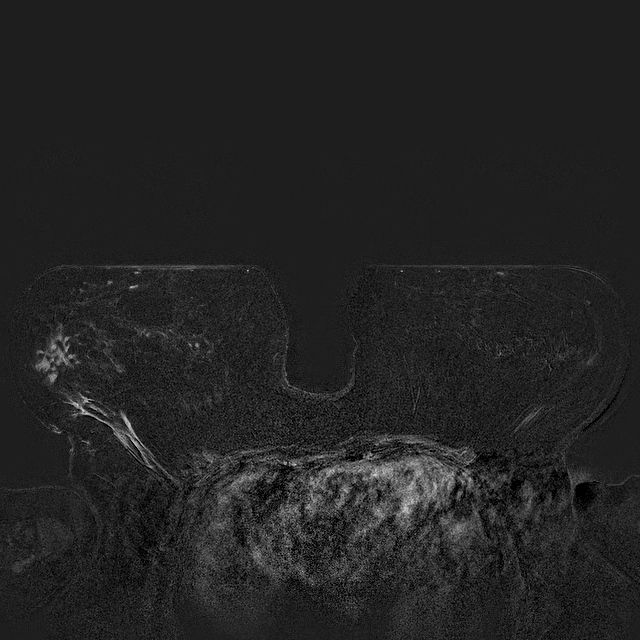

Supplement: Supplementary file 2 — Electronic Supplementary Material [file 330_2024_11115_MOESM2_ESM.zip › Digital Supplementary Material/Magnetic Resonance Imaging/14Magnetic Resonance Imaging.PNG]

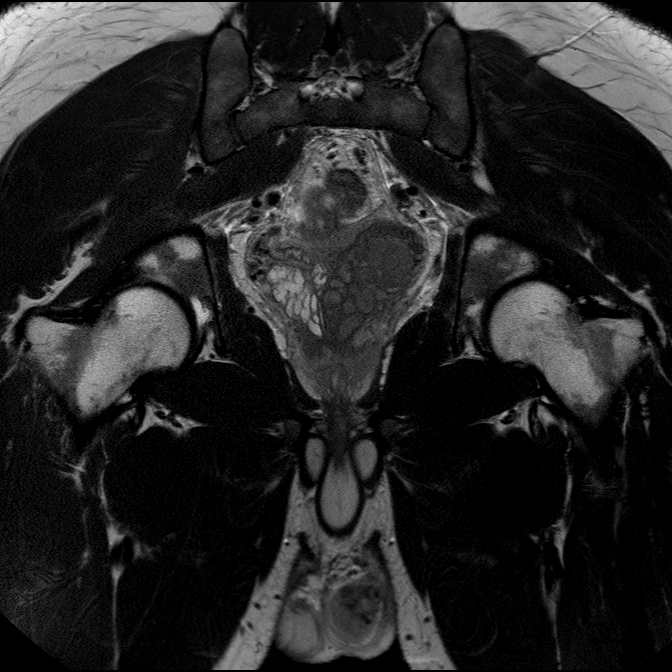

Supplement: Supplementary file 2 — Electronic Supplementary Material [file 330_2024_11115_MOESM2_ESM.zip › Digital Supplementary Material/Magnetic Resonance Imaging/51Magnetic Resonance Imaging.PNG]

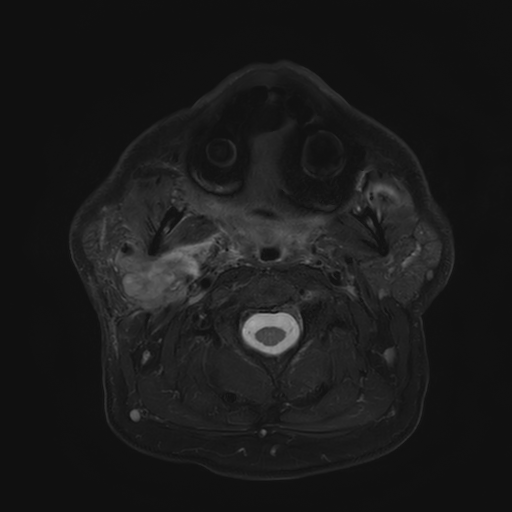

Supplement: Supplementary file 2 — Electronic Supplementary Material [file 330_2024_11115_MOESM2_ESM.zip › Digital Supplementary Material/Magnetic Resonance Imaging/31Magnetic Resonance Imaging.PNG]

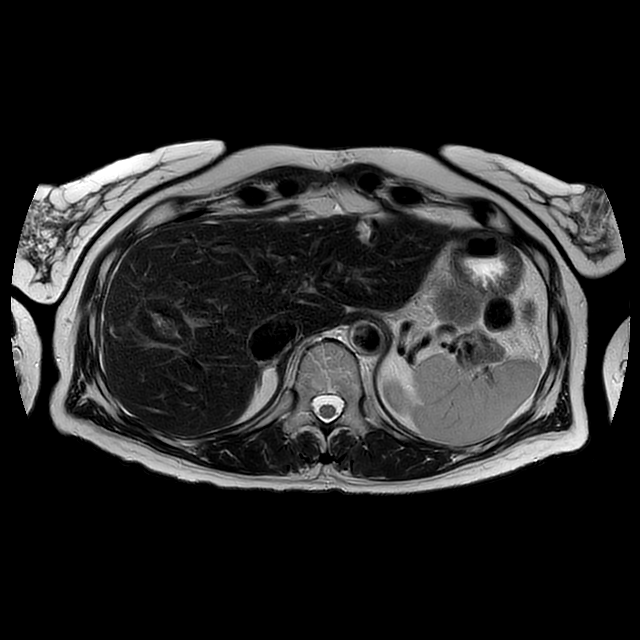

Supplement: Supplementary file 2 — Electronic Supplementary Material [file 330_2024_11115_MOESM2_ESM.zip › Digital Supplementary Material/Magnetic Resonance Imaging/37Magnetic Resonance Imaging.PNG]

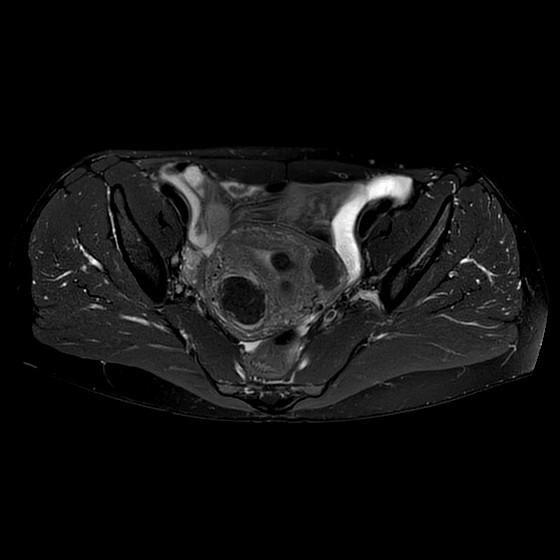

Supplement: Supplementary file 2 — Electronic Supplementary Material [file 330_2024_11115_MOESM2_ESM.zip › Digital Supplementary Material/Magnetic Resonance Imaging/29Magnetic Resonance Imaging.PNG]

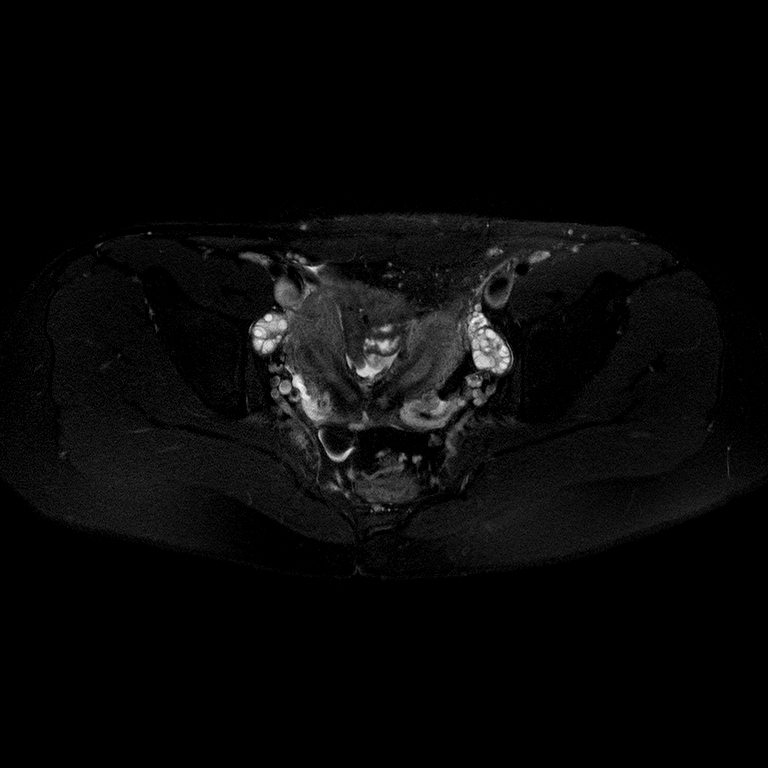

Supplement: Supplementary file 2 — Electronic Supplementary Material [file 330_2024_11115_MOESM2_ESM.zip › Digital Supplementary Material/Magnetic Resonance Imaging/49Magnetic Resonance Imaging.PNG]

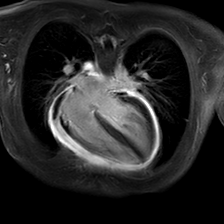

Supplement: Supplementary file 2 — Electronic Supplementary Material [file 330_2024_11115_MOESM2_ESM.zip › Digital Supplementary Material/Magnetic Resonance Imaging/12Magnetic Resonance Imaging.PNG]

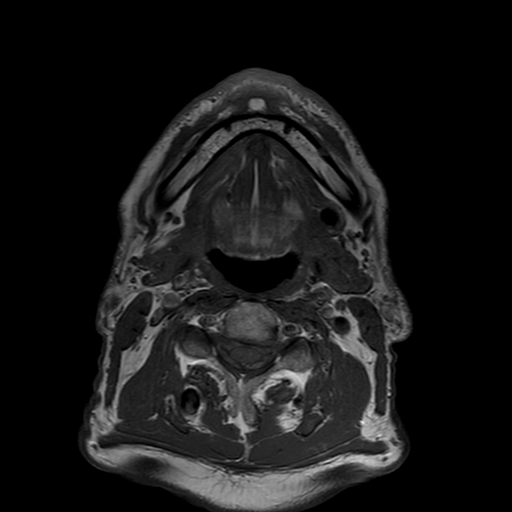

Supplement: Supplementary file 2 — Electronic Supplementary Material [file 330_2024_11115_MOESM2_ESM.zip › Digital Supplementary Material/Magnetic Resonance Imaging/57Magnetic Resonance Imaging.PNG]

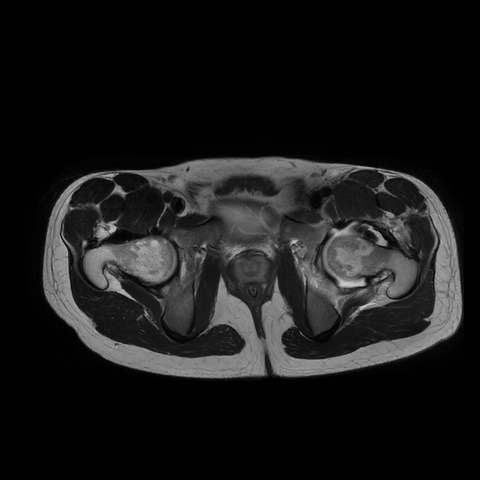

Supplement: Supplementary file 2 — Electronic Supplementary Material [file 330_2024_11115_MOESM2_ESM.zip › Digital Supplementary Material/Magnetic Resonance Imaging/38Magnetic Resonance Imaging.PNG]

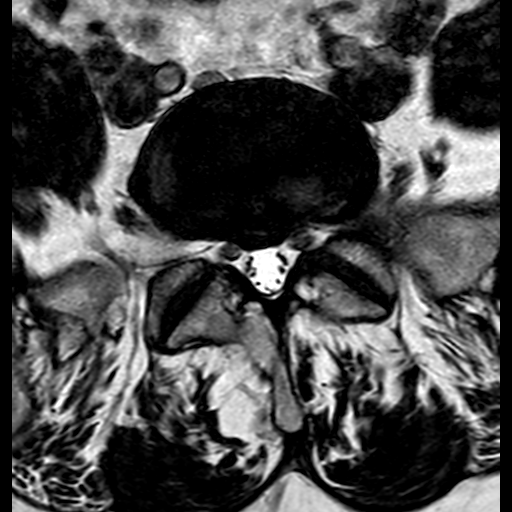

Supplement: Supplementary file 2 — Electronic Supplementary Material [file 330_2024_11115_MOESM2_ESM.zip › Digital Supplementary Material/Magnetic Resonance Imaging/26Magnetic Resonance Imaging.PNG]

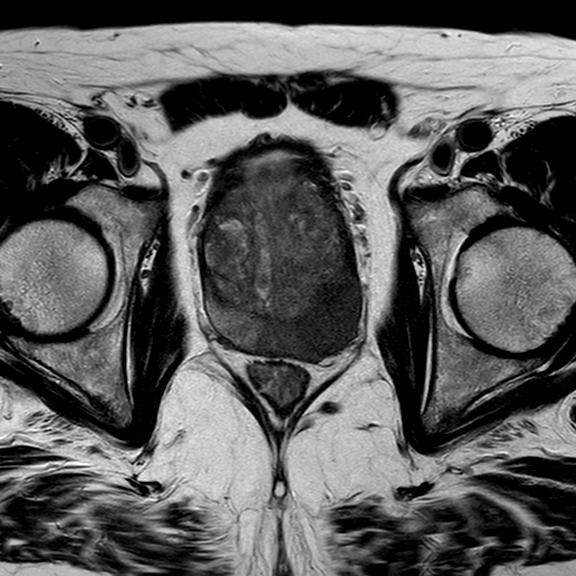

Supplement: Supplementary file 2 — Electronic Supplementary Material [file 330_2024_11115_MOESM2_ESM.zip › Digital Supplementary Material/Magnetic Resonance Imaging/46Magnetic Resonance Imaging.PNG]

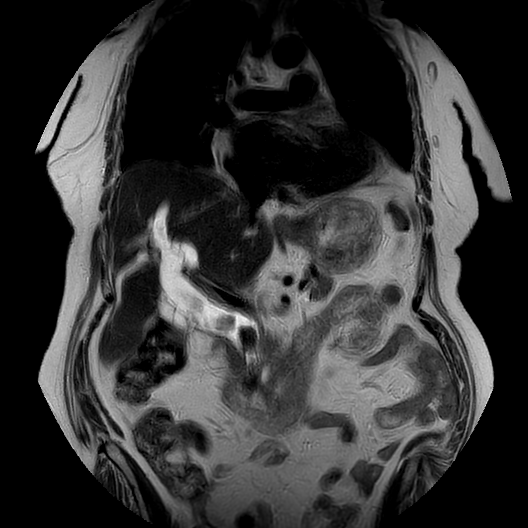

Supplement: Supplementary file 2 — Electronic Supplementary Material [file 330_2024_11115_MOESM2_ESM.zip › Digital Supplementary Material/Magnetic Resonance Imaging/58Magnetic Resonance Imaging.PNG]

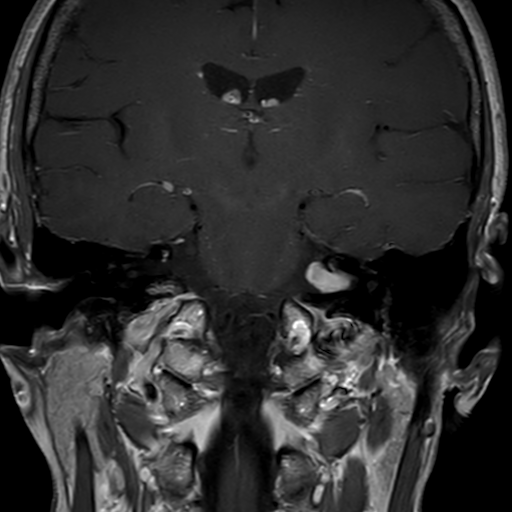

Supplement: Supplementary file 2 — Electronic Supplementary Material [file 330_2024_11115_MOESM2_ESM.zip › Digital Supplementary Material/Magnetic Resonance Imaging/5Magnetic Resonance Imaging.PNG]

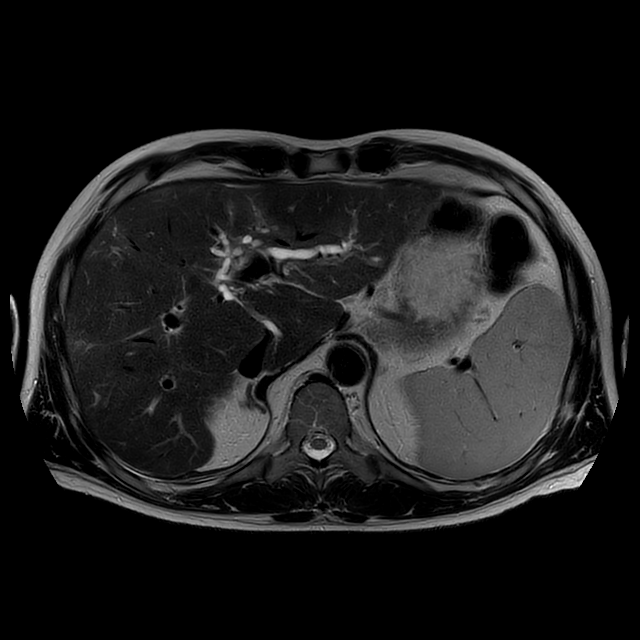

Supplement: Supplementary file 2 — Electronic Supplementary Material [file 330_2024_11115_MOESM2_ESM.zip › Digital Supplementary Material/Magnetic Resonance Imaging/30Magnetic Resonance Imaging.PNG]

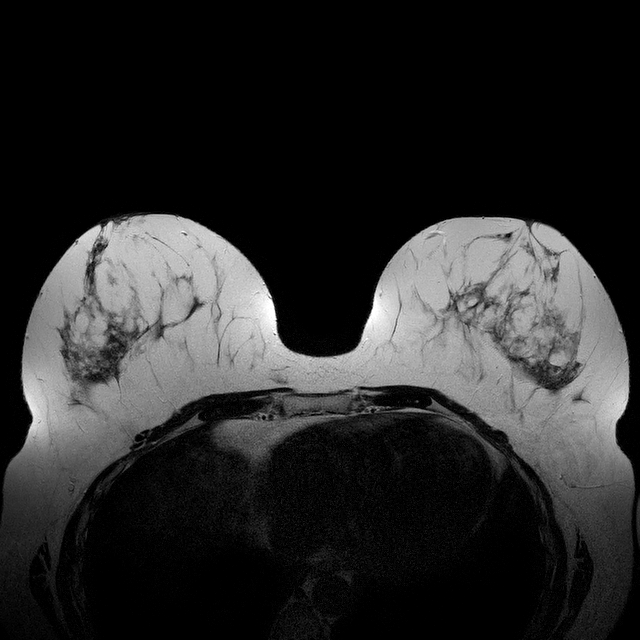

Supplement: Supplementary file 2 — Electronic Supplementary Material [file 330_2024_11115_MOESM2_ESM.zip › Digital Supplementary Material/Magnetic Resonance Imaging/15Magnetic Resonance Imaging.PNG]

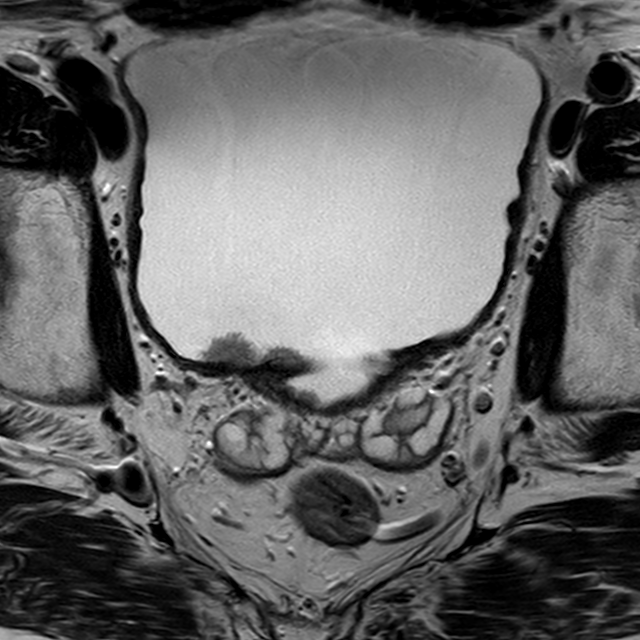

Supplement: Supplementary file 2 — Electronic Supplementary Material [file 330_2024_11115_MOESM2_ESM.zip › Digital Supplementary Material/Magnetic Resonance Imaging/50Magnetic Resonance Imaging.PNG]

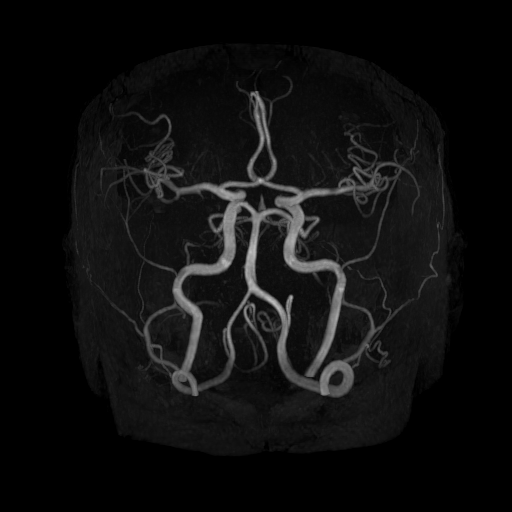

Supplement: Supplementary file 2 — Electronic Supplementary Material [file 330_2024_11115_MOESM2_ESM.zip › Digital Supplementary Material/Magnetic Resonance Imaging/21Magnetic Resonance Imaging.PNG]

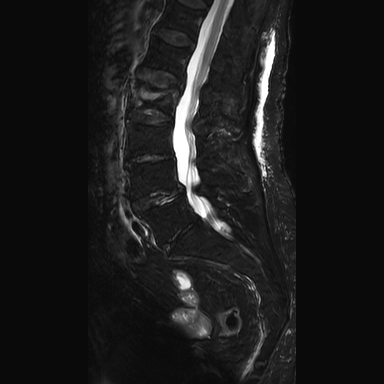

Supplement: Supplementary file 2 — Electronic Supplementary Material [file 330_2024_11115_MOESM2_ESM.zip › Digital Supplementary Material/Magnetic Resonance Imaging/41Magnetic Resonance Imaging.PNG]

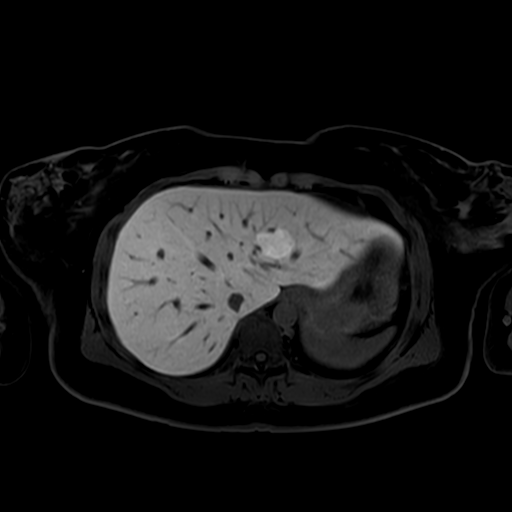

Supplement: Supplementary file 2 — Electronic Supplementary Material [file 330_2024_11115_MOESM2_ESM.zip › Digital Supplementary Material/Magnetic Resonance Imaging/2Magnetic Resonance Imaging.PNG]

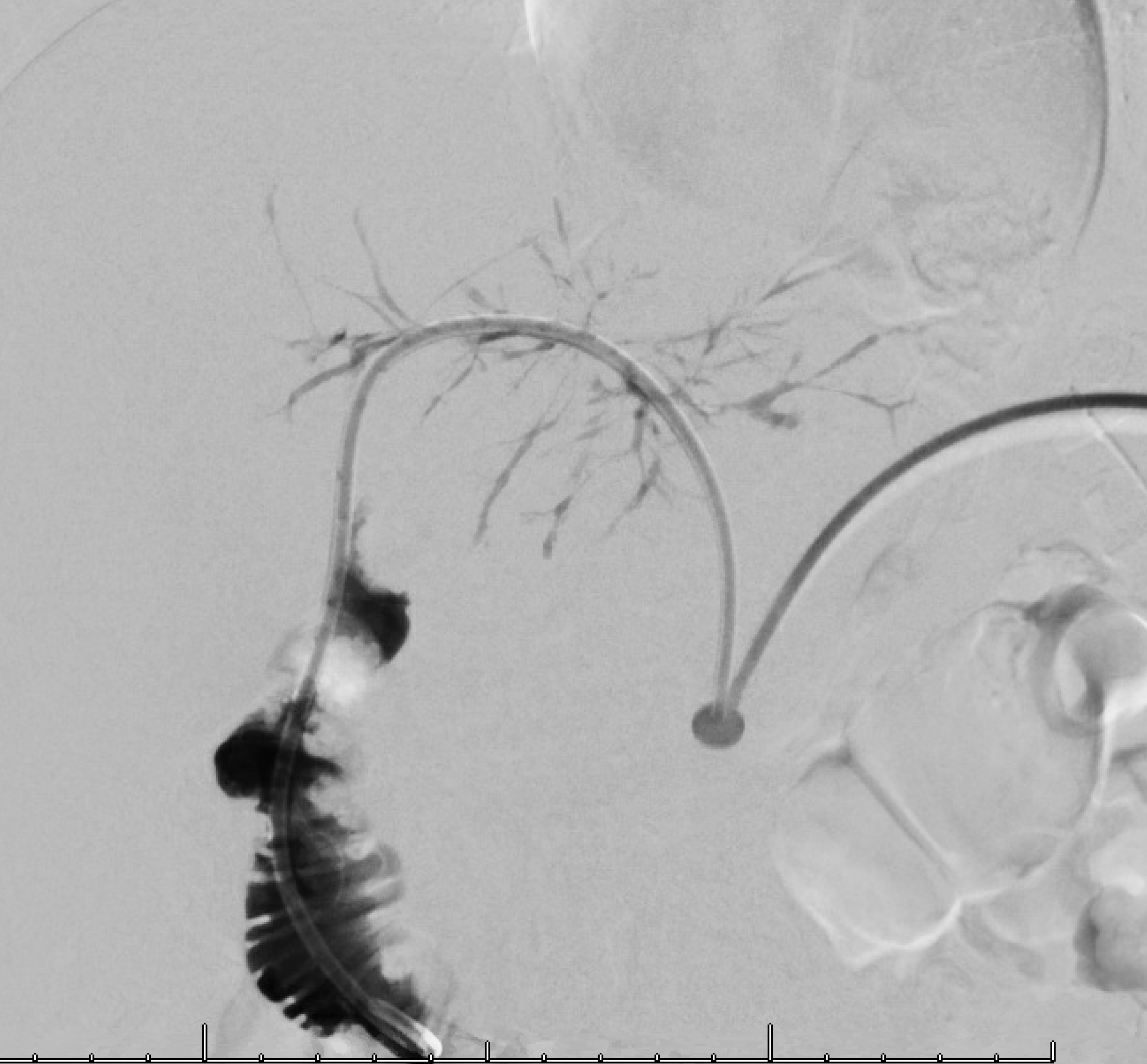

Supplement: Supplementary file 2 — Electronic Supplementary Material [file 330_2024_11115_MOESM2_ESM.zip › Digital Supplementary Material/Angiography/16Angiography.PNG]

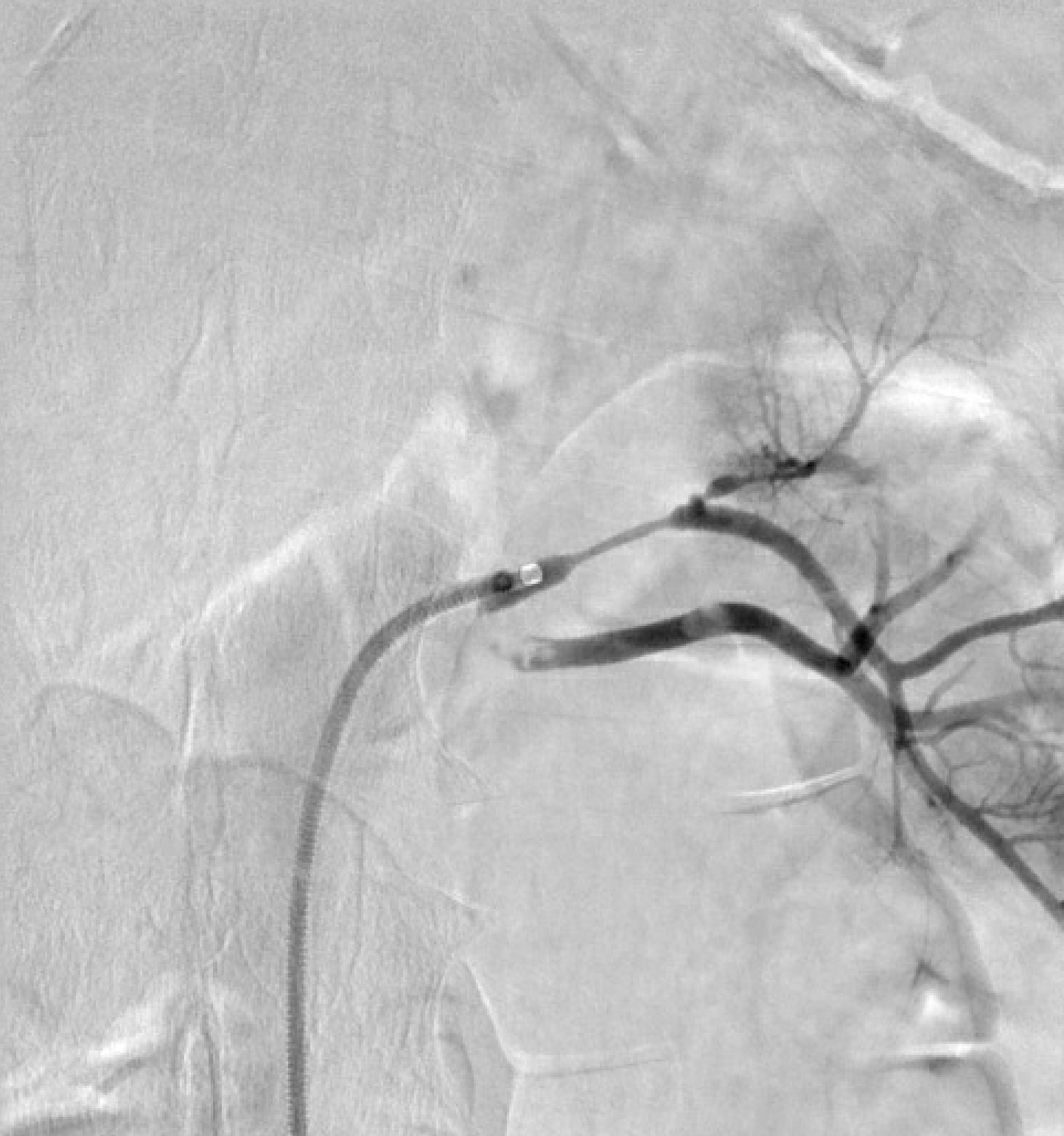

Supplement: Supplementary file 2 — Electronic Supplementary Material [file 330_2024_11115_MOESM2_ESM.zip › Digital Supplementary Material/Angiography/19Angiography.PNG]

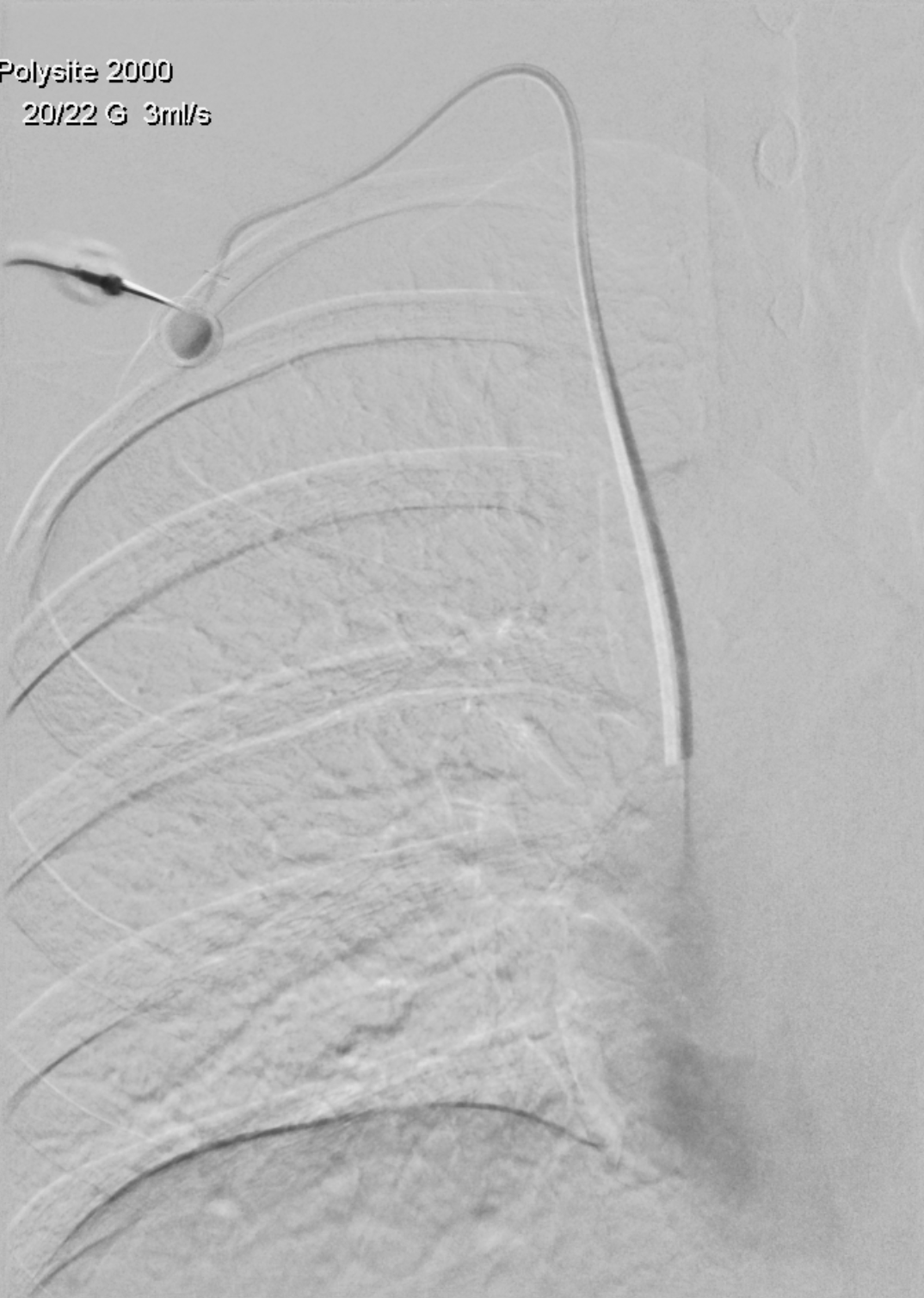

Supplement: Supplementary file 2 — Electronic Supplementary Material [file 330_2024_11115_MOESM2_ESM.zip › Digital Supplementary Material/Angiography/11Angiography.PNG]

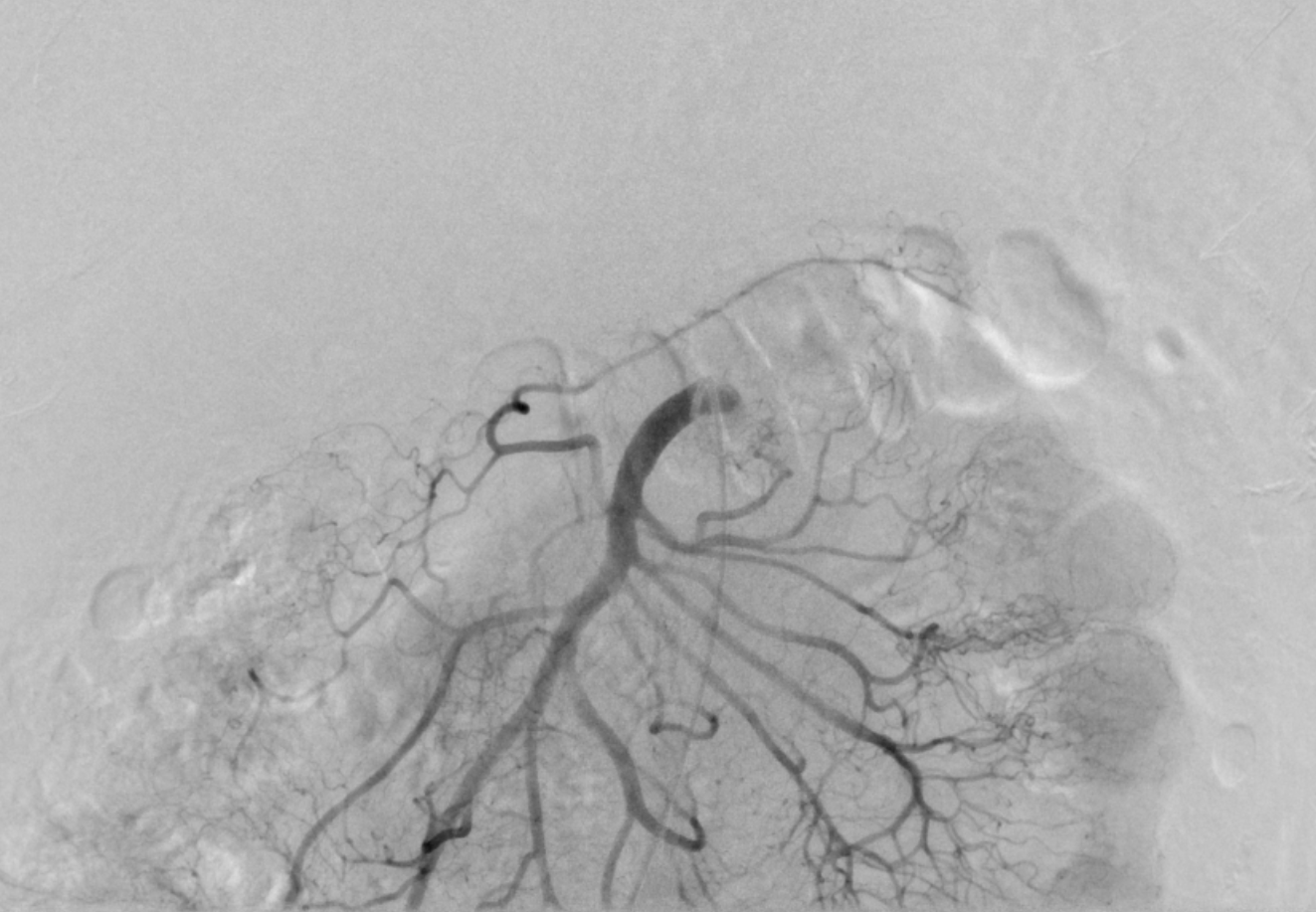

Supplement: Supplementary file 2 — Electronic Supplementary Material [file 330_2024_11115_MOESM2_ESM.zip › Digital Supplementary Material/Angiography/10Angiography.PNG]

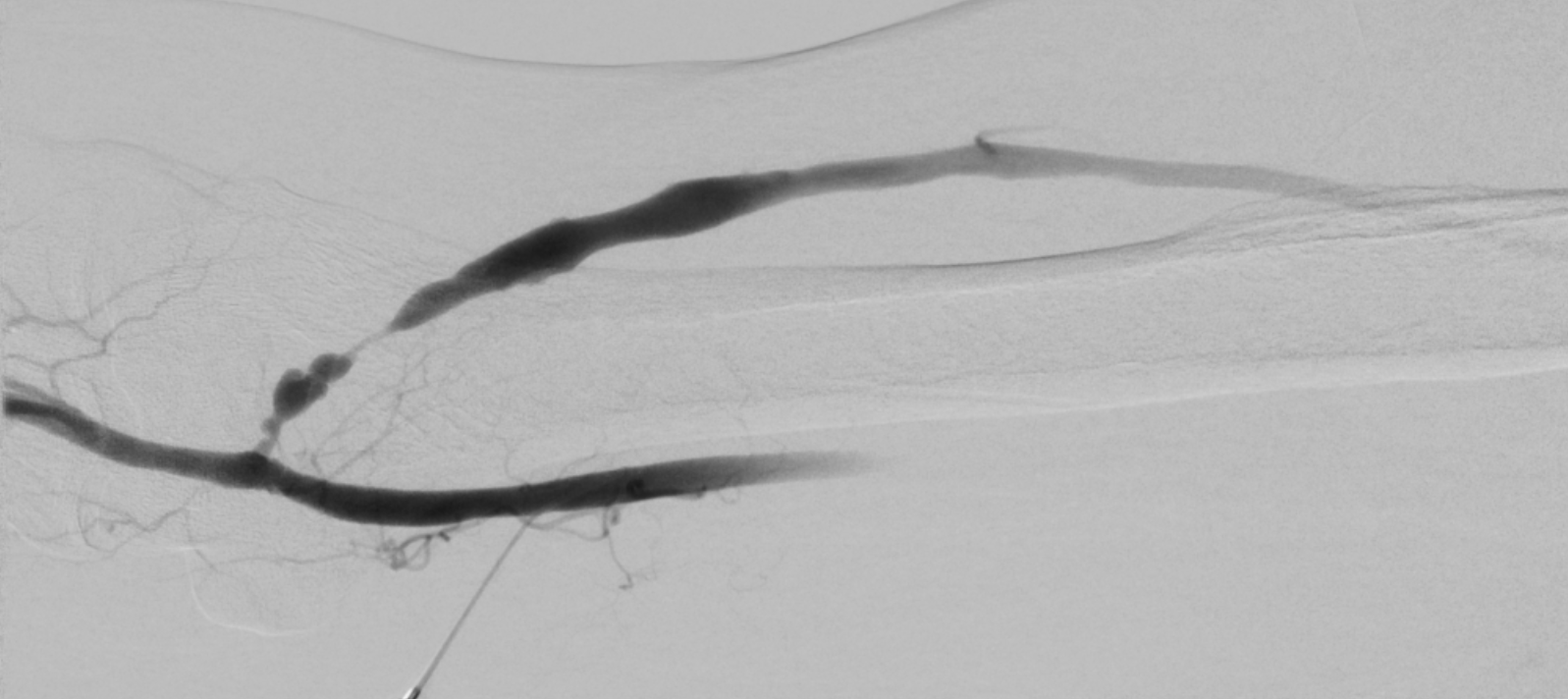

Supplement: Supplementary file 2 — Electronic Supplementary Material [file 330_2024_11115_MOESM2_ESM.zip › Digital Supplementary Material/Angiography/18Angiography.PNG]

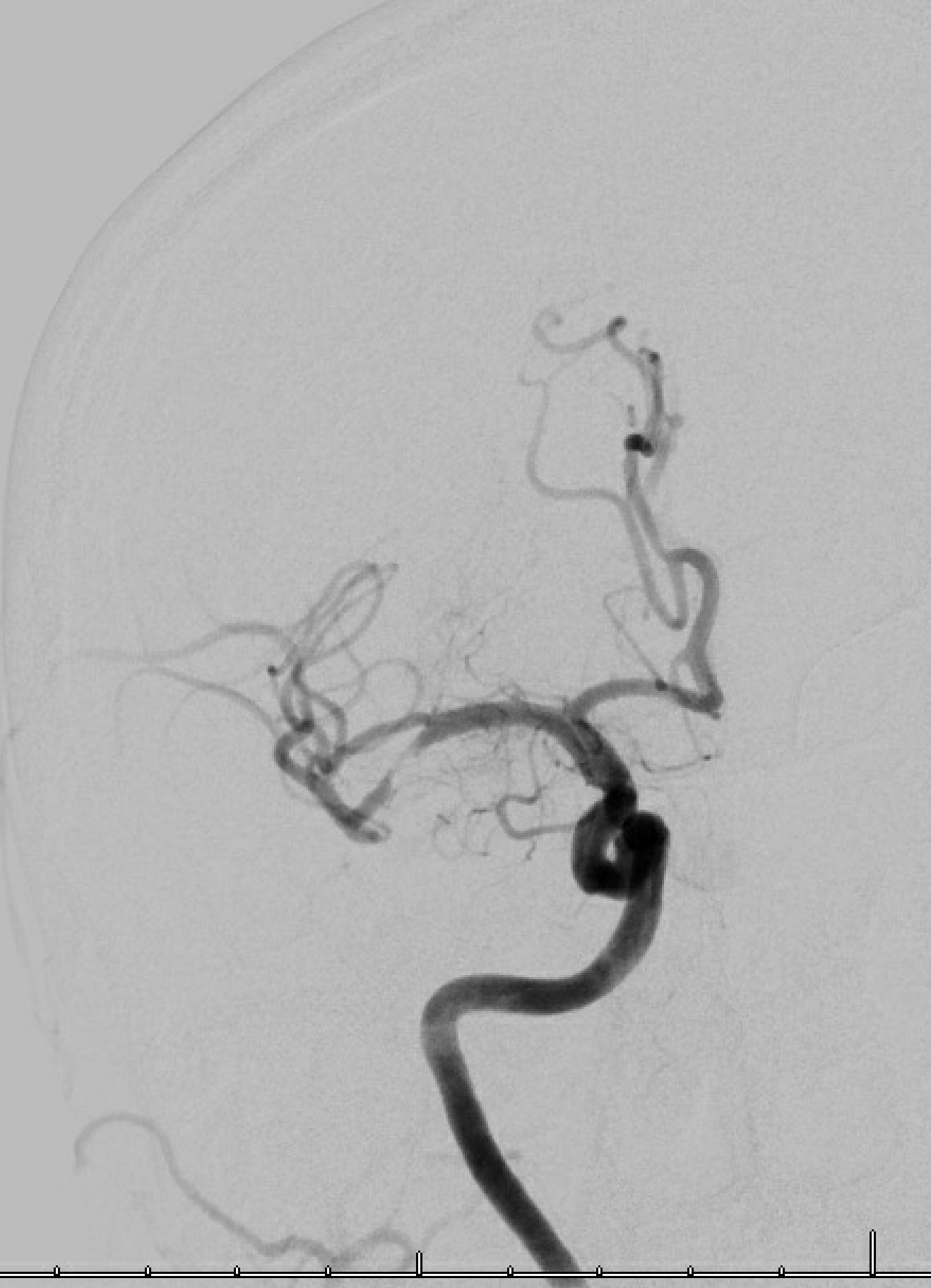

Supplement: Supplementary file 2 — Electronic Supplementary Material [file 330_2024_11115_MOESM2_ESM.zip › Digital Supplementary Material/Angiography/17Angiography.PNG]

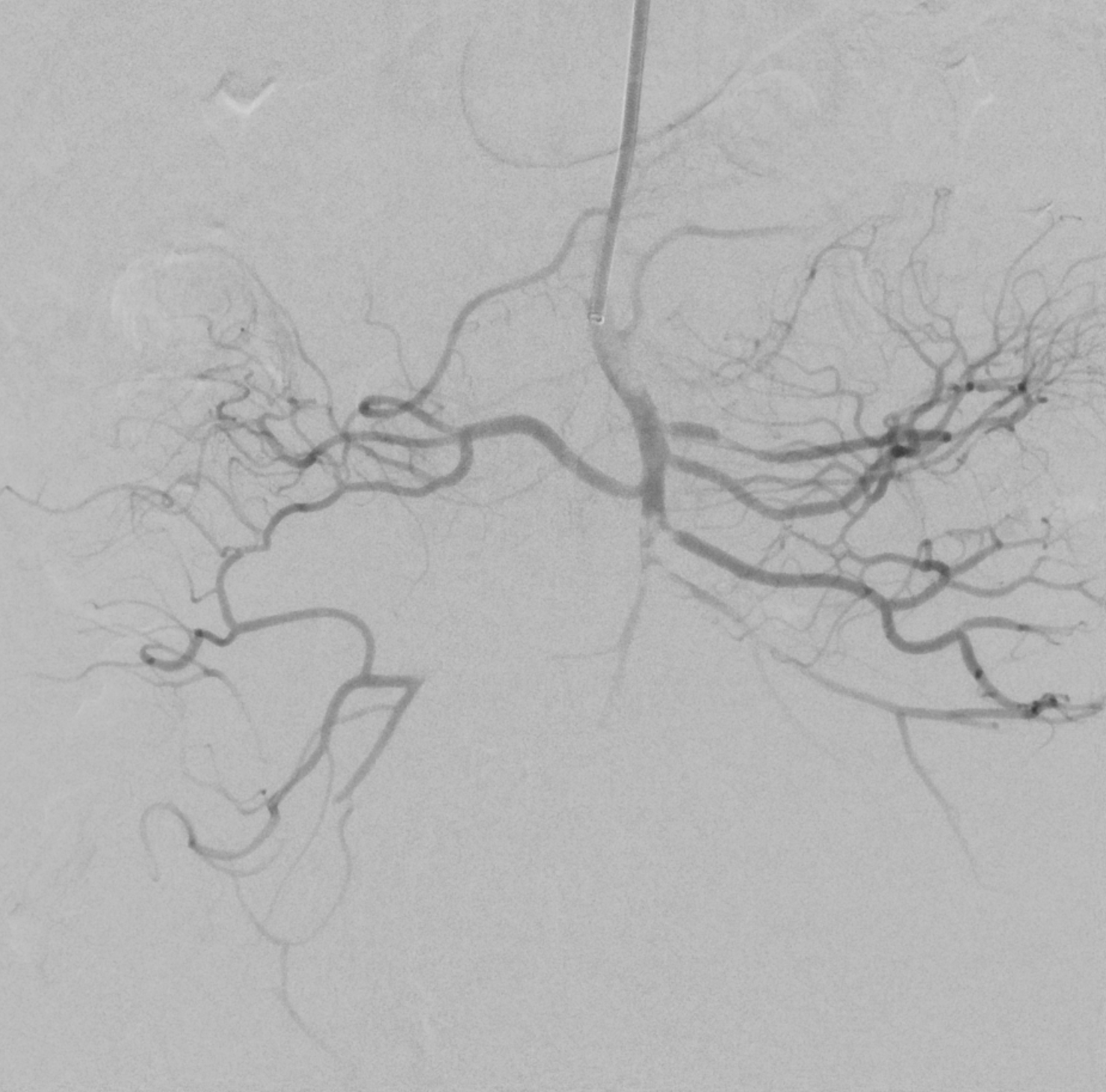

Supplement: Supplementary file 2 — Electronic Supplementary Material [file 330_2024_11115_MOESM2_ESM.zip › Digital Supplementary Material/Angiography/15Angiography.PNG]

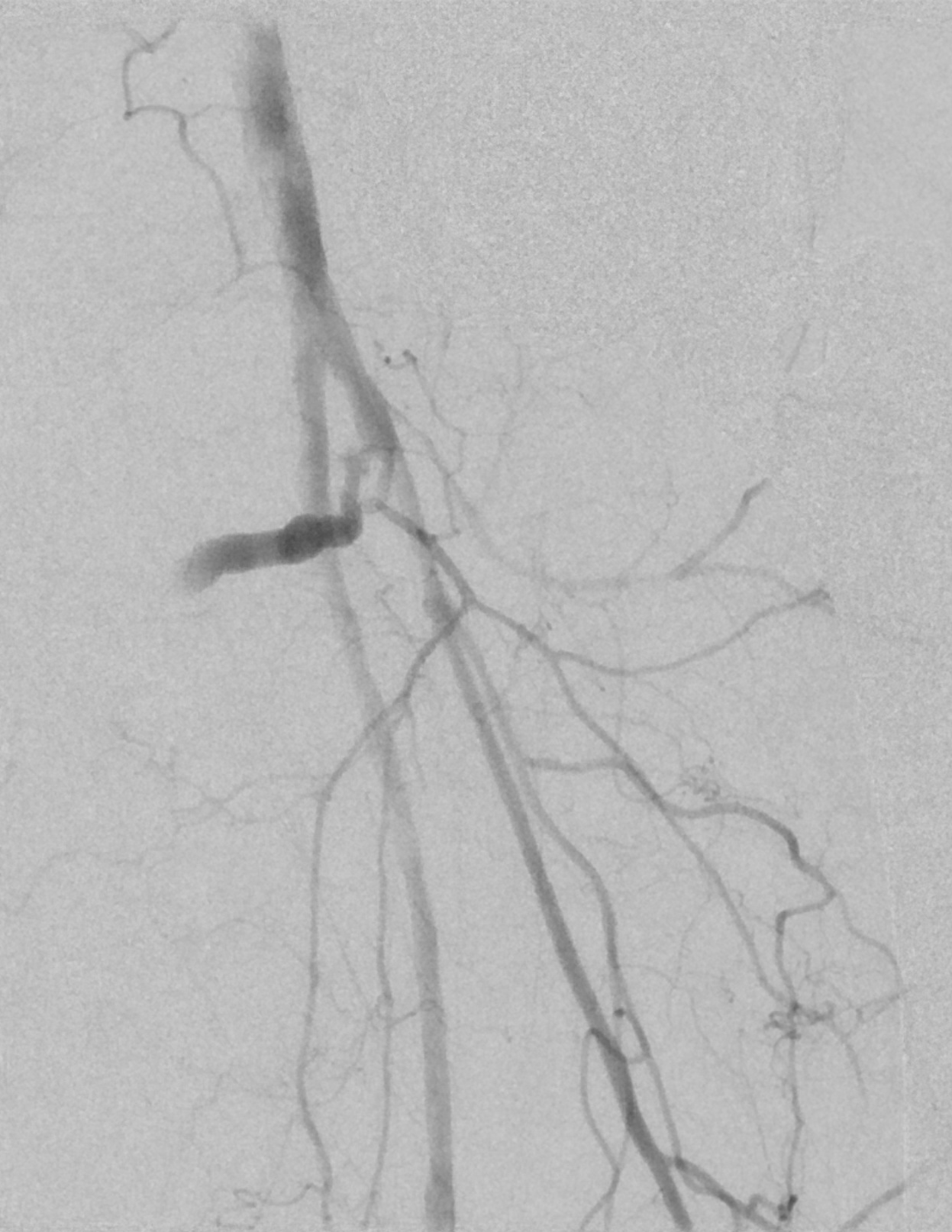

Supplement: Supplementary file 2 — Electronic Supplementary Material [file 330_2024_11115_MOESM2_ESM.zip › Digital Supplementary Material/Angiography/12Angiography.PNG]

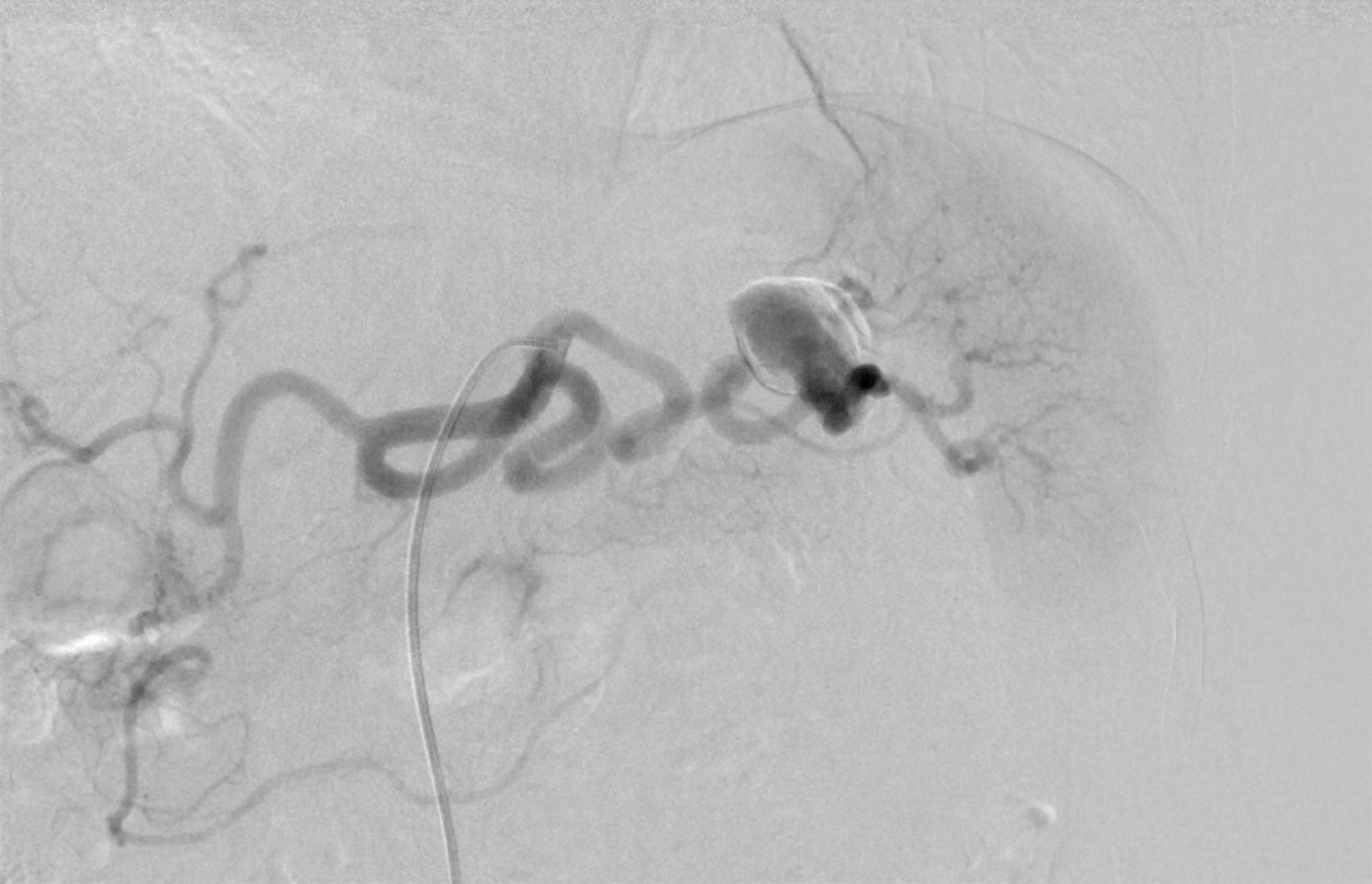

Supplement: Supplementary file 2 — Electronic Supplementary Material [file 330_2024_11115_MOESM2_ESM.zip › Digital Supplementary Material/Angiography/13Angiography.PNG]

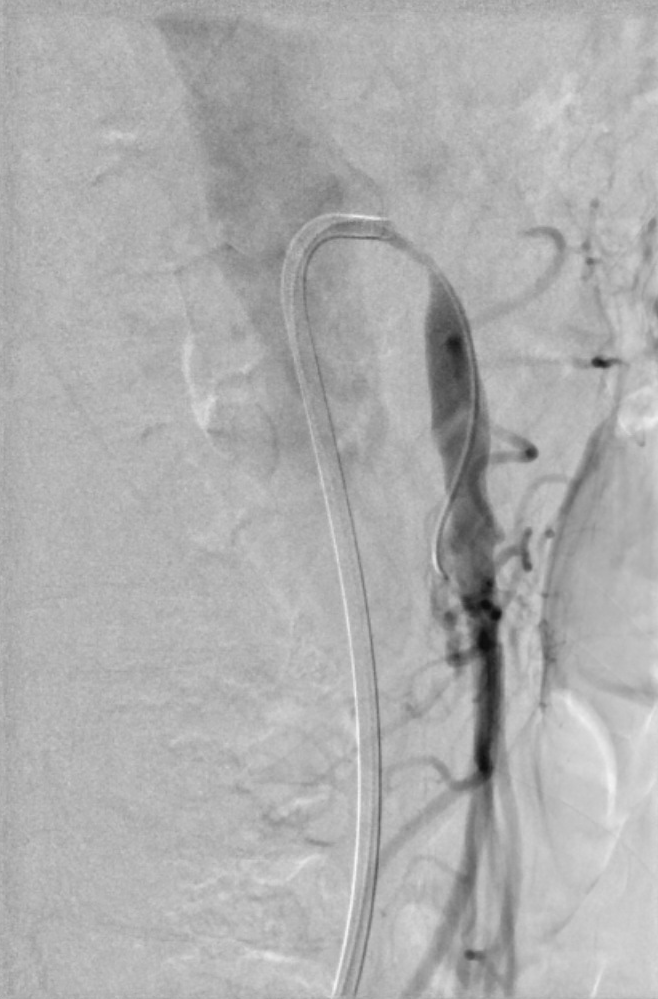

Supplement: Supplementary file 2 — Electronic Supplementary Material [file 330_2024_11115_MOESM2_ESM.zip › Digital Supplementary Material/Angiography/14Angiography.PNG]

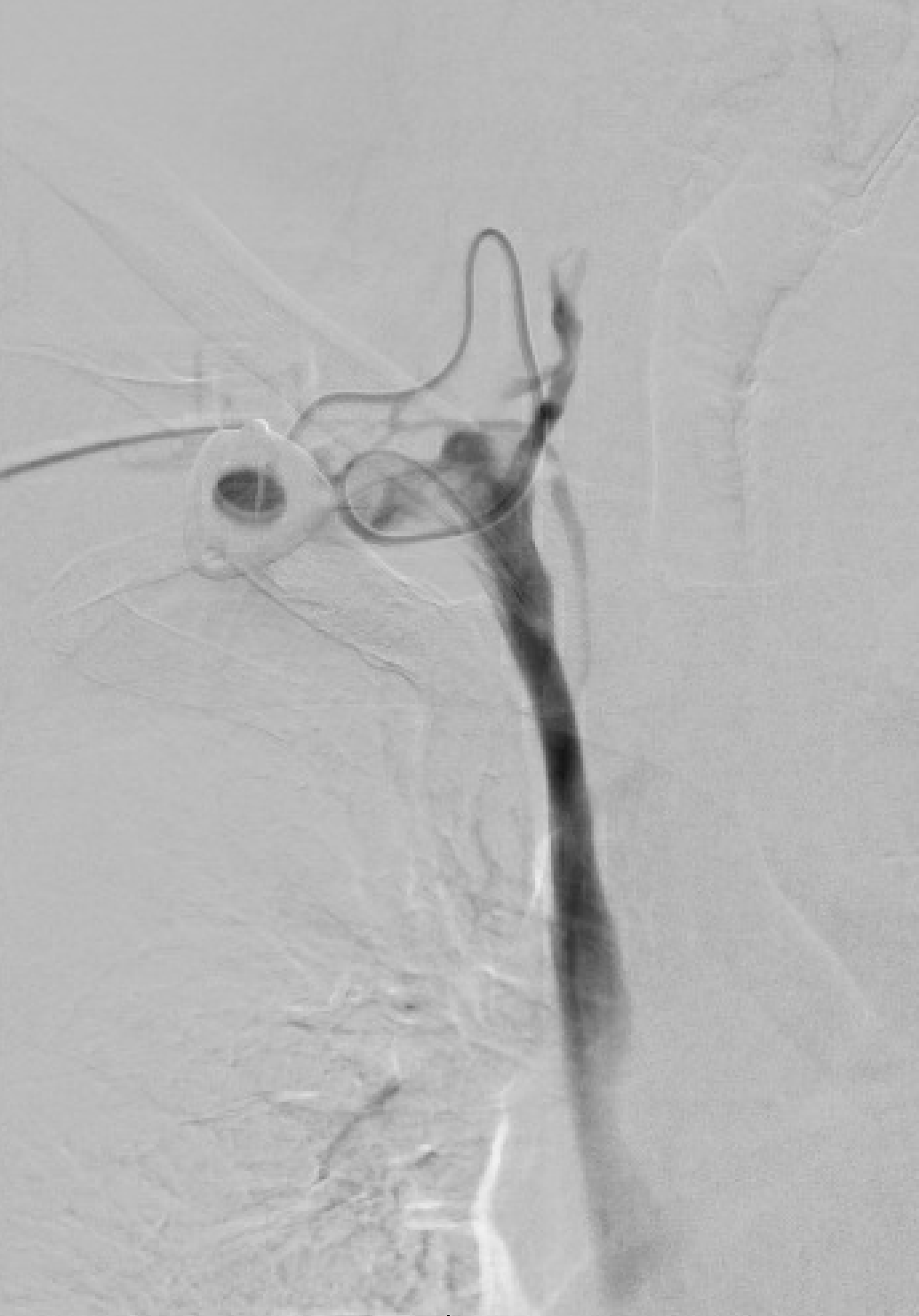

Supplement: Supplementary file 2 — Electronic Supplementary Material [file 330_2024_11115_MOESM2_ESM.zip › Digital Supplementary Material/Angiography/20Angiography.PNG]

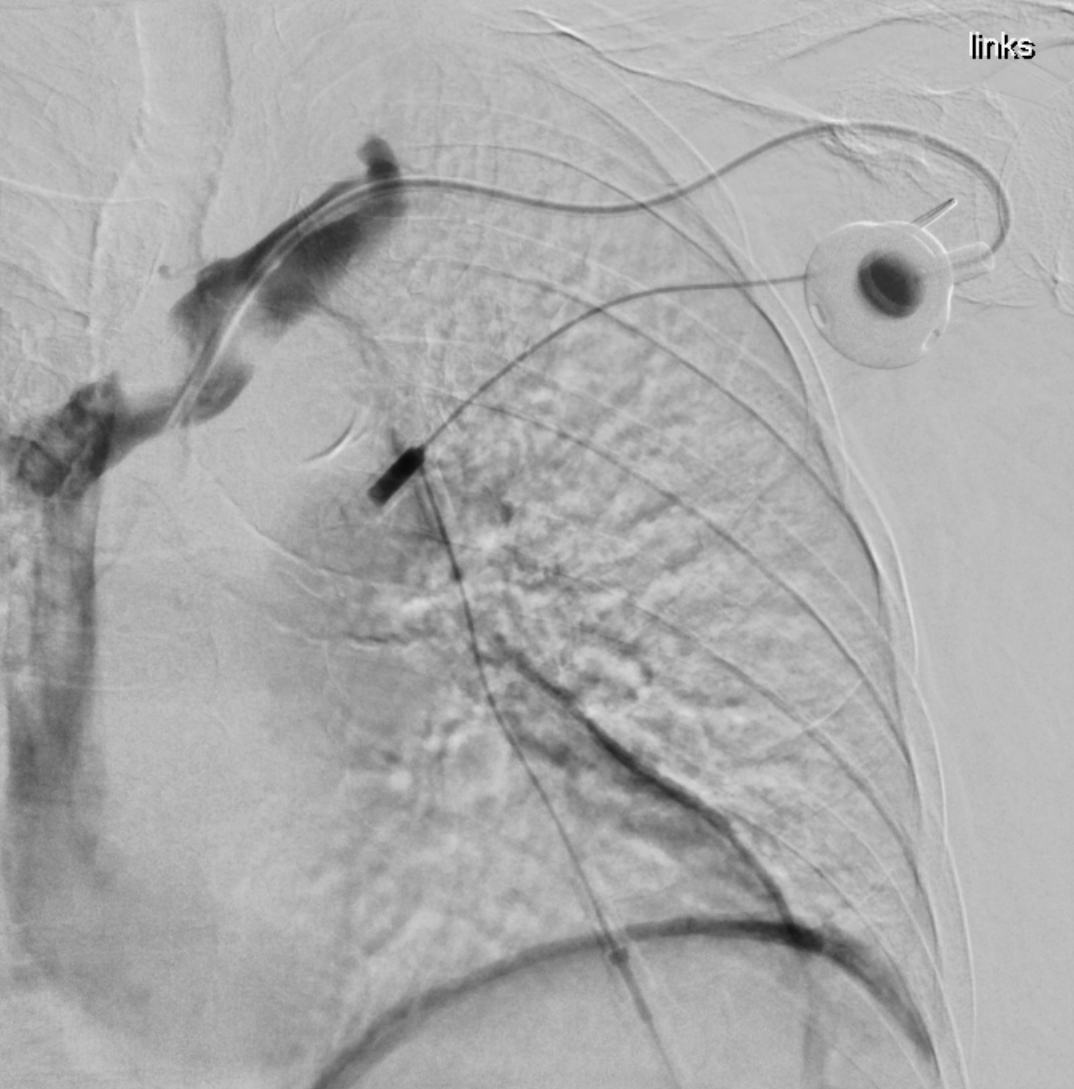

Supplement: Supplementary file 2 — Electronic Supplementary Material [file 330_2024_11115_MOESM2_ESM.zip › Digital Supplementary Material/Angiography/5Angiography.PNG]

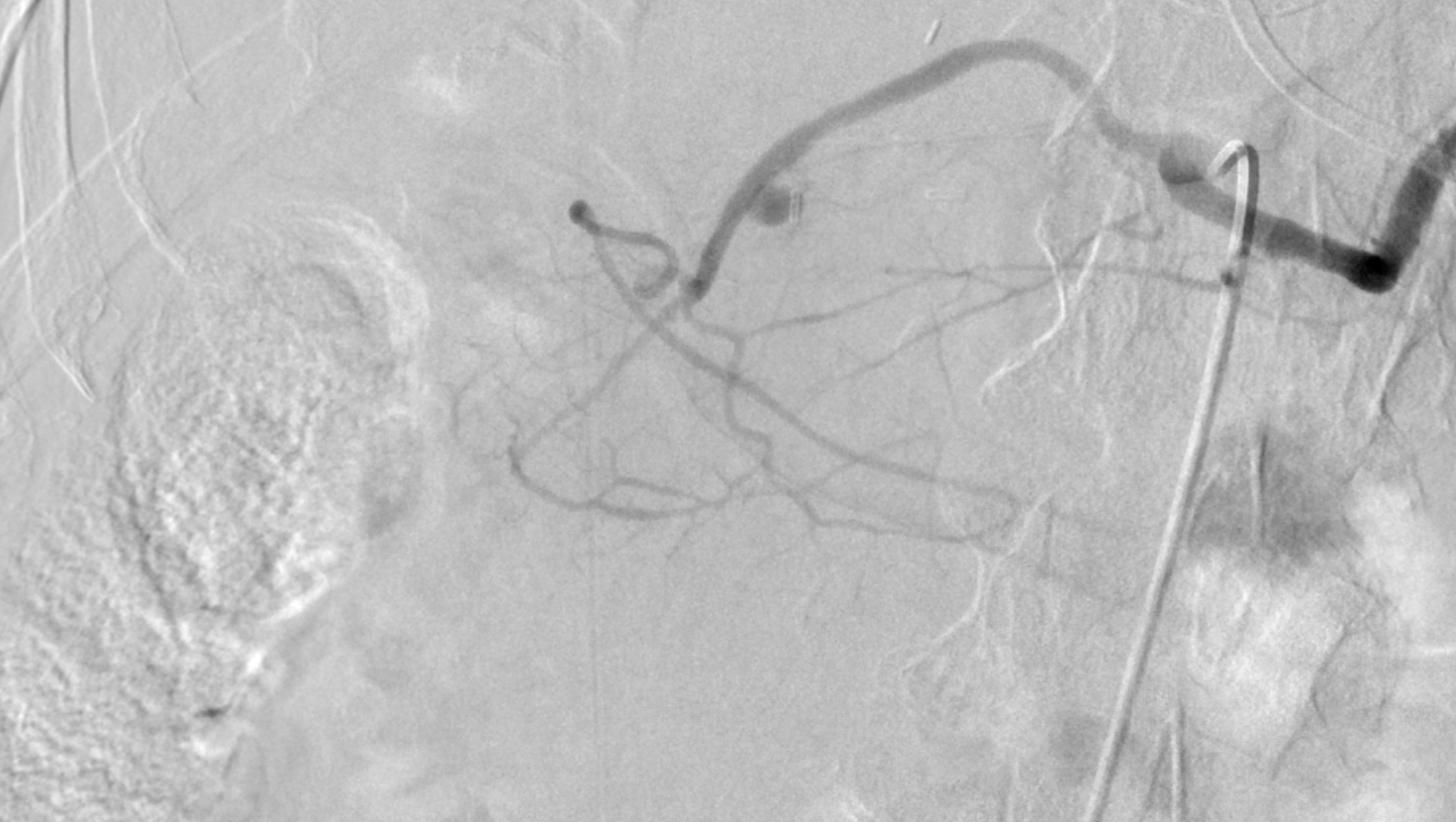

Supplement: Supplementary file 2 — Electronic Supplementary Material [file 330_2024_11115_MOESM2_ESM.zip › Digital Supplementary Material/Angiography/2Angiography.PNG]

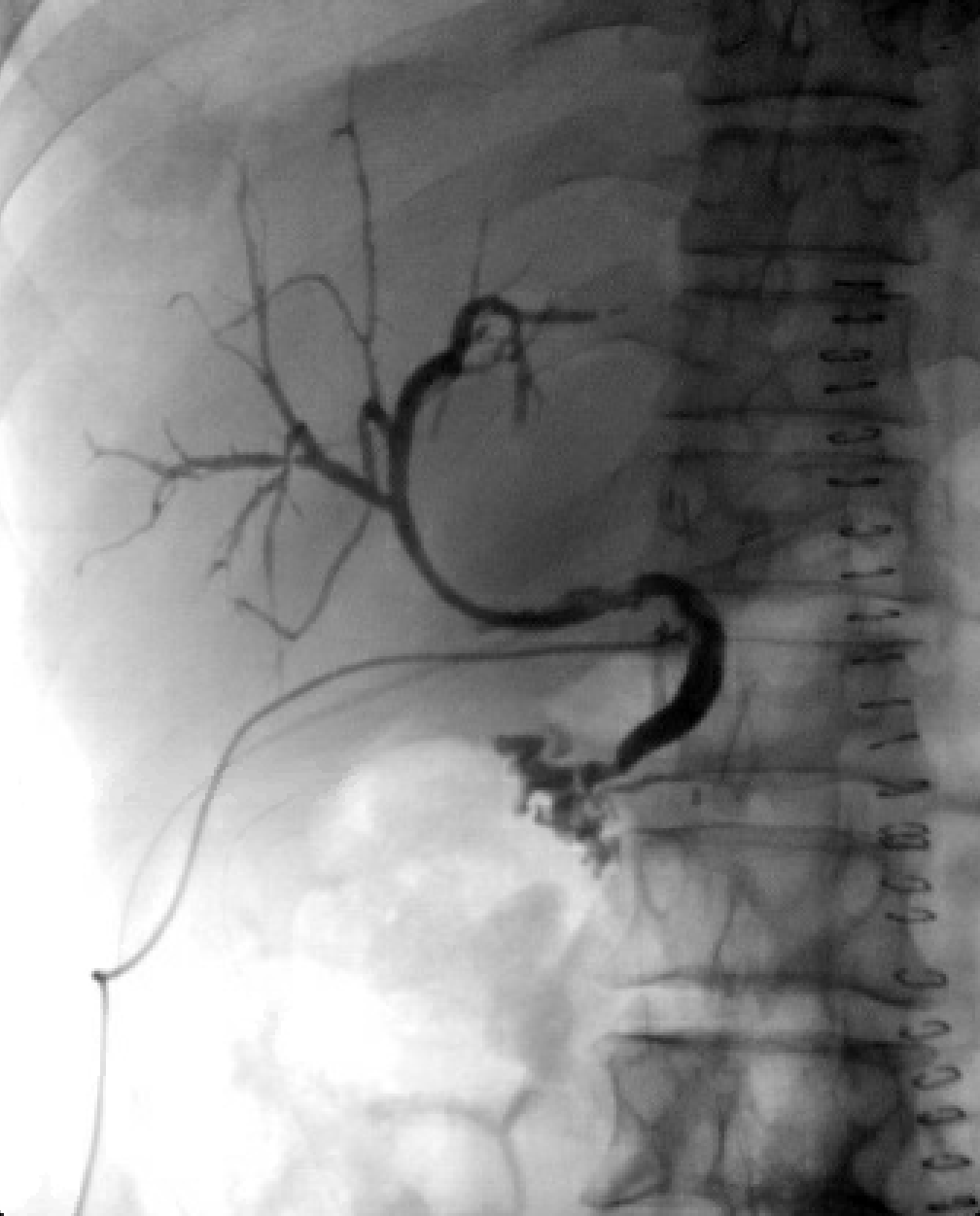

Supplement: Supplementary file 2 — Electronic Supplementary Material [file 330_2024_11115_MOESM2_ESM.zip › Digital Supplementary Material/Angiography/26Angiography.PNG]

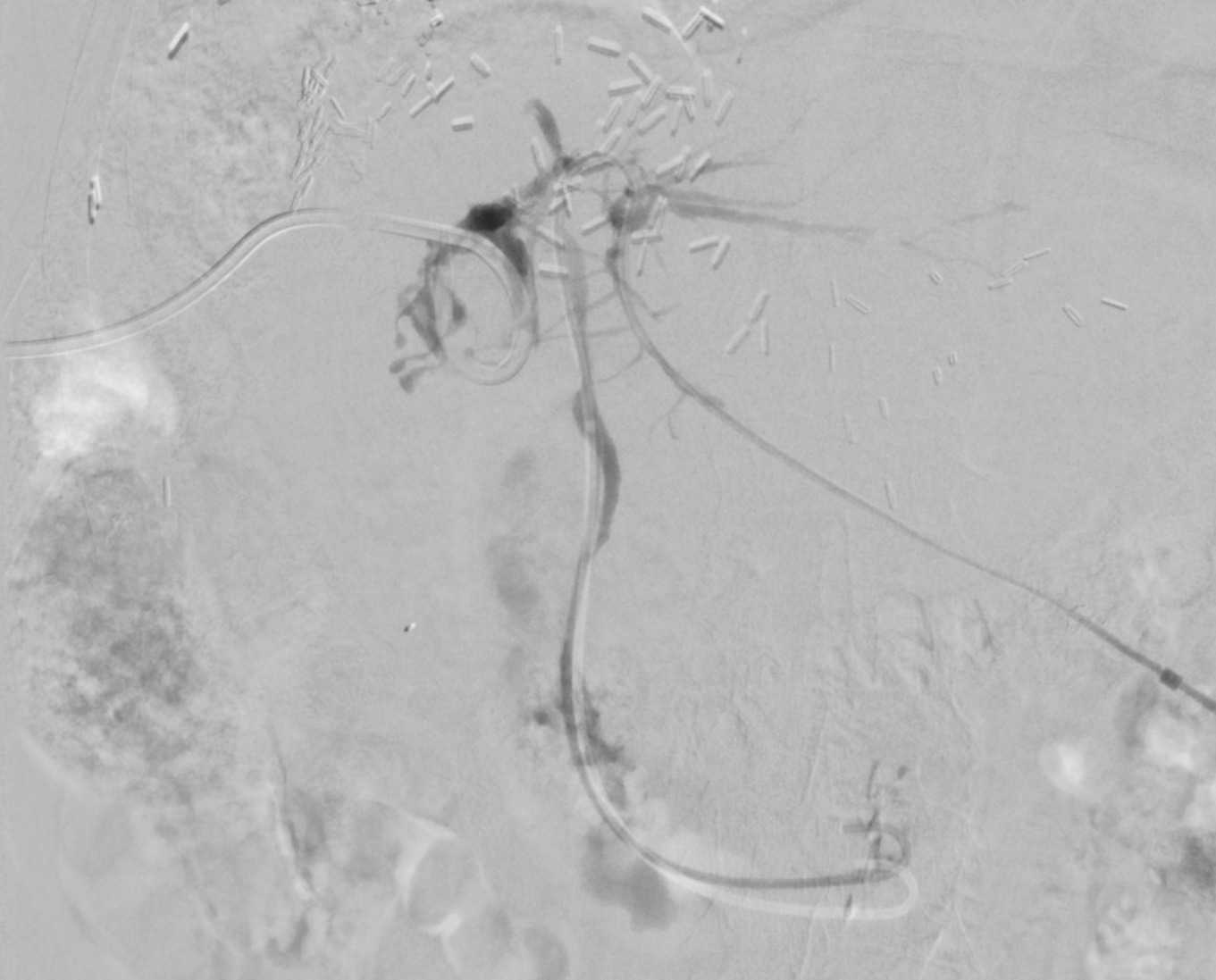

Supplement: Supplementary file 2 — Electronic Supplementary Material [file 330_2024_11115_MOESM2_ESM.zip › Digital Supplementary Material/Angiography/3Angiography.PNG]

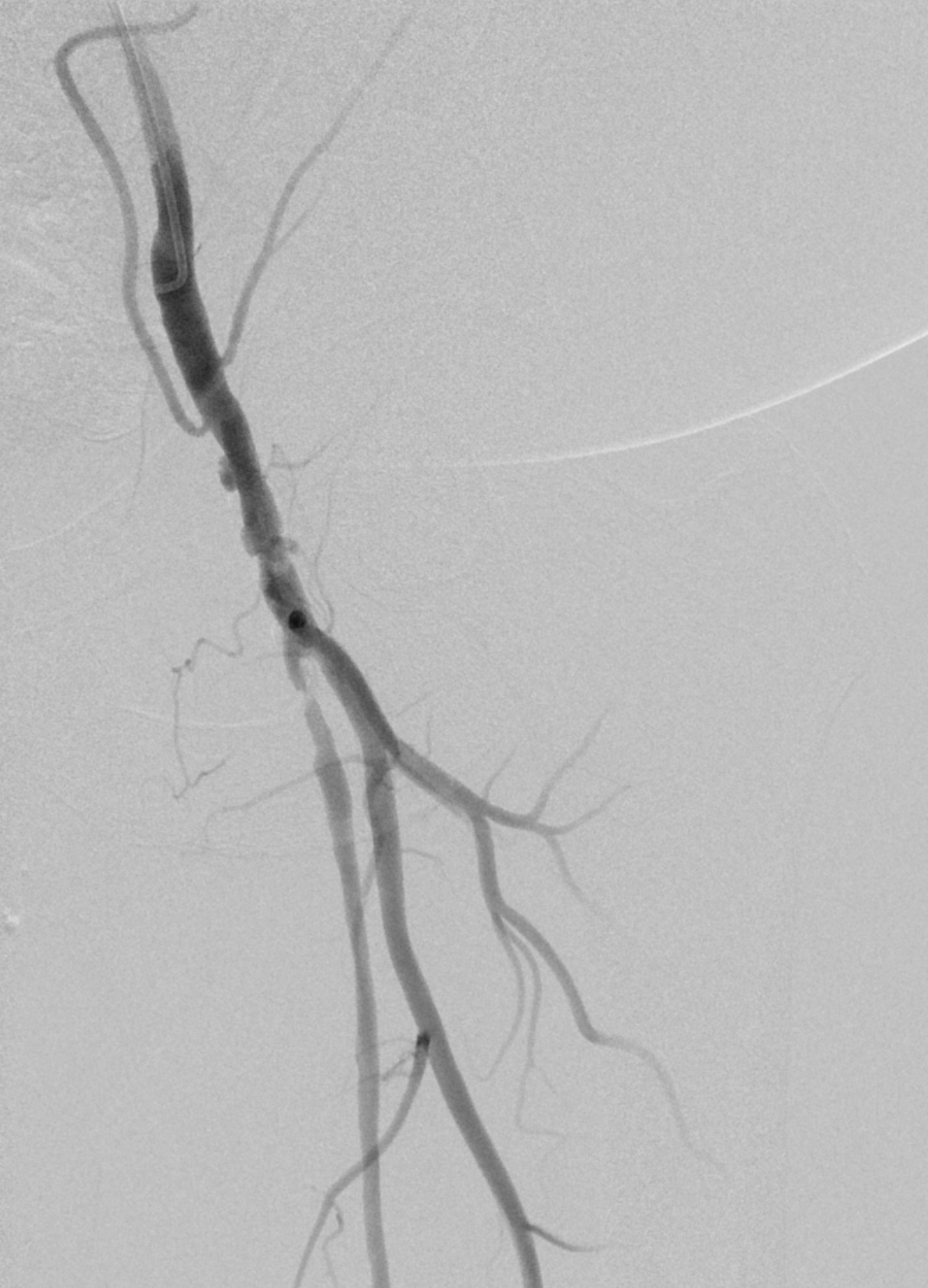

Supplement: Supplementary file 2 — Electronic Supplementary Material [file 330_2024_11115_MOESM2_ESM.zip › Digital Supplementary Material/Angiography/4Angiography.PNG]

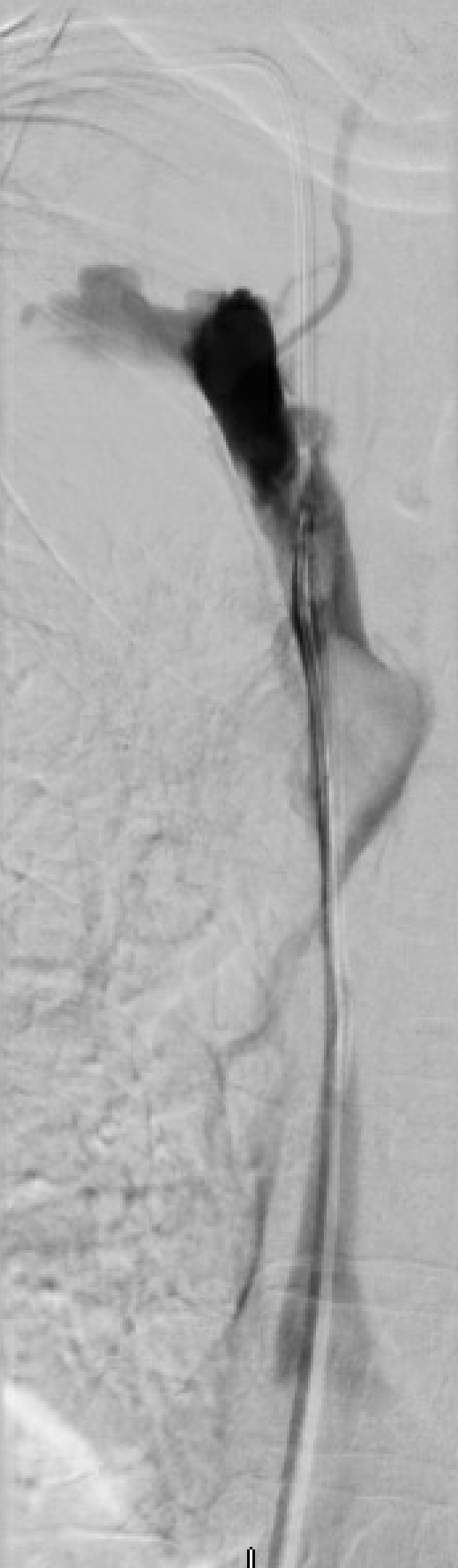

Supplement: Supplementary file 2 — Electronic Supplementary Material [file 330_2024_11115_MOESM2_ESM.zip › Digital Supplementary Material/Angiography/21Angiography.PNG]

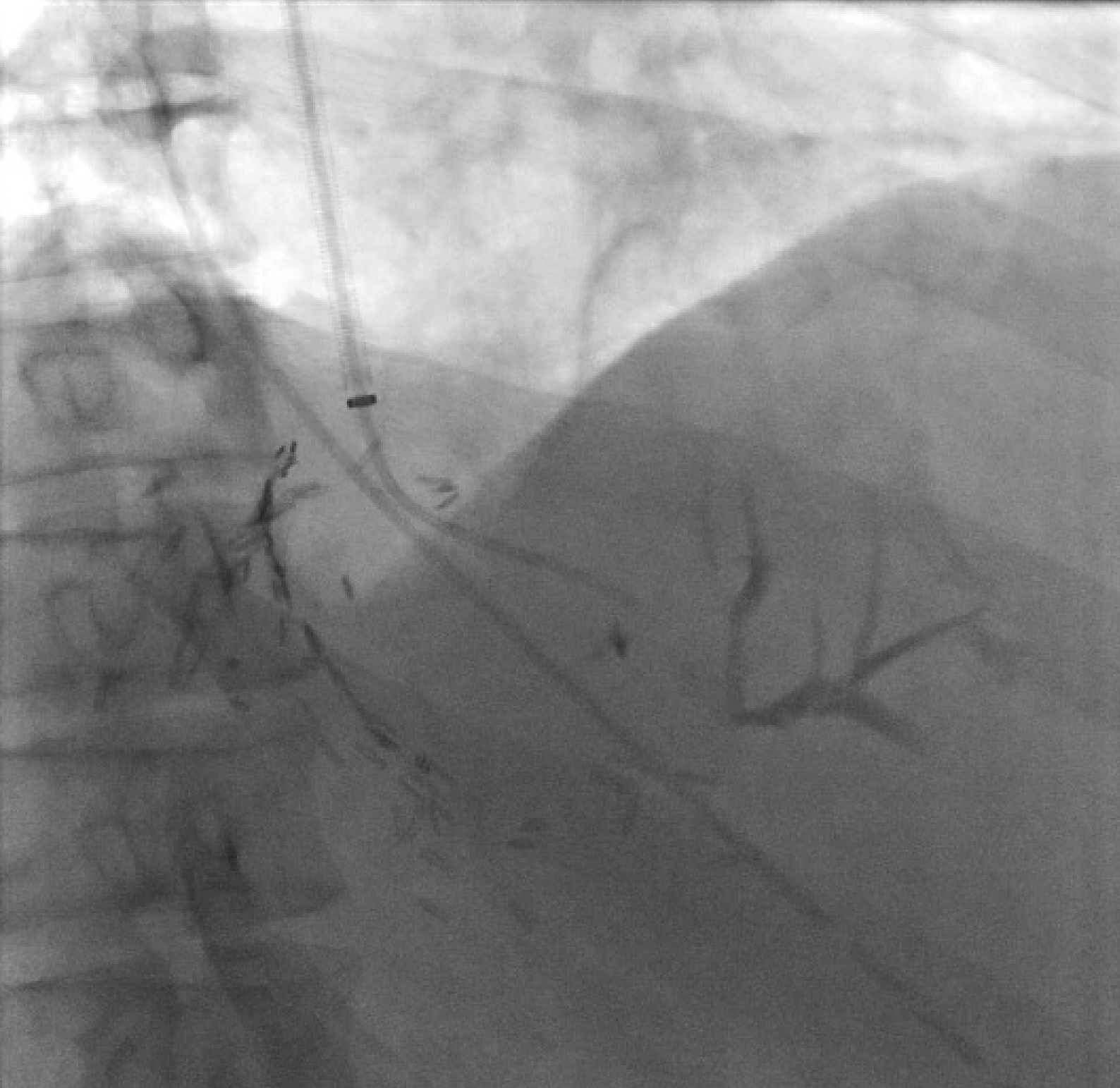

Supplement: Supplementary file 2 — Electronic Supplementary Material [file 330_2024_11115_MOESM2_ESM.zip › Digital Supplementary Material/Angiography/23Angiography.PNG]

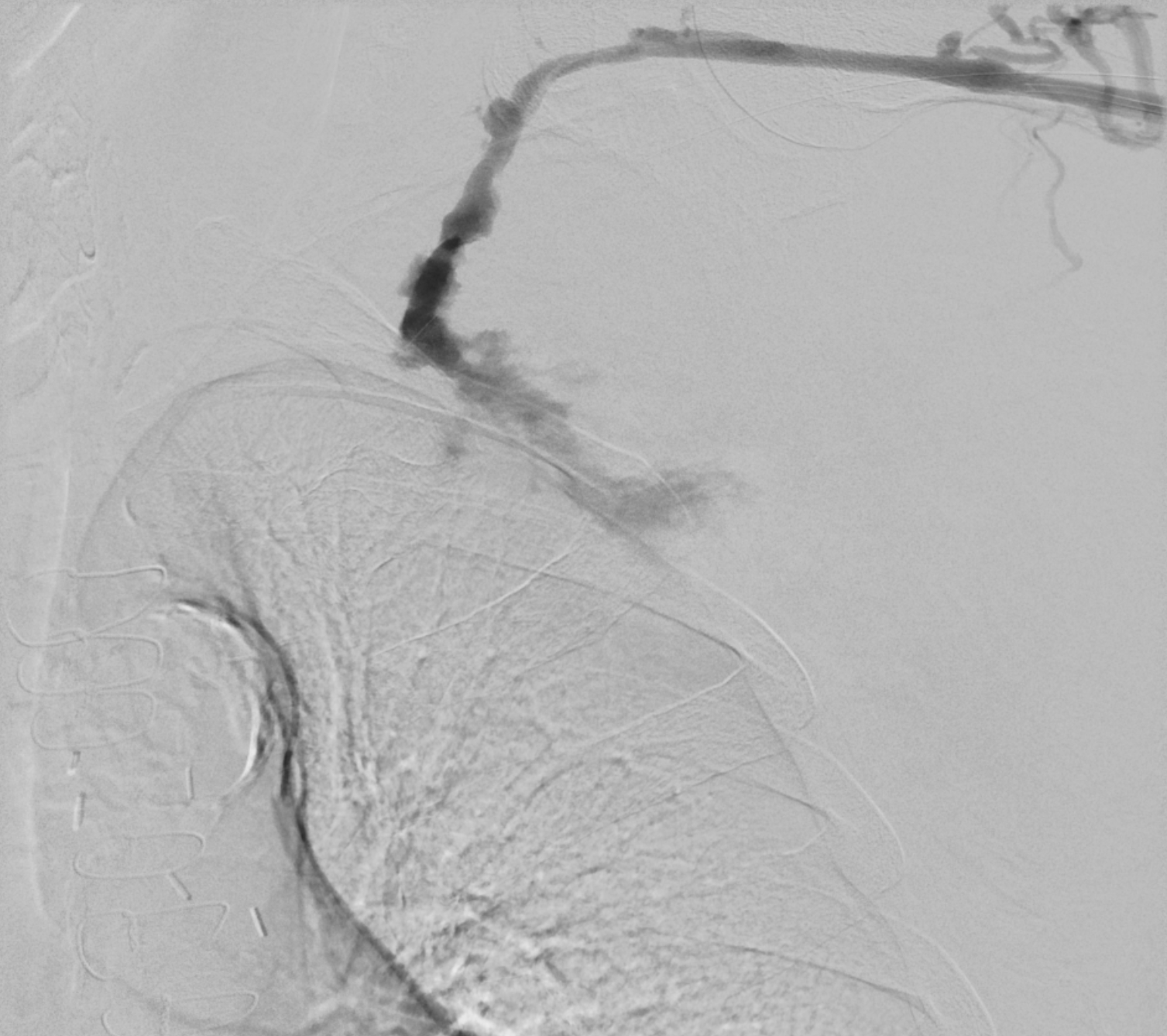

Supplement: Supplementary file 2 — Electronic Supplementary Material [file 330_2024_11115_MOESM2_ESM.zip › Digital Supplementary Material/Angiography/6Angiography.PNG]

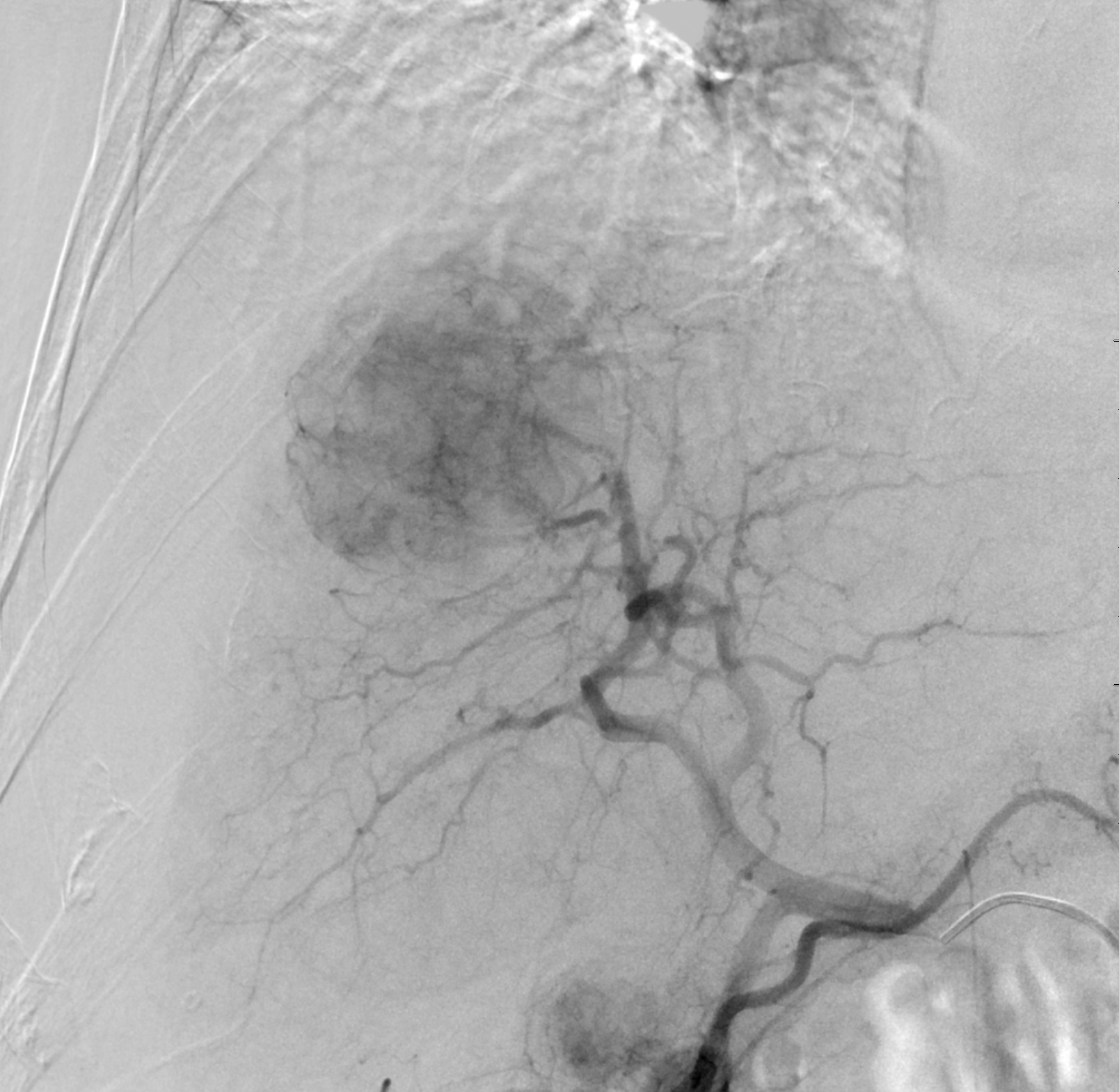

Supplement: Supplementary file 2 — Electronic Supplementary Material [file 330_2024_11115_MOESM2_ESM.zip › Digital Supplementary Material/Angiography/1Angiography.PNG]

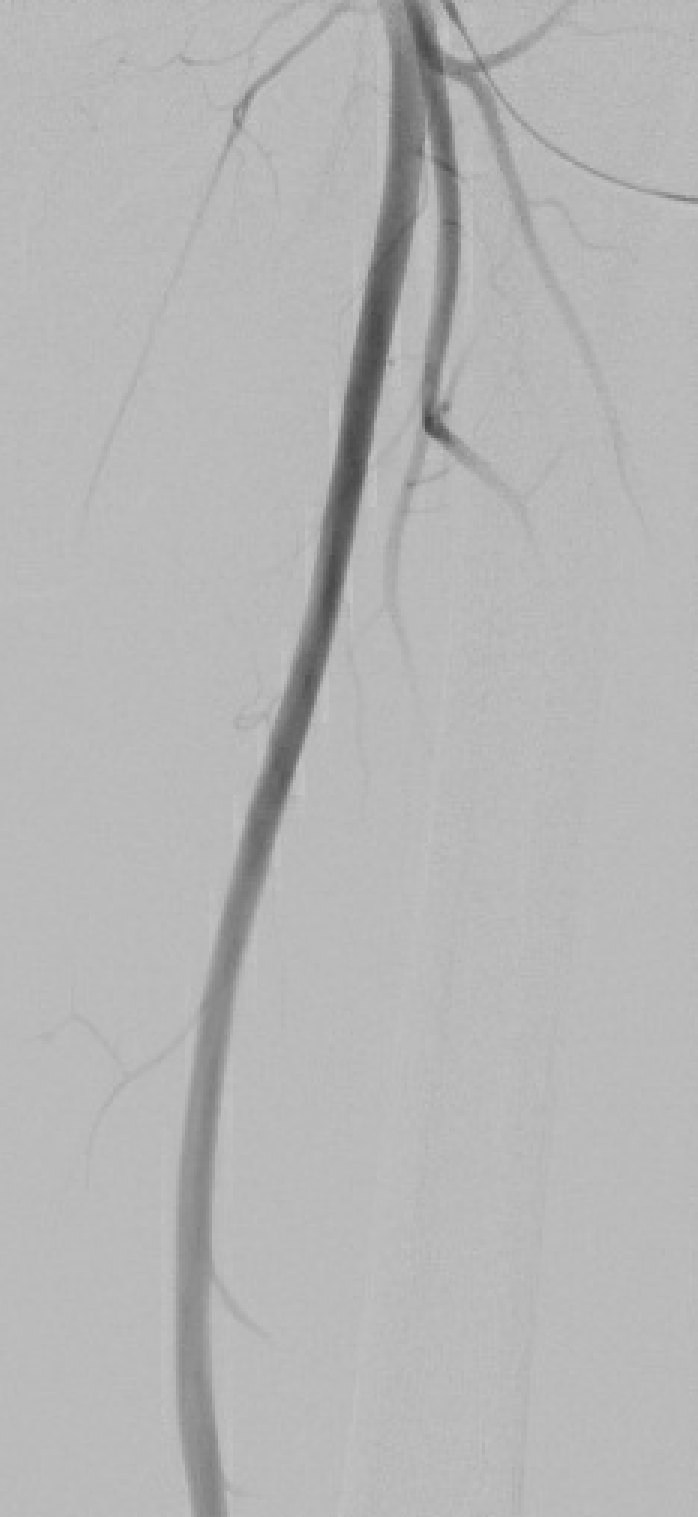

Supplement: Supplementary file 2 — Electronic Supplementary Material [file 330_2024_11115_MOESM2_ESM.zip › Digital Supplementary Material/Angiography/24Angiography.PNG]

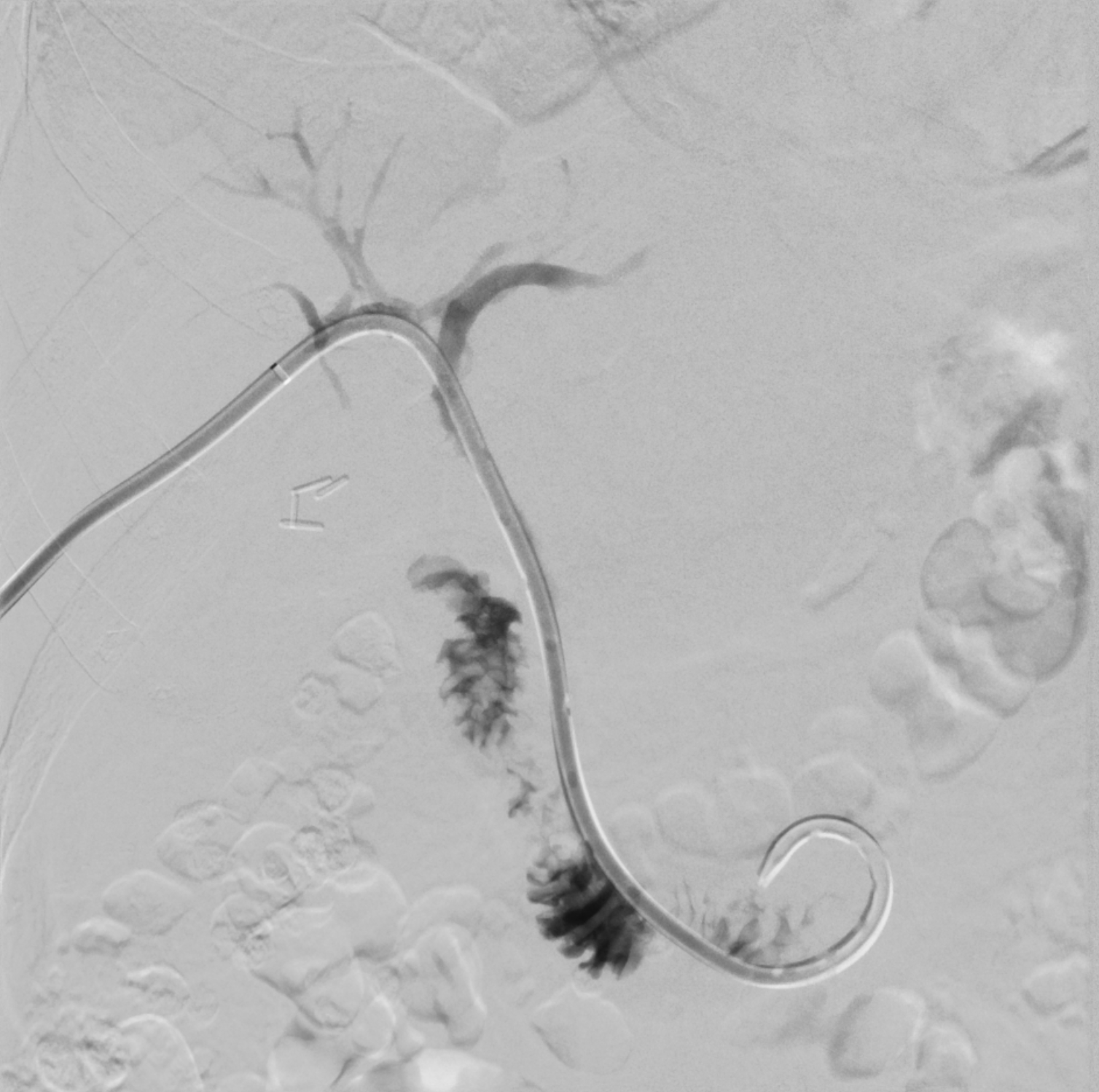

Supplement: Supplementary file 2 — Electronic Supplementary Material [file 330_2024_11115_MOESM2_ESM.zip › Digital Supplementary Material/Angiography/9Angiography.PNG]

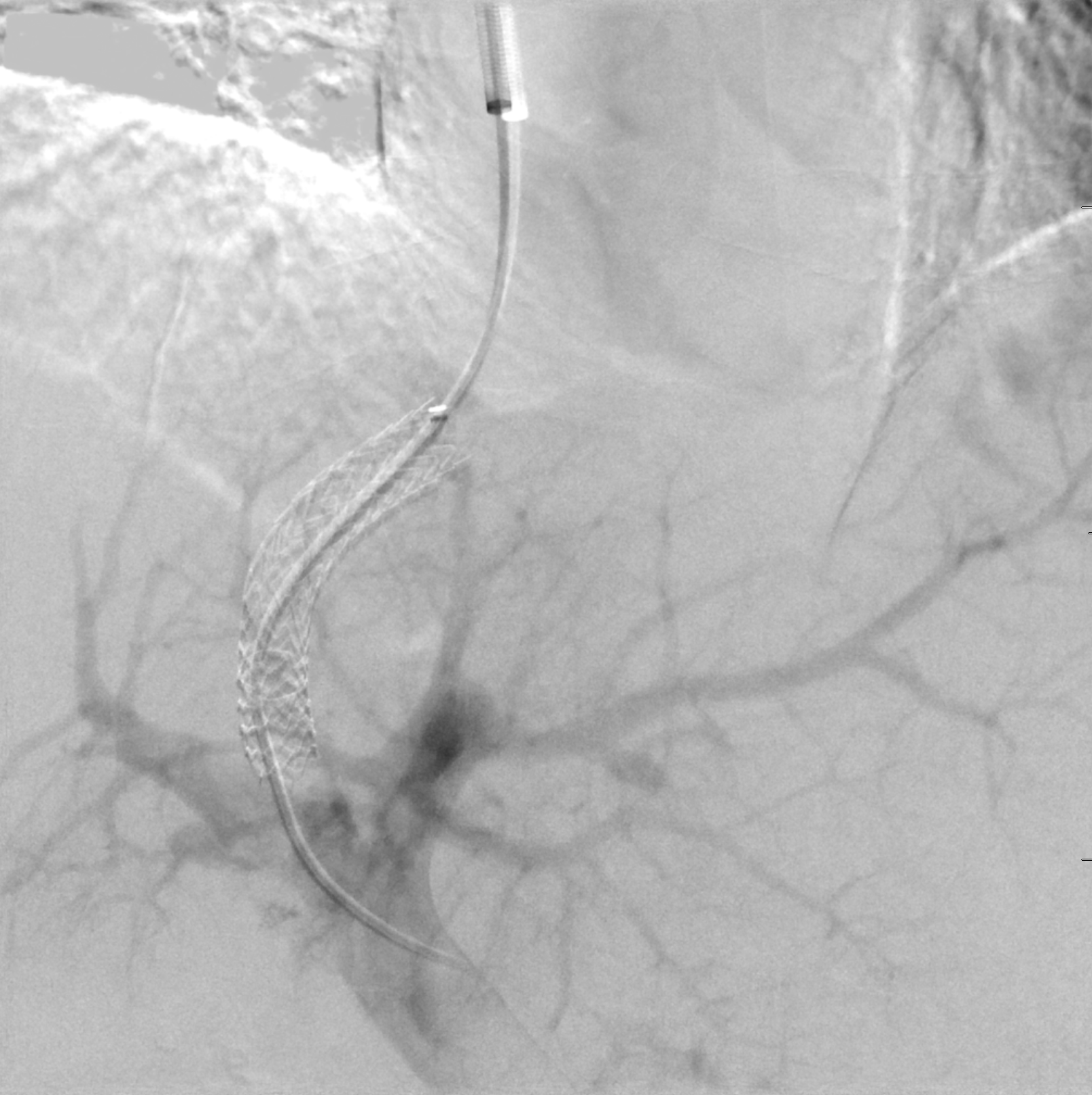

Supplement: Supplementary file 2 — Electronic Supplementary Material [file 330_2024_11115_MOESM2_ESM.zip › Digital Supplementary Material/Angiography/8Angiography.PNG]

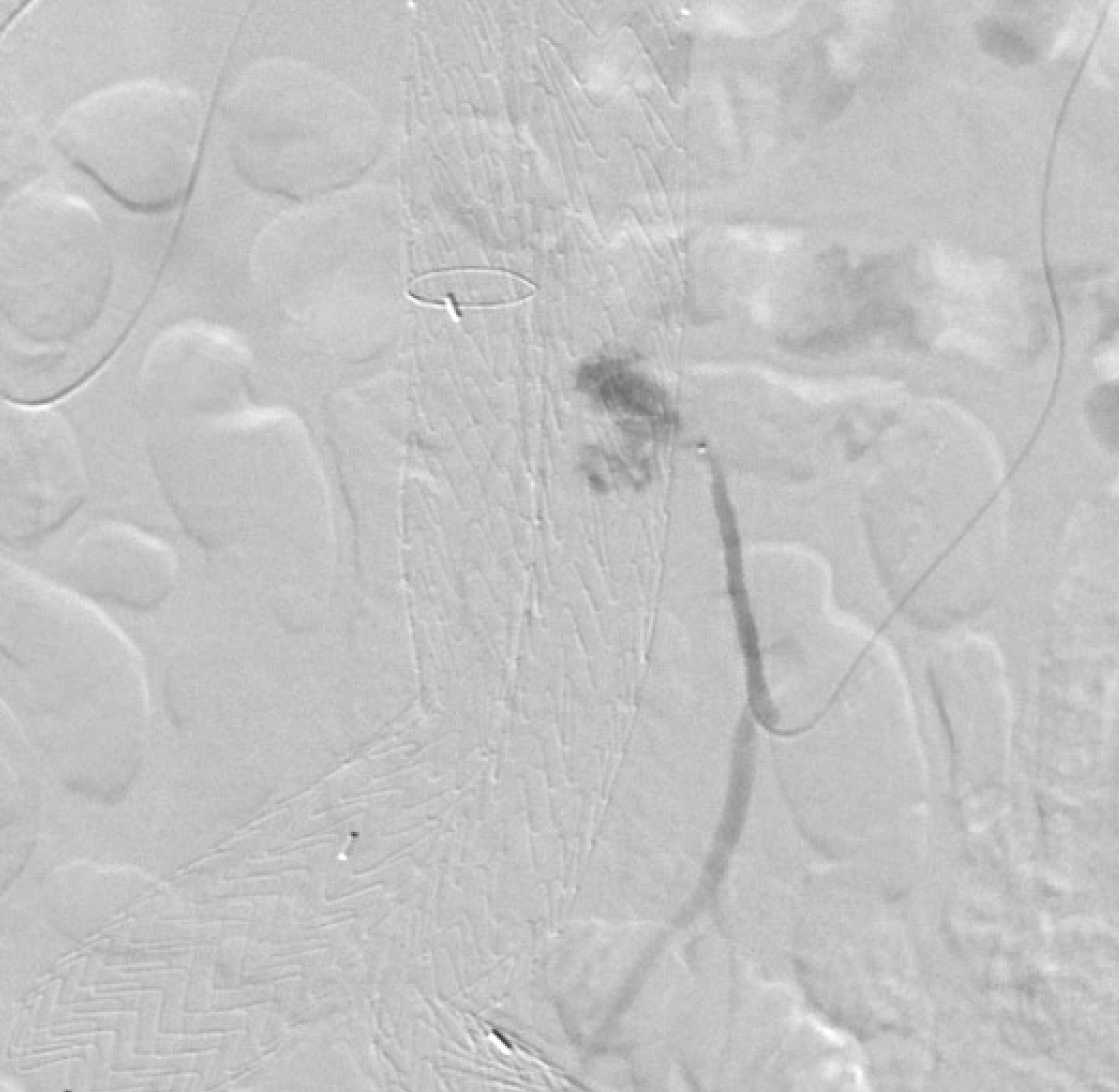

Supplement: Supplementary file 2 — Electronic Supplementary Material [file 330_2024_11115_MOESM2_ESM.zip › Digital Supplementary Material/Angiography/25Angiography.PNG]

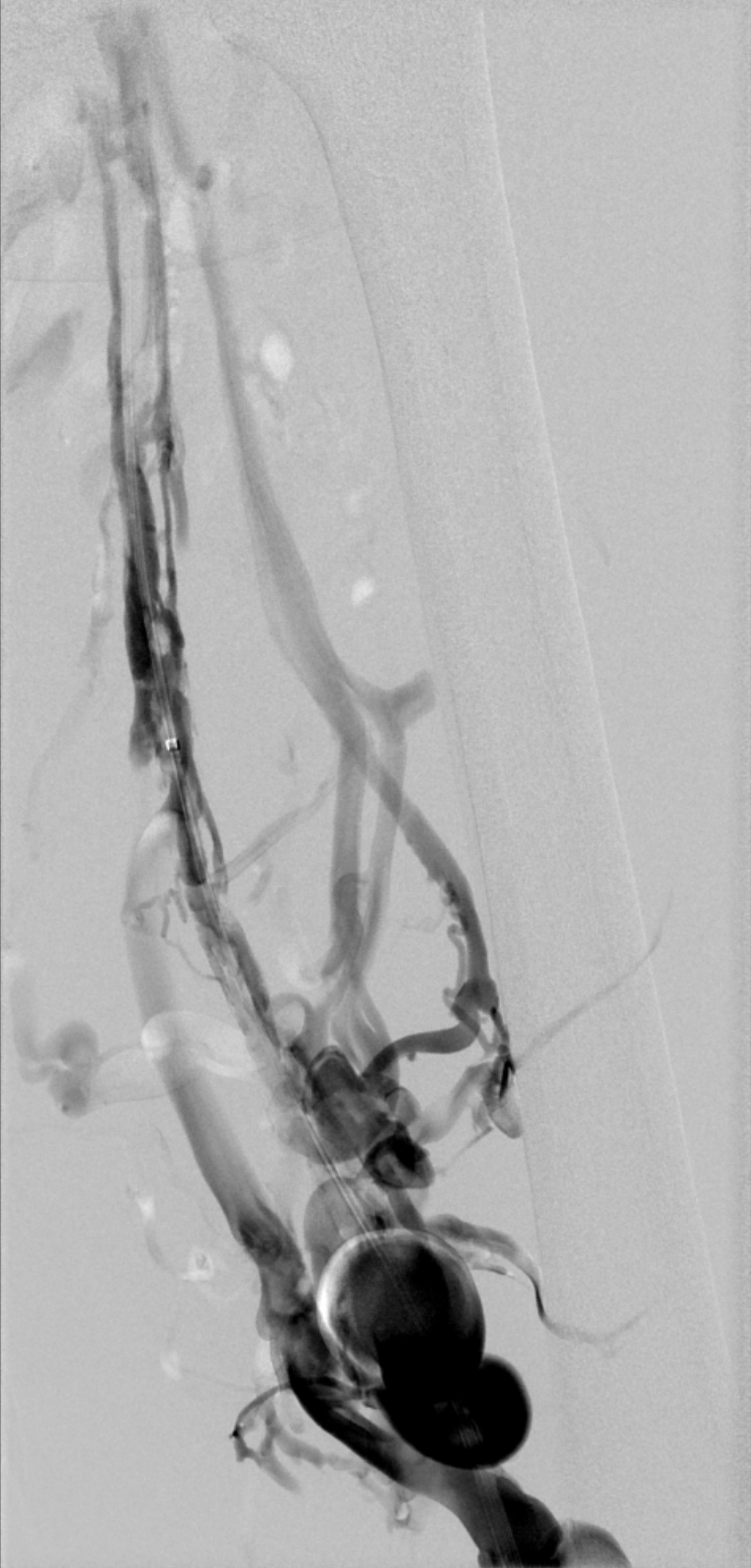

Supplement: Supplementary file 2 — Electronic Supplementary Material [file 330_2024_11115_MOESM2_ESM.zip › Digital Supplementary Material/Angiography/7Angiography.PNG]

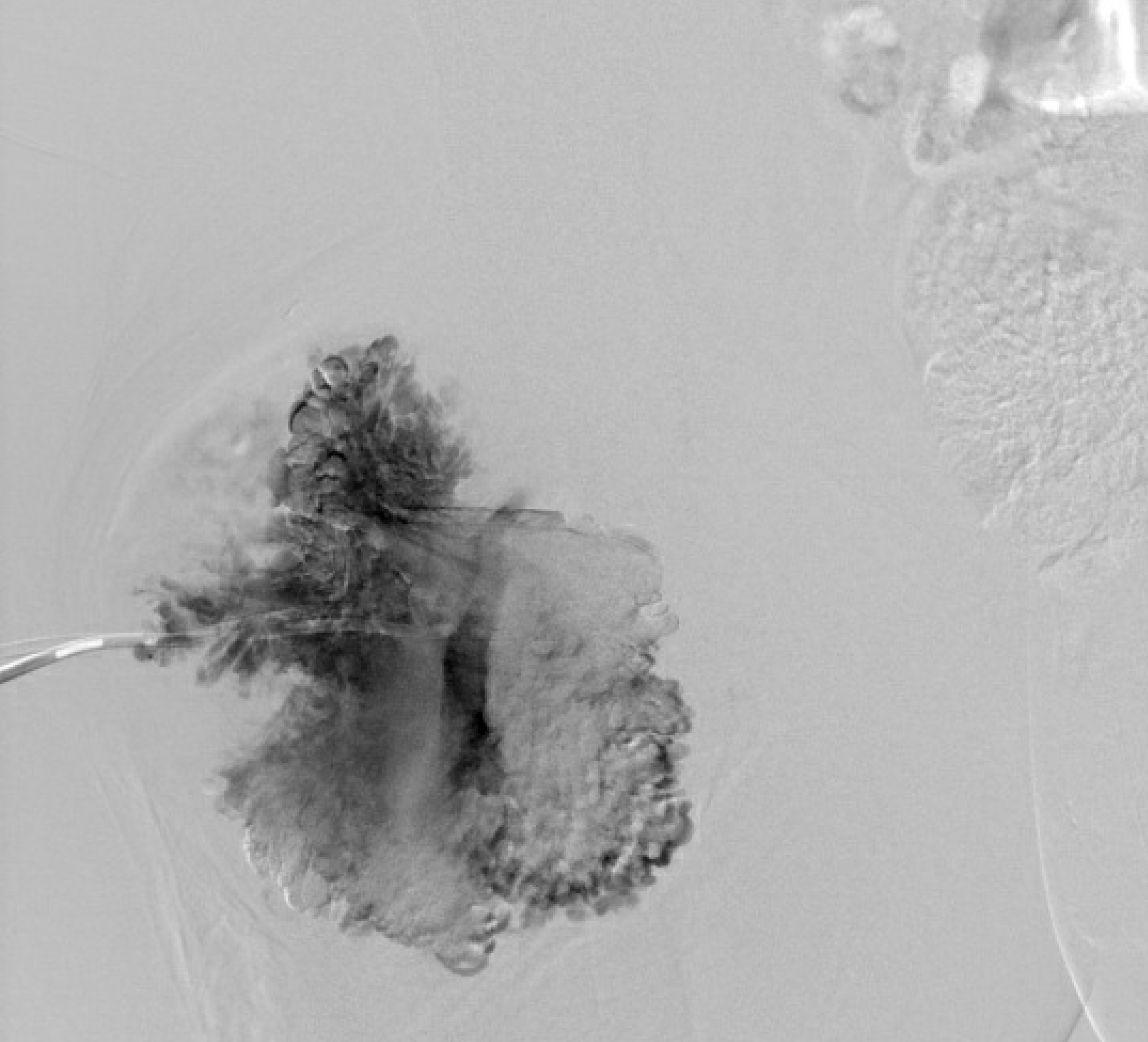

Supplement: Supplementary file 2 — Electronic Supplementary Material [file 330_2024_11115_MOESM2_ESM.zip › Digital Supplementary Material/Angiography/22Angiography.PNG]

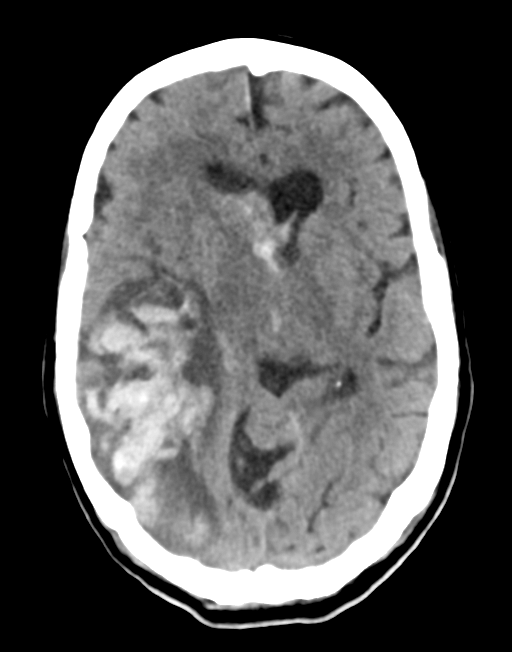

Supplement: Supplementary file 2 — Electronic Supplementary Material [file 330_2024_11115_MOESM2_ESM.zip › Digital Supplementary Material/Computed Tomography/31Computed Tomography.PNG]

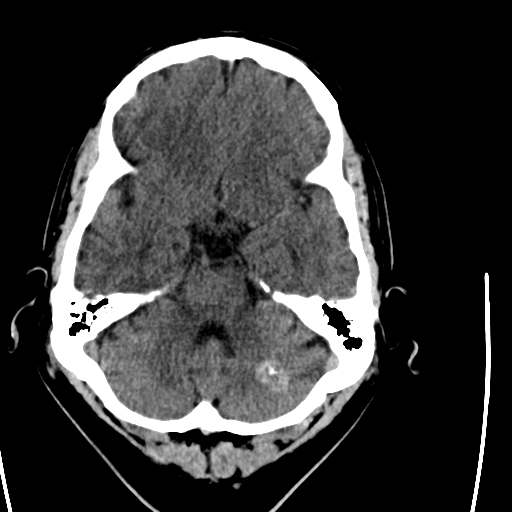

Supplement: Supplementary file 2 — Electronic Supplementary Material [file 330_2024_11115_MOESM2_ESM.zip › Digital Supplementary Material/Computed Tomography/44Computed Tomography.PNG]

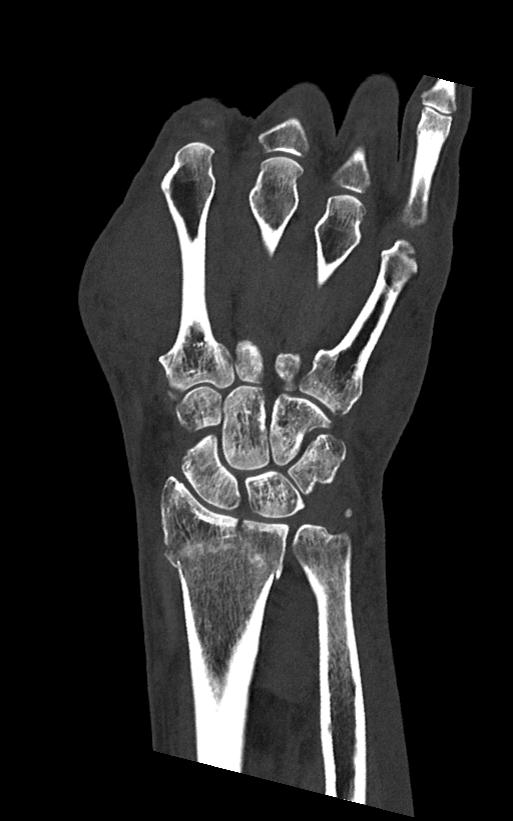

Supplement: Supplementary file 2 — Electronic Supplementary Material [file 330_2024_11115_MOESM2_ESM.zip › Digital Supplementary Material/Computed Tomography/36Computed Tomography.PNG]

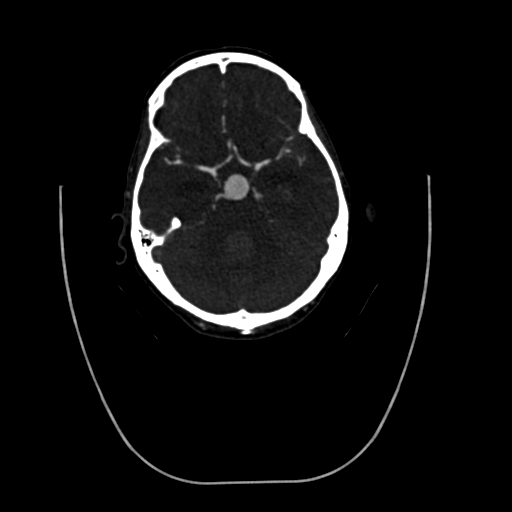

Supplement: Supplementary file 2 — Electronic Supplementary Material [file 330_2024_11115_MOESM2_ESM.zip › Digital Supplementary Material/Computed Tomography/43Computed Tomography.PNG]

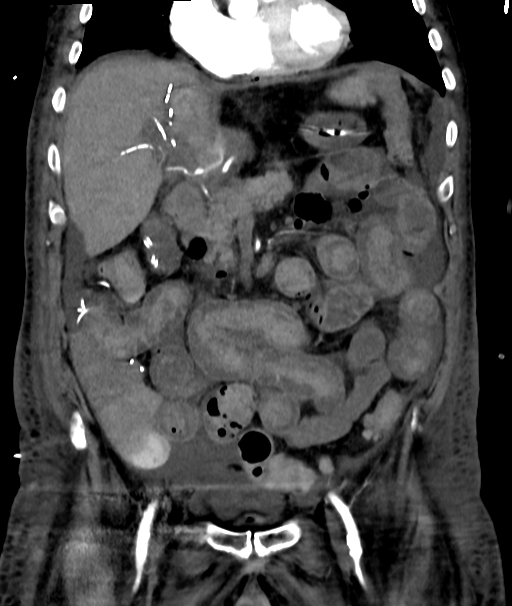

Supplement: Supplementary file 2 — Electronic Supplementary Material [file 330_2024_11115_MOESM2_ESM.zip › Digital Supplementary Material/Computed Tomography/49Computed Tomography.PNG]

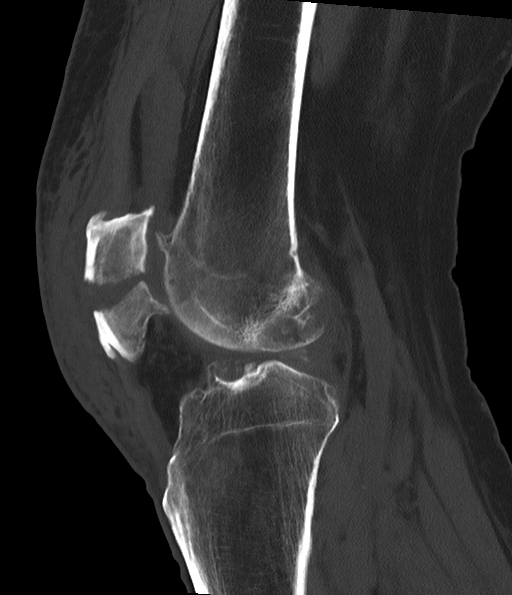

Supplement: Supplementary file 2 — Electronic Supplementary Material [file 330_2024_11115_MOESM2_ESM.zip › Digital Supplementary Material/Computed Tomography/40Computed Tomography.PNG]

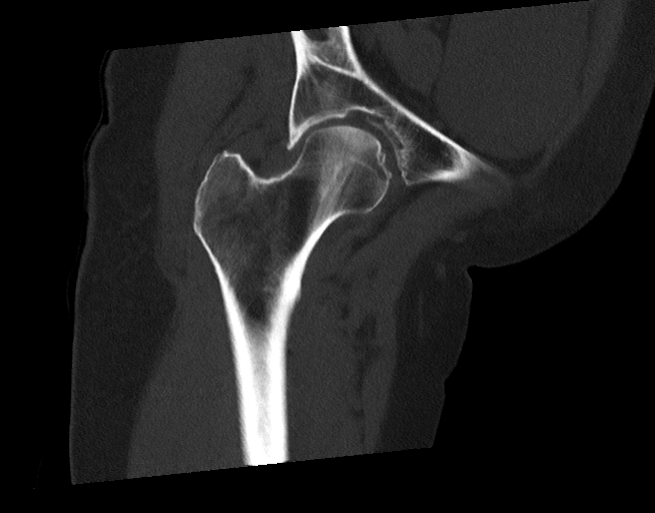

Supplement: Supplementary file 2 — Electronic Supplementary Material [file 330_2024_11115_MOESM2_ESM.zip › Digital Supplementary Material/Computed Tomography/38Computed Tomography.PNG]

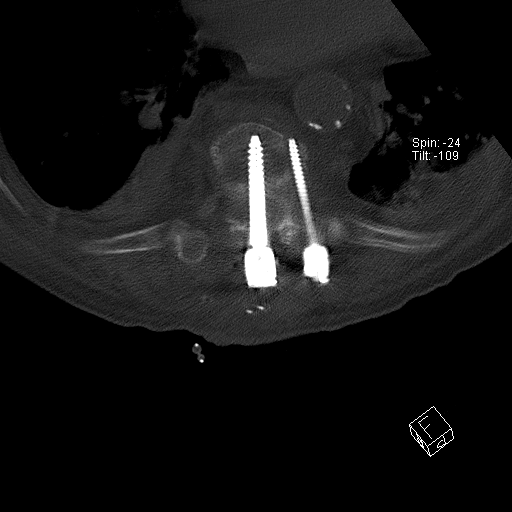

Supplement: Supplementary file 2 — Electronic Supplementary Material [file 330_2024_11115_MOESM2_ESM.zip › Digital Supplementary Material/Computed Tomography/47Computed Tomography.PNG]

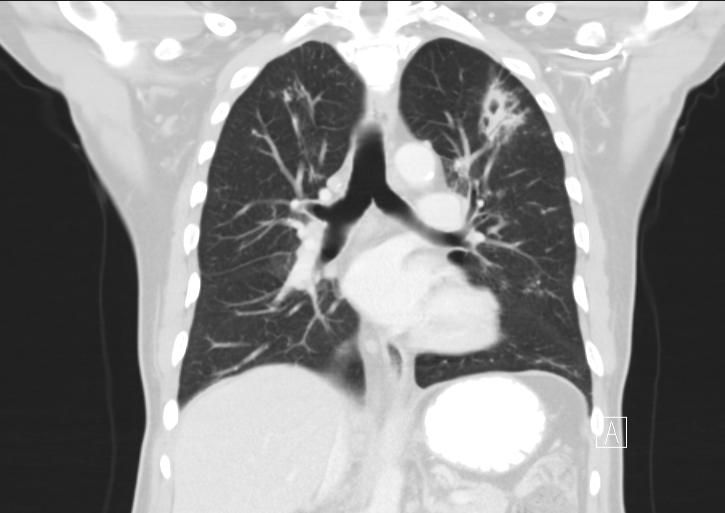

Supplement: Supplementary file 2 — Electronic Supplementary Material [file 330_2024_11115_MOESM2_ESM.zip › Digital Supplementary Material/Computed Tomography/32Computed Tomography.PNG]

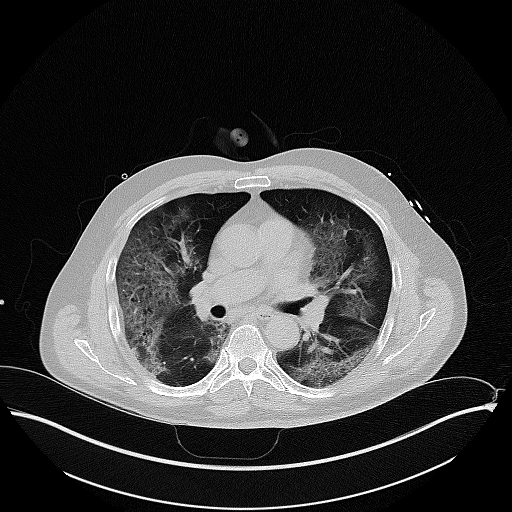

Supplement: Supplementary file 2 — Electronic Supplementary Material [file 330_2024_11115_MOESM2_ESM.zip › Digital Supplementary Material/Computed Tomography/46Computed Tomography.PNG]

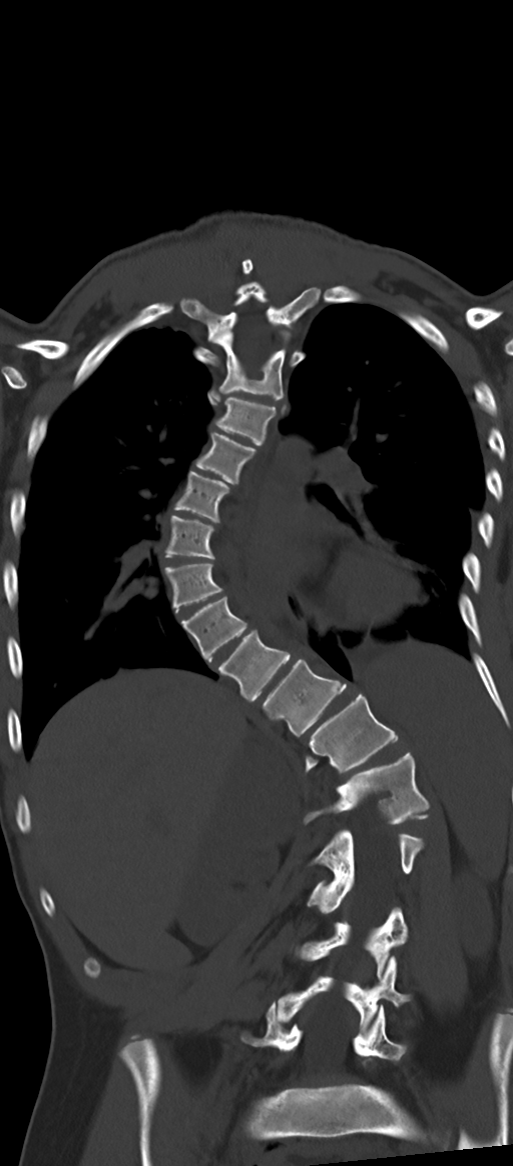

Supplement: Supplementary file 2 — Electronic Supplementary Material [file 330_2024_11115_MOESM2_ESM.zip › Digital Supplementary Material/Computed Tomography/33Computed Tomography.PNG]

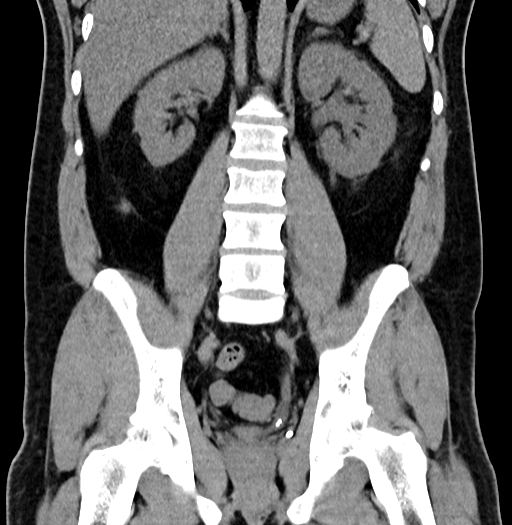

Supplement: Supplementary file 2 — Electronic Supplementary Material [file 330_2024_11115_MOESM2_ESM.zip › Digital Supplementary Material/Computed Tomography/39Computed Tomography.PNG]

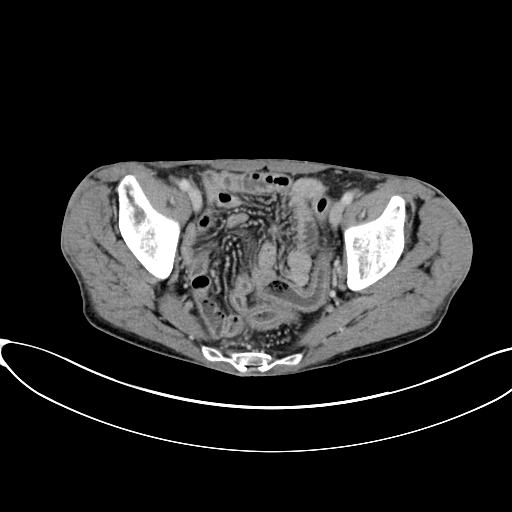

Supplement: Supplementary file 2 — Electronic Supplementary Material [file 330_2024_11115_MOESM2_ESM.zip › Digital Supplementary Material/Computed Tomography/41Computed Tomography.PNG]

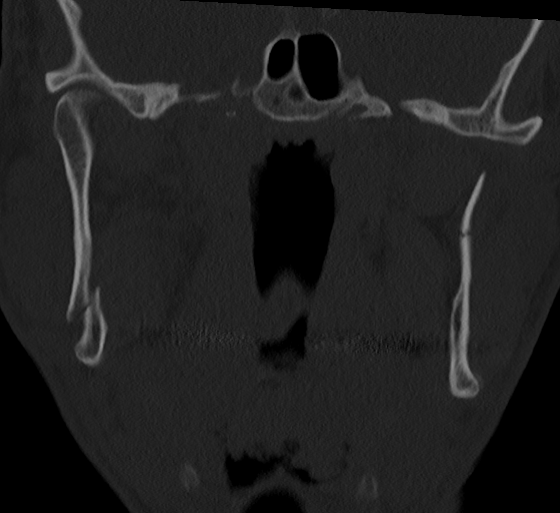

Supplement: Supplementary file 2 — Electronic Supplementary Material [file 330_2024_11115_MOESM2_ESM.zip › Digital Supplementary Material/Computed Tomography/34Computed Tomography.PNG]
